# Short-Chain Oleanolic Acid Esters and Furoyl Hybrids: Pharmacological Prediction, ADMETox Profiling, In Vitro Cytotoxicity Evaluation, Antioxidant Testing and EGFR Docking

**DOI:** 10.3390/pharmaceutics18070832

**Published:** 2026-07-07

**Authors:** Barbara Bednarczyk-Cwynar, Piotr Ruszkowski, Maciej Kulawik, Szymon Sip, Przemysław Zalewski, Dobrosława Wiśniewska, Andrzej Günther

**Affiliations:** 1 Department of Organic Chemistry, Faculty of Pharmacy, Poznan University of Medical Sciences, Collegium Pharmaceuticum 2, Rokietnicka Str. 3, 60-806 Poznan, Poland; 89387@student.ump.edu.pl (D.W.); andrzej.gunther@me.pl (A.G.); 2 Center of Innovative Pharmaceutical Technology, Rokietnicka Str. 3, 60-806 Poznan, Poland; 3 Department of Pharmacology, Faculty of Pharmacy, Poznan University of Medical Sciences, Collegium Pharmaceuticum 1, Rokietnicka Str. 3, 60-806 Poznan, Poland; pruszkowski@gmail.com; 4 Department of Pharmacognosy and Biomaterials, Faculty of Pharmacy, Poznan University of Medical Sciences, Collegium Pharmaceuticum 1, Rokietnicka Str. 3, 60-806 Poznan, Poland; maciej.kulawik@student.ump.edu.pl (M.K.); szymonsip@ump.edu.pl (S.S.); pzalewski@ump.edu.pl (P.Z.); 5 Doctoral School, Poznan University of Medical Sciences, Bukowska Str. 70, 60-812 Poznan, Poland

**Keywords:** oleanolic acid, triterpene derivatives, 3-O-furoyl hybrids, structure–activity relationship, cytotoxic activity, Selectivity Index, molecular docking, ADMETox prediction, antioxidant assays

## Abstract

**Background/Objectives:** This study aimed to improve the biological profile of oleanolic acid (OA) through structural modification at the C-17 carboxyl group and the C-3 hydroxyl group, with a focus on the design of short-chain alkyl esters and 3-O-furoyl hybrids. **Methods**: Two series of OA derivatives were synthesized and characterized using spectroscopic methods, including ^1^H NMR, ^13^C NMR and MS. In silico structure–activity relationship (SAR) analysis, ADMETox profiling, and molecular docking to the epidermal growth factor receptor (EGFR) tyrosine kinase domain were performed as predictive and hypothesis-generating tools. Anticancer activity was evaluated in vitro using the MTT assay against human cancer cell lines, including HeLa, MCF-7, A-549, SKBR-3, PC-3 and SKOV-3, as well as non-malignant human dermal fibroblasts (HDFs). Antioxidant properties were assessed using cell-free CUPRAC and DPPH assays. **Results**: The C-17 esterification markedly enhanced cytotoxic potency compared to the parent OA, while the introduction of the 3-O-furoyl moiety further improved antiproliferative activity in several derivatives. Selected compounds showed low-micromolar IC_50_ values and moderate selectivity toward cancer cells. Molecular docking suggested favorable accommodation of selected derivatives within the EGFR ATP-binding pocket, mainly through hydrophobic and π-related interactions; however, these results do not confirm direct EGFR binding and require experimental validation. The CUPRAC and DPPH assays provided preliminary insight into chemical redox behavior but should not be directly extrapolated to intracellular antioxidant or pro-oxidant activity. Predicted ADMETox profiles indicated moderate permeability and relatively low predicted risk for selected toxicity endpoints, while also highlighting high lipophilicity, poor aqueous solubility and potential metabolic liabilities. **Conclusions**: Overall, the results identify several OA derivatives as promising anticancer lead compounds for further optimization and mechanistic investigation.

## 1. Introduction

Oleanolic acid (OA) is a naturally occurring pentacyclic triterpenoid belonging to the oleanane-type skeleton and is widely distributed throughout the plant kingdom. It has been identified in more than 1600 species of medicinal and edible plants, including *Olea europaea* [1], *Calendula officinalis* [2], *Viscum album* [3], *Ligustrum lucidum* [4], and *Panax* species [5]. Due to its abundance, low toxicity, and well-defined chemical structure, oleanolic acid has attracted significant attention as a versatile scaffold for the development of new pharmacologically active compounds (e.g., refs. [6,7,8]).

Over the past decades, extensive pharmacological studies have demonstrated that oleanolic acid exhibits a broad spectrum of biological activities, including anti-inflammatory [9], antidiabetic and antioxidant [4], hepatoprotective [10], antiviral [11], antibacterial and antiparasitic [2], neuroprotective [12], gastroprotective [13], and cardioprotective effects [14]. Among these properties, its anticancer activity has been particularly intensively investigated and considered one of the most promising directions for its therapeutic application (e.g., refs. [15,16,17,18]).

Numerous in vitro and in vivo studies have shown that oleanolic acid can inhibit proliferation and induce apoptosis in various cancer cell lines, including breast [19], colon [20], liver [21], lung [22], cervical [23], prostate [24], and ovarian cancer types [25]. Mechanistic investigations have shown that these effects are associated with modulation of multiple signaling pathways involved in cancer progression, such as PI3K/Akt (crucial intracellular signaling cascade that regulates cell survival, growth, and proliferation), mitogen-activated protein kinase (MAPK), nuclear factor kappa B (NF-κB), signal transducer and activator of transcription 3 (STAT3), and tumor protein p53 (p53) pathways, as well as the induction of mitochondrial dysfunction and reactive oxygen species (ROS) generation [26]. Importantly, oleanolic acid exhibits relatively low cytotoxicity toward non-malignant cells, which makes it an attractive lead compound for anticancer drug development [21,27].

Accumulating evidence indicates that OA and selected OA derivatives may influence cellular redox homeostasis, although the direction and biological consequences of these effects are strongly dependent on compound structure, cell type and experimental conditions [26,28,29,30,31]. ROS play a dual role in cancer biology. At moderate levels, they participate in intracellular signaling, whereas excessive ROS production may contribute to oxidative stress, deoxyribonucleic acid (DNA) damage, lipid peroxidation and genomic instability [32,33,34]. Cancer cells often maintain elevated basal ROS levels and may therefore be more vulnerable to compounds that disturb redox balance. However, redox modulation cannot be reliably inferred solely from cell-free antioxidant assays [28,35,36]. In the present study, 2,2-diphenyl-1-picrylhydrazyl (DPPH) and cupric reducing antioxidant capacity (CUPRAC) assays were used only to characterize the chemical redox behavior of the synthesized derivatives, whereas intracellular ROS levels were not directly measured. Therefore, any connection between redox properties and cytotoxicity should be considered hypothetical and requires further experimental validation.

Numerous studies have demonstrated that triterpenes, including oleanolic acid, exhibit significant antioxidant activity through free radical scavenging, inhibition of lipid peroxidation, metal ion chelation, and regulation of endogenous antioxidant enzymes [26,28,31]. Structural modification of the oleanolic acid scaffold, especially at the C-17 carboxyl group, has frequently resulted in derivatives with enhanced antioxidant activity in standard in vitro assays. This enhanced antioxidant activity has been reported to correlate with improved anticancer effects and supports the role of redox modulation as an important component of their mechanism of action [26,31].

Despite its promising biological profile, the clinical application of oleanolic acid is limited by its low permeability, low aqueous solubility, and suboptimal bioavailability [37]. Consequently, numerous efforts have been undertaken to improve its pharmacological properties through chemical modification. Structure–activity relationship (SAR) studies have demonstrated that structural transformations, particularly at the C-3 hydroxyl group and the C-17 carboxyl group, can significantly enhance anticancer activity. Among these strategies, esterification or amidation of the C-17 carboxyl group has proven to be especially effective, often resulting in derivatives with markedly increased cytotoxicity and improved selectivity toward cancer cells (e.g., refs. [6,7,8,38]). Previous studies have shown that modification of the C-17 carboxyl group can improve the biological performance of OA derivatives by increasing lipophilicity, membrane affinity and cellular uptake. C-3 modification, in turn, may influence molecular conformation, hydrogen-bonding capacity, polarity and interactions with biological targets. However, many previously reported analogues were evaluated as isolated structures or within limited derivative series. The present work was designed to provide a systematic comparison between short-chain C-17 alkyl esters and their corresponding 3-O-furoyl analogues. This paired design enables direct evaluation of how C-3 furoylation modifies cytotoxicity, selectivity, redox behavior and predicted developability relative to the non-acylated esters.

In parallel with advances in triterpene chemistry, furoic acid (furan-2-carboxylic acid) has emerged as a biologically relevant heteroaromatic compound with documented anticancer activity. Several recent studies have reported that furoic acid and its derivatives exhibit cytotoxic effects against a range of human cancer cell lines, including breast [39], colon, lung [40], and cholangiocarcinoma cell lines [41]. The anticancer mechanisms of furoic-acid-based compounds have been linked to apoptosis induction, oxidative stress modulation, mitochondrial impairment, and interference with DNA replication and repair processes. These findings indicate that furoic acid represents a promising pharmacophoric moiety for anticancer drug design.

The conjugation of natural product scaffolds with biologically active small molecules is a well-established medicinal chemistry strategy aimed at enhancing anticancer potency, improving pharmacokinetic properties, or achieving synergistic biological effects. In the case of oleanolic acid, hybrid molecules obtained through conjugation with heterocyclic or aromatic carboxylic acids have frequently demonstrated superior anticancer activity compared to the parent compound (e.g., refs. [42,43,44,45,46]).

Based on these considerations, we hypothesized that conjugation of the OA scaffold with a furoyl moiety could generate derivatives with improved anticancer activity compared with the corresponding non-acylated esters. Therefore, the aim of the present study was to synthesize two related series of OA derivatives, namely short-chain C-17 esters and their 3-*O*-furoyl counterparts, and to compare their cytotoxicity, selectivity toward malignant versus non-malignant cells, chemical redox behavior, predicted absorption, distribution, metabolism, excretion, and toxicity (ADMETox) properties and possible epidermal growth factor receptor (EGFR) binding modes. The study was intended as an integrated medicinal chemistry evaluation. The docking and ADMETox analyses were used as predictive tools and do not replace experimental target-engagement or pharmacokinetic studies.

To the best of our knowledge, the present study represents the first report of triterpenoid derivatives—not limited to oleanolic acid—in which a furoyl moiety is introduced at the C-3 position of the triterpene scaffold. This structural concept constitutes a significant innovation in triterpenoid chemistry, as such modification has not been previously described. The incorporation of a furoyl group into the triterpene framework opens a new direction in the design of hybrid molecules, combining a privileged natural scaffold with an additional bioactive heteroaromatic pharmacophore. Consequently, this approach not only expands the chemical diversity of triterpenoids but may also initiate a new line of research focused on furoyl-functionalized triterpenes, with potential implications for anticancer drug development. We believe that this contribution represents a substantial advancement in the field of triterpenoid-based medicinal chemistry, providing both a novel structural platform and a foundation for further investigation.

## 2. Materials and Methods

### 2.1. Synthesis

#### 2.1.1. General Information

All solvents (analytical grade or higher) were purchased from Chempur (Piekary Śląskie, Poland) or POCh (Gliwice), Poland, while all other chemical reagents were purchased from Merck KgaA (Darmstadt, Germany).

Thin-layer chromatography (TLC) was used to monitor reaction progress and assess compound purity. Analyses were performed on high-performance thin-layer chromatography (HPTLC) aluminum plates coated with silica gel 60 F_254_ (Merck KGaA, Darmstadt, Germany; HPTLC Alufolien, Art. 5547). Elution was carried out using benzene and ethyl acetate in various proportions. Developed chromatograms were visualized by spraying with a 10% ethanol solution of sulfuric acid, followed by heating at approximately 110 °C for several minutes.

Melting points were determined using open capillaries with a Büchi melting point apparatus.

Proton nuclear magnetic resonance (^1^H NMR) spectra and carbon nuclear magnetic resonance (^13^C NMR) spectra were recorded in deuterated chloroform (CDCl_3_) on a Varian Gemini 400 VT (Bruker, Poznań, Polska) spectrometer operating at 400 megahertz (MHz) for proton and 100 MHz for carbon nuclei. Tetramethylsilane (TMS) was used as the internal standard. Chemical shift values (δ) are reported with a precision of ±0.01 ppm (part per million) for ^1^H NMR and for ^13^C NMR spectra. Coupling constants (*J*) are given in ±0.1 Hz (hertz). Signal multiplicities in the ^1^H NMR spectra are defined as follows (in alphabetical order): d = doublet, dd = doublet of doublets, ddt = doublet of doublets of doublets, dt = doublet of triplets, dq = doublet of quartets, m = multiplet, p = pentet, q = quartet, s = singlet, t = triplet, td = triplet of doublets, tt = triplet of triplets. The abbreviation “Fur” refers to the furoyl moiety, whereas “TT” refers to the triterpene skeleton.

#### 2.1.2. General Method of Oleanolic Acid Ester Formation

Oleanolic acid esters **2a**–**2j** were prepared according to a previously reported procedure [47].

**Methyl ester of oleanolic acid (methyl oleanolate, compound 2a)**: the ester was prepared using 170 mg (1.2 mmol) of methyl iodide. **Mol. formula**: C_31_H_50_O_3_. **Mol. mass**: 470.3760. **Yield**: Ref. [48]. **M.p.**: Ref. [48]. **R_f_**: Table 1. **^13^C NMR** (δ): 178.29 (C_q_, C-28); 143.72 (C_q_, C-13); 122.29 (CH, C-12); 78.94 (CH, C-3); 51.52 (CH_3_, -COO-**CH_3_**); 46.66 (C_q_, C-17). **^1^H NMR** (δ): 5.29 (1H, t, *J* = 3.6 Hz, C_12_-H); 3.62 (3H, s, -COO-**CH_3_**); 3.21 (1H, dd, *J* = 4.9 and 11.0 Hz, C_3_-H_α_); 2.86 (1H, dd, *J* = 4.7 and 14.0 Hz, C_18_-H_β_); 1.13, 0.99, 0.92, 0.90 × 2, 0.78, 0.72 (5 × 3H + 1 × 6H, 6 × s, 7 × CH_3_ groups of oleanane skeleton). **MS**: 470.7 (19.8%, M^•+^).

**Ethyl ester of oleanolic acid (ethyl oleanolate, compound 2b)**: the ester was prepared using 131 mg (1.2 mmol) of ethyl bromide. **Mol. formula**: C_32_H_52_O_3_. **Mol. mass**: 484.3916. **Yield**: Ref. [47]. **M.p.**: Ref. [47]; lit. m.p. = 175–178 °C [49], m.p. 175–177 [50]; **R_f_**: Table 1. **^13^C NMR** (δ): 177.69 (C_q_, C-28), 143.76 (C_q_, C-13), 122.36 (CH, C-12), 78.95 (CH, C-3), 60.05 (CH_2_, -COO-**CH_2_**-CH_3_), 45.88 (C_q_, C-17), 14.24 (CH_3_, -COO-CH_2_-**CH_3_**). **^1^H NMR** (δ): 5.28 (1H, t, *J* = 3.7 Hz, C_12_-H), 4.19–3.97 (2H, m, -COO-**CH_2_**-CH_3_), 3.21 (1H, dd, *J* = 10.4 and 5.1 Hz, C_3_-H_α_), 2.86 (1H, dd, *J* = 14.2 and 4.1 Hz, C_18_-H_β_), 1.23 (3H, t, *J* = 7.1 Hz, -COO-CH_2_-**CH_3_**), 1.13, 0.99, 0.92, 0.90 × 2, 0.78, 0.74 (5 × 3H + 1 × 6H, 6 × s, 7 × CH_3_ groups of oleanane skeleton). **MS**: 484.4 (6.2%, M^•+^).

***n*-Propyl ester of oleanolic acid (*n*-propyl oleanolate, compound 2c)**: the ester was prepared using 147 mg (1.2 mmol) of *n*-propyl bromide (=1-bromopropane). **Mol. formula**: C_33_H_54_O_3_. **Mol. mass**: 498.407296. **Yield**: 472 mg (94.8%). **M.p.**: 110–112 °C (EtOH). **R_f_**: Table 1. **^13^C NMR** (δ): 177.75 (C_q_, C-28); 143.77 (C_q_, C-13); 122.30 (CH, C-12); 78.95 (CH, C-3); 65.77 (CH_2_, -COO-**CH_2_**-CH_2_-CH_3_); 58.35 (traces, CH_2_, CH_3_-**CH_2_**-OH); 46.64 (C_q_, C-17); 21.96 (CH_2_, -COO-CH_2_-**CH_2_**-CH_3_); 18.38 (traces, CH_3_, **CH_3_**-CH_2_-OH); 10.55 (CH_3_, -COO-CH_2_-CH_2_-**CH_3_**). **^1^H NMR** (δ): 5.28 (1H, t, *J* = 3.8 Hz, C_12_-H), 4.00–3.95 (2H, m, -COO-**CH_2_**-CH_2_-CH_3_), 3.72 (traces, dd, *J* = 7.0 and 14.0 Hz, CH_3_-**CH_2_**-OH), 3.21 (1H, dd, *J* = 11.1 and 4.9 Hz, C_3_-H_α_), 2.88 (1H, dd, *J* = 14.0 and 4.9 Hz, C_18_-H_β_), 1.64–1.60 (2H, m, -COO-CH_2_-**CH_2_**-CH_3_); 1.24 (traces, t, *J* = 7.0 Hz, **CH_3_**-CH_2_-OH); 1.13, 0.99, 0.94, 0.93, 0.90 × 2, 0.78, 0.73 (6 × 3H + 1 × 6H, 7 × s, 7 × CH_3_ groups of oleanane skeleton) + -COO-CH_2_-CH_2_-**CH_3_**). **MS**: 498.6 (25.6%, M^•+^).

**Allyl ester of oleanolic acid (allyl oleanolate, compound 2d)**: the ester was prepared using 145 mg (1.2 mmol) of allyl bromide (=3-bromo-1-propene). **Mol. formula**: C_33_H_52_O_3_. **Mol. mass**: 496.391646. **Yield**: 481 mg (96.6%). **M.p.**: 155–156 °C (EtOH), lit. mp: 148–150 °C [51]. **R_f_**: Table 1. **^13^C NMR** (δ): 177.40 (C_q_, C-28), 143.68 (C_q_, C-13), 132.53 (CH, -COO-CH_2_-**CH**=CH_2_), 122.44 (CH, C-12), 117.71 (CH_2_, -COO-CH_2_-CH=**CH_2_**), 78.96 (CH, C-3), 64.81 (CH_2_, -COO-**CH_2_**-CH=CH_2_), 58.30 (traces, CH_3_-**CH_2_**-OH), 46.75 (C_q_, C-17), 18.39 (traces, **CH_3_**-CH_2_-OH). **^1^H NMR** (δ): 5.96–5.82 (1H, m, -COO-CH_2_-**CH**=CH_2_), 5.31 (2H, dq + t, for dq: *J* = 18.8 and 3.3 Hz, -COO-CH_2_-CH=**CH_2_**; for t: *J* = 1.6 Hz, C_12_-H), 5.20 (1H, dq, *J* = 10.5 and 1.4 Hz, -COO-CH_2_-CH=**CH_2_**), 4.52 (2H, tt, *J* = 5.5 and 1.5 Hz, -COO-**CH_2_**-CH=CH_2_), 3.70 (traces, dd, *J* = 7.0 and 14.0 Hz, CH_3_-**CH_2_**-OH), 3.20 (1H, dd, *J* = 11.0 and 5.0 Hz, C_3_-H_α_), 2.88 (1H, dd, *J* = 13.9 and 4.1 Hz, C_18_-H_β_), 1.23 (traces, t, *J* = 7.0 Hz, **CH_3_**-CH_2_-OH), 1.13, 0.98, 0.92, 0.90 × 2, 0.77, 0.72 (5 × 3H + 1 × 6H, 6 × s, 7 × CH_3_ groups of oleanane skeleton). **MS**: 496.5 (15.8%, M^•+^).

**Propargyl ester of oleanolic acid (propargyl oleanolate, compound 2e)**: the ester was prepared using 143 mg (1.2 mmol) of propargyl bromide (=3-bromo-1-propyne). **Mol. formula**: C_33_H_50_O_3_. **Mol. mass**: 494.375996. **Yield**: 482 mg (97.5%). **M.p.**: 132–135 °C (EtOH), lit. m.p.: mp 158−160 °C [52], 90–92 °C [53]. **R_f_**: Table 1. **^13^C NMR** (δ): 176.84 (C_q_, C-28), 143.38 (C_q_, C-13), 122.63 (CH, C-12), 79.00 (CH, C-3), 78.13 (C_q_, -COO-CH_2_-**C**≡CH), 74.44 (CH, -COO-CH_2_-C≡**CH**), 58.30 (traces, CH_3_-**CH_2_**-OH), 51.65 (CH_2_, -COO-**CH_2_**-C≡CH), 46.78 (C_q_, C-17), 18.39 (traces, **CH_3_**-CH_2_-OH). **^1^H NMR** (δ): 5.30 (1H, t, *J* = 3.8 Hz, C_12_-H), 4.68 (1H, dd, *J* = 15.6 and 2.5 Hz, -COO-**CH_2_**-C≡CH), 4.58 (1H, dd, *J* = 15.6 and 2.5 Hz, -COO-**CH_2_**-C≡CH), 3.72 (weak, dd, *J* = 7.0 and 14.0 Hz, CH_3_-**CH_2_**-OH), 3.21 (1H, dd, *J* = 11.1 and 4.9 Hz, C_3_-H_α_), 2.87 (dd, *J* = 13.9 and 4.9 Hz, C_18_-H_β_), 2.43 (1H, t, *J* = 2.4 Hz, -COO-CH_2_-C≡**CH**), 1.23 (traces, t, *J* = 7.0 Hz, **CH_3_**-CH_2_-OH), 1.14, 0.98, 0.93, 0.90 × 2, 0.78, 0.75 (5 × 3H + 1 × 6H, 6 × s, 7 × CH_3_ groups of oleanane skeleton). **MS**: 494.5 (22.2%, M^•+^).

**Isopropyl ester of oleanolic acid (isopropyl oleanolate, compound 2f)**: the ester was prepared using 147 mg (1.2 mmol) of isopropyl bromide (=2-bromopropane). **Mol. formula**: C_33_H_54_O_3_. **Mol. mass**: 498.407296. **Yield**: 486 mg (97.5%). **M.p.**: 99–101 °C (EtOH). **R_f_**: Table 1. **^13^C NMR** (δ): 177.04 (C_q_, C-28), 143.70 (C_q_, C-13), 122.19 (CH, C-12), 78.91 (CH, C-3), 66.98 (CH, -COO-**CH**-[CH_3_]_2_), 58.31 (weak, CH_2_, CH_3_-**CH_2_**-OH), 46.29 (C_q_, C-17), 21.74 and 21.70 (CH_3_, -COO-CH_2_-**[CH_3_]_2_**), 18.36 (weak, CH_2_, **CH_3_**-CH_2_-OH).^**1**^**H NMR** (δ): 5.29 (1H, t, *J* = 3.8 Hz, C_12_-H), 4.94 (1H, p, *J* = 6.3 Hz, -COO-**CH**-[CH_3_]_2_), 3.71 (weak, dd, *J* = 7.0 and 14.0 Hz, CH_3_-**CH_2_**-OH), 3.21 (1H, dd, *J* = 10.9 and 5.0 Hz, C_3_-H_α_), 2.86 (1H, dd, *J* = 14.0 and 5.0 Hz, C_18_-H_β_), 1.24 (weak, t, *J* = 7.0 Hz, **CH_3_**-CH_2_-OH); 1.19 (6H, t, *J* = 5.7 Hz, -COO-CH_2_-**[CH_3_]_2_**); 1.13, 0.99, 0.92, 0.90, 0.89, 0.78, 0.76 (7 × 3H, 7 × s, 7 × CH_3_ group). **MS**: 498.5 (24.2%, M^•+^).

***n*-Butyl ester of oleanolic acid (*n*-butyl oleanolate, compound 2g)**: the ester was prepared using 164 mg (1.2 mmol) of *n*-butyl bromide (=1-bromobutane). **Mol. formula**: C_34_H_56_O_3_. **Mol. mass**: 512.422946. **Yield**: 494 mg (96.5%). **M.p.**: 114–116 °C (EtOH + H_2_O). **R_f_**: Table 1. **^13^C NMR** (δ): 177.73 (C_q_, C-28), 143.78 (C_q_, C-13), 122.32 (CH, C-12), 78.95 (CH, C-3), 63.94 (CH_2_, -COO-**CH_2_**-CH_2_-CH_2_-CH_3_), 46.62 (C_q_, C-17), 33.10 (CH_2_, -COO-CH_2_-**CH_2_**-CH_2_-CH_3_), 19.22 (CH_2_, -COO-CH_2_-CH_2_-**CH_2_**-CH_3_), 13.70 (CH_3_, -COO-CH_2_-CH_2_-CH_2_-**CH_3_**). **^1^H NMR** (δ): 5.28 (1H, t, *J* = 3.5 Hz, C_12_-H), 4.04 (2H, td, *J* = 6.4 and 0.5 Hz, -COO-**CH_2_**-CH_2_-CH_2_-CH_3_), 3.21 (1H, dd, *J* = 10.4 and 5.1 Hz, C_3_-H_α_), 2.87 (1H, dd, *J* = 13.9 and 4.2 Hz, C_18_-H_β_), 1.55–1.51 (2H, m, -COO-CH_2_-**CH_2_**-CH_2_-CH_3_), 1.36–1.28 (2H, m, -COO-CH_2_-CH_2_-**CH_2_**-CH_3_), 1.13, 0.99, 0.93, 0.92, 0.91, 0.90, 0.78, 0.74 (8 × CH3, 8 × s, 7 × 3H, 7 × s, 7 × CH_3_ group of oleanane skeleton and -COO-CH_2_-CH_2_-CH_2_-**CH_3_**). **MS**: 512.6 (22.0%, M^•+^).

**Isobutyl ester of oleanolic acid (isobutyl oleanolate, compound 2h)**: the ester was prepared using 164 mg (1.2 mmol) of isobutyl bromide (=1-bromo-2-methylpropane). **Mol. formula**: C_34_H_56_O_3_. **Mol. mass**: 512.422946. **Yield**: 493 mg (96.3%). **M.p.**: 200–203 °C (EtOH). **R_f_**: Table 1. **^13^C NMR** (δ): 177.71 (C_q_, C-28), 143.81 (C_q_, C-13), 122.39 (CH, C-12), 78.98 (CH, C-3), 70.39 (CH_2_, -COO-**CH_2_**-CH-[CH_3_]_2_), 46.78 (C_q_, C-17), 28.10 (CH_2_, -COO-CH_2_-**CH**-[CH_3_]_2_), 19.20 and 19.18 (CH_3_, -COO-CH_2_-CH-**[CH_3_]_2_**). **^1^H NMR** (δ): 5.28 (1H, t, *J* = 3.6 Hz, C_12_-H), 3.79 (2H, dd, *J* = 6.4, 3.5 Hz, -COO-**CH_2_**-CH-[CH_3_]_2_), 3.21 (1H, dd, *J* = 10.5 and 5.3 Hz, C_3_-H_α_), 2.89 (1H, dd, *J* = 13.6 and 4.3 Hz, C_18_-H_β_), 1.85 (1H, dd, *J* = 5.3 and 2.7 Hz, -COO-CH_2_-**CH**-[CH_3_]_2_); 1.13; 0.99, 0.95, 0.93 × 2, 0.90 × 2, 0.78, 0.73 (5 × 3H + 2 × 6H, 7 × s, 7 × CH_3_ group of oleanane skeleton + -COO-CH_2_-CH-**[CH_3_]_2_**). **MS**: 512.7 (26.4%, M^•+^).

***sec*-Butyl ester of oleanolic acid (*sec*-butyl oleanolate, compound 2i)**: the ester was prepared using 164 mg (1.2 mmol) of sec-butyl bromide (=2-bromobutane). **Mol. formula**: C_34_H_56_O_3_. **Mol. mass**: 512.422946. **Yield**: 489 mg (95.5%). **M.p.**: 152–154 °C (EtOH + H_2_O). **R_f_**: Table 1. **^13^C NMR** (δ): 177.24 (C_q_, C-28), 143.80 (C_q_, C-13), 122.25 (CH, C-12), 79.01 (CH, C-3), 71.75 (CH, -COO-**CH**(CH_3_)-CH_2_-CH_3_), 46.60 (C_q_, C-17), 28.77 (CH_2_, -COO-CH(CH_3_)-**CH_2_**-CH_3_), 19.43 (CH_3_, -COO-CH(**CH_3_**)-CH_2_-CH_3_), 9.77 (CH_3_, -COO-CH(CH_3_)-CH_2_-**CH_3_**). **^1^H NMR** (δ): 5.30 (1H, t, *J* = 3.6 Hz, C_12_-H), 4.82–4.74 (1H, m, -COO-**CH**(CH_3_)-CH_2_-CH_3_), 3.23 (1H, dd, *J* = 11.1 and 3.3 Hz, C_3_-H_α_), 2.89 (1H, dt, *J* = 14.1 and 4.2 Hz, C_18_-H_β_), 1.93–1.86 (2H, m, -COO-CH(CH_3_)-**CH_2_**-CH_3_); 1.19 (3H, t, *J* = 6.7 Hz, -COO-CH(**CH_3_**)-CH_2_-CH_3_), 1.15 × 2, 1.00, 0.93, 0.92, 0.91, 0.79 (5 × 3H + 1 × 6H, 6 × s, 7 × CH_3_ group of oleanane skeleton), 0.78 (3H, d, *J* = 1.9 Hz, -COO-CH(CH_3_)-CH_2_-**CH_3_**). **MS**: 512.5 (22.0%, M^•+^).

**2,3-Dichloropropyl ester of oleanolic acid (2,3-dichloropropyl oleanolate, compound 2j)**: the ester was prepared using 177 mg (1.2 mmol) of 1,2,3-trichloropropane. **Mol. formula**: C_33_H_52_Cl_2_O_3_. **Mol. mass**: 566.329351. **Yield**: 532 mg (93.9%). **M.p.**: 153–154 °C (EtOH + H_2_O). **R_f_**: Table 1. **^13^C NMR** (δ): 176.81 (C_q_, C-28), 143.48 (C_q_, C-13), 136.24 (CH, -COO-CH_2_-**CH**(Cl)-CH_2_-Cl), 122.68 (CH, C-12), 114.83 (CH_2_, -COO-CH_2_-CH(Cl)-**CH_2_**-Cl), 78.99 (CH, C-3), 65.85 (CH_2_, -COO-**CH_2_**-CH(Cl)-CH_2_-Cl), 58.43 (traces, CH_2_, CH_3_-**CH_2_**-OH), 46.91 (C_q_, C-17), 18.45 (traces, CH_2_, **CH_3_**-CH_2_-OH). **^1^H NMR** (δ): 5.48 (1H, q, *J* = 1.5 Hz, -COO-CH_2_-CH(Cl)-**CH_2_**-Cl) and 5.39 (1H, d, *J* = 1.8 Hz, -COO-CH_2_-CH(Cl)-**CH_2_**-Cl), 5.31 (1H, t, *J* = 3.8 Hz, C_12_-H), 4.59 (2H, dd, *J* = 13.8 and 7.5 Hz, -COO-**CH_2_**-CH(Cl)-CH_2_-Cl), 3.72 (weak, dd, *J* = 7.0 and 14.0 Hz, CH_3_-**CH_2_**-OH), 3.22 (1H, dd, *J* = 11.1 and 4.9 Hz, C_3_-H_α_), 2.89 (1H, dd, *J* = 13.7 and 4.9 Hz, C_18_-H_β_), 2.01 (1H, td, *J* = 13.7 and 4.2 Hz, -COO-CH_2_-**CH**(Cl)-CH_2_-Cl), 1.24 (weak, t, *J* = 7.0 Hz, **CH_3_**-CH_2_-OH), 1.15, 0.99, 0.94, 0.91 × 2, 0.79, 0.73 (5 × 3H + 1 × 6H, 6 × s, 7 × CH_3_ group of oleanane skeleton). **MS**: 566.4 (19.6%, M^•+^).

**Erythrodiol (compound 2k)**: erythrodiol was prepared from oleanolic acid or its methyl ester according to the procedure given in our earlier publication [54]. **Mol. formula**: C_30_H_50_O_2_. **Mol. mass**: 442.381081. **Yield**: 427 mg (96.6%). **M.p.**: [54], lit. m.p. = 226–228 °C [49]. **R_f_**: Table 1. **^13^C NMR** (δ): 145.83 (C_q_, C-13), 123.95 (CH, C-12), 78.98 (CH, C-3), 71.28 (CH_2_, C-28), 38.52 (C_q_, C-17). **^1^H NMR** (δ): 5.18 (1H, t, *J* = 3.4 Hz, C_12_-H), 3.68–3.60 (1H, m, C_28_-H_a_) and 3.53 (1H, d, *J* = 11.7 Hz, C_28_-H_b_), 3.17 (1H, dd, *J* = 14.1 and 8.0 Hz, C_3_-H_α_), 1.97 (1H, dd, *J* = 14.1 and 5.4 Hz, C_18_-H_β_), 1.16, 0.99, 0.94, 0.93, 0.88, 0.87, 0.78 (7 × 3H, 7 × s, 7 × CH_3_ group). **MS**: 442.5 (29.6%, M^•+^).

#### 2.1.3. General Method of Oleanolic Acid Esters and Erythrodiol Acylation with 2-Furoic Acid

A saturated solution of oleanolic acid esters (**2a**–**2k**, 1.0 mmol) in dioxane, stirred at room temperature, was treated with N,N′-dicyclohexylcarbodiimide (DCC; 2.0 mmol, 416 mg). After the complete dissolution, 4-(N,N-dimethylamine)pyridine (DMAP; 3.0 mmol, 366 mg) was added, and the mixture was stirred for about 30 min. Subsequently, 2-furoic acid (2.0 mmol, 224 mg) was added, and the reaction was stirred at room temperature for an additional 30 minutes under TLC monitoring. The reaction mixture was filtered, and the filtrate was poured into about five volumes of water slightly acidified with hydrochloric acid (HCl). The resulting precipitate was collected by (i) filtration and washed with water or (ii) extracted with dichloromethane (CH_2_Cl_2_), dried over anhydrous magnesium sulfate (MgSO_4_), and the solvent was evaporated under reduced pressure. The crude product was dissolved in a small amount of CH_2_Cl_2_ and purified by column chromatography on silica gel using CH_2_Cl_2_ as the eluent. The final product was recrystallized from ethanol (EtOH) or precipitated from an ethanolic solution by the addition of water.

**Methyl ester of 3-O-furoyloleanolic acid (methyl 3-O-furoyl oleanolate, compound 3a)**: the ester **3a** was prepared using 456 mg (1.0 mmol) of methyl oleanolate (**2a**). **Mol. formula**: C_36_H_52_O_5_. **Mol. mass**: 564.381475. **Yield**: 520 mg (92.2%). **M.p.**: 253–255 °C (EtOH). **R_f_**: Table 1. **^13^C NMR** (δ): 178.30 (C_q_, C-28), 158.67 (C_q_, Fur-**CO**O-TT), 146.14 (CH, **Fur**-COO-TT), 145.18 (CH, **Fur**-COO-TT), 143.81 (C_q_, C-13), 122.25 (CH, C-12), 117.35 (CH, **Fur**-COO-TT), 111.69 (CH, **Fur**-COO-TT), 81.61 (CH, C-3), 51.54 (CH_3_, -COOCH_3_), 46.72 (C_q_, C-17). **^1^H NMR** (δ): 7.58 (1H, dd, *J* = 1.8 and 0.9 Hz, **Fur**-COO-TT), 7.13 (1H, dd, *J* = 1.0 and 3.5 Hz, **Fur**-COO-TT), 6.50 (1H, dd, *J* = 3.4 and 1.8 Hz, **Fur**-COO-TT), 5.29 (1H, t, *J* = 3.8 Hz, C_12_-H), 4.73 (1H, dd, *J* = 6.4 and 10.0 Hz, C_3_-H_α_), 3.63 (3H, s, -COO-**CH_3_**), 2.87 (1H, dd, *J* = 13.8 and 4.8 Hz, C_18_-H_β_), 1.15, 0.97 × 2, 0.93 × 2, 0.91, 0.74 (3 × 3H + 2 × 6H, 5 × singlets, 7 × CH_3_ groups of oleanane skeleton). **MS**: 564.5 (14.2%, M^•+^).

**Ethyl ester of 3-O-furoyloleanolic acid (ethyl 3-O-furoyl oleanolate, compound 3b)**: the ester **3b** was prepared using 484 mg (1.0 mmol) of ethyl oleanolate (**2b**). **Mol. formula**: C_37_H_54_O_5_. **Mol. mass**: 578.397125. **Yield**: 534 mg (92.4%). **M.p.**: 189–191 °C (EtOH). **R_f_**: Table 1. **^13^C NMR** (δ): 177.72 (C_q_, C-28), 158.68 (C_q_, Fur-**CO**O-TT), 146.14 (CH, **Fur**-COO-TT), 145.18 (CH, **Fur**-COO-TT), 143.83 (C_q_, C-13), 122.19 (CH, C-12), 117.35 (CH, **Fur**-COO-TT), 111.68 (CH, **Fur**-COO-TT), 81.62 (CH, C-3), 60.07 (CH_2_, -COO-**CH_2_**-CH_3_), 46.49 (C_q_, C-17), 14.29 (CH_3_, -COO-CH_2_-**CH_3_**). **^1^H NMR** (δ): 7.58 (1H, dd, *J* = 1.8 and 0.9 Hz, **Fur**-COO-TT), 7.14 (1H, dd, *J* = 3.4 and 0.9 Hz, **Fur**-COO-TT), 6.50 (1H, dd, *J* = 3.5 and 1.8 Hz, **Fur**-COO-TT), 5.30 (1H, t, *J* = 3.7 Hz, C_12_-H), 4.73 (1H, dd, *J* = 9.1 and 7.3 Hz, C_3_-H_α_), 4.14–4.03 (2H, m, -COO-**CH_2_**-CH_3_), 3.72 (traces, dd, *J* = 7.0 and 14.0 Hz, CH_3_-**CH_2_**-OH), 2.88 (1H, dd, *J* = 13.8 and 4.8 Hz, C_18_-H_β_), 1.24 (3H + traces, t, *J* = 7.1 Hz, -COO-CH_2_-**CH_3_** (3H) and traces of **CH_3_**-CH_2_-OH), 1.15, 0.97 × 2, 0.93 × 2, 0.91, 0.76 (3 × 3H + 2 × 6H, 5 × singlets, 7 × CH_3_ groups of oleanane skeleton). **MS**: 578.4 (11.9%, M^•+^).

***n*-Propyl ester of 3-O-furoyloleanolic acid (*n*-propyl 3-O-furoyl oleanolate, compound 3c)**: the ester **3c** was prepared using 498 mg (1.0 mmol) of *n*-propyl oleanolate (**2c**). **Mol. formula**: C_38_H_56_O_5_. **Mol. mass**: 592.412775. **Yield**: 547 mg (92.3%). **M.p.**: 152–155 °C (EtOH). **R_f_**: Table 1. **^13^C NMR** (δ): 177.80 (C_q_, C-28), 158.70 (C_q_, Fur-**CO**O-TT), 146.14 (CH, **Fur**-COO-TT), 145.18 (CH, **Fur**-COO-TT), 143.86 (C_q_, C-13), 122.24 (CH, C-12), 117.36 (CH, **Fur**-COO-TT), 111.69 (CH, **Fur**-COO-TT), 81.64 (CH, C-3), 65.80 (CH_2_, -COO-**CH_2_**-CH_2_-CH_3_), 46.69 (C_q_, C-17), 22.01 (CH_2_, -COO-CH_2_-**CH_2_**-CH_3_), 10.61 (CH_3_, -COO-CH_2_-CH_2_-**CH_3_**). **^1^H NMR** (δ): 7.58 (1H, dd, *J* = 1.9 and 0.9 Hz, **Fur**-COO-TT), 7.14 (1H, dd, *J* = 3.4 and 0.9 Hz, **Fur**-COO-TT), 6.51 (1H, dd, *J* = 3.5 and 1.8 Hz, **Fur**-COO-TT), 5.30 (1H, t, *J* = 3.8 Hz, C_12_-H), 4.73 (1H, dd, *J* = 9.1 and 7.3 Hz, C_3_-H_α_), 4.04–3.94 (2H, m, -COO-**CH_2_**-CH_2_-CH_3_), 3.72 (traces, dd, *J* = 7.0 and 14.0 Hz, CH_3_-**CH_2_**-OH), 2.89 (1H, dd, *J* = 13.8 and 4.8 Hz, C_18_-H_β_), 1.68–1.62 (2H, m, -COO-CH_2_-**CH_2_**-CH_3_); 1.24 (weak, t, *J* = 7.0 Hz, **CH_3_**-CH_2_-OH); 1.15, 0.97 × 2, 0.96, 0.94, 0.93, 0.91, 0.76 (6 × 3H + 1 × 6H, 7 singlets, 7 × CH_3_ groups of oleanane skeleton + -COO-CH_2_-CH_2_-**CH_3_**). **MS**: 592.5 (22.2%, M^•+^).

**Allyl ester of 3-O-furoyloleanolic acid (allyl 3-O-furoyl oleanolate, compound 3d)**: the ester **3d** was prepared using 496 mg (1.0 mmol) of allyl oleanolate (**2d**). **Mol. formula**: C_38_H_54_O_5_. **Mol. mass**: 590.397125. **Yield**: 569 mg (96.4%). **M.p.**: 184–186 °C (EtOH). **R_f_**: Table 1. **^13^C NMR** (δ): 177.36 (C_q_, C-28), 158.68 (C_q_, Fur-**CO**O-TT), 146.14 (CH, **Fur**-COO-TT), 145.18 (CH, **Fur**-COO-TT), 143.74 (C_q_, C-13), 132.56 (CH, -COO-CH_2_-**CH**=CH_2_), 122.33 (CH, C-12), 117.71 (CH_2_, -COO-CH_2_-CH=**CH_2_**), 117.35 (CH, **Fur**-COO-TT), 111.69 (CH, **Fur**-COO-TT), 81.61 (CH, C-3), 64.80 (CH_2_, -COO-**CH_2_**-CH=CH_2_), 46.75 (C_q_, C-17). **^1^H NMR** (δ): 7.58 (1H, dd, *J* = 1.8 and 0.9 Hz, **Fur**-COO-TT), 7.14 (1H, dd, *J* = 3.5 and 1.0 Hz, **Fur**-COO-TT), 6.50 (1H, dd, *J* = 3.5 and 1.8 Hz, **Fur**-COO-TT), 5.91 (1H, ddt, *J* = 17.1, 10.4 and 5.6 Hz, -COO-CH_2_-**CH**=CH_2_), 5.32 (2H, t + ddt, *J*_t_ = 1.7 Hz, C_12_-H; *J*_ddt_ = 17.2, 4.7 and 1.6 Hz, -COO-CH_2_-CH=**CH_2_**), 5.21 (1H, dq, *J* = 10.4 and 1.4 Hz, -COO-CH_2_-CH=**CH_2_**), 4.73 (1H, dd, *J* = 9.1 and 7.3 Hz, C_3_-H_α_), 4.54 (2H, tt, *J* = 4.9 and 1.4 Hz, -COO-**CH_2_**-CH=CH_2_), 3.72 (traces, dd, *J* = 7.0 and 14.0 Hz, CH_3_-**CH_2_**-OH), 2.90 (1H, dd, *J* = 13.9, and 4.8 Hz, C_18_-H_β_), 1.23 (traces, t, *J* = 7.0 Hz, **CH_3_**-CH_2_-OH), 1.15, 0.97 × 2, 0.94, 0.93, 0.91, 0.75 (5 × 3H + 1 × 6H, 6 × singlets, 7 × CH_3_ groups of oleanane skeleton). **MS**: 590.5 (13.5%, M^•+^).

**Propargyl ester of 3-O-furoyloleanolic acid (propargyl 3-O-furoyl oleanolate, compound 3e)**: the ester **3e** was prepared using 494 mg (1.0 mmol) of propargyl oleanolate (**2e**). **Mol. formula**: C_38_H_52_O_5_. **Mol. mass**: 588.381475. **Yield**: 534 mg (90.9%). **M.p**.: 259–260 °C (EtOH). **R_f_**: Table 1. **^13^C NMR** (δ): 176.88 (C_q_, C-28), 158.71 (C_q_, Fur-**CO**O-TT), 146.14 (CH, **Fur**-COO-TT), 145.20 (CH, **Fur**-COO-TT), 143.44 (C_q_, C-13), 122.54 (CH, C-12), 117.35 (CH, **Fur**-COO-TT), 111.69 (CH, **Fur**-COO-TT), 81.63 (CH, C-3), 78.15 (C_q_, -COO-CH_2_-**C**≡CH), 74.39 (CH, -COO-CH_2_-C≡**CH**), 51.65 (CH_2_, -COO-**CH_2_**-C≡CH), 46.80 (C_q_, C-17). **^1^H NMR** (δ): 7.60 (1H, dd, *J* = 1.8 and 0.9 Hz, **Fur**-COO-TT), 7.15 (1H, dd, *J* = 3.5 and 0.9 Hz, **Fur**-COO-TT), 6.52 (1H, dd, *J* = 3.5 and 1.8 Hz, **Fur**-COO-TT), 5.34 (1H, t, *J* = 3.6 Hz, C_12_-H), 4.75 (1H, dd, *J* = 9.1 and 7.3 Hz, C_3_-H_α_), 4.69 (1H, dd, *J* = 15.6 and 2.5 Hz, -COO-**CH_2_**-C≡CH), 4.61 (1H, dd, *J* = 15.5 and 2.5 Hz, -COO-**CH_2_**-C≡CH), 2.90 (1H, dd, *J* = 13.7 and 4.1 Hz, C_18_-H_β_), 2.44 (1H, t, *J* = 2.5 Hz, -COO-CH_2_-C≡**CH**), 1.17, 0.99, 0.98, 0.95 × 2, 0.93, 0.79 (5 × 3H + 1 × 6H, 6 × s, 7 × CH_3_ groups of oleanane skeleton). **MS**: 588.5 (19.9%, M^•+^).

**Isopropyl ester of 3-O-furoyloleanolic acid (isopropyl 3-O-furoyl oleanolate, compound 3f)**: the ester **3f** was prepared using 498 mg (1.0 mmol) of *iso*-propyl oleanolate (**2f**). **Mol. formula**: C_38_H_56_O_5_. **Mol. mass**: 592.412775. **Yield**: 537 mg (90.7%). **M.p.**: 192–193 °C (EtOH). **R_f_**: Table 1. **^13^C NMR** (δ): 177.10 (C_q_, C-28), 158.71 (C_q_, Fur-**CO**O-TT), 146.14 (CH, **Fur**-COO-TT), 145.19 (CH, **Fur**-COO-TT), 143.83 (C_q_, C-13), 122.16 (CH, C-12), 117.36 (CH, **Fur**-COO-TT), 111.69 (CH, **Fur**-COO-TT), 81.65 (CH, C-3), 67.03 (CH, -COO-**CH**-[CH_3_]_2_), 46.38 (C_q_, C-17), 21.82 and 21.77 (CH_3_, -COO-CH_2_-**[CH_3_]_2_**). **^1^H NMR** (δ): 7.59 (1H, dd, *J* = 1.8 and 0.9 Hz, **Fur**-COO-TT), 7.15 (1H, dd, *J* = 3.5 and 0.9 Hz, **Fur**-COO-TT), 6.51 (1H, dd, *J* = 3.4 and 1.8 Hz, **Fur**-COO-TT), 5.31 (1H, t, *J* = 3.6 Hz, C_12_-H), 4.96 (1H, dd, *J* = 12.5 and 6.3 Hz, -COO-**CH**-[CH_3_]_2_), 4.75 (1H, dd, *J* = 9.1 and 7.3 Hz, C_3_-H_α_), 2.89 (1H, dd, *J* = 13.9 and 4.4 Hz, C_18_-H_β_), 1.23 (6H, t, *J* = 5.7 Hz, -COO-CH_2_-**[CH_3_]_2_**); 1.20, 1.16, 0.98 × 2, 0.94, 0.92, 0.79 (5 × 3H + 1 × 6H, 6 × s, 7 × CH_3_ groups of oleanane skeleton). **MS**: 592.4 (16.7%, M^•+^).

***n*-Butyl ester of 3-O-furoyloleanolic acid (*n*-butyl 3-O-furoyl oleanolate, compound 3g)**: the ester **3g** was prepared using 512 mg (1.0 mmol) of *n*-butyl oleanolate (**2g**). **Mol. formula**: C_39_H_58_O_5_. **Mol. mass**: 606.428425. **Yield**: 559 mg (92.2%). **M.p.**: 165–167 °C (EtOH). **R_f_**: Table 1. **^13^C NMR** (δ): 177.82 (C_q_, C-28), 158.70 (C_q_, Fur-**CO**O-TT), 146.14 (CH, **Fur**-COO-TT), 145.19 (CH, **Fur**-COO-TT), 143.85 (C_q_, C-13), 122.25 (CH, C-12), 117.35 (CH, **Fur**-COO-TT), 111.68 (CH, **Fur**-COO-TT), 81.64 (CH, C-3), 63.99 (CH_2_, -COO-**CH_2_**-CH_2_-CH_2_-CH_3_), 33.13 (CH_2_, -COO-CH_2_-**CH_2_**-CH_2_-CH_3_), 19.27 (CH_2_, -COO-CH_2_-CH_2_-**CH_2_**-CH_3_), 13.75 (CH_3_, -COO-CH_2_-CH_2_-CH_2_-**CH_3_**). **^1^H NMR** (δ): 7.59 (1H, dd, *J* = 1.8 and 0.9 Hz, **Fur**-COO-TT), 7.15 (1H, dd, *J* = 3.5 and 0.9 Hz, **Fur**-COO-TT), 6.51 (1H, dd, *J* = 3.4 and 1.8 Hz, **Fur**-COO-TT), 5.31 (1H, t, *J* = 3.5 Hz, C_12_-H), 4.74 (1H, dd, *J* = 9.1 and 7.3 Hz, C_3_-H_α_), 4.04 (2H, td, *J* = 6.6 and 1.1 Hz, -COO-**CH_2_**-CH_2_-CH_2_-CH_3_), 2.90 (1H, dd, *J* = 13.9, 4.5 Hz, C_18_-H_β_), 1.55–1.51 (2H, m, -COO-CH_2_-**CH_2_**-CH_2_-CH_3_), 1.36–1.28 (2H, m, -COO-CH_2_-CH_2_-**CH_2_**-CH_3_), 1.16, 0.98 × 2, 0.95, 0.94 × 2, 0.92, 0.77 (4 × 3H + 2 × 6H, 6 × s, 7 × CH_3_ group of oleanane skeleton and -COO-CH_2_-CH_2_-CH_2_-**CH_3_**). **MS**: 606.6 (16.3%, M^•+^).

**Isobutyl ester of 3-O-furoyloleanolic acid (isobutyl 3-O-furoyl oleanolate, compound 3h)**: the ester **3h** was prepared using 512 mg (1.0 mmol) of *iso*-butyl oleanolate (**2h**). **Mol. formula**: C_39_H_58_O_5_. **Mol. mass**: 606.428425. **Yield**: 579 mg (95.5%). **M.p.**: 183–185 °C (EtOH). **R_f_**: Table 1. **^13^C NMR** (δ): ^13^C NMR (101 MHz, CDCl3) δ: 177.09 (C_q_, C-28), 158.70 (C_q_, Fur-**CO**O-TT), 146.14 (CH, **Fur**-COO-TT), 145.19 (CH, **Fur**-COO-TT), 143.83 (C_q_, C-13), 122.16 (CH, C-12), 117.35 (CH, **Fur**-COO-TT), 111.68 (CH, **Fur**-COO-TT), 81.64 (CH, C-3), 70.39 (CH_2_, -COO-**CH_2_**-CH-[CH_3_]_2_), 46.37 (C_q_, C-17), 28.14 (CH_2_, -COO-CH_2_-**CH**-[CH_3_]_2_), 19.44 and 19.30 (CH_3_, -COO-CH_2_-CH-**[CH_3_]**_2_). **^1^H NMR** (δ): 7.59 (1H, dd, *J* = 1.8 and 0.9 Hz, **Fur**-COO-TT), 7.14 (1H, dd, *J* = 3.5 and 0.9 Hz, **Fur**-COO-TT), 6.51 (1H, dd, *J* = 3.4 and 1.8 Hz, **Fur**-COO-TT), 5.31 (1H, t, *J* = 3.6 Hz, C_12_-H), 3.79 (2H, dd, *J* = 6.4, 3.5 Hz, -COO-**CH_2_**-CH-[CH_3_]_2_), 4.74 (1H, dd, *J* = 9.1 and 7.3 Hz, C_3_-H_α_), 2.88 (1H, dd, *J* = 13.6, 4.3 Hz, C_18_-H_β_), 1.96 (1H, dd, *J* = 5.3 and 2.7 Hz, -COO-CH_2_-**CH**-[CH_3_]_2_); 1.22 (6H, dd, *J* = 11.5 and 6.3 Hz, -COO-CH_2_-CH-**[CH_3_]_2_**), 1.16. 0.98, 0.97 × 2, 0.94, 0.91, 0.79 (5 × 3H + 1 × 6H, 6 × s, 7 × CH_3_ group of oleanane skeleton). **MS**: 606.5 (11.1%, M^•+^).

***sec*-Butyl ester of 3-O-furoyloleanolic acid (*sec*-butyl 3-O-furoyl oleanolate, compound 3i)**: the ester **3i** was prepared using 512 mg (1.0 mmol) of *sec*-butyl oleanolate (**2i**). **Mol. formula**: C_39_H_58_O_5_. **Mol. mass**: 606.428425. **Yield**: 577 mg (95.2%). **M.p.**: 160–162 °C (EtOH). **R_f_**: Table 1. **^13^C NMR** δ: 177.16 (C_q_, C-28), 158.68 (C_q_, Fur-**CO**O-TT), 146.13 (CH, **Fur**-COO-TT), 145.20 (CH, **Fur**-COO-TT), 143.83 (C_q_, C-13), 122.27 (CH, C-12), 117.34 (CH, **Fur**-COO-TT), 111.67 (CH, **Fur**-COO-TT), 81.63 (CH, C-3), 71.58 (CH, -COO-**CH**(CH_3_)-CH_2_-CH_3_), 58.43 (weak, CH_3_-**CH_2_**-OH), 46.58 (C_q_, C-17), 28.84 (CH_2_, -COO-CH(CH_3_)-**CH_2_**-CH_3_), 19.43 (CH_3_, -COO-CH(**CH_3_**)-CH_2_-CH_3_), 18.44 (weak, **CH**_3_-CH_2_-OH), 9.68 (CH_3_, -COO-CH(CH_3_)-CH_2_-**CH_3_**). **^1^H NMR** δ: 7.58 (1H, dd, *J* = 1.8 and 0.9 Hz, **Fur**-COO-TT), 7.14 (1H, dd, *J* = 3.5 and 0.9 Hz, **Fur**-COO-TT), 6.50 (1H, dd, *J* = 3.5 and 1.8 Hz, **Fur**-COO-TT), 5.31 (1H, q, *J* = 3.6 Hz, C_12_-H), 4.82–4.70 (2H, m + dd; m: -COO-**CH**(CH_3_)-CH_2_-CH_3_ and dd: *J* = 9.1 and 7.3 Hz, C_3_-H_α_), 3.72 (traces, dd, *J* = 7.0 and 14.0 Hz, CH_3_-**CH_2_**-OH), 2.90 (1H, dt, *J* = 13.8 and 4.3 Hz, C_18_-H_β_), 1.94–1.87 (2H, m, -COO-CH(CH_3_)-**CH_2_**-CH_3_), 1.24 (weak, t, *J* = 7.0 Hz, **CH_3_**-CH_2_-OH), 1.18 (3H, t, *J* = 6.7 Hz, -COO-CH(**CH_3_**)-CH_2_-CH_3_), 1.16 × 2, 0.98, 0.97, 0.94 × 2, 0.91 (3 × 3H + 2 × 6H, 5 × s, 7 × CH_3_ group of oleanane skeleton), 0.79 (3H, d, *J* = 1.9 Hz, -COO-CH(CH_3_)-CH_2_-**CH_3_**). **MS**: 606.4 (22.1%, M^•+^).

**2,3-Dichloropropyl ester of 3-O-furoyloleanolic acid (2,3-dichloropropyl 3-O-furoyl oleanolate, compound 3j)**: the ester **3j** was prepared using 566 mg (1.0 mmol) of 2,3-dichloropropyl oleanolate (**2j**). **Mol. formula**: C_38_H_54_Cl_2_O_5_. **Mol. mass**: 660.334831. **Yield**: 614 mg (93.0%). **M.p.**: 90–93 °C precipt. with water from EtOH sol.). **R_f_**: Table 1. **^13^C NMR**: 176.78 (C_q_, C-28), 158.67 (C_q_, Fur-**CO**O-TT), 146.13 (CH, **Fur**-COO-TT), 145.17 (CH, **Fur**-COO-TT), 143.50 (C_q_, C-13), 136.25 (CH, -COO-CH_2_-**CH**(Cl)-CH_2_-Cl), 122.57 (CH, C-12), 117.34 (CH, **Fur**-COO-TT), 114.78 (CH_2_, -COO-CH_2_-CH(Cl)-CH_2_-Cl), 111.67 (CH, **Fur**-COO-TT), 81.60 (CH, C-3), 65.82 (CH_2_, -COO-**CH_2_**-CH(Cl)-CH_2_-Cl), 46.91 (C_q_, C-17). **^1^H NMR**: 7.58 (1H, dd, *J* = 1.8, 0.9 Hz, **Fur**-COO-TT), 7.14 (1H, dd, *J* = 3.4, 0.9 Hz, **Fur**-COO-TT), 6.49 (1H, dd, *J* = 3.5, 1.8 Hz, **Fur**-COO-TT), 5.48 (1H, q, *J* = 1.3 Hz, -COO-CH_2_-CH(Cl)-**CH_2_**-Cl) and 5.39 (1H, d, *J* = 1.8 Hz, -COO-CH_2_-CH(Cl)-**CH_2_**-Cl), 5.31 (1H, t, *J* = 3.7 Hz, C_12_-H), 4.72 (1H, dd, *J* = 9.1, 7.3 Hz, C_3_-H_α_), 4.62 (2H, dd, *J* = 13.8, 7.5 Hz, -COO-**CH_2_**-CH(Cl)-CH_2_-Cl), 2.89 (1H, dd, *J* = 13.8, 4.8 Hz, C_18_-H_β_), 2.01 (1H, td, *J* = 13.7, 41 Hz, -COO-CH_2_-**CH**(Cl)-CH_2_-Cl), 1.15, 0.96 × 2, 0.94, 0.93, 0.91, 0.74 (5 × 3H + 1 × 6H, 6 × s, 7 × CH_3_ group of oleanane skeleton). **MS**: 660.5 (9.8%, M^•+^).

**3,28-difuroylerythrodiol (compound 3k)**: the ester **3k** was prepared using 442 mg (1.0 mmol) of erythrodiol (**2k**). **Mol. formula**: C_40_H_54_O_6_. **Mol. mass**: 630.392040. **Yield**: 588 mg (93.3%). **M.p.**: 210–212 °C (EtOH). **R_f_**: Table 1.

**^13^C NMR** (δ): 158.92 (C_q_, Fur-**CO**O-TT-OCO-Fur), 158.67 (C_q_, Fur-COO-TT-O**CO**-Fur), 146.21 (CH, Fur-COO-TT-OCO-**Fur**), 146.13 (CH, **Fur**-COO-TT-OCO-Fur), 145.16 (CH, **Fur**-COO-TT-OCO-Fur), 144.89 (CH, Fur-COO-TT-OCO-**Fur**), 143.48 (C_q_, C-13), 122.95 (CH, C-12), 117.45 (CH, Fur-COO-TT-OCO-**Fur**), 117.35 (CH, **Fur**-COO-TT-OCO-Fur), 111.72 (CH, Fur-COO-TT-OCO-**Fur**), 111.68 (CH, **Fur**-COO-TT-OCO-Fur), 81.57 (CH, C-3), 71.01 (CH_2_, C-28), 38.25 (C_q_, C-17). **^1^H NMR** (δ): 7.59 (1H, dd, *J* = 1.8, 0.9 Hz) and 7.58 (1H, dd, *J* = 1.5, 0.6 Hz, **Fur**-COO-TT-OCO-**Fur**), 7.15 (dd, *J* = 3.5, 0.9 Hz) and 7.14 (1H, dd, *J* = 3.4, 0.9 Hz, **Fur**-COO-TT-OCO-**Fur**), 6.51 (1H, dd, *J* = 3.5, 1.8 Hz) and 6.50 (1H, dd, *J* = 3.5, 1.7 Hz, **Fur**-COO-TT-OCO-**Fur**), 5.24 (1H, t, *J* = 3.8 Hz, C_12_-H), 4.73 (1H, dd, *J* = 9.2, 7.2 Hz, C_3_-H_α_), 4.27 (1H, d, *J* = 10.9 Hz, C_28_-H_a_) and 3.93 (1H, d, *J* = 11.0 Hz, C_28_-H_b_), 2.14 (1H, dd, *J* = 8.5, 5.4 Hz, C_18_-H_β_), 1.19, 1.00, 0.99, 0.97, 0.93, 0.91 × 2 (5 × 3H + 1 × 6H, 6 × s, 7 × CH_3_ group of oleanane skeleton). **MS**: 630.4 (10.0%, M^•+^).

#### 2.1.4. Polarity

The polarity of oleanolic acid derivatives was evaluated relative to the parent compound (OA, **1**) using a HPTLC method based on the R_f_ value.

HPTLC was performed using aluminum-backed plates precoated with silica gel 60 F_254_ (Merck, Germany; #1.05554). The investigated compounds (**1**, **2a**–**2k** and **3a**–**3k**) were prepared as solutions in CH_2_Cl_2_ (0.5%, *w*/*v*), and 2 μL of each solution was applied to the starting line. Chromatographic development was performed on 10 × 10 cm plates at 22 ± 1 °C in a horizontal developing chamber presaturated with mobile-phase vapors for 10 min. The mobile phase consisted of benzene and ethyl acetate mixed in various volume ratios (1:1, 2:1, 4:1, 9:1, 15:1, and 25:1) or with pure ethyl acetate and pure benzene. After development, the plates were air-dried and derivatized by spraying with a 20% (*v*/*v*) sulfuric acid solution in ethanol, followed by heating at 105–110 °C for 5 min. The resulting colored spots were visualized against a white background. All chromatographic analyses were performed in triplicate, and the mean retention factor (R_f_) values were calculated.

### 2.2. Structure–Activity Prediction

The structure–activity relationship assessment was conducted using the Prediction of Activity Spectra for Substances (PASS) computer system [55]. PASS enables the prediction of a wide range of pharmacological activities and mechanisms of action based solely on the chemical structure of a compound, employing multilevel neighborhoods of atoms (MNA) descriptors for this purpose. The computational analysis yields a list of potential activities, each accompanied by two parameters: P_a_, representing the probability that a given activity is present, and P**_i_**, indicating the probability that the activity is absent. Both probabilities range from 0 to 1.

### 2.3. ADMETox Profile Prediction

**Figure 1:**
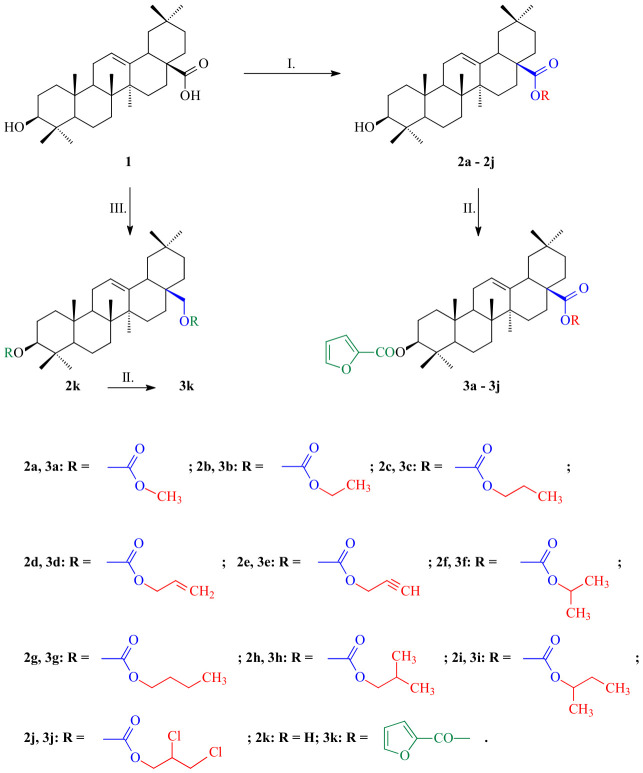
Synthesis of anticancer and antioxidant agents **2a**–**2k** and **3a**–**3k**. **Legend**: **I**: DMF. 60 °C, K_2_CO_3_, alkyl halide; **II**: dioxane, r.t., furoic acid, DMAP, DCC; **III**: THF, r.t., LiAlH_4_.

The physicochemical characteristics, pharmacokinetic profiles, and ADMETox properties of compounds **1**, **2a**–**2k**, **3a**–**3k** (Figure 1) were predicted using the comprehensive ADMETlab 2.0 database [56]. Initially, the chemical structures of all compounds under investigation were constructed and optimized using the web-based JavaScript Molecule Editor (JSME), ensuring accurate representation for subsequent computational analyses. These predicted parameters provided an integrated assessment of the potential drug-likeness and safety profiles of the compounds prior to experimental evaluation.

### 2.4. Anticancer Activity

The anticancer activity of the obtained oleanolic acid derivatives was evaluated using the 3-(4,5-dimethylthiazol-2-yl)-2,5-diphenyltetrazolium bromide (MTT) assay, according to previously established protocols, e.g., ref. [48]. All cytotoxicity experiments were performed in 3 independent biological experiments, each including three technical replicates per concentration. Data are presented as mean half-maximal inhibitory concentration (IC_50_) values with standard deviation (SD). The Selectivity Index (SI) was calculated as the ratio of the IC_50_ value obtained for non-malignant human dermal fibroblast (HDF) cells to that obtained for cancer cell lines. Unless otherwise specified, the study was designed as a descriptive screening analysis, and no formal post hoc comparisons were performed. Statistical analyses for the cytotoxic activity were carried out using the non-parametric Kruskal–Wallis H test for unpaired data and Friedman two-way analysis of variance test for paired data. Statistical significance was tested using Dunn’s post hoc test. The IC_50_ values compared to the control IC_50_ were calculated using GraphPad Prism v. 10 software.

The MTT assay is a colorimetric method that relies on the ability of mitochondrial dehydrogenases present in viable cells to reduce the yellow tetrazolium salt (known as MTT) to insoluble violet formazan crystals. The extent of formazan formation directly reflects the metabolic activity of the cells and, consequently, their viability. After incubation with MTT, the resulting crystals are solubilized, and the absorbance is measured spectrophotometrically, providing a quantitative estimate of the number of living cells. Detailed experimental conditions and procedures are described in our previous paper [48].

### 2.5. Antioxidant Activity

The DPPH assay is based on the capacity of antioxidant compounds to neutralize the stable DPPH radical through hydrogen atom or electron donation. This interaction leads to a reduction of the violet DPPH radical and a corresponding decrease in absorbance, which reflects the radical-scavenging potential of the tested sample. In contrast, the CUPRAC assay evaluates the reducing capacity of a compound by measuring its efficiency in reducing the Cu(II) form, neocuproine complex, to the Cu(I) form; the resulting chromophore allows for quantitative spectrophotometric analysis.

For both assays, the antioxidant activity is expressed in two complementary ways: as the percentage inhibition of the respective oxidizing agents and as Trolox equivalents (TEs), calculated from calibration curves. Full experimental procedures and methodological details are provided in our previous paper [57]. The antioxidant assays were performed in three independent experiments, each including three technical replicates. Results are expressed as mean values with SD. The DPPH and CUPRAC assays were used as cell-free chemical assays to describe radical-scavenging and reducing capacity under simplified experimental conditions. These assays were not intended to directly measure intracellular antioxidant or pro-oxidant activity.

### 2.6. Molecular Docking

#### 2.6.1. Ligand Preparation

Two-dimensional (2D) structures of the investigated compounds were drawn using ChemDraw 23.0.1 and subsequently converted into three-dimensional (3D) models with OpenBabel [58], generating low-energy conformations and corresponding coordinates. Geometry optimization was performed in Avogadro 1.91.0 using the universal force field (UFF) and the steepest descent algorithm. The optimized ligands were saved as structure data files (SDFs) and used as input for docking calculations on the CB-Dock2 [59] platform.

#### 2.6.2. Protein Preparation

The crystallographic structure of the EGFR tyrosine kinase domain was retrieved from the Research Collaboratory for Structural Bioinformatics Protein Data Bank (RCSB PDB; PDB ID: 1M17, resolution 2.60 Å) [60]. In this X-ray structure, EGFR is co-crystallized with the inhibitor erlotinib, providing an experimentally validated reference binding site. The 1M17.pdb file was uploaded to CB-Dock2, and protein preprocessing steps (e.g., removal of co-crystallized ligands and crystal waters, addition of hydrogens, and standardization of the input structure) were carried out automatically by the server without manual editing.

#### 2.6.3. Protocol Validation (Redocking)

To validate the docking workflow, the co-crystallized ligand (erlotinib) was redocked into the EGFR model. The resulting pose was aligned with the crystallographic ligand conformation in PyMOL 3.1 (Python Molecular Graphics, Schrödinger, LLC, New York, NY, USA)), and root mean square deviation (RMSD) was calculated in PyMOL 3.1 based on the superposition (ligand heavy atoms). The obtained RMSD values below 2 Å confirmed that the adopted docking protocol reliably reproduces the experimental binding mode.

#### 2.6.4. Detecting Cavities and Uploading Ligands

After uploading EGFR (1M17) to CB-Dock2, potential binding sites were identified using the Search Cavities function, with the number of detected cavities set to 5 (“more parameters”). The ligands were then uploaded and docking was performed using Auto Blind Docking with the same cavity settings. For comparative analysis, docking results were primarily discussed for the largest identified cavity, which corresponds to the co-crystallized ligand binding site in 1M17 (the inhibitor/ATP-binding pocket). This approach ensures consistent compound comparison within a biologically relevant binding region.

## 3. Results

### 3.1. Preparation of Oleanolic Acid Derivatives **2a**–**2k** and **3a**–**3k**

#### 3.1.1. Synthesis 

The sequence of chemical modifications leading to the two series of oleanolic acid derivatives is presented in Figure 1. The synthetic approach was based on a previously reported method developed by Bednarczyk-Cwynar and described in an earlier publication [47]. First, oleanolic acid (**1**) was subjected to alkylation with alkyl halides in dimethylformamide (DMF) in the presence of potassium carbonate (K_2_CO_3_). This transformation represents a classical carboxyl group alkylation. In this process, the poorly reactive carboxylic acid is converted into the more reactive potassium carboxylate, which subsequently undergoes nucleophilic substitution with an alkyl halide to afford the desired ester. Oleanolic acid (**1**) was also reduced to erythrodiol (**2k**, Figure 1) using lithium aluminum hydride (LiAlH_4_) in tetrahydrofuran (THF).

The obtained esters of oleanolic acid (**2a**–**2j**, Figure 1) and erythrodiol (**2k**, Figure 1) were then subjected to acylation with 2-furoic acid in dioxane, using DMAP and DCC as activating agents, giving the corresponding furoyl derivatives (**3a**–**3k**, Figure 1).

#### 3.1.2. Spectral Data Analysis 

The key diagnostic signals derived from the ^13^C NMR and ^1^H NMR spectra for all synthesized oleanolic acid derivatives (**2a**–**2k** and **3a**–**3k**, Figure 1) are summarized in the Methods and Materials section. The NMR spectra are provided in Supplementary Materials, Figures S1 and S2.

#### 3.1.3. Polarity Comparison 

The chromatographic behavior of oleanolic acid (**1**) and its derivatives (**2a**–**2k** and **3a**–**3k**; Figure 1) was systematically evaluated by analyzing the dependence of their R_f_ values on the composition of the mobile phase. HPTLC was performed on silica gel plates using solvent systems of decreasing polarity, consisting of benzene (C_6_H_6_) and ethyl acetate (AcOEt) mixed in various volume ratios as well as in pure AcOEt, C_6_H_6_ and CH_2_Cl_2_. The results are given in Table 1.

### 3.2. SAR Analysis

The highest SAR prediction results obtained using the PASS Inet platform for oleanolic acid (**1**), its esters **2a**–**2j**, erythrodiol **2k** and furoyl derivatives of these triterpenes (compounds **3a**–**3j**; Figure 1) are presented in Table 2 and Table 3. The detailed results of the SAR analysis with P_a_ ≥ 0.700 are provided in Tables S1–S3. These tables offer a comprehensive overview of the anticipated biological activities for the analyzed derivatives, expressed as P_a_ and P_i_ probability values. The compiled data enable a comparative assessment of how specific structural modifications within the oleanolic acid scaffold influence the likelihood of particular pharmacological effects. This comprehensive presentation serves as a basis for identifying key functional groups and substituent patterns associated with enhanced or diminished predicted activity, thereby supporting further rational optimization of the studied compounds.

### 3.3. Prediction of ADMETox Parameters

Selected key ADMETox parameters predicted using the ADMETlab platform for oleanolic acid (**1**), its ester derivatives (**2a**–**2j**), erythrodiol (**2k**), and the corresponding furoyl derivatives (**3a**–**3k**) are summarized in Table 4 and Table 5. These parameters were selected to provide a preliminary computational overview of physicochemical properties, predicted pharmacokinetic behavior and potential toxicity-related liabilities. Because these results were obtained exclusively in silico, they should be interpreted as predictive estimates and not as experimentally verified pharmacokinetic or toxicological data. A comprehensive set of ADMETox prediction results, including physicochemical descriptors and extended absorption, distribution, metabolism, excretion and toxicity endpoints, is provided in Tables S3 and S4.

### 3.4. Anticancer Activity

The cytotoxic properties of oleanolic acid derivatives (**2a**–**2k** and **3a**–**3k**; Figure 1) were assessed in vitro against a panel of human cancer cell lines, including HeLa (human cervical carcinoma), MCF-7 (human breast carcinoma), A-549 (human lung adenocarcinoma), SKBR-3 (human breast adenocarcinoma), PC-3 (human prostate carcinoma) and SKOV-3 (ovarian cystadenocarcinoma). In addition, their effects on a non-malignant human cell line, HDF, were evaluated to estimate potential selectivity toward malignant cells. The HDF cell line was used as non-malignant reference model. However, this approach provides only a preliminary estimate of cytotoxicity and should not be considered a complete toxicological assessment. Cytotoxicity was determined using the MTT assay, in which the amount of produced formazan correlates directly with the number of viable cells, enabling quantitative assessment of growth inhibition.

The calculated IC_50_ values for oleanolic acid (**1**, Figure 1) and its derivatives (**2a**–**2k** and **3a**–**3k**; Figure 1) are summarized in Table 6, providing a comparative overview of their cytotoxic potential across the tested malignant and non-malignant cell lines.

### 3.5. Selectivity Index

The Selectivity Index (SI) values, calculated as a ratio of IC_50_ for the non-malignant cell line (HDF) and IC_50_ for the respective cancerous cell line, are presented in Table 7.

### 3.6. Antioxidant Activity

**Figure 2:**
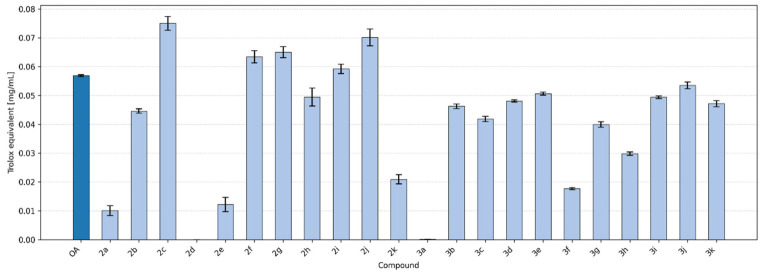
Antioxidant activity in the CUPRAC assay of OA and its derivatives **2a**–**2k** and **3a**–**3k**, expressed as Trolox equivalents. Data are presented as mean values with SD from 3 independent experiments, each performed with 3 technical replicates.

**Figure 3:**
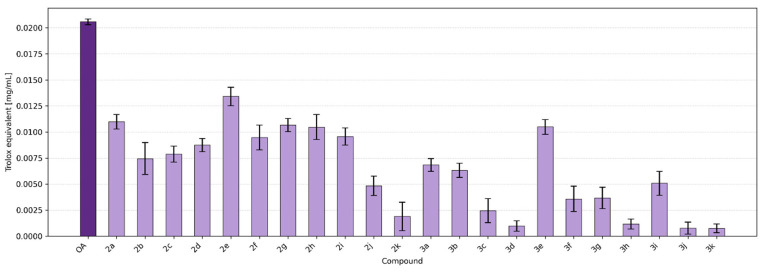
Antioxidant activity in the DPPH assay of OA and its derivatives **2a**–**2k** and **3a**–**3k**, expressed as Trolox equivalent. Data are presented as mean values with SD from 3 independent experiments, each performed with 3 technical replicates.

The antioxidant potential of oleanolic acid (OA) and its newly synthesized triterpene derivatives (**2a**–**2k**, **3a**–**3k**, Figure 1) was evaluated using two complementary in vitro assays: the CUPRAC method, which measures the ability to reduce Cu(II) ions via single electron transfer (SET), and the DPPH assay, based on free-radical-scavenging capacity primarily through the hydrogen atom transfer (HAT) mechanism. The results are presented in Figure 2 and Figure 3. Blue bars represent CUPRAC results, whereas pink bars correspond to DPPH assay outcomes. In both assays, the activity was expressed as a percentage of inhibition (of Cu(II) ions and DPPH radicals, respectively) and as Trolox equivalents, which were calculated using the standard curves provided in Figure S3.

### 3.7. Molecular Docking

#### 3.7.1. Cavity Detection and Selection of the Docking Site (C1)

**Figure 4:**
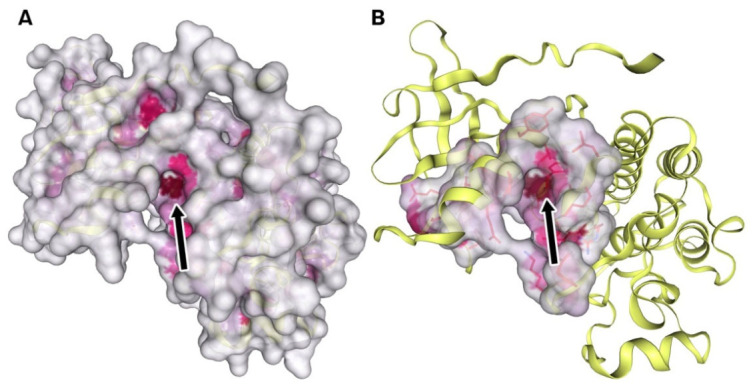
(**A**). Whole 1M17 molecule, the dark pink color indicates the cavity of the molecule. (**B**). View of the largest pocket of C1 with a volume of 991 Å^3^ indicated by a black arrow.

Binding-site detection using the CurPocket method implemented in CB-Dock2 identified five potential cavities in the EGFR structure (PDB: 1M17). The largest cavity (C1, Table 8) exhibited a volume of 991 Å^3^ with the geometric center at (36, 8, 51) and was annotated as a typical EGFR active-site pocket. Due to its size and correspondence to the inhibitor/ATP-binding region in the 1M17 reference complex (Figure 4), subsequent analysis was restricted to C1. The docking protocol was validated by redocking the co-crystallized ligand (erlotinib). The reproduced pose matched the crystallographic conformation with root mean square deviation (RMSD) < 2 Å, calculated in PyMOL after structure superposition, supporting the suitability of the adopted settings for pose reproduction in this system.

#### 3.7.2. Docking Outcomes in the C1 Pocket

Docking was performed for oleanolic acid derivatives (**2c**, **2i**, **2j**, Figure 1) and 3-(2-furoyl) derivatives (**3c**, **3f**, **3h**, **3i**, **3j**, Figure 1) which presented the highest anticancer activity (Table 6). All compounds yielded favorable predicted binding energies in C1, with Vina scores ranging from −7.2 to −8.5 kcal × mol^−1^ (Table 9), consistently within the same large pocket volume (991 Å^3^). The top-ranked ligands were: *sec*-butyl oleanolate **2i** = −8.5 kcal × mol^−1^, *n*-propyl 3-O-furoyl oleanolate **3c** = −8.3 kcal × mol^−1^. The next best result was: isopropyl 3-O-furoyl oleanolate **3f** = −7.8 kcal × mol^−1^. The remaining ligands showed slightly weaker but still favorable scores: *n*-propyl oleanolate **2c** = −7.5 kcal × mol^−1^, 2,3-dichloropropyl oleanolate **2j** = −7.5kcal × mol^−1^, *sec*-butyl 3-O-furoyl oleanolate **3i** = −7.4 kcal × mol^−1^, and isobutyl 3-O-furoyl oleanolate **3h** = −7.3, **3j** = −7.2 kcal × mol^−1^.

#### 3.7.3. Molecular Docking Results—Contact Profile in C1

The contact-residue lists indicate that ligand accommodation in C1 is dominated by extensive hydrophobic complementarity within the inhibitor/ATP-pocket environment, with recurrent involvement of residues such as PHE699 (phenylalanine at position 699 within a protein’s 3D structural model), LYS721 (lysine at position 721), LEU723 (leucine at position 723), GLU734, (glutamic acid at position 734)/GLU738 (glutamic acid at position 738), ASP813 (aspartic acid at position 813), ARG817 (arginine at position 817), ASN818 (asparagine at position 818), ASP831 (aspartic acid at position 831), GLY833 (glycine at position 833), LEU834 (leucine at position 834), LYS851 (lysine at position 851), VAL852 (valine at position 852), and PRO853 (proline at position 853 within a protein’s 3D structural model). The two best-scoring compounds (**2i** and **3c**) displayed the broadest contact patterns within this region, consistent with their lowest Vina scores.

For clarity in the main manuscript, interaction visualizations are presented for the two top-scoring ligands (**2i** and **3c**), whereas the complete dataset (tables and additional interaction maps) is provided in the Figures S4–S12 and Table S5.

#### 3.7.4. Molecular Docking Results (C1 Pocket): Compounds **2i** and **3c**

Within docking analyses restricted to the largest C1 cavity (volume 991 Å^3^; center 36, 8, 51), the two most favorable results in the investigated subset “2” series (**2c**, **2i**, **2j**, Figure 1) and “3” series (**3c**, **3f**, **3h**, **3i**, **3j**, Figure 1) derivatives were obtained for *sec*-butyl oleanolate **2i** (Vina score = −8.5 kcal × mol^−1^) and *n*-propyl 3-O-furoyl oleanolate **3c** (Vina score = −8.3 kcal × mol^−1^). Details are shown in Table 9.

**Figure 5:**
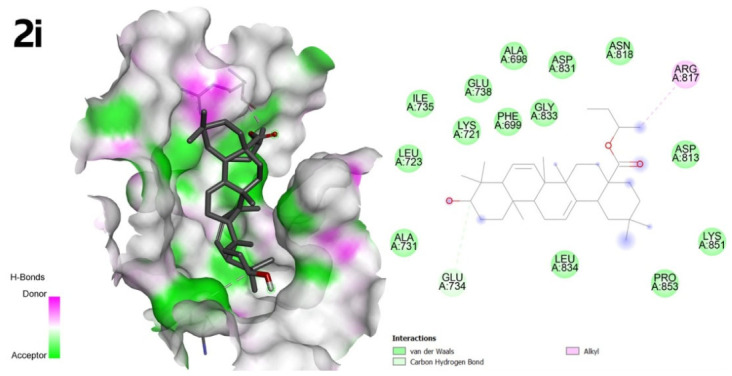
3D surface of structure 1M17 with ligand compound **2i** with H-bond gradient map in C1 pocket and 2D diagram with interactions. Predicted docking pose of derivative **2i** is computationally predicted and does not confirm experimental EGFR inhibition.

Compound **2i** (*sec*-butyl oleanolate) showed the strongest predicted binding in the C1 pocket among the structures discussed here (−8.5 kcal × mol^−1^). The 2D interaction map indicates that the complex is dominated by extensive hydrophobic/van der Waals contacts between the triterpenoid scaffold and residues lining the cavity (e.g., PHE699, LEU723, ILE735, LEU834, VAL852, PRO853), which is consistent with bulky, lipophilic ligands occupying a large binding pocket. The 3D surface of structure 1M17 with ligand compound **2i** with the H-bond gradient map in the C1 pocket and 2D diagram with interactions are presented in Figure 5.

Notably, two directional interactions stand out: (i) a carbon hydrogen bond (C–H···O) between a ligand oxygen atom (ester/carboxylate region) and GLU734, and (ii) an alkyl contact involving ARG817, suggesting favorable placement of the sec-butyl/hydrophobic surface relative to this residue. Overall, the docking pose of **2i** appears to be stabilized primarily by hydrophobic complementarity, with only a limited contribution from polar contacts and without a strong network of conventional hydrogen bonds.

**Figure 6:**
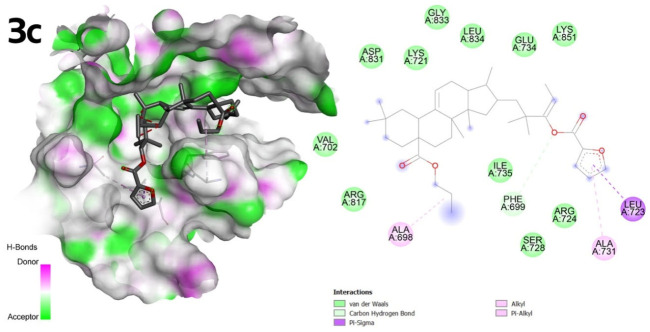
3D surface of structure 1M17 with ligand compound **3c** with H-bond gradient map in C1 pocket and 2D diagram with interactions. Predicted docking pose of derivative **3c** is computationally predicted and does not confirm experimental EGFR inhibition.

Compound **3c** (*n*-propyl 3-O-furoyl oleanolate) produced the second-best C1 score (−8.3 kcal × mol^−1^). Compared with derivative **2i**, the discussed ester **3c** contains an additional furoyl moiety that introduces an aromatic/heteroatom element, reflected by the interaction profile. The 2D map (Figure 6) shows π-type interactions within the hydrophobic region of C1, including a π-sigma contact between the furan ring and LEU723 and a π-alkyl interaction with ALA731, which together support stable positioning of the aromatic fragment in the pocket. In addition, ester oxygen atoms contribute to weak polar stabilization, including a carbon hydrogen bond with PHE699 (C–H···O). The pose is further reinforced by multiple van der Waals contacts with neighboring residues, e.g., ALA698 (alanine at position 698 within a protein’s 3D structural model), GLY700 (glycine at position 700), VAL702 (valine at position 702), LYS721, GLU734, ILE735 (isoleucine at position 737), ASP813, ASN818, ASP831, LEU834, LYS851.

These findings suggest that the favorable score of **3c** arises from a synergy between the hydrophobic fit of the triterpenoid core and additional π-driven stabilization provided by the furan fragment, which may improve pose retention within the C1-binding site.

## 4. Discussion

### 4.1. Synthesis and Structure Confirmation

In all NMR spectra recorded for the obtained oleanolic acid derivatives (**2a**–**2j** and **3a**–**3j**, Figure 1), the presence of signals characteristic of the expected molecular structures was confirmed. For instance, in the ^13^C NMR spectra, the signal corresponding to the C-28 atom consistently appeared at approximately δ 177–178 ppm, which is typical for the substituted carboxyl carbon of oleanane-type triterpenes. For erythrodiol (**2k**, Figure 1), the signal derived from the secondary carbon at position C-28 was observed at δ 71.28 ppm, whereas in its furoyl derivative (**3k**, Figure 1) the analogous carbon signal appeared at 71.01 ppm. Additionally, the characteristic chemical shifts of atoms C-13, C-12, and C-3 in all synthesized derivatives (**2a**–**2k** and **3a**–**3k**, Figure 1) were detected within the following chemical shift ranges: δ 143–146 ppm (C-13), δ 122–124 ppm (C-12), and δ approximately 78 ppm (C-3).

In the ^1^H NMR spectra, the proton at the C-12 position of oleanolic acid derivatives (**2a**–**2j** and **3a**–**3j**, Figure 1) typically appeared as a triplet at δ 5.2–5.3 ppm, consistent with its vinylic environment. The C-3 proton, present in all oleanolic acid derivatives (**2a**–**2k** and **3a**–**3k**, Figure 1), formed a characteristic doublet of doublets, usually observed at around 3.2 ppm. Meanwhile, the proton at the C-18 position also appeared as a doublet of doublets and was observed at δ approximately 2.9 ppm for derivatives **2a**–**2j** and **3a**–**3j** (Figure 1) or at δ approximately 2.0 ppm in the case of **2k** and **3k** (Figure 1).

For all furoyl derivatives of oleanolic acid (**3a**–**3j**, Figure 1) and erythrodiol (**3k**, Figure 1), the ^13^C NMR spectra exhibited a distinct signal of the carboxyl carbon within the furoyl moiety at δ approximately 159 ppm. The four carbon atoms of the furan ring gave characteristic resonances, typically observed at δ 146, 145, 117, and 112 ppm, confirming the presence of the heteroaromatic ring system.

The corresponding ^1^H NMR spectra of all furoyl derivatives revealed three well-resolved doublets of doublets, generally located at δ approximately 7.6, 7.1, and 6.5 ppm, consistent with the proton pattern of a substituted furan ring.

The structural identity of all synthesized derivatives (**2a**–**2k** and **3a**–**3k**, Figure 1) was further confirmed by mass spectrometry, which revealed molecular ion peak corresponding to the calculated molecular masses.

### 4.2. Polarity

The obtained unsubstituted derivatives of oleanolic acid (**2a**–**2k**; Figure 1) are, in general, less polar than the parent oleanolic acid (**1**; Figure 1). This difference in polarity is clearly associated with the esterification of the carboxyl group at the C-28 position. A carboxylic acid group (–COOH) is polar and capable of hydrogen bonding both as donor and acceptor, which increases molecular polarity and often improves interactions with polar solvents such as water. In contrast, an ester group (–COO–R) still has a carbonyl, but the hydrogen-bond donor ability is lost and only hydrogen-bond acceptance remains. Consequently, esters generally exhibit lower polarity and reduced hydrogen-bonding capacity compared with their parent carboxylic acids.

A clear and consistent trend was observed for all unsubstituted oleanolic acid derivatives (compounds **2a**–**2k**; Figure 1): R_f_ values decreased progressively with an increasing proportion of benzene in the mobile phase (Table 1). In solvent systems rich in ethyl acetate (e.g., AcOEt or C_6_H_6_:AcOEt = 1:1), most compounds exhibited high R_f_ values (generally ≥0.80, Table 1), indicating weak interactions with the polar stationary phase and enhanced elution strength of the mobile phase. This behavior reflects the relatively higher polarity of ethyl acetate, which effectively competes with analyte–silica interactions.

As the proportion of benzene increased (C_6_H_6_:AcOEt = 4:1 to 25:1), a gradual reduction in R_f_ values was noted. This trend indicates stronger retention of the compounds on the silica gel surface due to reduced mobile phase polarity and weaker solvation of polar functional groups present in the analyzed molecules. The effect was particularly pronounced in highly non-polar system (C_6_H_6_:AcOEt = 25:1) and benzene, where R_f_ values dropped sharply, in some cases almost approaching zero (Table 1).

Despite the structural similarity of compounds **2a**–**2k**, minor but reproducible differences in R_f_ values were observed across the solvent systems. These differences can be attributed to subtle variations in molecular polarity, functional group composition, and steric effects, which influence the strength of intermolecular interactions with the stationary phase.

The chromatographic behavior of compounds **3a**–**3k** provides insight into structure–polarity relationships within this series of oleanolic acid derivatives. In highly polar mobile phases (ethyl acetate or its mixture with small amount of benzene), all compounds exhibited very high R_f_ values (exceeding 0.90, Table 1), indicating weak interactions with the silica gel stationary phase and minimal structural discrimination under these conditions. This is consistent with the strong elution power of ethyl-acetate-rich solvent systems. As the polarity of the mobile phase decreased with increasing benzene content, clear differences in R_f_ values emerged, reflecting variations in lipophilicity and steric effects among the derivatives. Compounds with more hydrophobic or bulkier substituents, such as ester **3d** (allyl oleanolate; Figure 1) or **3j** (2,3-dichloropropyl 3-O-furoyl oleanolate) generally maintained higher R_f_ values in non-polar systems, indicating reduced adsorption to silica gel. In contrast, derivatives with lower R_f_ values, such as ester **3b** (ethyl 3-O-furoyl oleanolate; Figure 1) or **3c** (*n*-propyl oleanolate) exhibited stronger interactions with the stationary phase, suggesting relatively higher polarity or greater accessibility of polar functional groups.

When comparing the unsubstituted oleanolic acid derivatives from series “2” (i.e., compounds **2a**–**2k**, Figure 1) with the corresponding furoyl derivatives from series “3” (compounds **3a**–**3k**, Figure 1), it is evident that the furoyl derivatives are distinctly less polar than both the parent oleanolic acid and its unsubstituted derivatives from series “2”. This behavior can be attributed to the presence of the furoyl moiety at the C-3 position of the oleanane skeleton.

It is well established that substitution of the hydrogen atom of the C-3 hydroxyl group in the oleanane framework with a non-polar or moderately polar substituent results in a decrease in overall molecular polarity [62]. In thin-layer chromatography, this reduction in polarity is reflected by increased R_f_ values, consistent with the chromatographic behavior observed for the furoyl derivatives analyzed in this study.

When comparing the polarity of oleanolic acid esters bearing a three-carbon chain in the ester moiety—namely compounds **2c** and **3c** with a saturated chain, **2d** and **3d** containing a double bond, and **2e** and **3e** containing a triple bond (Figure 1)—only minor differences in polarity were observed (Table 1). These limited variations can be attributed to the dominant contribution of the non-polar triterpenoid carbon skeleton, which largely governs the overall physicochemical properties of the molecules.

Among compounds **2c**, **2d**, and **2e** (Figure 1), the most polar derivative, as indicated by the lowest R_f_ value, was compound **2c** bearing a triple bond in the ester side chain (Table 1). In contrast, for the corresponding furoyl analogues **3c**, **3d**, and **3e** (Figure 1), the polarity of these three derivatives was found to be very similar, with no pronounced differences in their chromatographic behavior.

When comparing the polarity of oleanolic acid derivatives bearing unbranched ester chains (**2c**, **2g**, **3c**, and **3g**; Figure 1) with their isomeric counterparts containing branched ester chains (derivatives **2f**, **2h**, and **2i** as well as **3f**, **3h**, and **3i**), only minor differences in polarity were observed (Table 1). As in the case of the presence or absence of multiple bonds within the ester moiety, these small variations can be attributed to the relatively minor contribution of the branched substituent compared with the dominant influence of the large, non-polar triterpenoid skeleton.

The observed trends demonstrate that ester substitution patterns systematically influence chromatographic mobility, with even minor structural variations affecting compound behavior. Overall, the TLC data confirm structure-dependent polarity differences within the series and provide indirect evidence of physicochemical trends that may be relevant to properties such as solubility, membrane permeability, and biological activity.

### 4.3. Analysis of Structure–Activity Relationship (SAR) Prediction Results

The predicted pharmacological activity profiles obtained for oleanolic acid (**1**, Figure 1), its ester derivatives (**2a**–**2k**, Figure 1), erythrodiol (**2k**, Figure 1), and the corresponding furoyl-modified derivatives (**3a**–**3k**, Figure 1) provide a detailed insight into how systematic structural modifications influence both the breadth and specificity of biological activity (Tables S1 and S2). Overall, the data clearly demonstrate that the oleanane triterpenoid scaffold inherently supports multitarget pharmacological potential, while functionalization at the C-17 and the C-3 positions modulates activity intensity, selectivity, and mechanistic orientation.

#### 4.3.1. Multitarget Profile of Oleanolic Acid

The parent compound, oleanolic acid (**1**, Figure 1), displays high predicted probabilities for a wide range of activities, including anti-inflammatory, antineoplastic, hepatoprotective, antioxidant-related (oxidoreductase inhibition), apoptosis-promoting, chemopreventive, and transcription-factor-modulating effects (Tables S1 and S2). Particularly high P_a_ values for caspase-3 and caspase-8 stimulation, apoptosis agonism, membrane integrity antagonism, and transcription factor stimulation highlight the strong association of OA with redox-sensitive signaling pathways and programmed cell death mechanisms.

This broad baseline activity supports the classification of OA as a privileged natural scaffold and provides a rational foundation for chemical derivatization aimed at enhancing specific biological endpoints or improving drug-like properties.

#### 4.3.2. Consequences of the C-17 Carboxyl Esterification: Series “2” (2a–2j)

Esterification of the C-17 carboxyl group resulted in retention or enhancement of many key biological activities, while selectively attenuating others. Across series “2” (compounds **2a**–**2j**, Figure 1), high P_a_ values were consistently observed for anti-inflammatory, antineoplastic, hepatoprotective, chemopreventive, apoptosis-inducing, insulin-promoting, and lipid-metabolism-regulating activities.

For example, despite the pronounced anti-inflammatory profile of oleanolic acid (**1**, Figure 1; P_a_ = 0.819), most esters in series “2” (Figure 1) retain predicted probabilities within a comparable range (approximately P_a_ = 0.753–0.809), only slightly lower than that of the parent compound (**1**). In contrast, the predicted probability of anti-inflammatory activity for the unsubstituted ester **2j** (2,3-dichloropropyl oleanolate; Figure 1) decreases below the accepted threshold (P_a_ < 0.700). This observation suggests that simple esterification of the OA carboxyl group does not abolish the core anti-inflammatory activity of the molecule; however, the specific structural modification present in the discussed derivative **2j**, namely the 2,3-dichloropropyl oleanolate moiety introduction, exerts an unfavorable effect in this activity domain.

Within series “2” (compounds **2a**–**2j**, Figure 1), structural modifications are largely confined to the ester “tail”, primarily affecting lipophilicity, steric bulk, and conformational flexibility, while preserving the triterpenoid scaffold. Such changes generally result in a fine-tuning of P_a_ values rather than a complete shift in the predicted pharmacological profile. The observed exceptions, exemplified by compound **2j** (2,3-dichloropropyl oleanolate; Figure 1), indicate that strongly electron-withdrawing and/or sterically demanding ester substituents may adversely influence the predicted activity within a given pharmacological class.

An interesting contrast is observed in the predicted antinociceptive activity. For oleanolic acid (**1**, Figure 1), the probability of this activity remains below the threshold (P_a_ < 0.700), whereas several esters from series “2” exhibit markedly higher P_a_ values, reaching approximately 0.76–0.85 (e.g., for methyl ester **2a**: P_a_ = 0.848; for ethyl ester **2b**: P_a_ = 0.829), with compound **2j** (2,3-dichloropropyl ester) again falling below the threshold (P_a_ < 0.700). These results suggest that esterification may “unlock” antinociceptive activity that is not predicted for the parent OA molecule. This effect is likely associated with ester-induced changes in ADME-related properties, particularly increased lipophilicity and improved membrane permeability, which may enhance the interaction of these derivatives with molecular targets involved in pain modulation.

Notably, hepatoprotective activity was among the most strongly predicted endpoints for series “2”, with several derivatives (e.g., **2a**, **2b**, **2f**, **2g**, and **2i**; Figure 1) exhibiting P_a_ values approaching or exceeding 0.950. This trend aligns well with the known hepatotropic properties of oleanolic acid [63] and suggests that increased lipophilicity and reduced ionization may facilitate hepatic uptake and intracellular retention.

Among the predicted activities, apoptosis-related mechanisms emerged as particularly prominent across nearly all derivatives, with high P_a_ values consistently observed for apoptosis agonism and caspase-3 and caspase-8 stimulation, indicating a strong potential for proapoptotic therapeutic applications. Oleanolic acid esters such as **2a** (methyl ester), **2b** (ethyl ester), and **2f** (isopropyl ester) (**1k**) exhibited exceptionally high probabilities (P_a_ > 0.950) for these targets.

Apoptosis agonism is a well-established and strategically significant mechanism underlying the anticancer activity of numerous therapeutic agents. Apoptosis, or programmed cell death, is an essential cellular process responsible for maintaining tissue homeostasis and eliminating damaged or transformed cells. In cancer, apoptotic pathways are frequently dysregulated, allowing malignant cells to evade death and proliferate uncontrollably. Therefore, compounds capable of reactivating or enhancing apoptotic signaling—termed apoptosis agonists—are considered promising anticancer agents. These compounds often act by stimulating either the extrinsic (death receptor-mediated) or intrinsic (mitochondrial) pathways of apoptosis, leading to the activation of key executioner enzymes such as caspase-3 and caspase-8, which are crucial for the dismantling of tumor cells [64,65].

Therapeutic agents that mimic natural death ligands (e.g., TRAIL receptor agonists = tumor necrosis factor-related apoptosis-inducing ligand) or inhibit antiapoptotic proteins like B-cell lymphoma 2 (Bcl-2) can restore apoptotic sensitivity in resistant tumor types [66]. For instance, compounds such as procaspase-activating compound-1 (PAC-1) directly activate procaspase-3 and have shown selective cytotoxicity toward cancer cells both in vitro and in vivo. The reactivation of apoptotic signaling induces cancer cell death and can sensitize tumors to chemotherapeutic agents and reduce metastatic potential. Thus, the ability of a compound to function as an apoptosis agonist is strongly associated with its potential antineoplastic effects, particularly in the context of apoptosis-resistant cancers [67].

In contrast, activities that rely on strong polar interactions—such as α-glucosidase inhibition or certain phosphatase-related endpoints—were largely absent among series “2” derivatives. The notable exception is erythrodiol (**2k**, Figure 1), which uniquely exhibited P_a_ values above 0.700 for α-glucosidase inhibition and several cancer-type-specific antineoplastic activities. This distinctive profile highlights the critical role of free hydroxyl groups at C-3 and C-28, which enhance hydrogen-bonding capacity and may enable more precise interactions with enzyme active sites.

#### 4.3.3. Consequences of the C-17 Carboxyl Reduction: Erythrodiol **2k**

Notably, erythrodiol (olean-12-ene-3β,28-diol, **2k**, Figure 1) was the only derivative among the eleven tested compounds for which the program predicted a high probability (>0.700) of exhibiting activity as an α-glucosidase inhibitor, as well as antineoplastic activity specifically against breast cancer, colon cancer, colorectal cancer, and melanoma.

This discrepancy can be rationalized by considering structural and physicochemical differences between OA esters and erythrodiol. OA esters are typically more hydrophobic and sterically hindered due to the esterification of the carboxyl group at the C-17 position. Such modifications may negatively affect the molecule’s interaction with enzyme active sites or cellular targets involved in cancer-related pathways. Specifically, the C-17 carboxyl group is crucial for hydrogen bonding and electrostatic interactions with α-glucosidase and other target proteins [68]. Esterification of this site often reduces biological activity, including antidiabetic and anticancer effects [69].

In contrast, erythrodiol (**2k**) retains both free hydroxyl groups at C-3 and C-28, allowing for greater hydrogen-bonding capacity, improved target engagement, and increased likelihood of recognition by prediction algorithms trained on activity-related structural motifs. Moreover, the absence of a bulky ester moiety results in lower steric hindrance, potentially enhancing molecular docking within active or allosteric sites of α-glucosidase or cancer-related enzymes such as topoisomerases or kinases [70,71].

#### 4.3.4. Consequences of the C-17 Carboxyl Esterification with Saturated vs. Unsaturated Alkyl Chain: Esters “d” and “e”

Within series “2” (compounds **2a**–**2j**, Figure 1), variation in ester chain length, branching, and saturation resulted primarily in quantitative rather than qualitative changes in predicted activity. While most derivatives shared a similar activity spectrum, differences in P_a_ values suggest that subtle steric and physicochemical factors fine-tune target affinity.

Based on the predictive pharmacological profiles presented in the SAR dataset, a comparative analysis of the *n*-propyl ester (compound **2c**, Figure 1), allyl ester (**2d**), and propargyl ester (**2e**) of oleanolic acid reveals notable differences in predicted biological activity, particularly in terms of potency and spectrum.

However, more distinct variations were observed in activities related to anticancer potential. The allyl ester (**2d**, Figure 1) exhibited a higher probability (P_a_ = 0.844) for general antineoplastic activity compared to the *n*-propyl ester (**2c**) with P_a_ = 0.811, while the propargyl ester (**2e**) showed slightly stronger prediction with P_a_ = 0.854. In the case of lung-cancer-specific antineoplastic activity, compound **2d** again outperformed **2c** and **2e** (P_a_ = 0.720 vs. 0.713 and ≤0.700, respectively), indicating a potential influence of the unsaturated allylic group on cellular uptake or target affinity. Such variation may be attributed to the electronic and conformational flexibility introduced by the double or triple bonds in allyl and propargyl groups, potentially affecting membrane permeability or target protein interactions [70].

Unsaturated triterpene esters—such as allyl or propargyl derivatives—are often found to exhibit enhanced pharmacological activity compared to their saturated analogues. This enhancement is commonly attributed to increased lipophilicity and more favorable interactions with cellular membranes, which together facilitate improved cellular uptake and target engagement. For instance, cinnamate-based esters of oleanolic and ursolic acids have demonstrated significantly greater cytotoxicity in cancer cell lines like HeLa, MCF-7, and HepG2 than their non-esterified or saturated ester counterparts [72]. Tanachatchairatana et al. [73] reported that introducing an unsaturated aromatic ester enhanced antimycobacterial and antiproliferative effects, a phenomenon attributed to better membrane permeability and intracellular accumulation. Moreover, enhanced lipophilicity associated with π-electron-rich unsaturated chains may improve binding affinity to intracellular targets such as caspases or kinases involved in apoptosis and cell-cycle regulation. Therefore, the structural presence of double or triple bonds within ester moieties serves as a biophysical and biochemical advantage, enhancing anticancer efficacy by optimizing membrane interaction and pharmacodynamic potential.

#### 4.3.5. Consequences of the C-17 Carboxyl Esterification with Linear vs. Branched Alkyl Chain: Esters “**f**”, “**h**”, “**i**”

Based on the computational activity data, a comparative analysis of linear (unbranched) esters of oleanolic acid—*n*-propyl (**2c**, Figure 1) and *n*-butyl (**2g**)—versus branched esters, such as isopropyl (**2f**), isobutyl (**2h**), and sec-butyl (**2i**), reveals distinct differences in predicted pharmacological activity, particularly regarding anticancer, anti-inflammatory, and apoptosis-related effects.

The linear esters **2c** and **2g** demonstrated consistently higher or comparable predicted probabilities (P_a_) across key pharmacological categories. For example, the antineoplastic activity of *n*-propyl ester (**2c**, Figure 1) was predicted at P_a_ = 0.811 and *n*-butyl ester (**2g**) at P_a_ = 0.835, whereas branched esters **2f**, **2h**, and **2i** showed slightly lower values (P_a_ = 0.808–0.820). Similar trends were observed for apoptosis agonism, with **2c** and **2g** scoring P_a_ = 0.857 and 0.869, respectively, compared to slightly lower values for the branched analogues (e.g., 0.844 for **2i**).

Branched esters can be potent but are more structure-sensitive, e.g., branched-chain fatty acid derivatives like n-butyl and phenyl esters of oleic acid showed notable antiproliferative activity (IC_50_ ≈ 48–82 ppm), suggesting that controlled branching can enhance activity [74]. However, anticancer studies on guaianolide-type sesquiterpene lactone esters revealed a size–activity optimum: adding a tertiary α-carbon (like isopropyl or isobutyl ester) improved potency by ~30–75%, but larger branches (e.g., isovaleryl) led to ~60% activity loss due to steric hindrance [75]. Excessive branching hinders molecular interactions, limiting cytotoxic effects despite increased lipophilicity [76].

#### 4.3.6. Consequences of the C-3 Furoyl Substitution: Series “3” (**3a**–**3k**)

Introduction of a furoyl moiety at the C-3 hydroxyl position led to a distinct reorganization of the predicted activity landscape. Compared with series “2” (compounds **2a**–**2k**, Figure 1), furoyl derivatives (compounds **3a**–**3k**, Figure 1) generally exhibited lower P_a_ values for many enzyme-inhibitory and antiviral endpoints, suggesting that the bulky aromatic substituent imposes steric constraints that limit access to certain active sites.

Nevertheless, series “3” retained strong predicted activity in domains closely associated with oxidative stress, inflammation, and transcriptional regulation. High P_a_ values for oxidoreductase inhibition, chemopreventive activity, hepatoprotection, and transcription factor stimulation (including NF-κB-related pathways) were consistently observed. These findings suggest that the furoyl group may enhance interactions with redox-regulated signaling proteins and transcriptional machinery, which is highly relevant in the context of antioxidant and anti-inflammatory research.

Antineoplastic activity remained present but was generally predicted with lower P_a_ values than for series “2” (**2a**–**2k**, Figure 1) or the parent compound (**1**, Figure 1). This attenuation may reflect reduced flexibility or altered molecular recognition caused by aromatic substitution. However, the relatively uniform P_a_ values across series “3” derivatives indicate that, once the C-3 position is modified, variations at the C-17 ester group exert a diminished influence on overall activity.

Direct comparison of the two derivative series reveals complementary pharmacological profiles. Series “2” derivatives are characterized by broad, multitarget activity with strong emphasis on metabolic, hepatic, antiviral, and cytoprotective endpoints. In contrast, series “3” derivatives show a more focused profile, preferentially targeting redox balance, transcription factor regulation, and chemopreventive mechanisms.

These differences can be rationalized by considering both steric and electronic effects. Esterification alone enhances lipophilicity while preserving the flexible triterpenoid framework, enabling interactions with a wide range of biological targets. Furoyl substitution, by contrast, introduces aromaticity and rigidity, which may enhance selectivity toward signaling pathways while limiting promiscuous enzyme binding.

Importantly, these in silico predictions complement the experimentally observed cytotoxic and antioxidant activities and provide a strong rationale for further validation. Integration of these SAR insights with ADMETox profiling and targeted biological assays will be essential for prioritizing derivatives with the most favorable balance between efficacy, selectivity, and drug-like properties.

### 4.4. Analysis of ADMETox Prediction Results

The ADMETox profiles of the parent compound (**1**) and its derivatives (series **2a**–**2k** and **3a**–**3k**) were evaluated using computational prediction tools. Therefore, the following discussion should be understood as an initial developability assessment rather than as a description of experimentally confirmed pharmacokinetic or safety properties. The calculated parameters indicate that structural modifications introduced in both series may substantially influence drug-likeness, permeability, solubility, metabolism-related liabilities and predicted toxicity endpoints.

#### 4.4.1. Physicochemical Properties and Drug-Likeness

All analyzed compounds exhibit relatively high molecular weights, ranging from 442.38 Da (**2k**; erythrodiol) to 660.33 Da (**3j**, 2,3-dichloropropyl 3-O-furoyl oleanolate, Figure 1), exceeding the classical Lipinski threshold of 500 Da for most derivatives (Tables S3 and S4). This trend is accompanied by large molecular volumes and high lipophilicity. The calculated LogP values are consistently elevated, with values of 6.37 for OA and increasing up to 8.55 for compound **3g** (*n*-butyl 3-O-furoyl oleanolate), indicating pronounced hydrophobic character. Similarly, LogD (the logarithm of the n-octanol/water distribution coefficient) values remain above 4.0 for all derivatives, confirming limited aqueous solubility, which is reflected in low predicted LogS values (the logarithm of aqueous solubility value, e.g., −6.71 for compound **2c**; *n*-propyl ester, and −7.38 for compound **3c**, *n*-propyl 3-O-furoyl oleanolate, Figure 1).

Topological polar surface area (TPSA) values remain below 80 Å^2^ for all compounds, ranging from 46.53 Å^2^ (most 2-series derivatives) to 78.88 Å^2^ (**3k**), suggesting that polarity is not excessive despite the high molecular weight. The fraction of sp^3^ carbons (Fsp^3^) is high across both series (0.70–0.93), indicating significant three-dimensional character, which is often associated with improved target selectivity and reduced attrition risk.

Quantitative estimates of drug-likeness (QEDs) are moderate to low, with values between 0.17 and 0.42. The parent compound OA shows a QED of 0.409, while most derivatives exhibit lower scores (e.g., 0.233 for **2g** and 0.183 for **3g**, *n*-butyl oleanolate and *n*-butyl 3-O-furoyl oleanolate, respectively; Figure 1), reflecting deviations from ideal oral drug-like space due mainly to high lipophilicity and molecular size. Synthetic accessibility scores (SA score~4.6–5.1) suggest that all compounds are of moderate synthetic complexity.

#### 4.4.2. Absorption and Permeability

Predicted intestinal permeability values indicate moderate membrane permeability. Caco-2 permeability values are consistently around −5.0 (e.g., −4.96 for **2b**; ethyl oleanolate, and −4.97 for **3e**, propargyl oleanolate; Figure 1); cancer coli-2, Caco-2, is a widely used, immortalized line of human colorectal adenocarcinoma cells serving as in vitro model for predicting human oral drug absorption, intestinal permeability, and nutrient uptake. The MDCK permeability values remain in the range of 1.0–1.9 × 10^−5^ cm/s, supporting passive diffusion as the dominant transport mechanism; Madin–Darby canine kidney (MDCK) is an in vitro cell monolayer assay used to measure membrane permeability, intestinal absorption, and active drug efflux.

Human intestinal absorption (HIA) probabilities are generally low (<0.04), consistent with the high lipophilicity and poor solubility of the compounds. Nevertheless, several derivatives show favorable oral bioavailability indicators, with F20% and F30% values (the human oral bioavailability 20% and 30%, respectively) exceeding 0.8 for selected compounds (e.g., F20% = 0.98 for **2g**, *n*-butyl oleanolate, and F30% = 0.95 for **3k**, erythrodiol; Figure 1), suggesting that formulation strategies could potentially mitigate solubility limitations.

All compounds are predicted to be non-substrates of P-glycoprotein (P-gp), while many derivatives, especially in the series “3”, show strong P-gp inhibition probabilities (e.g., 0.98 for **3a**, methyl 3-O-furoyl oleanolate, and 0.99 for **3k**, 3,28-difuroylerythrodiol; Figure 1), which may enhance intracellular exposure but also raises the possibility of drug–drug interactions.

#### 4.4.3. Distribution

Plasma protein binding (PPB) is very high for all compounds, exceeding 95% in every case and reaching up to 99.99% for compound **2j** (2,3-dichloropropyl ester; Figure 1). This suggests extensive binding to serum proteins, which may limit free drug concentrations. Consistently low unbound fractions (Fu < 6%) support this conclusion.

The predicted volume of distribution at steady state (VDss) ranges from moderate to high, increasing from 0.71 L/kg for OA to values above 2.0 L/kg for several series “3” derivatives (e.g., 2.67 L/kg for **3k**, 3,28-difuroylerythrodiol; Figure 1), indicating extensive tissue distribution. Blood–brain barrier (BBB) penetration probabilities are moderate for OA (0.58) but decrease substantially for most derivatives, particularly in the series “3” (e.g., 0.02 for **3a**, methyl 3-O-furoyl oleanolate; Figure 1), suggesting reduced central nervous system exposure.

#### 4.4.4. Metabolism and Clearance

Cytochrome P450 interaction profiles reveal that most compounds are likely inhibitors and substrates of CYP3A4, with inhibition probabilities frequently above 0.7 (e.g., 0.800 for **2e**, propargyl oleanolate, and 0.860 for **3k**, 3,28-difuroylerythrodiol; Figure 1). Interactions with other isoforms, such as CYP2C19 and CYP2D6, are moderate and compound-dependent. These results indicate a potential risk of metabolic drug–drug interactions, particularly for derivatives with strong CYP3A4 inhibition.

Predicted plasma clearance (CL) values are moderate to high (approximately 7.6–11.6 mL/min/kg), while estimated half-lives are short (<0.03 h for all compounds), suggesting rapid systemic elimination. This rapid clearance may necessitate dosing optimization or structural refinement to improve metabolic stability.

#### 4.4.5. Toxicity Assessment

The toxicity predictions indicate an overall favorable safety profile. The probability of the human ether-a-go-go related gene (hERG) channel blockade remains low for most compounds (e.g., 0.03–0.06), suggesting a low risk of cardiotoxicity. Hepatotoxicity, expressed as drug-induced liver injury (DILI), and Ames mutagenicity (the Ames *Salmonella*/microsome mutagenicity) probabilities are also low, with DILI values typically below 0.008 and Ames toxicity below 0.030.

No pan-assay interference compounds (PAINS), ALARM NMR (thiol reactive compounds), or chelator alerts were detected, indicating a low likelihood of assay interference or non-specific reactivity. Environmental toxicity indicators (e.g., aquatic toxicity rules) were triggered for several compounds, which may be relevant for environmental risk assessment but are less critical at the early drug discovery stage.

Overall, the predicted ADMETox profiles support further investigation of the compounds but also reveal important developability limitations. The low predicted risk for selected toxicity endpoints is encouraging; however, high lipophilicity, poor aqueous solubility, extensive plasma protein binding, potential CYP-mediated interactions and short predicted half-lives indicate that these derivatives cannot yet be considered drug-like candidates without further optimization. These results should be treated as computational guidance for compound prioritization. Experimental studies, including solubility determination, plasma and microsomal stability, permeability assays and CYP inhibition profiling, will be required to verify the predicted pharmacokinetic and safety behavior. Table 10 summarizes data of the selected lead compounds ranked according to cytotoxic potency, selectivity and predicted developability.

### 4.5. Anticancer Potential

#### 4.5.1. Anticancer Activity of Oleanolic Acid as a Reference Scaffold

Oleanolic acid (**1**, Figure 1) is characterized by its moderate but consistent anticancer activity across all tested cancer cell lines. The IC_50_ values obtained for OA (IC_50_ from ~9 to ~20 μM for cancer cell lines, Table 6) indicate a balanced cytotoxic effect rather than high selectivity toward a single tumor type, suggesting that its biological activity arises from the modulation of multiple cellular pathways rather than from interaction with a single molecular target [26,77]. This behavior is typical for pentacyclic triterpenes and provides a favorable foundation for structural optimization, as modest chemical modifications may selectively amplify specific biological effects while preserving the overall pharmacological spectrum of the parent compound [78,79].

#### 4.5.2. Impact of the C-17 Esterification: Series “2” (**2a**–**2j**)

Within oleanolic acid derivatives of series “2” (**2a**–**2k**, Figure 1), esterification of the C-17 carboxyl group led to a marked increase in cytotoxic potency (Table 6) relative to the parent acid (**1**), underscoring the importance of neutralizing the acidic functionality. Conversion of the carboxyl group into an ester likely reduces ionization at physiological pH, thereby increasing lipophilicity and facilitating passive diffusion across cellular membranes. Recent research in drug design and prodrug strategies confirms that esterification of carboxyl groups reduces ionization at physiological pH and increases lipophilicity, thereby facilitating passive diffusion across biological membranes, including epithelial and endothelial barriers. This fundamental principle underlies many successful modifications in medicinal chemistry aimed at improving membrane permeability and bioavailability [80].

Among derivatives of “2” series, compounds **2c** (*n*-propyl ester, Figure 1), **2i** (*sec*-butyl ester), and **2j** (2,3-dichloropropyl ester) were consistently the most potent, exhibiting IC_50_ values close to or slightly above 1 μM across all tested cancer cell lines (Table 6). Notably, this enhanced activity was largely independent of tumor type, suggesting a general cytotoxic mechanism rather than cell-line-specific susceptibility [81]. At the same time, these compounds retained higher IC_50_ values toward HDF cells, indicating a potentially favorable therapeutic window.

A closer examination of the ester moieties reveals that optimal chain length and branching are critical. While elongation of the alkyl chain from methyl and ethyl (**2a**, **2b**, respectively; Figure 1) to propyl and butyl derivatives (**2c**, **2g**, respectively) improved activity, further increases in steric bulk or unfavorable branching did not necessarily lead to additional gains (Table 6). For example, compound **2i** (sec-butyl ester) showed markedly stronger activity than its isomeric counterparts **2f** (isopropyl) and **2h** (isobutyl), highlighting the importance of subtle conformational and steric effects rather than lipophilicity alone. Subtle changes in steric and conformational features of small molecules are known to significantly influence anticancer activity by modulating molecular interactions with diverse biological targets and pathways [82].

The strong activity of the dichloro-substituted ester **2j** (Figure 1) suggests that electronic effects may also contribute to cytotoxic potency (Table 6). The presence of electron-withdrawing chlorine atoms could influence molecular recognition, metabolic stability, or intracellular retention, thereby enhancing biological activity [83].

#### 4.5.3. Impact of the C-17 Carboxyl Reduction: Erythrodiol (**2k**)

Reduction of the C-17 carboxyl group to yield erythrodiol (**2k**, Figure 1) resulted in a noticeable change in biological behavior compared with oleanolic acid (**1**) and its esters (**2a**–**2j**). Although erythrodiol (**2k**) retains measurable anticancer activity, its IC_50_ values are generally higher than those observed for the most active C-17 esters (Table 6), indicating a partial loss of cytotoxic potency. This observation highlights the importance of the carboxyl group at C-17 within the molecule of triterpene for optimal anticancer activity, possibly due to its involvement in polar interactions or its influence on molecular conformation [84]. At the same time, erythrodiol displays a distinct activity profile, suggesting that the C-17 reduction does not merely weaken biological effects but rather redirects them, leading to a functionally differentiated scaffold.

#### 4.5.4. Impact of the C-17 Carboxyl Esterification with Saturated vs. Unsaturated Alkyl Chains: Esters “**d**” and “**e**”

Comparison of esters bearing unsaturated substituents (**2d** and **2e**, bearing allyl or propargyl chains respectively; Figure 1) reveals that the degree of unsaturation at the ester side chain plays a modulatory role in anticancer activity (Table 6). Esters containing double bonds tend to show moderately reduced or comparable activity (IC_50_ ~9–10 μM) relative to their saturated counterparts (IC_50_ ~1 μM), suggesting that limited conformational rigidification may be tolerated without severely compromising cytotoxic potency [82]. In contrast, the presence of a triple bond often leads to a more pronounced decrease in activity, likely due to excessive rigidity or unfavorable electronic effects that impair optimal target engagement [85]. This observation suggests that the presence of double or triple bonds within the ester moiety does not significantly enhance activity and may even introduce conformational constraints that are unfavorable for optimal target interaction. These findings also indicate that unsaturation within the ester chain can subtly influence biological activity and must be carefully optimized to avoid detrimental effects. The results obtained for erythrodiol (**2k**) emphasize that the presence of additional hydroxyl groups—while increasing polarity—may reduce membrane permeability and thus limit cytotoxic efficacy.

#### 4.5.5. Impact of the C-17 Carboxyl Esterification with Linear vs. Branched Alkyl Chains: Esters “**f**”, “**h**”, and “**i**”

Comparison of esters bearing linear and branched alkyl chains indicates that anticancer potency is governed by an optimal balance between chain length, branching, and hydrophobic surface rather than by linearity alone. The *n*-propyl ester (**2c**) and the *sec*-butyl ester **2i** (Figure 1) displayed the strongest activity within series “2”, with IC_50_ values close to 1 µM across the tested cancer cell lines, whereas the isopropyl (**2f**), **n**-butyl (**2g**), and isobutyl (**2h**) derivatives were less potent (Table 6). These results suggest that moderate hydrophobicity combined with favorable steric orientation of the ester substituent is beneficial, while excessive chain extension or unfavorable branching may reduce activity.

The trend of anticancer activity lowering by compounds **2f**, **2h** and **2i** can be attributed primarily to steric and conformational effects. Short linear alkyl chains provide greater conformational flexibility and a more extended hydrophobic surface, which likely facilitates favorable van der Waals interactions with cellular membranes and hydrophobic regions of molecular targets, thereby improving cellular uptake and target engagement. In contrast, elongation or branching increases steric bulk around the ester moiety, which may hinder optimal molecular recognition or reduce effective membrane permeation [86].

#### 4.5.6. Impact of the C-3 Furoyl Moiety Incorporation: Series “3” (**3a**–**3k**)

The furoyl derivatives of oleanolic acid and erythrodiol (compounds **3a**–**3k**, Figure 1) exhibited a pronounced and consistent enhancement of cytotoxic activity compared to both the parent compound (**1**) and the corresponding non-acylated esters (**2a**–**2j**) and erythrodiol (**2k**). Overall, this series demonstrated low-micromolar to near-micromolar antiproliferative potency across all tested cancer cell lines (Table 6), highlighting the furoyl group as a key structural element contributing to biological activity.

The enhancement observed after C-3 furoylation may be rationalized by several structural and physicochemical factors. First, the furoyl fragment introduces a compact heteroaromatic system that increases molecular rigidity and may restrict conformational flexibility around the C-3 substituent. Second, the electron-rich furan ring can contribute to pi-related and hydrophobic interactions, as also suggested by the docking models for selected derivatives. Third, conversion of the C-3 hydroxyl group into an ester modifies the hydrogen-bonding pattern and increases lipophilicity, which may facilitate membrane association and passive cellular uptake. At the same time, excessive lipophilicity may reduce aqueous solubility and negatively affect developability. Thus, the furoyl group appears to improve cytotoxic performance in several derivatives, but this effect should be considered as a balance between increased hydrophobic/protein-binding interactions and unfavorable solubility-related liabilities.

Among the simplest furoyl esters, compounds **3a** (methyl 3-O-furoyl oleanolate) and **3b** (ethyl 3-O-furoyl oleanolate) displayed high cytotoxicity, with IC_50_ values ranging from 2.2 to 2.8 µM against cancer cells. For example, compound **3a** showed IC_50_ values of 2.59 µM in HeLa cells and 2.52 µM in MCF-7 cells, while compound **3b** exhibited slightly improved potency (IC_50_ = 2.20 µM in HeLa and 2.26 µM in MCF-7). These findings indicate that short alkyl chains combined with a furoyl moiety already confer a substantial improvement in activity relative to oleanolic acid.

Extension of the alkyl chain length led to further enhancement of cytotoxic effects. The *n*-propyl derivative **3c** demonstrated consistently lower IC_50_ values across all cancer cell lines, ranging from 1.72 µM (MCF-7) to 1.88 µM (HeLa). This trend suggests that a moderate increase in hydrophobic surface area improves interactions with cellular membranes and possibly facilitates intracellular accumulation.

Branched alkyl substituents yielded particularly favorable results. Compound **3f** (isopropyl 3-O-furoyl oleanolate) showed strong and uniform cytotoxicity, with IC_50_ values of 1.28 µM in MCF-7 cells, 1.47 µM in HeLa and A-549 cells, and 1.16 µM in SKOV-3 cells. Similarly, compound **3h** (isobutyl 3-O-furoyl oleanolate) exhibited enhanced potency, especially against SKBR-3 cells (IC_50_ = 1.12 µM), while maintaining IC_50_ values below 2 µM in all remaining cancer cell lines.

The most potent derivatives within this series were compounds **3i** and **3j**. The *sec*-butyl derivative **3i** displayed near-uniform near-micromolar to low-micromolar activity, with IC_50_ values of 1.07 µM in MCF-7 cells, 1.09 µM in SKBR-3 cells, and 1.13 µM in HeLa cells. Likewise, the dichloro-substituted compound **3j** showed IC_50_ values tightly clustered around 1.25–1.34 µM across all cancer cell lines, indicating both high potency and low cell-line dependence. The strong activity of compound **3j** may be attributed to the increased lipophilicity and electron-withdrawing nature of the dichloro substituent, which could enhance binding interactions with hydrophobic regions of molecular targets.

Finally, compound **3k**, bearing two furoyl groups on the erythrodiol scaffold, exhibited slightly reduced potency compared to the most active mono-furoyl derivatives, with IC_50_ values around 2.0–2.4 µM across cancer cell lines. This observation may indicate that excessive steric bulk or increased rigidity partially counteracts the beneficial effects of additional aromatic acylation.

The attachment of a 2-furanocarboxylate moiety to oleanolic acid esters significantly tweaks their structural and physicochemical landscape, explaining the observed contraction of predicted pharmacological activities (excluding chemoprevention). Oleanolic acid, a pentacyclic triterpenoid, relies on its predominantly hydrophobic backbone for broad activities such as anti-inflammatory, antiviral, hepatoprotective, and antitumor effects [87]. These effects often stem from its ability to interact with hydrophobic binding pockets in proteins and integrate into cell membranes [30]. The methyl ester form maintains this lipophilicity, enhancing cellular uptake and target engagement. When a furoyl moiety replaces part of this framework with its planar aromatic scaffold and polar carboxylate, the molecule becomes more polar, bulkier, and conformationally rigid. Such changes can disrupt favorable hydrophobic contacts and sterically hinder association with non-redox targets, thereby reducing binding affinity across many pathways.

Moreover, the furan ring is well known for its metabolic liability: cytochrome P450 enzymes can oxidize it into reactive intermediates like epoxides or cis-enedials [88], which often exhibit toxicity and compromise the compound’s stability and bioavailability [89]. These metabolic risks diminish the therapeutic index, further narrowing pharmacological applicability.

Interestingly, furan-containing structures are also established activators of the Nrf2 pathway and phase II detoxifying enzymes, providing a plausible explanation for retained or even enhanced chemopreventive predictions [71,90]. Such pathways involve redox-sensitive transcription factors and electrophile responses, which furan moieties can modulate through mild oxidative stress or electrophilic signaling.

In summary, the results for compounds **3a**–**3k** clearly demonstrate that furoyl moiety substitution at the C-3 position significantly enhances cytotoxic activity. Optimal activity is achieved with short to moderately branched alkyl substituents, particularly *sec*-butyl and isopropyl groups, as well as halogenated derivatives. These findings underscore compounds **3f**, **3i**, and **3j** as the most promising candidates within this series for further mechanistic and in vivo evaluation.

#### 4.5.7. Relationship Between Cytotoxic Activity and Polarity

The cytotoxic activity of the investigated oleanolic acid derivatives appears to be closely related to their physicochemical properties, particularly polarity, as inferred from TLC analysis, and to general ADMET-related considerations. Although TLC provides only qualitative information, the observed differences in R_f_ values offer useful insight into relative lipophilicity, which is a key determinant of membrane permeability, intracellular accumulation, and overall biological performance.

In general, derivatives **2a**–**2k** (Figure 1) exhibiting lower polarity, reflected by higher R_f_ values, showed enhanced cytotoxic activity compared with the parent oleanolic acid (Table 1 and Table 6). This trend is especially evident upon esterification of the C-17 carboxyl group, which reduces ionization at physiological pH and increases lipophilicity [91]. Consistent with ADMET principles, this modification likely facilitates passive diffusion across cell membranes, thereby improving cellular uptake and contributing to increased cytotoxic potency.

Within series “2”, the most active compounds (**2c**, **2i**, and **2j**) displayed intermediate polarity relative to other esters, suggesting the presence of an optimal lipophilicity range. Excessively polar derivatives may suffer from limited membrane permeability [92], whereas overly hydrophobic compounds may exhibit reduced solubility or increased non-specific interactions [93]. This non-linear relationship highlights that cytotoxic activity depends on a balance between polarity and molecular architecture rather than on lipophilicity alone.

A more pronounced correlation was observed for the 3-O-furoyl derivatives (series “3”, compounds **3a**–**3k**, Figure 1), which consistently exhibited lower polarity and higher cytotoxic activity than both the parent compound and the corresponding non-furoyl analogues (Table 1 and Table 6). The introduction of the furoyl moiety at the C-3 position appears to dominate the physicochemical profile of these compounds, resulting in relatively uniform R_f_ values and consistently low IC_50_ values across different cancer cell lines. Variations in the ester chain length or branching within the C-17 ester function had a comparatively minor influence within this series.

### 4.6. Selectivity Index

#### 4.6.1. Selectivity Index Analysis

The Selectivity Index (SI) is widely regarded as a critical parameter in studies focused on the cytotoxic and anticancer potential of medicinal preparations derived from natural sources, isolated natural compounds, as well as chemically modified derivatives of natural origin. In many instances, the SI serves as a key criterion for determining whether further investigation of a given extract or compound is scientifically justified. According to Peña-Morán et al. [94], an SI value of at least 10 is considered the minimum threshold to support the continuation of research on crude preparations or complex mixtures. By contrast, Valderrama and co-workers have proposed a lower cutoff for individual, well-defined chemical entities, suggesting that compounds intended as potential anticancer agents should exhibit an SI value of no less than 2 to be regarded as sufficiently selective [95].

In the present study, the SI values calculated for oleanolic acid (**1**, Figure 1) and its derivatives **2a**–**2k** and **3a**–**3k** (Figure 1) ranged from 1.16 to 3.25 (Table 7), indicating varying degrees of selectivity depending on the nature of the structural modification and the cancer cell line examined.

The parent compound, oleanolic acid (**1**, Figure 1), exhibits moderate selectivity across the tested cancer cell lines, with SI values ranging from 1.27 (SKBR-3) to 2.83 (A-549) (Table 7). These results confirm that OA itself possesses a certain degree of preferential cytotoxicity toward cancer cells, particularly lung adenocarcinoma cells, and thus serves as a suitable reference scaffold for further structural optimization.

Within the series of simple alkyl ester derivatives (**2a**–**2j**) and erythrodiol (**2k**), a clear differentiation in selectivity profiles can be observed. Most compounds in this group (**2b**–**2h** and **2k**) display SI values between approximately 1.5 and 1.8 across all tested cell lines (Table 7), indicating only moderate selectivity. These results suggest that small or moderately sized alkyl substituents at the C-3 position enhance cytotoxic potency but do not substantially improve discrimination between malignant and non-malignant cells. In contrast, derivatives **2i** (*sec*-butyl ester) and **2j** (2,3-dichloropropyl ester) stand out, exhibiting consistently higher SI values, frequently exceeding 2.0 and reaching up to 2.93 (Table 7). This indicates that specific structural features—namely branched alkyl chains or electron-withdrawing substituents—can significantly improve the selectivity profile, likely by influencing membrane interactions, cellular uptake, or differential metabolic processing in cancer versus non-malignant cells.

A more pronounced improvement in selectivity is observed in the furoyl-substituted series “3” (compounds **3a**–**3k**, Figure 1). In this group, several compounds demonstrate SI values above 2.0 across multiple cancer cell lines, highlighting the beneficial impact of introducing a furan moiety. In particular, derivative **3f** (isopropyl 3-O-furoyl oleanolate) exhibits the highest SI values within the entire dataset, reaching up to 3.25 for SKOV-3 cells, while maintaining SI values above 2.0 for most other cancer lines. Similarly, compounds **3b** (ethyl 3-O-furoyl oleanolate) and **3j** (2,3-dichloropropyl 3-O-furoyl oleanolate) consistently show SI values in the range of approximately 2.1–2.35 (Table 7), indicating a favorable balance between potency and selectivity.

Other furoyl derivatives, such as **3c** (*n*-propyl 3-O-furoyl oleanolate), **3e** (propargyl 3-O-furoyl oleanolate), **3g** (*n*-butyl 3-O-furoyl oleanolate), and **3h** (isobutyl 3-O-furoyl oleanolate), display intermediate SI values (approximately 1.3–1.9; Table 7). Although these compounds are highly cytotoxic, their selectivity toward cancer cells is less pronounced, suggesting that excessive cytotoxicity may also affect non-malignant cells. The lowest SI values in the dataset are observed for compound **3c** (*n*-propyl 3-O-furoyl oleanolate), indicating that, while short alkyl chains may enhance activity, they do not necessarily translate into improved selectivity.

Across the different cancer cell lines, SI values are relatively consistent, indicating that selectivity is primarily compound-dependent rather than strongly influenced by cancer type. Slightly higher SI values are observed for A-549 and SKOV-3 cells, which may reflect differences in membrane composition, metabolic capacity, or susceptibility to the tested compounds.

Overall, the SI analysis demonstrates that chemical modification of the oleanolic acid scaffold can not only enhance anticancer potency but also improve biological selectivity in selected cases. The most promising candidates—namely **2i**, **2j**, **3f**, **3b**, and **3j** (Figure 1)—combine strong cytotoxic activity with SI values exceeding the commonly accepted threshold of 2 for individual compounds, supporting their further investigation as potential anticancer leads. These findings underscore the importance of fine-tuning lipophilicity and electronic properties to achieve an optimal balance between efficacy and safety in the development of oleanolic-acid-based anticancer agents.

It should be emphasized, however, that SI values slightly above 2 indicate only moderate preferential activity toward malignant cells. Therefore, the present results should not be interpreted as evidence of strong tumor selectivity. Rather, they identify compounds that may deserve further investigation in broader panels of non-malignant cells and in more advanced toxicity models. Comparison with previously reported OA derivatives also indicates that improvement in cytotoxic potency does not automatically translate into a proportionally improved safety margin.

#### 4.6.2. Selectivity and Safety Consideration

A consistent and noteworthy trend observed across both series of oleanolic acid derivatives (**2a**–**2k** and **3a**–**3k**, Figure 1) is the systematically higher IC_50_ values determined for non-malignant human dermal fibroblasts (HDFs) compared with all tested cancer cell lines (Table 6). This difference is particularly pronounced for the most potent compounds, including derivatives **2c** (*n*-propyl ester), **2i** (*sec*-butyl ester), and **2j** (2,3-dichloropropyl ester), as well as the furoyl-substituted analogues **3f** (isopropyl 3-O-furoyl oleanolate), **3h** (isobutyl 3-O-furoyl oleanolate), **3i** (*sec*-butyl 3-O-furoyl oleanolate), and **3j** (2,3-dichloropropyl 3-O-furoyl oleanolate). While these compounds exhibit near-micromolar to low-micromolar cytotoxicity against a broad panel of malignant cells (HeLa, MCF-7, A-549, SKBR-3, PC-3, and SKOV-3), their IC_50_ values in HDF cells are consistently shifted toward higher concentrations (Table 7), indicating reduced toxicity toward non-malignant cells.

This preferential activity toward cancer cells suggests a degree of intrinsic selectivity, which may be attributed to fundamental biological differences between non-malignant and transformed cells. Potential contributing factors include alterations in membrane composition and fluidity, enhanced metabolic activity, dysregulated redox homeostasis, and increased susceptibility of cancer cells to oxidative stress and apoptosis-inducing stimuli. Such differences may facilitate greater intracellular accumulation or heightened sensitivity of malignant cells to these derivatives, while relatively sparing non-malignant fibroblasts.

The combination of high cytotoxic potency against cancer cell lines and lower activity toward HDF cells highlights several derivatives as candidates for further optimization. Nevertheless, HDF cells represent only one non-malignant in vitro model and cannot substitute for comprehensive safety assessment. Among the unsubstituted alkyl esters (**2a**–**2j**), compounds **2c**, **2i** and **2j** displayed one of the most favorable balances between cytotoxicity and SI values. In the 3-O-furoyl derivatives **3f**, **3i** and **3j** combined strong antiproliferative activity with a moderate degree of preferential activity toward malignant cells. These findings justify further investigation but should be interpreted cautiously until confirmed in additional normal cell models and mechanistic toxicity assays.

#### 4.6.3. Mechanistic Basis for the Increased Sensitivity of Cancer Cells Compared to HDF Cells

1. Altered Membrane Composition and Increased Permeability

One of the fundamental differences between cancer and normal cells lies in the composition and biophysical properties of their plasma membranes. Cancer cells exhibit significant alterations in lipid metabolism, leading to changes in membrane composition, including variations in phospholipid content, cholesterol distribution, and fatty acid saturation. These modifications result in a more dynamic and less ordered lipid bilayer [96,97]. In particular, cancer cells are characterized by increased membrane fluidity due to a higher proportion of unsaturated fatty acids and disrupted cholesterol homeostasis. This enhanced fluidity facilitates the diffusion of small molecules across the membrane and may promote the accumulation of lipophilic or amphiphilic compounds within malignant cells [98].

In contrast, normal human dermal fibroblasts (HDF cells) maintain tightly regulated membrane organization and lipid homeostasis. Consequently, the altered membrane properties of cancer cells may preferentially enhance drug uptake, contributing to their increased sensitivity to bioactive compounds [99].

2. Elevated Oxidative Stress and Redox Vulnerability

A hallmark of cancer cells is the presence of elevated levels of reactive oxygen species (ROS), resulting from increased metabolic activity, mitochondrial dysfunction, and oncogenic signaling. While moderate ROS levels support tumor growth and survival, excessive ROS can induce irreversible oxidative damage and trigger cell death pathways [100,101]. To cope with this imbalance, cancer cells develop adaptive antioxidant systems (e.g., glutathione, thioredoxin), which maintain ROS at a tolerable but elevated level. However, this places cancer cells in a fragile redox state, often described as “on the threshold” of oxidative stress tolerance.

Therapeutic agents that further increase ROS production or impair antioxidant defenses can push cancer cells beyond this threshold, leading to selective cytotoxicity. This concept has been widely recognized as a key vulnerability in cancer therapy, as normal cells such as HDFs typically exhibit lower basal ROS levels and more stable redox homeostasis [36].

3. Metabolic Reprogramming (Warburg Effect)

Cancer cells undergo profound metabolic reprogramming to sustain rapid proliferation, a phenomenon classically described as the Warburg effect. In this state, tumor cells preferentially utilize aerobic glycolysis, converting glucose to lactate even in the presence of oxygen [102,103]. Although less efficient in ATP production, this metabolic shift provides several advantages, including the generation of biosynthetic intermediates required for cell growth and survival. Importantly, it also results in an altered intracellular environment characterized by increased glycolytic flux, lactate accumulation, and acidification [102].

This metabolic dependence creates vulnerabilities, as cancer cells rely heavily on specific pathways (e.g., glycolysis, glutamine metabolism, lipid biosynthesis). Disruption of these pathways or interference with energy production can therefore preferentially affect cancer cells. In contrast, normal cells such as HDFs exhibit more flexible metabolic profiles and are less dependent on a single energy-producing pathway [102,103].

4. Differential Expression of Molecular Targets

Another critical factor underlying the selective sensitivity of cancer cells is the differential expression of molecular targets. Tumor cells frequently overexpress specific receptors, enzymes, and signaling proteins that are involved in proliferation, survival, and metastasis. For example, overexpression of receptors such as human epidermal growth factor receptor 2 (HER2) or activation of oncogenic signaling pathways (e.g., PI3K/Akt/mTOR) is commonly observed in various cancers and contributes to uncontrolled cell growth. In addition, genetic mutations and epigenetic changes can alter protein structure, expression levels, and accessibility, affecting drug binding. This differential expression provides an opportunity for selective targeting, as compounds may exhibit higher affinity or efficacy toward proteins that are abundant or uniquely active in cancer cells. Conversely, normal cells, including HDFs, often express these targets at significantly lower levels, reducing their susceptibility to the same compounds [104].

5. Integrated Interpretation

The increased sensitivity of cancer cells relative to normal fibroblasts is a multifactorial phenomenon arising from the interplay of several cancer-specific vulnerabilities. Enhanced membrane permeability promotes intracellular accumulation of compounds, while elevated oxidative stress creates a narrow window of redox tolerance that can be therapeutically exploited. Simultaneously, metabolic reprogramming introduces dependencies on specific pathways, and differential expression of molecular targets further enhances selectivity.

Together, these factors create a biological context in which cancer cells are inherently more susceptible to chemical perturbation than normal HDF cells.

In conclusion, the observed selectivity toward cancer cells can be rationalized by well-established hallmarks of cancer biology, including altered membrane composition, redox imbalance, metabolic reprogramming, and target overexpression. These interconnected mechanisms collectively contribute to the enhanced vulnerability of cancer cells and provide a strong mechanistic basis for the observed differences in sensitivity.

### 4.7. Antioxidant Potential

The parent compound, oleanolic acid (**1**), exhibited moderate activity in both assays, yielding results of 0.0569 mg TE/mL in the CUPRAC assay and 0.0206 mg TE/mL in the DPPH assay (Figure 2 and Figure 3). Structural modifications at the C-17 carboxyl group (series “2”) and the C-3 hydroxyl group (series “3”) influenced this potential in distinct ways. Importantly, both DPPH and CUPRAC are cell-free chemical assays. They provide information about radical-scavenging or reducing capacity under simplified experimental conditions, but they do not directly reflect intracellular antioxidant or pro-oxidant behavior. Therefore, the results of these assays should not be directly linked to the cytotoxic effects observed in cancer cells. Determination of intracellular ROS levels and redox-sensitive signaling pathways would be required to establish whether redox modulation contributes to the antiproliferative activity of the tested compounds.

#### 4.7.1. Effect of the C-17 Carboxyl Group Esterification and Reduction: Series “2” (**2a**–**2k**)

Most derivatives in series “2” (Figure 1) showed a reduced DPPH-radical-scavenging capacity compared to OA (values ranging from 0.0048 to 0.0134 mg TE/mL, Figure 2 and Figure 3). This indicates that the free carboxyl group at C-17 plays a significant role in the direct antioxidant mechanism of OA in this assay.

However, exceptions were observed in the CUPRAC test. Derivative **2c** (*n*-propyl ester) exhibited the highest antioxidant potential among the entire study group (0.0750 mg TE/mL, Figure 2), surpassing the activity of the parent compound. Similarly, derivative **2j** (2,3-dichloropropyl ester) achieved a high result (0.0702 mg TE/mL, Figure 2), suggesting that the presence of halogen substituents or a specific alkyl chain length may favor the reduction potential via the SET mechanism.

The reduction of the C-17 carboxyl group to a primary alcohol in the erythrodiol molecule (**2k**, Figure 1) led to a drastic decline in activity in both assays (CUPRAC: 0.0209 mg TE/mL; DPPH: 0.0019 mg TE/mL, Figure 2 and Figure 3), confirming the crucial importance of the C-17 moiety for the redox properties of the oleanane skeleton. In the DPPH assay, this loss of activity is attributed to the removal of a labile hydrogen donor; the aliphatic hydroxyl hydrogen in **2k** is more strongly bound and less prone to homolysis than the acidic carboxyl proton of OA. Furthermore, the CUPRAC results suggest that the carboxyl group serves as a more effective redox-active center in SET mechanisms, whereas its reduction to a hydroxyl group diminishes the compound’s electron-donating capacity and alters the physicochemical interactions with the copper(II) reagent complexes.

#### 4.7.2. Effect of 3-O-Furoyl Moiety Moiety Incorporation: Series “3” (**3a**–**3k**)

Blocking the C-3 hydroxyl group by introducing a furan ring (series “3”) led, in most cases, to a decrease in antioxidant activity in comparison to series “2” and OA, particularly in the DPPH assay (Figure 2 and Figure 3). DPPH values for the entire series “3” were very low (range: 0.0008–0.0105 mg TE/mL, Figure 3), which is expected due to the lack of labile hydrogen atoms (at the blocked the C-3 hydroxyl and at the esterified C-17 carboxyl) necessary for effective DPPH radical quenching.

In the CUPRAC assay, furoyl derivatives **3a**–**3k** (Figure 1) showed varied activity (Figure 2 and Figure 3). The highest results in this series were recorded for compounds **3j** (0.0535 mg TE/mL) and **3e** (0.0506 mg TE/mL), which approached the levels of oleanolic acid (0.0569 mg TE/mL). Surprisingly low, near-zero activity in the CUPRAC test (0.0001 mg TE/mL) was observed for derivative **3a** (methyl ester with a furoyl group), whereas its DPPH activity remained low (0.0068 mg TE/mL), possibly due to unfavorable changes in solubility or molecular conformation preventing interaction with the reagents.

#### 4.7.3. Proposed Mechanism of Antioxidant Activity Changes

The DPPH mechanism relies primarily on hydrogen atom transfer (HAT). Oleanolic acid possesses two polar groups potentially capable of participating in this process: the C-3 hydroxyl group and the C-17 carboxyl group. Esterification of the C-17 carboxyl (series “2”) eliminates one potential active center, while additional esterification of the C-3 hydroxyl with 2-furoic acid (series “3”) removes the second. This may help to explain the general decline in DPPH activity in the order: OA > series “2” > series “3”, although these interpretations remain speculative in the absence of direct mechanistic validation. The low activity of series “3” is consistent with the furane ring lacking labile hydrogen atoms available for effective radical scavenging via the HAT mechanism under these conditions.

The CUPRAC assay promotes the single electron transfer (SET) mechanism. The increased activity for the n-propyl ester (2c, 0.0750 mg TE/mL) and 2,3-dichloropropyl ester (2j, 0.0702 mg TE/mL) compared to OA (0.0569 mg TE/mL) suggests that these modifications may influence the electron density of the triterpene skeleton or—more likely—alter physicochemical properties (lipophilicity), facilitating interaction with the copper(II)-bis(neocuproine) chromophore in the reaction medium. These interpretations are hypothetical and would require electrochemical or computational analysis for confirmation.

Although the furane ring is an electron-rich aromatic system, its attachment to the OA skeleton did not result in a direct increase in in vitro antioxidant potential. This may be because the antioxidant potential of furan often manifests only after metabolic activation or within cellular pathways (e.g., Nrf2 activation), which simple chemical assays do not reflect.

The antioxidant activity measured in cell-free systems should not be directly linked to the antiproliferative effects observed in cancer cell lines. An apparent paradox exists between antioxidant properties in cell-free assays and cytotoxic activity in cellular models; however, these observations are not mutually exclusive. Cell-free assays measure the capacity to donate hydrogen atoms or electrons to artificial substrates, whereas within living cells the same compounds may interact with endogenous redox components in a context-dependent manner. Cancer cells typically maintain elevated baseline ROS levels and exhibit compromised antioxidant defenses, rendering them more susceptible to further oxidative perturbation [28,35]. A compound that acts as a chemical antioxidant in a simple assay may therefore promote oxidative imbalance selectively in tumor cells, while exerting less pro-oxidant pressure on normal cells with intact antioxidant capacity [28,31]. Direct measurement of intracellular ROS levels following treatment with the lead compounds would be required to confirm this hypothesis and is planned as part of future mechanistic studies.

In conclusion, chemical modifications aimed at increasing cytotoxicity were associated with a general reduction in direct in vitro antioxidant activity, with the exception of specific ester derivatives (e.g., **2c**, **2j**) that retained or even enhanced their reduction potential in the CUPRAC assay. The antioxidant behavior observed in these cell-free models may not accurately reflect intracellular redox activity and should be interpreted with appropriate caution.

### 4.8. Molecular Docking

Molecular docking was used in this study as a hypothesis-generating tool to explore possible ligand accommodation within the EGFR tyrosine kinase domain. Docking can support structure–activity interpretation by suggesting plausible ligand–protein binding modes, but it cannot demonstrate target engagement, kinase inhibition or cellular mechanism of action [105,106]. In this work, docking calculations were performed using the CB-Dock2 platform. For consistency, the largest detected cavity (C1), corresponding to the inhibitor/ATP-binding region defined by the co-crystallized ligand in the 1M17 structure, was selected for comparative analysis. Docking poses were evaluated using the scoring scheme implemented in AutoDock Vina v. 1.2.5; this approach is commonly applied in comparative docking studies and is known to provide reliable pose prediction in many cases [107]. The docking protocol was validated by redocking of erlotinib, and the reproduced pose showed RMSD < 2 Å relative to the crystallographic conformation. However, Vina scores should be interpreted only qualitatively, as approximate ranking metrics rather than experimentally determined binding free energies. Experimental EGFR kinase inhibition assays, EGFR phosphorylation analysis or downstream Akt/ERK signaling studies would be required to verify whether the predicted binding modes translate into actual EGFR pathway modulation.

The crystal structure 1M17 represents the tyrosine kinase domain of the epidermal growth factor receptor (EGFR) in complex with erlotinib, an ATP-competitive EGFR inhibitor. EGFR is a central regulator of cellular proliferation and survival signaling, and its dysregulation contributes to cancer development and progression, making it an established therapeutic target. The relevance of EGFR inhibition has been demonstrated across diverse experimental contexts, including suppression of EGFR phosphorylation and radiosensitization in colon cancer cells [108], the activity of natural EGFR-targeting tyrosine kinase inhibitors [109], the role of drug-resistant EGFR mutations in lung cancer [110], therapeutic targeting of EGFR in pancreatic cancer models [111], and the effects of erlotinib in breast cancer cells [112]. Therefore, using the 1M17 complex as a structural template allows docking to be interpreted within an experimentally validated binding region, and restricting the analysis to cavity C1 ensures that ligand comparisons are made within the same biologically relevant pocket.

Molecular docking was performed using the EGFR tyrosine kinase domain crystal structure 1M17, i.e., EGFR in complex with erlotinib. This model is well justified because erlotinib occupies the ATP-binding site, providing a structural reference for assessing ligand accommodation within the functionally relevant active region of EGFR and for exploring new potential inhibitors. Using the CB-Dock2 CurPocket procedure, five binding cavities were identified; the largest cavity (C1) had a volume of 991 Å^3^, indicating the highest capacity for ligand binding within the analyzed structure. The present discussion focuses exclusively on C1, as this pocket is both the most spacious and the most relevant to the inhibitor-occupied region in the reference complex. All evaluated oleanolic acid esters (**2c**, **2i**, **2j**, Figure 1) and their C-3 furoyl derivatives (**3c**, **3f**, **3h**, **3i**, **3j**, Figure 1) yielded favorable predicted binding energies in C1 (Vina scores ranging from −7.2 to −8.5 kcal × mol^−1^) (Table 9).

Overall, this pattern suggests that the triterpenoid core is inherently compatible with the hydrophobic architecture of C1, whereas the ester substituent and the presence of the furoyl fragment modulate the pose and the extent of stabilizing contacts.

Inspection of contact residues indicates that most ligands occupy a region consistent with the inhibitor-binding environment, repeatedly engaging residues such as GLY697–GLY700, PHE699, VAL702, LYS721, LEU723/ARG724, SER728, LYS730/ALA731, GLU734, ILE735, and GLU738, as well as residues deeper in the pocket, including ASP813, ARG817, ASN818, ASP831, PHE832, GLY833, LEU834, LYS851, VAL852 and PRO853.

The recurrent involvement of residues located near functionally relevant regions of the kinase domain is consistent with possible ligand accommodation within the ATP-binding environment. However, this observation should not be interpreted as evidence of functional EGFR inhibition.

The top-ranked **2i** established extensive contacts within C1, including PHE699, LYS721, LEU723, GLU734/GLU738, ASP813, ARG817, ASN818, ASP831 and LEU834.

This indicates that complex stabilization is driven largely by hydrophobic complementarity of the bulky triterpenoid scaffold within the spacious pocket, while polar residues (e.g., ARG817/ASN818/GLU734) likely contribute to orienting the ester functionality. For the C-3 furoyl derivatives (**3c**, **3f**, **3h**, **3i**, **3j**, Figure 1), additional contacts extending toward residues such as TRP856 and the 848–850 region were observed, suggesting improved pocket filling and potential π/hydrophobic contributions from the heteroaromatic moiety.

Notably, ester **2j** (2,3-dichloropropyl oleanolate) exhibited a distinct contact profile, involving residues such as LYS782–ASN784 and more C-terminal parts of the kinase domain. This may reflect an alternative orientation within the large C1 cavity (or binding at its periphery), consistent with the high capacity and conformational permissiveness of the C1 pocket.

Overall, the docking results suggest that the bulky triterpenoid scaffold is compatible with the hydrophobic character of the EGFR ATP-binding region, while the ester and furoyl substituents may modulate pose orientation and pocket filling. In particular, the furoyl group may contribute additional hydrophobic and pi-related contacts. Nevertheless, the absence of experimental EGFR inhibition or phosphorylation data means that EGFR should be regarded only as a putative molecular target. The observed cytotoxicity may result from other mechanisms, including membrane effects, redox imbalance, apoptosis-related pathways or broader triterpenoid-associated cellular responses.

## 5. Hypothetical Considerations on the Influence of the Furoyl Moiety on the Structural and Biological Properties of Triterpene Derivatives

### 5.1. Influence on Molecular Conformation

Triterpenes are highly lipophilic, polycyclic frameworks characterized by a rigid, three-dimensional architecture composed of fused rings. The introduction of a furoyl moiety into such a scaffold represents the incorporation of a planar, heteroaromatic fragment bearing a conjugated carbonyl group. This structural modification significantly impacts the conformational landscape of the resulting derivatives [77,113].

The furoyl group, due to its intrinsic rigidity and planarity, imposes local conformational constraints near the substitution site through π-conjugation and restricted rotation around the ester linkage. In the context of a triterpene skeleton, which already exhibits limited flexibility, the addition of a furoyl substituent can further reduce conformational entropy and favor specific orientations of substituents at functional positions (e.g., C-3 or C-28 in pentacyclic triterpenes).

Moreover, steric interactions between the furoyl group and adjacent substituents on the triterpene core may lead to preferred conformers that optimize both intramolecular interactions and exposure of the aromatic ring. This conformational bias is particularly relevant for biological activity, as it can preorganize the molecule for more efficient binding to target proteins by minimizing conformational rearrangements upon complex formation.

### 5.2. Electronic Distribution and Intramolecular Polarization

The incorporation of a furoyl moiety into a triterpene framework introduces a localized π-electron system into an otherwise largely saturated hydrocarbon structure. The furan ring is an electron-rich heteroaromatic system, with significant electron density delocalized across the ring due to participation of an oxygen lone pair in the π-system.

When conjugated with a carbonyl group, as in the furoyl fragment, this electron-rich ring modulates the electronic properties of the carbonyl functionality. Specifically:

- the carbonyl carbon becomes more electrophilic,
- charge delocalization occurs over the furan-carbonyl system,
- intramolecular charge transfer stabilizes specific electronic states.

In triterpene derivatives, this results in the creation of a distinct polar region within an otherwise non-polar molecular environment. Consequently, the molecule exhibits an amphiphilic character, with a hydrophobic triterpene core and a more polar, electronically active furoyl substituent.

This redistribution of electron density can also influence long-range intramolecular interactions, including dipole–dipole interactions and weak hydrogen bonding (e.g., between the carbonyl oxygen and nearby functional groups on the triterpene scaffold). Such effects may subtly affect conformational equilibria and reactivity patterns [114].

### 5.3. Impact on Cell Membrane Permeability

Triterpenes are generally characterized by high lipophilicity, which facilitates their partitioning into lipid bilayers but may limit aqueous solubility. Functionalization with a furoyl group alters this balance by introducing both polar and aromatic features.

The furan ring can act as a bioisosteric replacement for phenyl groups, often reducing overall lipophilicity while maintaining favorable interactions with hydrophobic environments. Furthermore, the carbonyl group contributes hydrogen-bond acceptor capacity, increasing polarity and potentially improving solubility.

As a result, triterpene-furoyl derivatives typically exhibit a more balanced hydrophilic–lipophilic profile compared to the parent compounds. This balance can enhance passive diffusion across cell membranes by:

- enabling sufficient lipophilic interaction with the lipid bilayer (via the triterpene core and aromatic ring),
- while maintaining adequate polarity for desolvation and re-entry into aqueous intracellular environments.

Additionally, studies on heterocycle-containing systems indicate that planar aromatic moieties such as furan can improve membrane permeability by contributing to compact molecular geometry and partial shielding of polar groups. In triterpene systems, this effect may counteract excessive hydrophobicity and improve bioavailability [115].

### 5.4. Protein-Binding Interactions

The introduction of a furoyl fragment into a triterpene scaffold substantially enhances its capacity for specific interactions with protein targets. While the native triterpene core primarily engages in hydrophobic and van der Waals interactions, the furoyl group adds multiple interaction modalities.

The aromatic furan ring can participate in π–π stacking interactions with aromatic residues (e.g., phenylalanine, tyrosine, tryptophan) and contributes to shape complementarity within hydrophobic binding pockets. At the same time, the ring oxygen and the carbonyl oxygen serve as hydrogen-bond acceptors, enabling directional interactions with polar amino acid residues.

The electron-rich nature of the furan ring also enhances dispersion interactions, while the polarized carbonyl group can engage in dipole–dipole interactions, collectively strengthening ligand–protein binding. In some cases, the electrophilic character of the carbonyl carbon in the furoyl group may permit covalent or quasi-covalent interactions with nucleophilic residues (e.g., cysteine or serine), which is exploited in the design of chemical probes [114].

## 6. Conclusions

This study demonstrates that targeted modification of the oleanolic acid scaffold at the C-17 and C-3 positions can substantially influence cytotoxic potency, selectivity, chemical redox behavior and predicted developability. C-17 esterification improved cytotoxic activity compared with parent OA, while C-3 furoylation further enhanced antiproliferative effects in several derivatives. Among the evaluated compounds, **2i**, **2j**, **3f** and **3j** showed the most balanced profiles when cytotoxic potency, SI values and predicted ADMETox limitations were considered together, whereas **3i** may be regarded as a potency-focused candidate requiring further optimization.

The selectivity indices obtained for selected derivatives indicate moderate preferential activity toward malignant cells, but they should not be interpreted as evidence of strong tumor selectivity. Likewise, the DPPH and CUPRAC results describe cell-free chemical redox behavior and do not establish intracellular antioxidant or pro-oxidant mechanisms. Molecular docking suggested favorable accommodation of selected derivatives within the EGFR ATP-binding region; however, EGFR engagement remains hypothetical and requires experimental validation.

The predicted ADMETox profiles revealed both encouraging and limiting features. While selected toxicity-related endpoints were predicted to be low, high lipophilicity, poor aqueous solubility, high molecular weight, potential CYP-mediated interactions and short predicted half-lives represent important developability challenges. Future studies should therefore focus on experimental mechanism-of-action validation, including apoptosis, intracellular ROS, cell-cycle and EGFR pathway assays, as well as pharmacokinetic optimization through solubility improvement, metabolic stability testing and formulation strategies.

## 7. Translational Limitations and Future Directions

Highly lipophilic natural-product-derived anticancer compounds often face translational barriers that are not captured by cytotoxic potency alone. These include poor aqueous solubility, limited and variable oral absorption, extensive plasma protein binding, rapid metabolic clearance and formulation difficulties. The present derivatives share several of these limitations, particularly high LogP values and low predicted solubility. Therefore, further development should not prioritize potency alone. Instead, compound ranking should integrate cytotoxic activity, SI values, predicted solubility, permeability, metabolic stability and safety-related liabilities.

For the most active derivatives, formulation-based approaches such as lipid-based carriers, polymeric nanoparticles, cyclodextrin complexes or other solubilizing delivery systems may help overcome poor aqueous solubility. In parallel, structural optimization aimed at reducing excessive lipophilicity, introducing polar or ionizable groups, or designing hydrolysable prodrugs may improve pharmacokinetic behavior. These strategies should be combined with experimental ADMET validation, including solubility, permeability, plasma stability, microsomal stability and CYP inhibition assays.

A second important limitation of the present study is the absence of direct mechanistic cellular assays. The cytotoxicity results identify potent derivatives, but they do not establish whether the observed antiproliferative effects involve apoptosis induction, cell-cycle arrest, intracellular ROS modulation, mitochondrial dysfunction or EGFR-related signaling. Therefore, future work should include Annexin V/propidium iodide staining, caspase activation assays, cell-cycle analysis, intracellular ROS measurements and selected pathway studies such as p-EGFR/EGFR and downstream Akt/ERK analysis. These experiments will be necessary to determine whether the compounds act through one dominant mechanism or through broader, triterpenoid-associated pleiotropic effects.

The results obtained in the present study, together with accumulated literature data on triterpenoid derivatives, provide a coherent framework for directing subsequent research toward both structural optimization and biological validation. The investigated compounds demonstrated promising biological potential accompanied by acceptable predicted safety parameters; however, their physicochemical characteristics—particularly high lipophilicity, large molecular size, and limited aqueous solubility—remain the primary factors restricting pharmacokinetic performance. Future studies should therefore prioritize rational structural modification aimed at improving drug-likeness without compromising biological activity.

In particular, semisynthetic strategies introducing polar functionalities, ionizable groups, or cleavable linkers appear highly justified. Numerous reports on oleanane-type derivatives indicate that selective oxidation, esterification, amide formation, and conjugation with small heterocycles or aromatic fragments can substantially enhance solubility, permeability, and target selectivity. Prodrug approaches should also be considered, especially for highly lipophilic scaffolds, as enzymatically triggered hydrolysis may improve oral absorption while maintaining intracellular delivery of the active parent compound.

Equally important will be an experimental validation of the predicted ADMET profile. The next stage should include in vitro metabolic stability studies using liver microsomes, plasma stability assays, CYP inhibition panels, and permeability experiments (e.g., PAMPA or Caco-2 models). These assays will verify computational predictions concerning bioavailability, clearance, and drug–drug interaction risk. Additionally, plasma protein-binding and solubility measurements are necessary to establish realistic pharmacokinetic expectations.

Given the frequently reported pleiotropic mechanism of triterpenoids, further mechanistic biological studies are also strongly recommended. Investigation of apoptosis induction, cell-cycle arrest, oxidative stress modulation, and signaling pathway regulation (e.g., NF-κB, MAPK, PI3K/Akt) would clarify structure–activity relationships beyond simple cytotoxicity measurements. Such experiments should be complemented by molecular docking and target identification studies to determine potential primary biological targets.

Finally, considering the tendency of triterpenoids to accumulate in membranes and intracellular organelles, formulation-based approaches may significantly influence biological performance. Nanoformulations, lipid carriers, and cyclodextrin inclusion complexes should therefore be explored alongside chemical modification strategies. Integration of medicinal chemistry, computational modeling, pharmacokinetics, and mechanistic biology will be essential to transform the currently studied derivatives from promising bioactive molecules into viable preclinical drug candidates.

## Figures and Tables

**Table 1:** The comparison of R_f_ values for oleanolic acid (**1**) and its derivatives (**2a**–**2k** and **3a**–**3k**). **Legend**: **OA** = oleanolic acid (reference compound); **R_f_** = retention factor; **v:v** = volume ratio; **AcOEt** = ethyl acetate; **C_6_H_6_** = benzene; **CH_2_Cl_2_** = methylene chloride; **values written in *italics*** = the alcohol part of the oleanolic acid ester is branched; **values underlined** = the alcohol part of the ester contains a multiple bond.

≥ 0.90	0.89—0.80		0.79—0.70		0.69—0.60		0.59—0.50		0.49—0.40		0.39—0.30		0.29—0.20		0.19—0.10	≤ 0.09	
**Comp. No.**		**R_f_ value**															
		**AcOEt**		**C_6_H_6_:AcOEt (*v*:*v*)**													**C_6_H_6_**
				**1:1**		**2:1**		**4:1**		**9:1**		**15:1**		**25:1**			
**1**		0.86		0.77		0.64		0.29		0.16		0.05		---			---
**2a**		≥ 0.90		0.88		0.75		0.69		0.49		0.05		---			---
**2b**		0.87		0.78		0.69		0.59		0.37		0.27		0.23			0.09
**2c**		0.86		0.83		0.75		0.60		0.40		0.38		0.29			0.12
**2d**		0.88		0.76		0.74		0.61		0.46		0.33		0.29			0.05
**2e**		0.89		0.76		0.71		0.58		0.36		0.30		0.24			0.02
***2f***		*≥* *0.90*		*0.84*		*0.75*		*0.61*		*0.39*		*0.31*		*0.23*			*0.07*
**2g**		≥ 0.90		0.83		0.77		0.63		0.45		0.33		0.24			0.09
***2h***		*≥* *0.90*		*0.81*		*0.78*		*0.69*		*0.49*		*0.37*		*0.23*			*0.10*
***2i***		*≥* *0.90*		*0.77*		*0.72*		*0.58*		*0.41*		*0.31*		*0.20*			*0.06*
**2j**		0.89		0.84		0.67		0.56		0.40		0.27		0.17			0.07
**2k**		0.83		0.80		0.60		0.55		0.37		0.19		0.10			0.05
**3a**		≥ 0.90		≥ 0.90		≥ 0.90		0.88		0.76		0.69		0.63			0.59
**3b**		≥ 0.90		≥ 0.90		≥ 0.90		≥ 0.90		0.82		0.67		0.60			0.42
**3c**		≥ 0.90		≥ 0.90		≥ 0.90		0.88		0.77		0.66		0.58			0.41
**3d**		≥ 0.90		≥ 0.90		≥ 0.90		0.87		0.81		0.75		0.69			0.59
**3e**		≥ 0.90		≥ 0.90		≥ 0.90		0.89		0.80		0.63		0.58			0.45
***3f***		*≥* *0.90*		*≥* *0.90*		*≥* *0.90*		*0.87*		*0.80*		*0.69*		*0.60*			*0.40*
**3g**		≥ 0.90		≥ 0.90		≥ 0.90		0.88		0.78		0.64		0.53			0.39
***3h***		*≥* *0.90*		*≥* *0.90*		*≥* *0.90*		*0.88*		*0.79*		*0.65*		*0.61*			*0.37*
***3i***		*≥* *0.90*		*≥* *0.90*		*≥* *0.90*		*0.89*		*0.73*		*0.70*		*0.59*			*0.48*
**3j**		≥ 0.90		≥ 0.90		≥ 0.90		0.89		0.81		0.74		0.64			0.62
**3k**		≥ 0.90		≥ 0.90		≥ 0.90		≥ 0.90		0.85		0.74		0.64			0.46

**Table 2:** Predicted activity of oleanolic acid (**1**) and its derivatives **2a**–**2k** determined by the PASS method. **Legend: OA** = oleanolic acid; **P_a_** = probability of activity; **P_i_** = probability of inactivity; **ag.** = agonist; **stim.** = stimulant; **prev.** = preventive; **prot.** = protectant; **prom.** = promoter; **LM** = lipid metabolism; **reg.** = regulator; **MI** = membrane integrity; **ant.** = antagonist; **MM** = mucomembraneous; **OR** = oxidoreductase; **inh.** = inhibitor; **TF** = transcription factor; **stim.** = stimulant; **values written in *italics*** = the alcohol part of the oleanolic acid ester is branched; **values underlined** = the alcohol part of the ester contains a multiple bond.

1.000—0.950	0.949—0.900			0.899—0.850			0.849—0.800			0.799—0.750			0.749—0.700			≤0.700		
**activity**		**P_a_ factor (and P_i_ factor) of compounds 1 and 2a—2k (P_a_ ≥ 0.700)**																
		**OA (1)**	**2a**		**2b**	**2c**		**2d**	**2e**		***2f***	**2g**		***2h***	***2i***		**2j**	**2k**
**Antineoplastic**		0.876 (0.005)	0.867 (0.005)		0.834 (0.008)	0.811 (0.010)		0.844 (0.007)	0.854 (0.007)		*0.835 (0.008)*	0.808 (0.011)		*0.809 (0.011)*	*0.820 (0.009)*		0.794 (0.013)	0.920 (0.005)
**Apoptosis ag.**		0.901 (0.004)	0.890 (0.004)		0.868 (0.005)	0.857 (0.005)		0.888 (0.005)	0.849 (0.005)		*0.869 (0.005)*	0.857 (0.005)		*0.844 (0.005)*	*0.854 (0.005)*		0.781 (0.009)	0.892 (0.004)
**Caspase 3 stim.**		0.984 (0.002)	0.983 (0.002)		0.974 (0.002)	0.978 (0.002)		0.976 (0.002)	0.884 (0.004)		*0.969 (0.002)*	0.965 (0.002)		*0.949 (0.003)*	*0.953 (0.003)*		0.710 (0.010)	0.971 (0.002)
**Caspase 8 stim.**		0.914 (0.001)	0.916 (0.001)		0.863 (0.001)	0.885 (0.001)		0.883 (0.001)	0.810 (0.002)		*0.876 (0.001)*	0.890 (0.001)		*0.838 (0.001)*	*0.868 (0.001)*		0.755 (0.003)	0.878 (0.001)
**Chemo-prev.**		0.937 (0.002)	0.944 (0.002)		0.918 (0.002)	0.877 (0.003)		0.952 (0.002)	0.826 (0.003)		*0.866 (0.003)*	0.880 (0.003)		*0.912 (0.002)*	*0.841 (0.003)*		0.743 (0.005)	0.852 (0.003)
**Hepato-prot.**		0.930 (0.002)	0.969 (0.001)		0.965 (0.001)	0.948 (0.002)		0.956 (0.001)	0.915 (0.002)		*0.981 (0.001)*	0.967 (0.001)		*0.968 (0.001)*	*0.958 (0.001)*		0.803 (0.004)	0.906 (0.002)
**Insulin prom.**		0.869 (0.004)	0.978 (0.001)		0.982 (0.001)	0.975 (0.001)		0.950 (0.002)	0.942 (0.002)		*0.977 (0.001)*	0.970 (0.001)		*0.977 (0.001)*	*0.973 (0.001)*		0.930 (0.002)	0.970 (0.001)
**LM reg.**		≤0.700	0.833 (0.005)		0.928 (0.003)	0.884 (0.004)		0.960 (0.002)	≤0.700		*0.923 (0.003)*	0.922 (0.003)		*0.890 (0.004)*	*0.854 (0.004)*		≤0.700	0.815 (0.005)
**MI ant.**		0.928 (0.002)	0.914 (0.002)		0.930 (0.002)	0.928 (0.002)		0.874 (0.004)	0.874 (0.004)		*0.910 (0.002)*	0.941 (0.001)		*0.911 (0.002)*	*0.909 (0.002)*		0.817 (0.006)	0.898 (0.003)
**MM prot.**		0.894 (0.005)	0.807 (0.017)		0.804 (0.017)	0.808 (0.016)		0.872 (0.006)	≤0.700		*0.783 (0.023)*	0.815 (0.015)		*0.808 (0.016)*	*0.758 (0.032)*		≤0.700	0.824 (0.013)
**OR inh.**		0.904 (0.002)	0.879 (0.003)		0.912 (0.002)	0.892 (0.002)		0.924 (0.001)	0.851 (0.004)		*0.868 (0.003)*	0.901 (0.002)		*0.896 (0.002)*	*0.859 (0.004)*		0.878 (0.003)	0.888 (0.003)
**TF stim.**		0.954 (0.001)	0.934 (0.001)		0.930 (0.001)	0.925 (0.001)		0.931 (0.001)	0.918 (0.001)		*0.935 (0.001)*	0.922 (0.001)		*0.925 (0.001)*	*0.926 (0.001)*		0.903 (0.001)	0.931 (0.001)
**TF NF κB stim.**		0.954 (0.001)	0.934 (0.001)		0.930 (0.001)	0.925 (0.001)		0.931 (0.001)	0.918 (0.001)		*0.935 (0.001)*	0.922 (0.001)		*0.925 (0.001)*	*0.926 (0.001)*		0.903 (0.001)	0.931 (0.001)

**Table 3:** Predicted activity of oleanolic acid (**1**) and its derivatives **3a**–**3k** determined by the PASS method. **Legend: OA** = oleanolic acid; **P_a_** = probability of activity; **P_i_** = probability of inactivity; **ag.** = agonist; **stim.** = stimulant; **prev.** = preventive; **prot.** = protectant; **prom.** = promoter; **MI** = membrane integrity; **ant.** = antagonist; **OR** = oxidoreductase; **inh.** = inhibitor; **TF** = transcription factor; **values written in italics** = the alcohol part of the oleanolic acid ester is branched; **values underlined** = the alcohol part of the ester contains a multiple bond.

1.000—0.950	0.949—0.900			0.899—0.850			0.849—0.800			0.799—0.750			0.749—0.700			≤0.700		
**activity**		**P_a_ factor (and P_i_ factor) of compounds 1 and 3a—3k (P_a_** **≥** ** 0.700)**																
		**OA (1)**	**3a**		**3b**	**3c**		**3d**	**3e**		***3f***	**3g**		***3h***	***3i***		**3j**	**3k**
**Apoptosis ag.**		0.901 (0.004)	0.849 (0.005)		0.818 (0.007)	0.804 (0.008)		0.852 (0.005)	0.793 (0.008)		*0.819 (0.007)*	0.804 (0.008)		*0.785 (0.009)*	*0.799 (0.008)*		0.706 (0.014)	0.812 (0.007)
**Caspase 8 stim.**		0.914 (0.001)	0.830 (0.001)		0.766 (0.002)	0.794 (0.002)		0.792 (0.002)	≤0.700		*0.783 (0.002)*	0.800 (0.002)		*0.730 (0.003)*	*0.772 (0.002)*		≤0.700	0.755 (0.003)
**Chemo-prev.**		0.937 (0.002)	0.956 (0.001)		0.941 (0.002)	0.912 (0.002)		0.962 (0.001)	0.856 (0.003)		*0.904 (0.002)*	0.915 (0.002)		*0.937 (0.002)*	*0.874 (0.003)*		0.786 (0.004)	0.880 (0.003)
**Hepato-prot.**		0.930 (0.002)	0.896 (0.003)		0.891 (0.003)	0.866 (0.003)		0.878 (0.003)	0.801 (0.004)		*0.919 (0.002)*	0.893 (0.003)		*0.895 (0.003)*	*0.880 (0.003)*		≤0.700	0.825 (0.004)
**Insulin prom.**		0.869 (0.004)	0.839 (0.004)		0.873 (0.003)	0.805 (0.004)		≤0.700	≤0.700		*0.823 (0.004)*	0.764 (0.004)		*0.828 (0.004)*	*0.791 (0.004)*		≤0.700	0.713 (0.005)
**MI ant.**		0.928 (0.002)	0.773 (0.009)		0.849 (0.004)	0.841 (0.005)		≤0.700	≤0.700		*0.753 (0.010)*	0.880 (0.003)		*0.759 (0.010)*	*0.746 (0.011)*		≤0.700	≤700
**OR inh.**		0.904 (0.002)	0.772 (0.008)		0.860 (0.004)	0.816 (0.005)		0.876 (0.003)	≤0.700		*0.731 (0.012)*	0.841 (0.004)		*0.829 (0.005)*	*≤0.700*		0.768 (0.009)	0.779 (0.008)
**TF NF κB stim.**		0.954 (0.001)	0.892 (0.002)		0.886 (0.002)	0.879 (0.002)		0.891 (0.002)	0.869 (0.002)		*≤0.700*	0.876 (0.002)		*0.880 (0.002)*	*0.882 (0.002)*		0.848 (0.002)	0.860 (0.002)
**TF stim.**		0.954 (0.001)	0.892 (0.002)		0.886 (0.002)	0.879 (0.002)		0.891 (0.002)	0.869 (0.002)		*0.894 (0.002)*	0.876 (0.002)		*0.880 (0.002)*	*0.882 (0.002)*		0.848 (0.002)	0.860 (0.002)

**Table 4:** ADMETox data for oleanolic acid (**1**) and its derivatives **2a**–**2k**. **Legend:** For values given in a range 0.000–1.000 (unless stated otherwise): 0.000–0.300 = low probability, 0.301–0.699 = moderate probability, 0.700–1.000 = high probability; * = alerts, **Perm.** = permeability; **Inhib.** = inhibitor; **Sub.** = substrate; **Penetr.** = penetration; **Tox.** = toxicity; **Sensit**. = sensitization; **Carcinogen.** = carcinogenicity; **values written in *italics*** = the alcohol part of the oleanolic acid ester is branched; **values underlined** = the alcohol part of the ester contains a multiple bond. **Explanation of parameters and optimal values**: https://admetmesh.scbdd.com/explanation/index (accessed on 29 June 2026).

High value (optimal)			Moderate value				Low value					Neutral value			
**Physicochem. Properties**	**Compound number**														
	**1**	**2a**		**2b**	**2c**	**2d**		**2e**	***2f***	**2g**	***2h***		***2i***	**2j**	**2k**
**TPSA**	57.530	46.530		46.530	46.530	46.530		46.530	*46.530*	46.530	*46.530*		*46.530*	46.530	40.60
**LogS**	−4.933	−6.212		−6.493	−6.714	−6.501		−6.582	*−6.523*	−6.829	*−6.346*		*−6.778*	−6.279	−5.769
**LogD**	4.781	4.227		5.179	5.278	5.105		5.162	*5.361*	5.437	*5.501*		*5.378*	5.288	4.705
**LogP**	6.370	5.149		6.937	7.285	7.238		6.593	*7.104*	7.704	*7.574*		*7.522*	7.244	6.538
**SAscore**	4.589	4.608		4.605	4.647	4.736		4.812	*4.707*	4.636	*4.687*		*4.822*	4.990	4.702
**Fsp3**	0.900	0.903		0.906	0.909	0.848		0.848	*0.909*	0.912	*0.912*		*0.912*	0.909	0.933
**Npscore**	3.272	3.175		2.983	2.932	2.969		2.780	*2.830*	2.896	*2.903*		*2.783*	2.926	3.326
**Caco-2 Perm.**	−5.216	−4.963		−4.959	−4.963	−4.922		−4.973	*−4.940*	−4.979	*−5.011*		*−4.926*	−5.081	−4.867
**MDCK Perm.**	1.2 × 10^−5^	1.2 × 10^−5^		1.2 × 10^−5^	1.1 × 10^−5^	1.3 × 10^−5^		1.2 × 10^−5^	*1.3* × *10^−5^*	1.0 × 10^−5^	*1.1* × *10^−5^*		*1.1* × *10^−5^*	9.8 × 10^−6^	8.8 × 10^−6^
**F30%**	0.874	0.903		0.859	0.938	0.437		0.851	*0.668*	0.958	*0.870*		*0.847*	0.732	0.952
**BBB Penetr.**	0.577	0.368		0.334	0.352	0.368		0.551	*0.361*	0.340	*0.325*		*0.382*	0.685	0.562
**PPB**	96.976	97.028		98.103	98.371	97.275		97.837	*98.545*	98.673	*98.616*		*98.609*	99.990	98.236
**VDss**	0.707	1.004		1.069	1.007	0.960		1.109	*1.233*	1.058	*1.082*		*1.242*	1.293	1.194
**CYP2C19 Sub.**	0.901	0.944		0.939	0.942	0.930		0.919	*0.942*	0.941	*0.948*		*0.953*	0.946	0.924
**CYP2C9 Inh.**	0.179	0.215		0.210	0.192	0.220		0.361	*0.153*	0.169	*0.137*		*0.169*	0.179	0.187
**CYP2C9 Sub.**	0.758	0.254		0.157	0.215	0.303		0.189	*0.133*	0.236	*0.186*		*0.171*	0.263	0.124
**CYP3A4 Sub.**	0.227	0.577		0.583	0.524	0.655		0.719	*0.557*	0.505	*0.571*		*0.690*	0.698	0.455
**CLplasma**	3.813	10.512		11.094	11.328	11.584		7.605	*11.224*	10.870	*11.439*		*10.550*	10.129	10.509
**T1/2**	0.021	0.011		0.007	0.006	0.006		0.008	*0.008*	0.006	*0.006*		*0.006*	0.008	0.017
**Skin Sensit.**	0.046	0.050		0.084	0.110	0.169		0.111	*0.071*	0.146	*0.133*		*0.084*	0.070	0.076
**Carcinogen.**	0.095	0.069		0.069	0.059	0.245		0.328	*0.096*	0.057	*0.073*		*0.087*	0.272	0.067
**Eye Corrosion**	0.030	0.006		0.004	0.004	0.004		0.004	*0.006*	0.004	*0.004*		*0.005*	0.004	0.006
**Eye Irritation**	0.066	0.022		0.039	0.046	0.058		0.040	*0.021*	0.046	*0.069*		*0.018*	0.027	0.022
**BCF**	1.815	2.868		2.866	2.418	2.952		2.549	*3.003*	2.177	*2.632*		*2.809*	2.647	3.075
**IGC_50_**	4.971	5.170		5.246	5.318	5.232		5.220	*5.209*	5.431	*5.371*		*5.247*	5.399	5.176
**LC_50_DM**	6.345	6.174		6.797	6.790	6.799		6.948	*6.695*	6.792	*6.751*		*6.812*	6.851	6.676
**LC_50_FM**	5.884	6.144		6.339	6.357	6.726		6.648	*6.090*	6.446	*6.444*		*6.191*	6.537	6.126
**NR-AhR**	0.001	0.000		0.000	0.000	0.000		0.001	*0.000*	0.000	*0.000*		*0.000*	0.000	0.000
**NR-AR**	0.369	0.460		0.039	0.035	0.057		0.032	*0.035*	0.030	*0.026*		*0.160*	0.029	0.055
**NR-AR-LBD**	0.479	0.739		0.673	0.726	0.839		0.833	*0.435*	0.678	*0.289*		*0.750*	0.713	0.355
**NR-Aromatase**	0.798	0.630		0.637	0.604	0.548		0.637	*0.610*	0.575	*0.545*		*0.575*	0.674	0.702
NB Rule *	0	0		0	0	0		0	*0*	0	*0*		*0*	0	0
NGC Rule *	0	0		0	0	0		0	*0*	0	*0*		*0*	1	0
ChEMBL Rule *	0	0		0	0	0		1	*0*	0	*0*		*0*	0	0

**Table 5:** ADMETox data for oleanolic acid (**1**) and its derivatives **3a**–**3k**. **Legend**: For values given in a range 0.000–1.000 (unless stated otherwise): 0.000–0.300 = low probability, 0.301–0.699 = moderate probability, 0.700–1.000 = high probability; * = alerts, **Perm.** = permeability; **Inhib.** = inhibitor; **Sub.** = substrate; **Penetr.** = penetration; **Tox.** = toxicity; **Sensit.** = sensitization; **Carcinogen.** = carcinogenicity; **values written in *italics*** = the alcohol part of the oleanolic acid ester is branched; **values underlined** = the alcohol part of the ester contains a multiple bond; **Explanation of parameters and optimal values**: https://admetmesh.scbdd.com/explanation/index (accessed on 29 June 2026).

High value (optimal)			Moderate value				Low value					Neutral value			
**Physicochem. Properties**	**Compound number**														
	**1**	**3a**		**3b**	**3c**	**3d**		**3e**	***3f***	**3g**	***3h***		***3i***	**3j**	**3k**
**TPSA**	57.530	65.74		65.740	65.740	65.740		65.740	*65.740*	65.740	*65.740*		*65.740*	65.740	78.880
**LogS**	−4.933	−7.140		−7.251	−7.379	−7.230		−7.308	*−7.254*	−7.457	*−7.114*		*−7.433*	−6.995	−7.209
**LogD**	4.781	5.494		5.642	5.744	5.572		5.638	*5.866*	5.916	*6.085*		*5.866*	5.919	5.816
**LogP**	6.370	7.594		7.832	8.148	8.102		7.493	*7.981*	8.554	*8.440*		*8.389*	8.104	8.542
**SAscore**	4.589	4.746		4.753	4.797	4.870		4.933	*4.845*	4.976	*4.838*		*4.960*	5.109	4.932
**Fsp3**	0.900	0.778		0.784	0.789	0.737		0.737	*0.789*	0.795	*0.795*		*0.795*	0.789	0.700
**Npscore**	3.272	2.518		2.373	2.344	2.376		2.217	*2.259*	2.328	*2.334*		*2.233*	2.366	2.193
**Caco-2 Perm.**	−5.216	−4.713		−4971	−4.975	−4.934		−4.974	*−4.960*	−4.983	*−5.026*		*−4.937*	−5.033	−4.992
**MDCK perm.**	1.2 × 10^−5^	1.7 × 10^−5^		1.6 × 10^−5^	1.5 × 10^−5^	1.9 × 10^−5^		1.6 × 10^−5^	*1.5* *× 10^−5^*	1.4 × 10^−5^	*1.4* *× 10^−5^*		*1.4* *× 10^−5^*	1.5 × 10^−5^	1.6 × 10^−5^
**F_30%_**	0.874	0.763		0.623	0.780	0.138		0.710	*0.505*	0.843	*0.656*		*0.631*	0.422	0.630
**BBB penetr.**	0.577	0.024		0.018	0.020	0.012		0.049	*0.020*	0.020	*0.016*		*0.024*	0.036	0.003
**PPB**	96.976	95.951		96.585	96.497	98.014		96.852	*97.305*	96.539	*96.489*		*96.725*	99.819	96.890
**VDss**	0.707	1.004		1.904	1.954	1.338		1.791	*2.013*	2.062	*1.991*		*2.023*	2.096	2.667
**CYP2C19 Sub.**	0.901	0.937		0.916	0.911	0.895		0.908	*0.888*	0.901	*0.915*		*0.916*	0.915	0.724
**CYP2C9 Inh.**	0.179	0.207		0.201	0.159	0.172		0.463	*0.138*	0.139	*0.118*		*0.129*	0.152	0.128
**CYP2C9 Sub.**	0.758	0.492		0.407	0.480	0.564		0.444	*0.328*	0.491	*0.387*		*0.368*	0.539	0.323
**CYP3A4 Sub.**	0.227	0.736		0.733	0.690	0.759		0.846	*0.715*	0.682	*0.723*		*0.765*	0.786	0.719
**CL**	3.813	9.937		9.760	9.953	10.116		7.749	*10.093*	9.455	*9.838*		*9.332*	8.969	9.175
**T1/2 (<3h)**	0.021	0.004		0.002	0.002	0.002		0.002	*0.003*	0.002	*0.002*		*0.002*	0.003	0.003
**Skin Sensit.**	0.046	0.034		0.046	0.054	0.079		0.072	*0.041*	0.068	*0.059*		*0.041*	0.041	0.024
**Carcinogen.**	0.095	0.104		0.117	0.109	0.285		0.284	*0.141*	0.102	*0.124*		*0.133*	0.228	0.160
**Eye Corrosion**	0.030	0.004		0.003	0.003	0.003		0.003	*0.004*	0.003	*0.003*		*0.004*	0.003	0.003
**Eye Irritation**	0.066	0.031		0.093	0.104	0.158		0.091	*0.031*	0.099	*0.159*		*0.026*	0.071	0.145
**BCF**	1.815	2.222		2.214	1.649	2.289		1.758	*2.313*	1.521	*1.930*		*2.085*	1.995	1.545
**IGC_50_**	4.971	5.420		5.495	5.563	5.480		5.468	*5.460*	5.668	*5.614*		*5.494*	5.616	5.649
**LC_50_DM**	6.345	6.797		6.810	6.802	6.813		6.910	*6.709*	6.806	*6.764*		*6.822*	6.852	6.920
**LC_50_FM**	5.884	6.459		6.569	6.584	6.930		6.883	*6.390*	6.648	*6.654*		*6.453*	6.604	6.642
**NR-AhR**	0.001	0.001		0.001	0.001	0.001		0.001	*0.001*	0.001	*0.000*		*0.000*	0.001	0.001
**NR-AR**	0.369	0.516		0.037	0.044	0.088		0.033	*0.034*	0.036	*0.022*		*0.215*	0.030	0.026
**NR-AR-LBD**	0.479	0.862		0.830	0.857	0.901		0.899	*0.695*	0.838	*0.581*		*0.861*	0.854	0.820
**NR-Aromatase**	0.798	0.541		0.550	0.515	0.475		0.548	*0.523*	0.496	*0.474*		*0.501*	0.588	0.466
NB Rule *	0	0		0	0	0		0	*0*	0	*0*		*0*	0	0
NGC Rule *	0	0		0	0	0		0	*0*	0	*0*		*0*	1	0
ChEMBL Rule *	0	0		0	0	0		1	*0*	0	*0*		*0*	0	0

**Table 6:** Cytotoxic Activity (IC_50_) of oleanolic acid (**1**) and its derivatives (**2a**–**2k** and **3a**–**3k**). **Legend**: IC_50_ = half-maximal inhibitory concentration; **SD** = standard deviation; **^a^** = published earlier [61]; **^b^** = published earlier [46]; **^c^** = published earlier [62]; **values written in *italics*** = the alcohol part of the oleanolic acid ester is branched; **values underlined** = the alcohol part of the ester contains a multiple bond. Data are presented as mean IC_50_ values with SD from 3 independent biological experiments, each performed with 3 technical replicates.

≤ 2.00 μM		2.01–5.00 μM			6.01–10.0 μM		10.01–15.0 μM			≥15.01 μM	
**Comp. No.**	**IC_50_ (SD), μM**										
	HeLa		MCF-7	A-549		SKBR-3		PC-3	SKOV-3		HDF
**1**	11.82 (±0.04) ^a,b^		13.95 (±0.09) ^a,b^	8.79 (±0.28) ^b^		19.62 (±0.02) ^c^		18.63 (±0.05) ^c^	18.81 (±0.09) ^c^		24.87 (±0.04) ^b^
**2a**	7.69 (±0.04)		7.71 (±0.02)	7.71 (±0.01)		7.19 (±0.05)		7.38 (±0.03)	7.41 (±0.01)		9.29 (±0.02)
**2b**	8.09 (±0.01)		8.44 (±0.07)	8.41 (±0.03)		7.12 (±0.02)		8.03 (±0.07)	8.02 (±0.01)		14.88 (±0.17)
**2c**	1.15 (±0.03)		1.07 (±0.01)	1.05 (±0.01)		1.31 (±0.06)		1.09 (±0.02)	1.03 (±0.04)		1.98 (±0.02)
**2d**	9.44 (±0.03)		9.71 (±0.01)	9.88 (±0.06)		9.45 (±0.02)		9.30 (±0.02)	9.26 (±0.01)		17.33 (±0.48)
**2e**	8.31 (±0.05)		8.34 (±0.06)	8.17 (±0.01)		8.21 (±0.03)		8.59 (±0.12)	8.31 (±0.01)		13.06 (±0.11)
***2f***	*8.22 (±0.04)*		*8.19 (±0.02)*	*8.06 (±0.05)*		*8.28 (±0.03)*		*8.11 (±0.01)*	*8.26 (±0.02)*		*15.09 (±0.03)*
**2g**	7.19 (±0.05)		7.01 (±0.01)	7.11 (±0.01)		7.26 (±0.02)		7.05 (±0.01)	7.22 (±0.04)		11.31 (±0.09)
***2h***	*10.08 (±0.07)*		*9.76 (±0.12)*	*9.49 (±0.04)*		*9.46 (±0.03)*		*9.29 (±0.02)*	*9.16 (±0.05)*		*16.44 (±0.08)*
***2i***	*1.07 (±0.01)*		*1.18 (±0.02)*	*1.03 (±0.01)*		*1.02 (±0.02)*		*1.14 (±0.07)*	*1.03 (±0.01)*		*2.99 (±0.02)*
**2j**	1.37 (±0.01)		1.32 (±0.02)	1.51 (±0.01)		1.34 (±0.03)		1.32 (±0.03)	1.37 (±0.04)		3.19 (±0.01)
**2k**	8.99 (±0.03)		8.27 (±0.11)	8.49 (±0.02)		8.41 (±0.05)		8.12 (±0.02)	8.44 (±0.03)		13.04 (±0.03)
**3a**	2.59 (±0.04)		2.52 (±0.01)	2.58 (±0.04)		2.59 (±0.01)		2.81 (±0.01)	2.22 (±0.05)		3.86 (±0.04)
**3b**	2.20 (±0.01)		2.26 (±0.02)	2.23 (±0.01)		2.16 (±0.06)		2.29 (±0.02)	2.04 (±0.01)		4.80 (±0.03)
**3c**	1.88 (±0.03)		1.72 (±0.01)	1.77 (±0.01)		1.82 (±0.04)		1.73 (±0.01)	1.74 (±0.03)		2.19 (±0.05)
**3d**	2.53 (±0.03)		2.59 (±0.01)	2.04 (±0.03)		2.58 (±0.07)		2.56 (±0.01)	2.52 (±0.04)		4.91 (±0.04)
**3e**	3.56 (±0.01)		3.52 (±0.03)	3.57 (±0.03)		3.61 (±0.01)		3.28 (±0.07)	3.51 (±0.02)		6.38 (±0.03)
***3f***	*1.47 (±0.03)*		*1.28 (±0.01)*	*1.47 (±0.01)*		*1.88 (±0.06)*		*1.53 (±0.02)*	*1.16 (±0.05)*		*3.77 (±0.09)*
**3g**	2.07 (±0.01)		2.11 (±0.06)	2.05 (±0.01)		2.36 (±0.03)		2.39 (±0.03)	2.04 (±0.02)		3.19 (±0.06)
***3h***	*1.68 (±0.01)*		*1.74 (±0.06)*	*1.81 (±0.03)*		*1.12 (±0.01)*		*1.66 (±0.04)*	*1.62 (±0.01)*		*2.84 (±0.06)*
***3i***	*1.13 (±0.01)*		*1.07 (±0.05)*	*1.12 (±0.01)*		*1.09 (±0.01)*		*1.18 (±0.04)*	*1.17 (±0.01)*		*2.31 (±0.05)*
**3j**	1.28 (±0.02)		1.27 (±0.01)	1.30 (±0.03)		1.25 (±0.09)		1.28 (±0.01)	1.34 (±0.03)		2.88 (±0.02)
**3k**	2.12 (±0.01)		2.04 (±0.01)	2.02 (±0.03)		2.01 (±0.01)		2.09 (±0.05)	2.42 (±0.02)		4.19 (±0.03)

**Table 7:** Selectivity Index (SI) of oleanolic acid (**1**) and its derivatives (**2a**–**2k** and **3a**–**3k**) determined in the MTT assay. **Legend**: **^a^** = published earlier [61]; **^b^** = published earlier [46]; **^c^** = published earlier [62]; **values written in *italics*** = the alcohol part of the oleanolic acid ester is branched; **values underlined** = the alcohol part of the ester contains a multiple bond. SI values were calculated as IC_50_ (HDF)/IC_50_ (cancer cell line).

≥ 2.5		2.49—2.00			1.99—1.50			≤1.49	
**Comp. No.**	**SI**								
	**HeLa**		**MCF-7**	**A-549**		**SKBR-3**	**PC-3**		**SKOV-3**
**1**	2.10 ^a,b^		1.78 ^a,b^	2.83 ^b^		1.27 ^c^	1.33 ^c^		1.32 ^c^
**2a**	1.21		1.20	1.20		1.29	1.27		1.25
**2b**	1.84		1.76	1.77		2.09	1.85		1.85
**2c**	1.72		1.85	1.88		1.51	1.82		1.92
**2d**	1.83		1.78	1.75		1.83	1.86		1.55
**2e**	1.57		1.56	1.60		1.59	1.52		1.57
***2f***	*1.83*		*1.84*	*1.87*		*1.82*	*1.86*		*1.82*
**2g**	1.57		1.61	1.59		1.56	1.60		1.57
***2h***	*1.63*		*1.68*	*1.73*		*1.74*	*1.77*		*1.79*
***2i***	*2.79*		*2.53*	*2.90*		*2.93*	*2.62*		*2.90*
**2j**	2.33		2.41	2.11		2.38	2.41		2.33
**2k**	1.45		1.58	1.53		1.55	1.61		1.54
**3a**	1.49		1.53	1.50		1.49	1.37		1.74
**3b**	2.18		2.12	2.15		2.22	2.10		2.35
**3c**	1.16		1.27	1.23		1.20	1.26		1.26
**3d**	1.94		1.89	2.40		1.90	1.92		1.95
**3e**	1.79		1.81	1.79		1.77	1.94		1.82
***3f***	*2.56*		*2.94*	*2.56*		*2.01*	*2.46*		*3.25*
**3g**	1.54		1.51	1.56		1.35	1.33		1.56
***3h***	*1.69*		*1.63*	*1.57*		*2.54*	*1.71*		*1.75*
***3i***	*2.04*		*2.16*	*2.06*		*2.12*	*1.95*		*1.97*
**3j**	2.25		2.27	2.21		2.30	2.25		2.15
**3k**	1.98		2.05	2.07		2.08	2.00		1.73

**Table 8:** Largest pocket of 1M17, results from the CB-Dock2 web server.

CurPocket ID	Cavity Volume (Å^3^)	Center (x, y, z)	Cavity Size (x, y, z)
C1	991	36, 8, 51	14, 17, 13

**Table 9:** Data compiled from CB-Dock2 outputs for pocket C1 (volume 991 Å^3^; center 36, 8, 51).

Comp. No.	Vina Score (kcal × mol^−1^)	Cavity Volume (Å^3^)	Center (x, y, z)	Docking Box Size (x, y, z)
**2c**	−7.5	991	36, 8, 51	23, 23, 23
**2i**	−8.5	991	36, 8, 51	23, 23, 23
**2j**	−7.5	991	36, 8, 51	24, 24, 24
**3c**	−8.3	991	36, 8, 51	24, 24, 24
**3f**	−7.8	991	36, 8, 51	25, 25, 25
**3h**	−7.3	991	36, 8, 51	23, 23, 23
**3i**	−7.4	991	36, 8, 51	25, 25, 25
**3j**	−7.2	991	36, 8, 51	26, 26, 26

**Table 10:** Summary of selected lead compounds ranked according to cytotoxic potency, selectivity and predicted developability. **Legend**: physicochemical values are based on calculated or predicted descriptors. **MW** = molecular weight; **TPSA** = topological polar surface area; **HBD** = hydrogen-bond donors; **HBA** = hydrogen-bond acceptors; **SI** = Selectivity Index.

Comp. No.	MW	TPSA	LogP	HBD	HBA	Main Advantage	Main Limitation	Overall Prioritization
**2i**	512.42	46.53	7.52	1	3	Strong cytotoxicity and favorable SI profile	High lipophilicity and poor predicted solubility	Balanced lead
**2j**	566.33	46.53	7.24	1	3	Strong cytotoxicity, SI > 2 in several cell lines and retained CUPRAC activity	Halogenated substituent and possible metabolic liabilities	Balanced lead requiring safety follow-up
**3f**	592.41	65.74	7.98	0	5	High cytotoxicity and the most favorable SI trend among furoyl derivatives	High lipophilicity and poor predicted solubility	Selectivity-focused lead
**3i**	606.43	65.74	8.39	0	5	Very strong cytotoxic potency	Lower developability due to high lipophilicity	Potency-focused candidate
**3j**	660.33	65.74	8.10	0	5	Strong cytotoxicity and favorable SI values	Highest molecular weight and potential metabolic liabilities	Balanced but optimization-needed lead

## Data Availability

All data from this study are available in the manuscript body or in the Supplementary Materials.

## Supplementary Materials

The following supporting information can be downloaded at: https://www.mdpi.com/article/10.3390/pharmaceutics18070832/s1, Figure S1: ^1^H NMR and ^13^C NMR spectra of compounds **2a**–**2k**; Figure S2: ^1^H NMR and ^13^C NMR spectra of compounds **3a**–**3k**; Table S1. Predicted activity of oleanolic acid (**1**) and its derivatives **2a**–**2k** determined by the PASS method. Table S2. Predicted activity of oleanolic acid (**1**) and its derivatives **2a**–**2k** determined by the PASS method. Table S3. ADMETox data for oleanolic acid (**1**) and its derivatives **2a**–**2k**. Table S4 ADMETox data for oleanolic acid (**1**) and its derivatives **3a**–**3k**. Figure S3. Standard curve for (**A**) CUPRAC and (**B**) DPPH assay as Trolox equivalent. Figure S4. A. Whole 1M17 molecule, the dark pink color indicates the cavity of the molecule. B. View of the largest pocket of C1 with a volume of 991 Å3 indicated by a black arrow. Figures S5–S12: 3D surface of structure 1M17 with ligand compounds **2c** (Figure S5), **2i** (Figure S6), **2j** (Figure S7), **3c** (Figure S8), **3f** (Figure S9), **3h** (Figure S10), **3i** (Figure S11), **3j** (Figure S12) with H-bond gradient map in C1 pocket and 2D diagram with interactions. Table S5. Data compiled from CB-Dock2 outputs for pocket C1 (volume 991 Å^3^; center 36, 8, 51).
