# Supplementary material for: Short-Chain Oleanolic Acid Esters and Furoyl Hybrids: Pharmacological Prediction, ADMETox Profiling, In Vitro Cytotoxicity Evaluation, Antioxidant Testing and EGFR Docking
Source: Pharmaceutics. 2026 Jul 7;18(7):832. doi: 10.3390/pharmaceutics18070832 (PMC13414634; doi:10.3390/pharmaceutics18070832)
Supplement: Supplementary file 1 [file pharmaceutics-18-00832-s001.zip › pharmaceutics-4382875-supplementary.pdf]

Article

Short-Chain Oleanolic Acid Esters and Furoyl Hybrids: Pharmacological Prediction, ADMETox Profiling, In Vitro Cytotoxicity Evaluation, Antioxidant Testing and EGFR Docking

Barbara Bednarczyk-Cwynar[1,2,*], Piotr Ruszkowski[3], Maciej Kulawik[4,5], Szymon Sip[4], Przemysław Zalewski[4], Dobrosława Wiśniewska[1], Andrzej Günther[1]

[1] Department of Organic Chemistry, Faculty of Pharmacy, Poznan University of Medical Sciences, Collegium Pharmaceuticum 2, Rokietnicka Str. 3, 60–806 Poznan, Poland; 89387@student.ump.edu.pl (D.W.); andrzej.gunther@me.pl (A.G.)
[2] Center of Innovative Pharmaceutical Technology, Rokietnicka Str. 3, 60–806 Poznan, Poland
[3] Department of Pharmacology, Faculty of Pharmacy, Poznan University of Medical Sciences, Collegium Pharmaceuticum 1, Rokietnicka Str. 3, 60–806 Poznan, Poland; pruszkowski@gmail.com
[4] Department of Pharmacognosy and Biomaterials, Faculty of Pharmacy, Poznan University of Medical Sciences, Collegium Pharmaceuticum 1, Rokietnicka Str. 3, 60–806 Poznan, Poland; maciej.kulawik@student.ump.edu.pl (M.K.); szymonsip@ump.edu.pl (S.S.); pzalewski@ump.edu.pl (P.Z.)
[5] Doctoral School, Poznan University of Medical Sciences, Bukowska Str. 70, 60-812 Poznan, Poland
* Correspondence: bcwynar@ump.edu.pl

Supplementary Materials

Figure S1. ^{1}H NMR and ^{13}C NMR spectra of compounds **2a** – **2k**

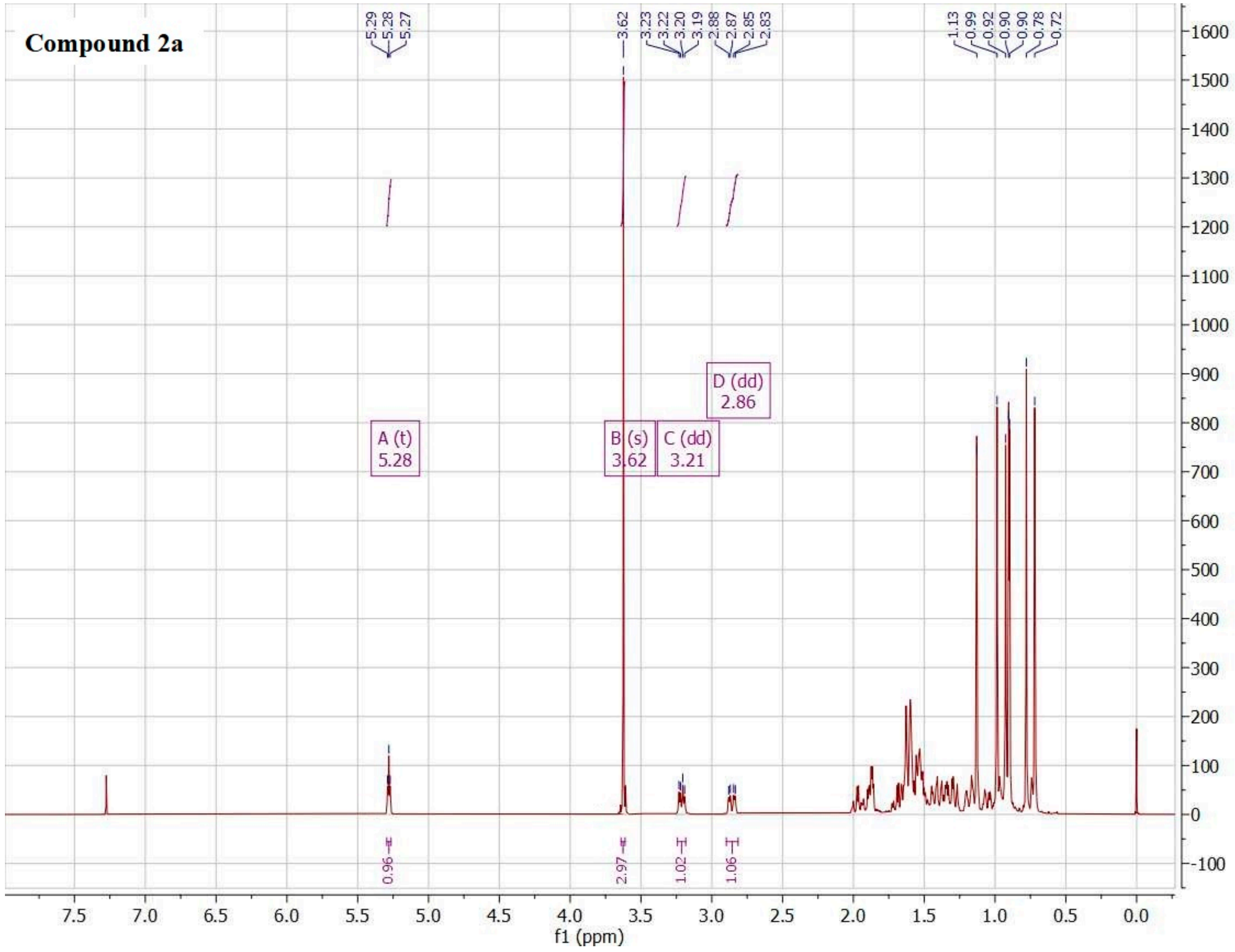
Compound 2a
5.29
5.28
5.27
3.62
3.23
3.22
3.20
3.19
2.88
2.87
2.85
2.83
1.13
0.99
0.92
0.90
0.90
0.78
0.72
A (t)
5.28
B (s)
3.62
C (dd)
3.21
D (dd)
2.86
0.96
2.97
1.02
1.06
f1 (ppm)

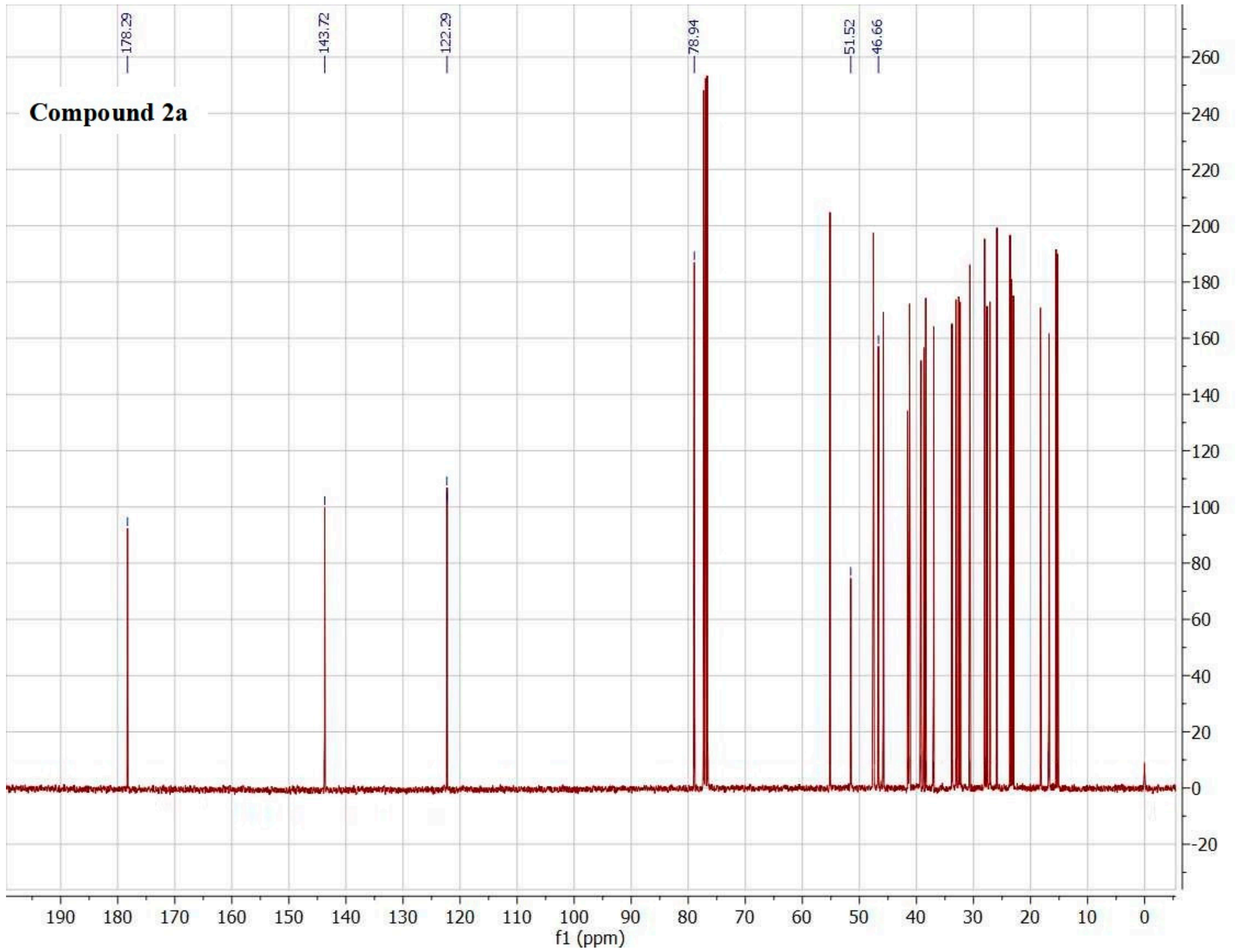

Compound 2a
178.29
143.72
122.29
78.94
51.52
46.66
f1 (ppm)

Compound 2b

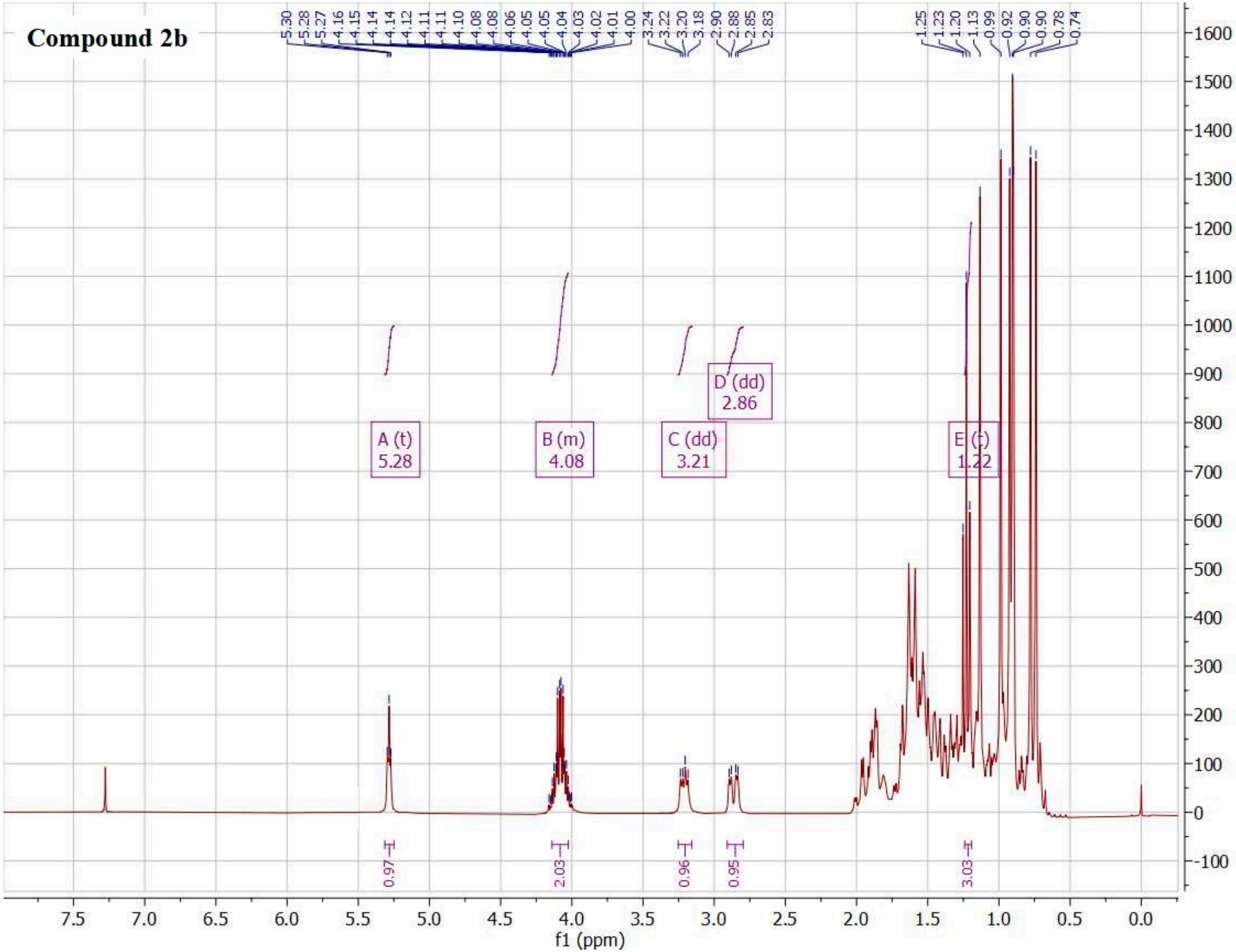
5.30 5.28 5.27 4.16 4.15 4.14 4.14 4.12 4.11 4.11 4.10 4.08 4.08 4.06 4.05 4.05 4.04 4.03 4.02 4.01 4.00 3.24 3.22 3.20 3.18 2.90 2.88 2.85 2.83
1.25 1.23 1.20 1.13 0.99 0.92 0.90 0.90 0.78 0.74
A (t) 5.28
B (m) 4.08
C (dd) 3.21
D (dd) 2.86
E (t) 1.22
0.97
2.03
0.96
0.95
3.03
f1 (ppm)

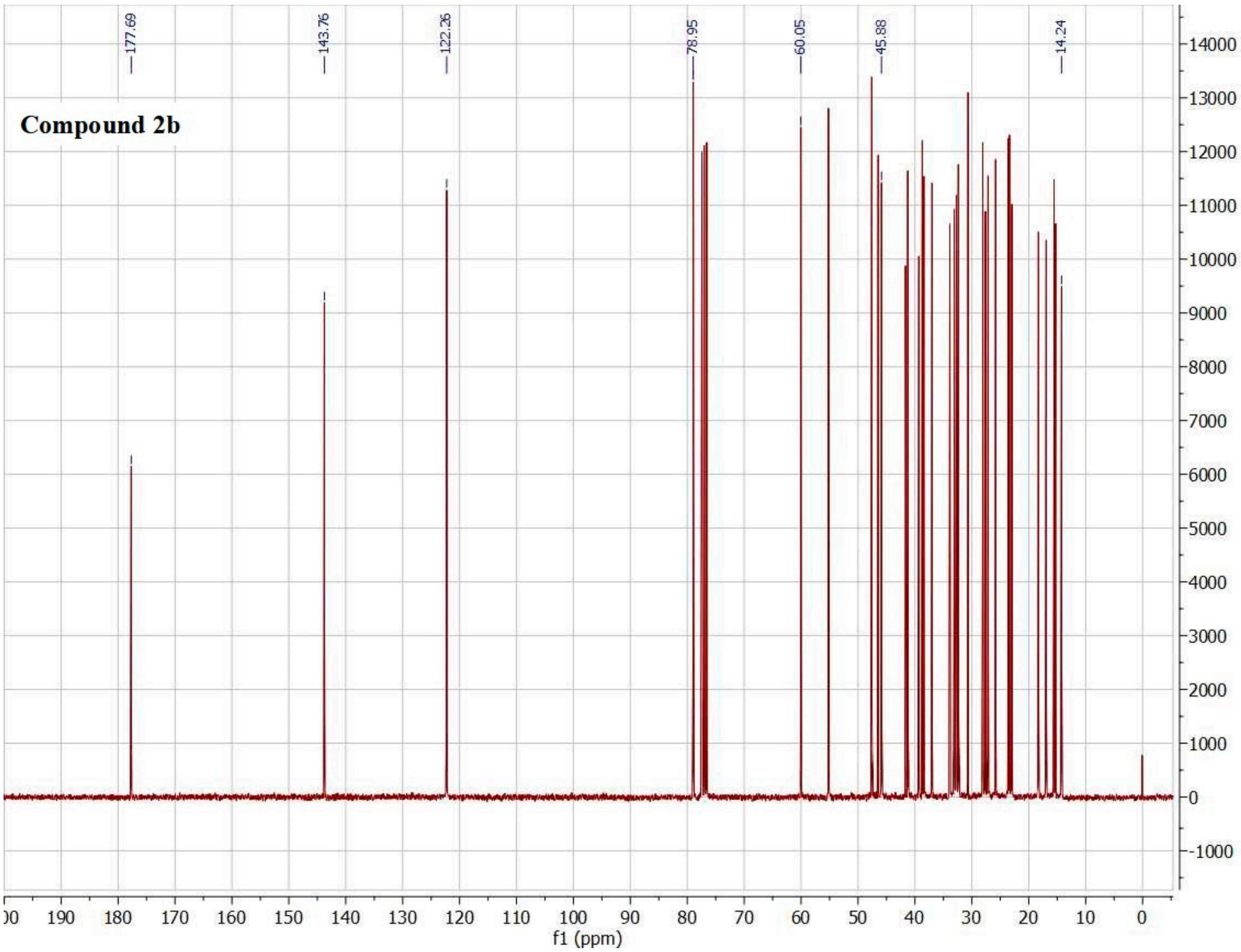

177.69
143.76
122.26
78.95
60.05
45.88
14.24
Compound 2b
f1 (ppm)

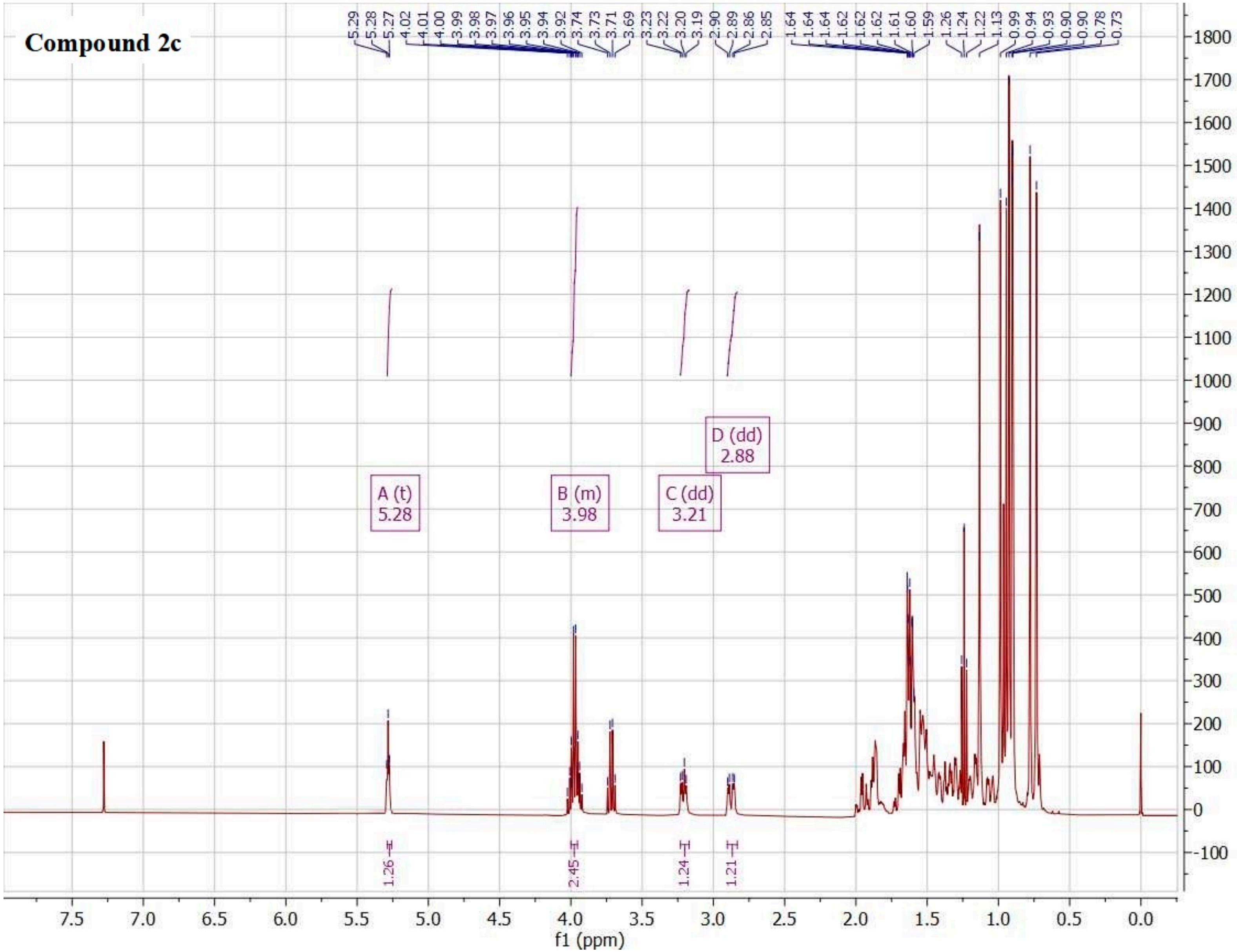

Compound 2c
5.29
5.28
5.27
4.02
4.01
4.00
3.99
3.98
3.97
3.96
3.95
3.94
3.92
3.74
3.73
3.71
3.69
3.23
3.22
3.20
3.19
2.90
2.89
2.86
2.85
1.64
1.64
1.64
1.62
1.62
1.62
1.61
1.60
1.59
1.26
1.24
1.22
1.13
0.99
0.94
0.93
0.90
0.90
0.78
0.73
A (t)
5.28
B (m)
3.98
C (dd)
3.21
D (dd)
2.88
1.26
2.45
1.24
1.21
f1 (ppm)

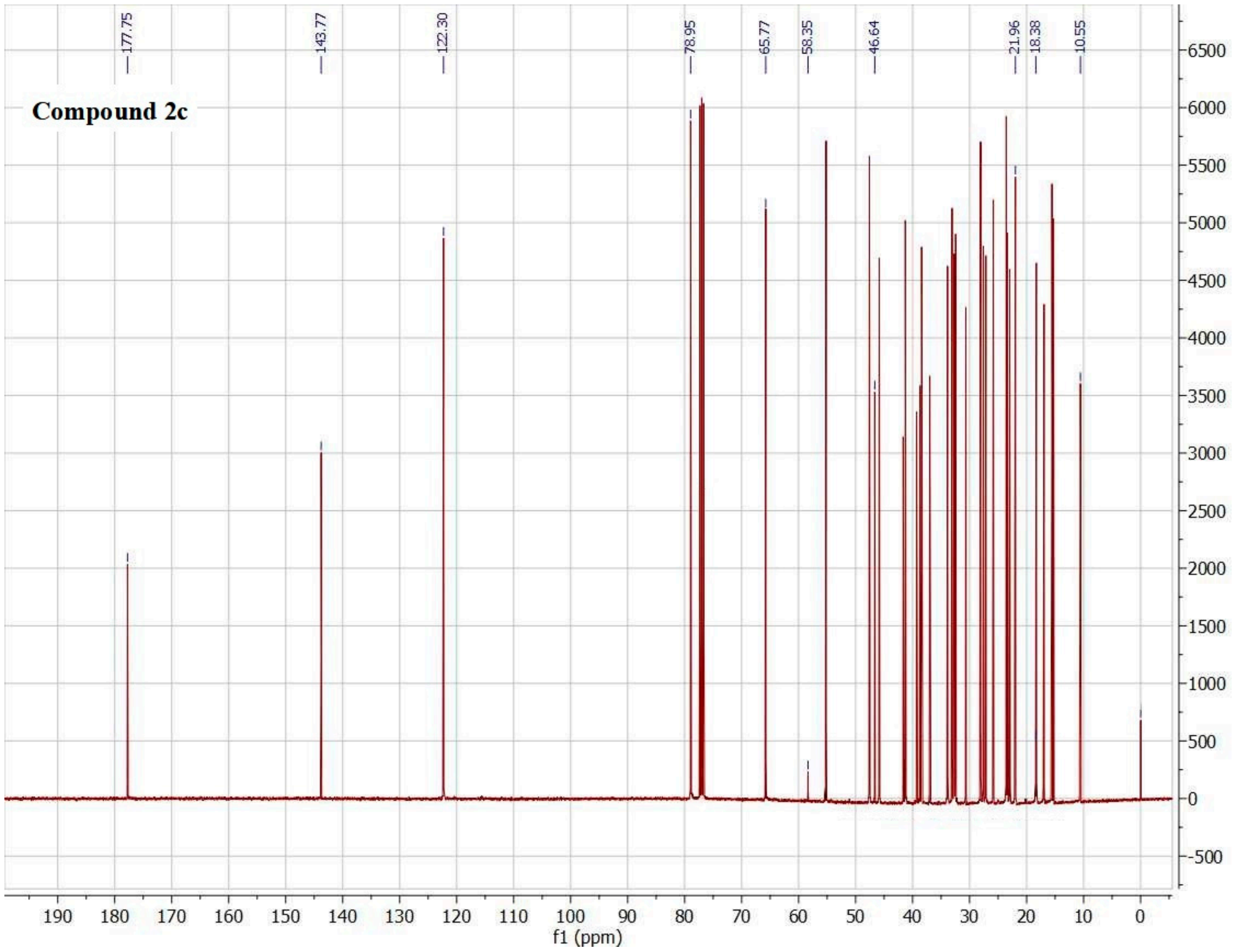

Compound 2c
177.75
143.77
122.30
78.95
65.77
58.35
46.64
21.96
18.38
10.55
f1 (ppm)

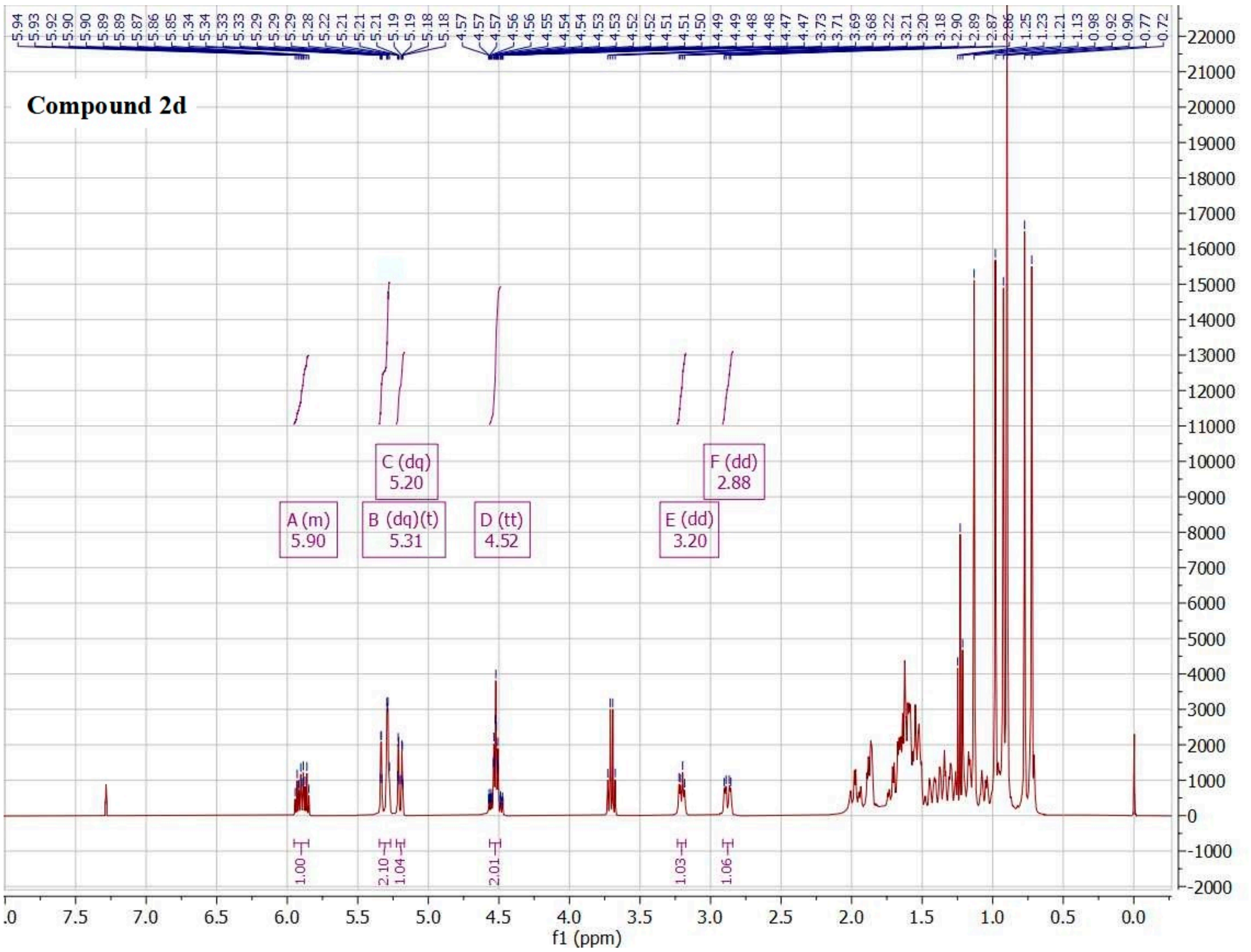

Compound 2d
A (m)
5.90
B (dq)(t)
5.31
C (dq)
5.20
D (tt)
4.52
E (dd)
3.20
F (dd)
2.88
1.00
2.10
1.04
2.01
1.03
1.06
f1 (ppm)

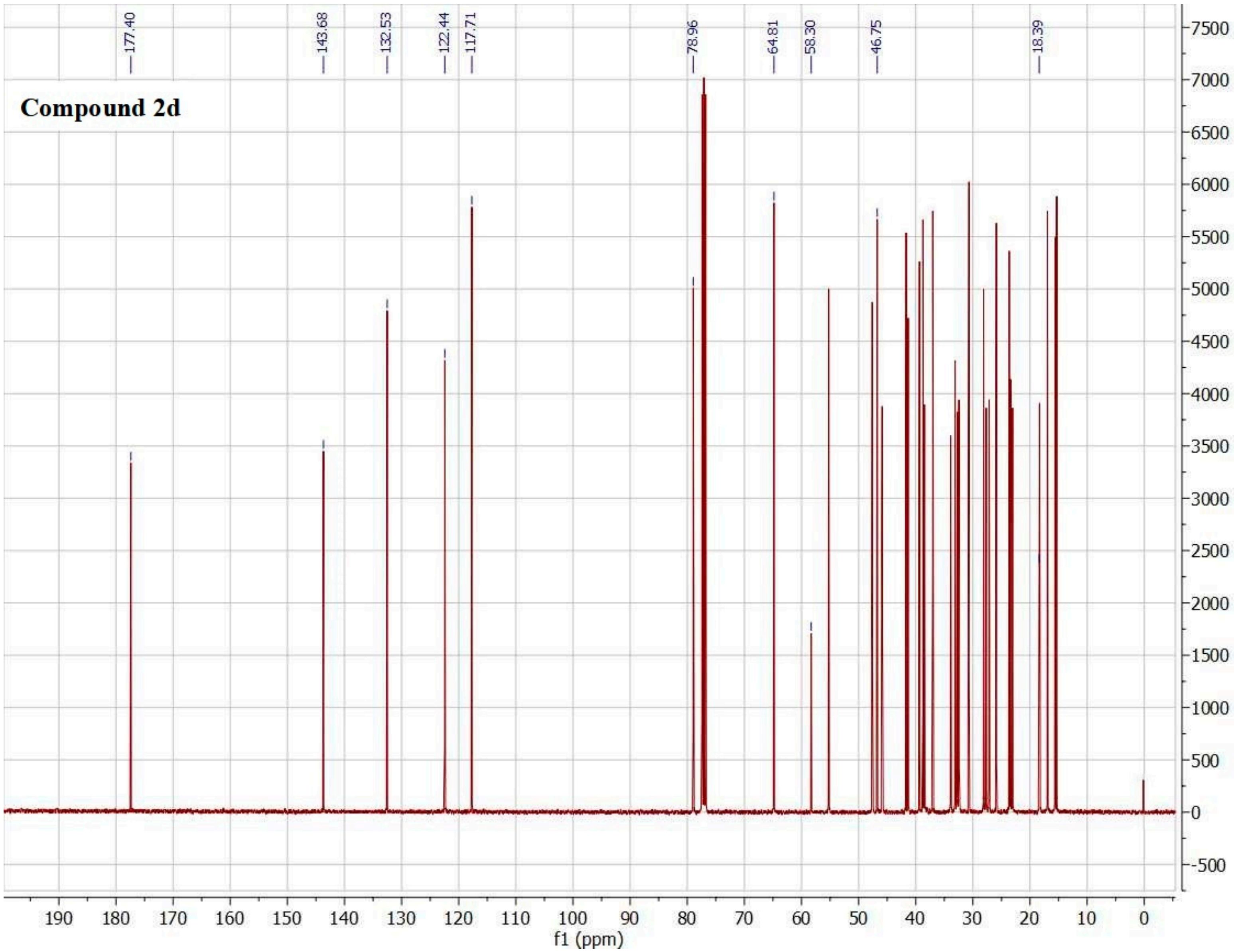
Compound 2d
177.40
143.68
132.53
122.44
117.71
78.96
64.81
58.30
46.75
18.39
f1 (ppm)

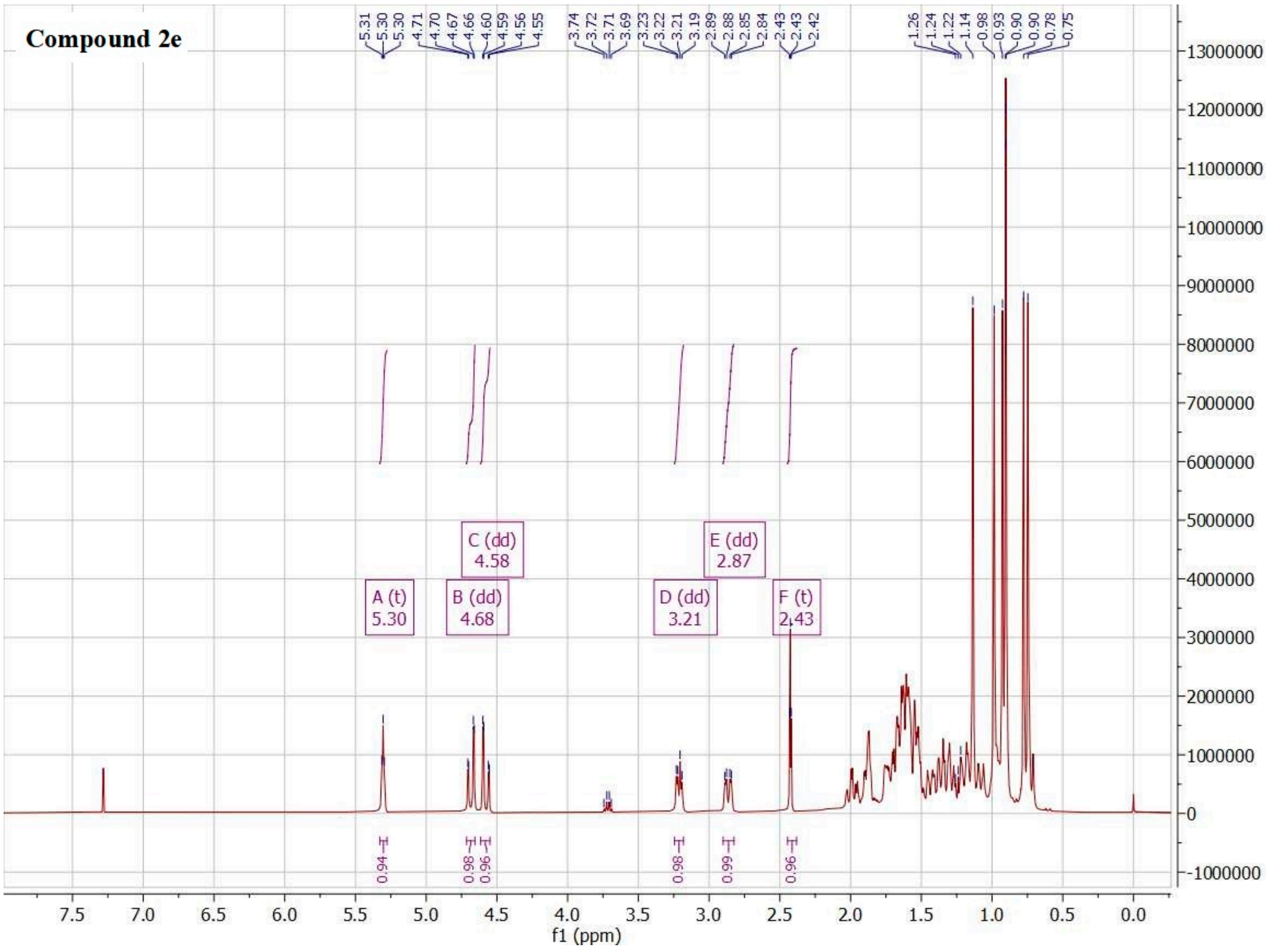

Compound 2e
5.31
5.30
5.30
4.71
4.70
4.67
4.66
4.60
4.59
4.56
4.55
3.74
3.72
3.71
3.69
3.23
3.22
3.21
3.19
2.89
2.88
2.85
2.84
2.43
2.43
2.42
1.26
1.24
1.22
1.14
0.98
0.93
0.90
0.90
0.78
0.75
A (t)
5.30
B (dd)
4.68
C (dd)
4.58
D (dd)
3.21
E (dd)
2.87
F (t)
2.43
0.94
0.98
0.96
0.98
0.99
0.96
f1 (ppm)

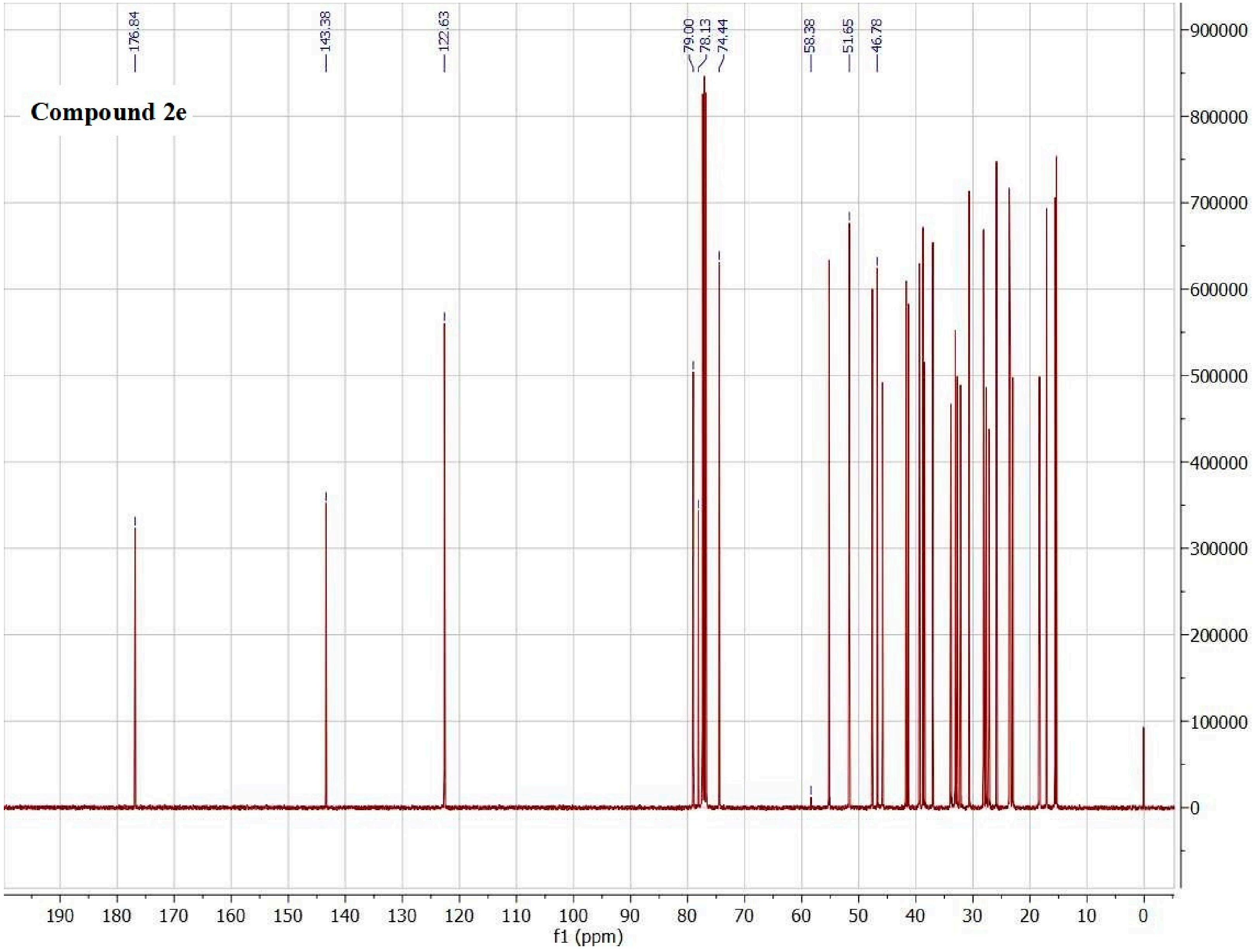

Compound 2e
176.84
143.38
122.63
79.00
78.13
74.44
58.38
51.65
46.78
f1 (ppm)

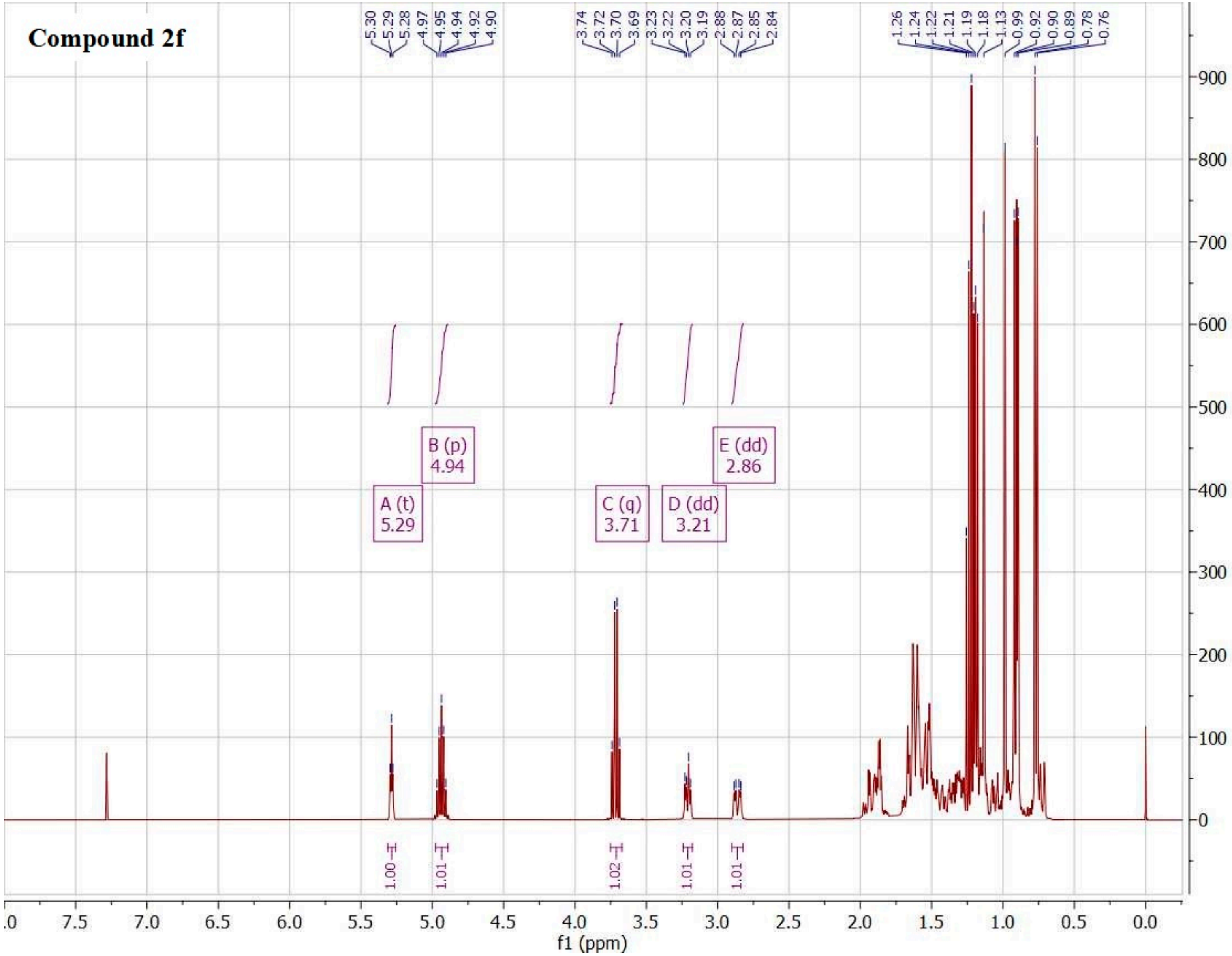
Compound 2f
5.30
5.29
5.28
4.97
4.95
4.94
4.92
4.90
3.74
3.72
3.70
3.69
3.23
3.22
3.20
3.19
2.88
2.87
2.85
2.84
1.26
1.24
1.22
1.21
1.19
1.18
1.13
0.99
0.92
0.90
0.89
0.78
0.76
A (t) 5.29
B (p) 4.94
C (q) 3.71
D (dd) 3.21
E (dd) 2.86
1.00
1.01
1.02
1.01
1.01
f1 (ppm)

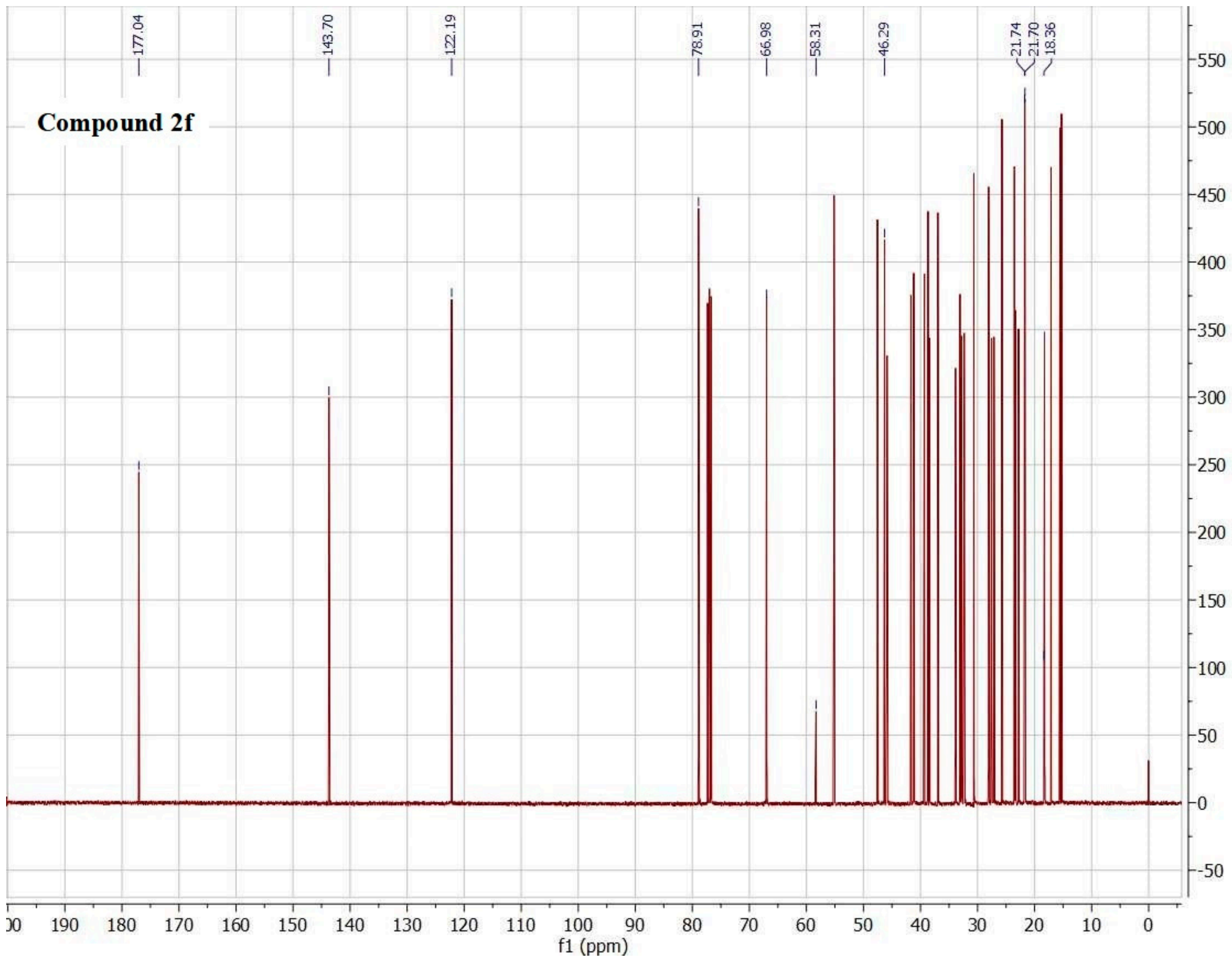

Compound 2f
177.04
143.70
122.19
78.91
66.98
58.31
46.29
21.74
21.70
18.36
f1 (ppm)

Compound 2g

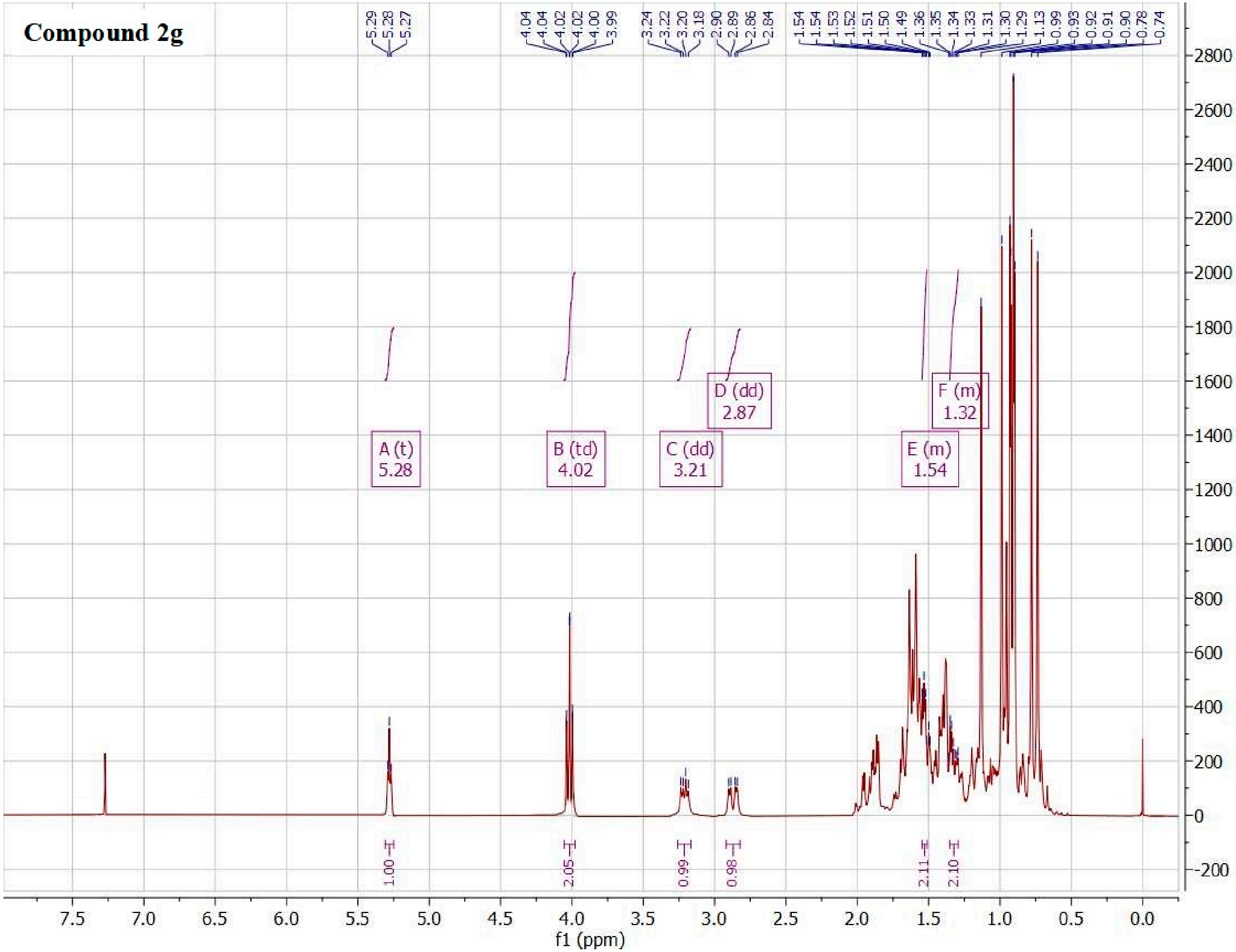
5.29
5.28
5.27
4.04
4.04
4.02
4.02
4.00
3.99
3.24
3.22
3.20
3.18
2.90
2.89
2.86
2.84
1.54
1.54
1.53
1.52
1.51
1.50
1.49
1.36
1.35
1.34
1.33
1.31
1.30
1.29
1.13
0.99
0.93
0.92
0.91
0.90
0.78
0.74
A (t)
5.28
B (td)
4.02
C (dd)
3.21
D (dd)
2.87
E (m)
1.54
F (m)
1.32
1.00
2.05
0.99
0.98
2.11
2.10
f1 (ppm)

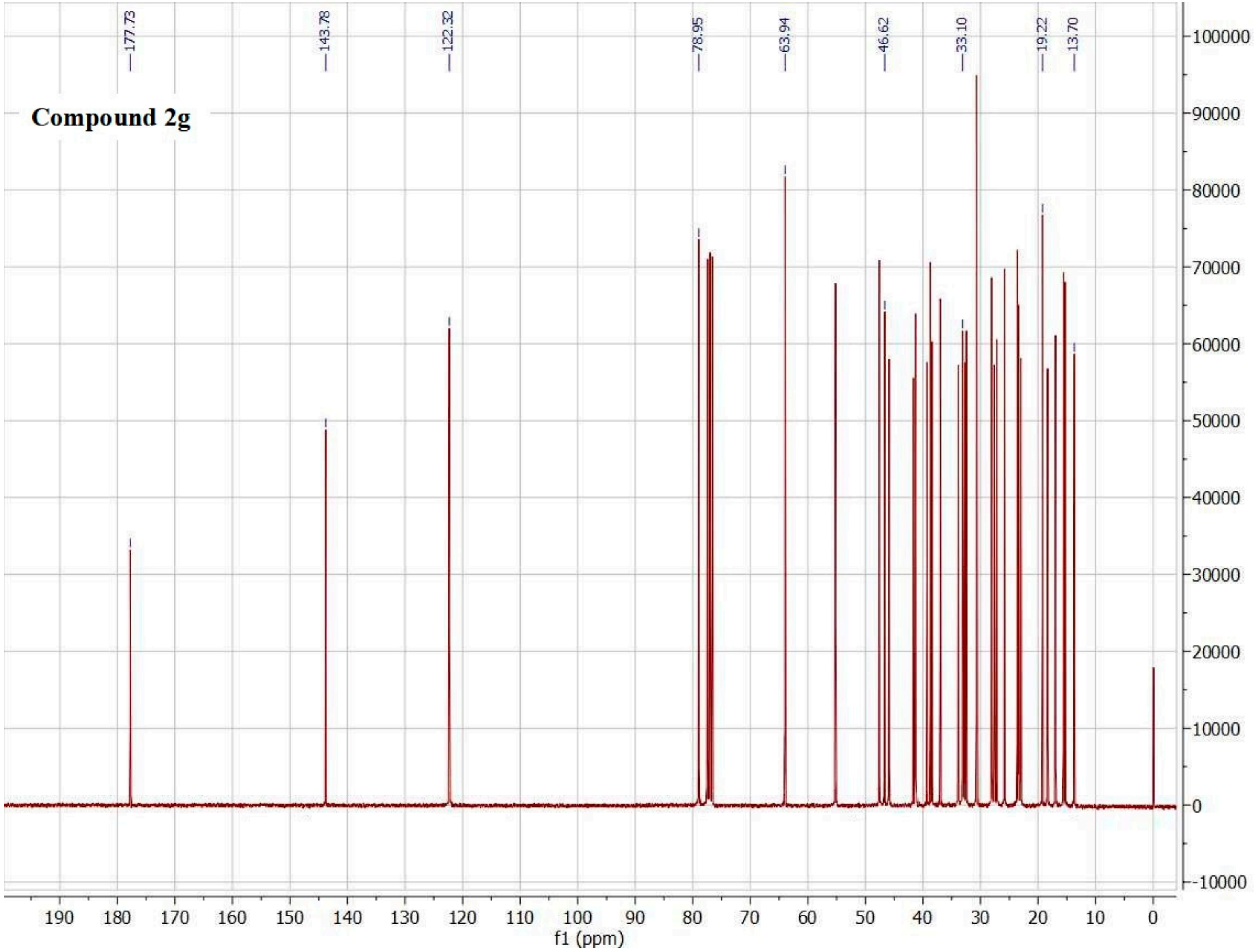
Compound 2g
177.73
143.78
122.32
78.95
63.94
46.62
33.10
19.22
13.70
100000
90000
80000
70000
60000
50000
40000
30000
20000
10000
0
-10000
190
180
170
160
150
140
130
120
110
100
90
80
70
60
50
40
30
20
10
0
f1 (ppm)

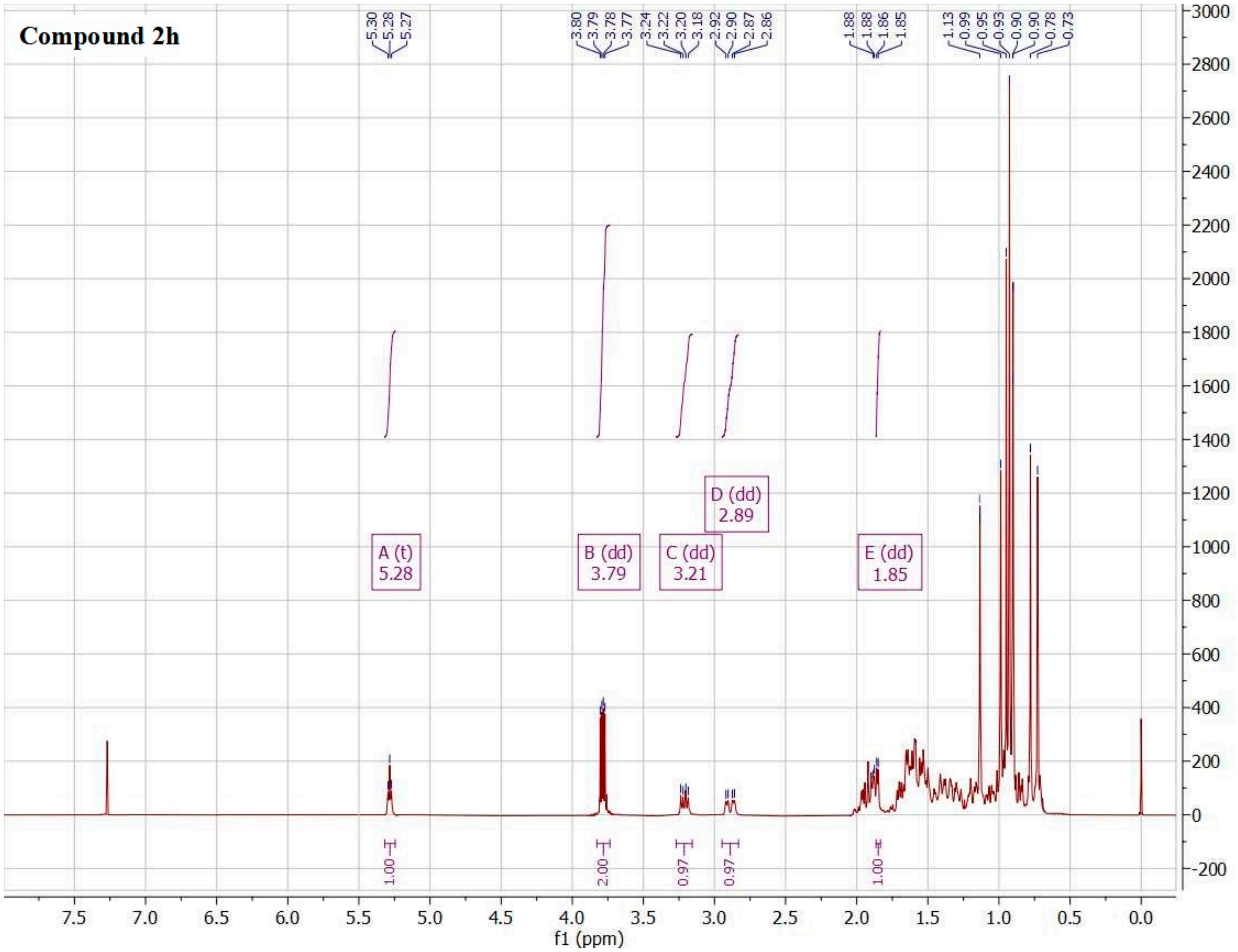
Compound 2h
5.30
5.28
5.27
3.80
3.79
3.78
3.77
3.24
3.22
3.20
3.18
2.92
2.90
2.87
2.86
1.88
1.88
1.86
1.85
1.13
0.99
0.95
0.93
0.90
0.78
0.73
A (t) 5.28
B (dd) 3.79
C (dd) 3.21
D (dd) 2.89
E (dd) 1.85
1.00
2.00
0.97
0.97
1.00
f1 (ppm)

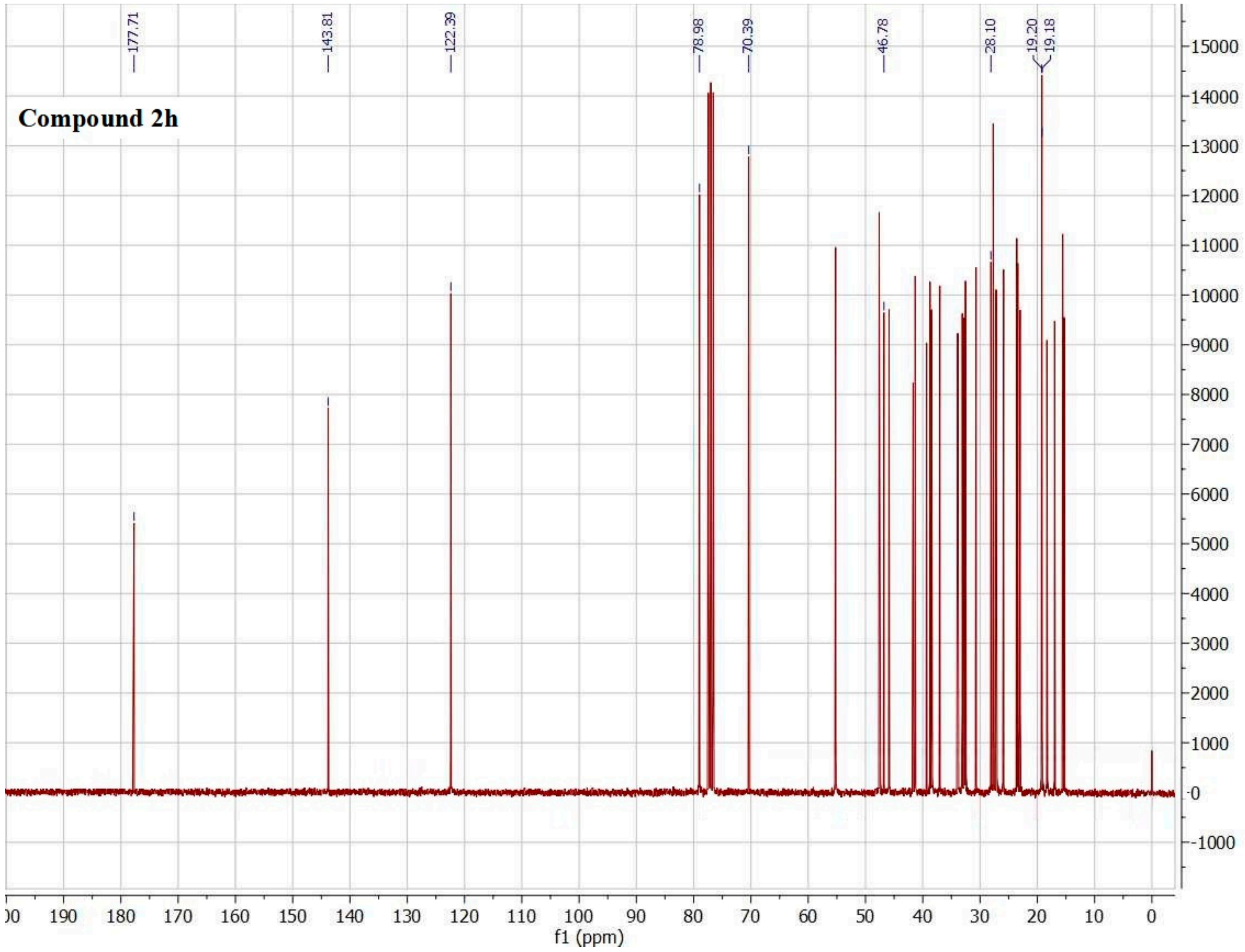
Compound 2h
177.71
143.81
122.39
78.98
70.39
46.78
28.10
19.20
19.18
f1 (ppm)

Compound 2i

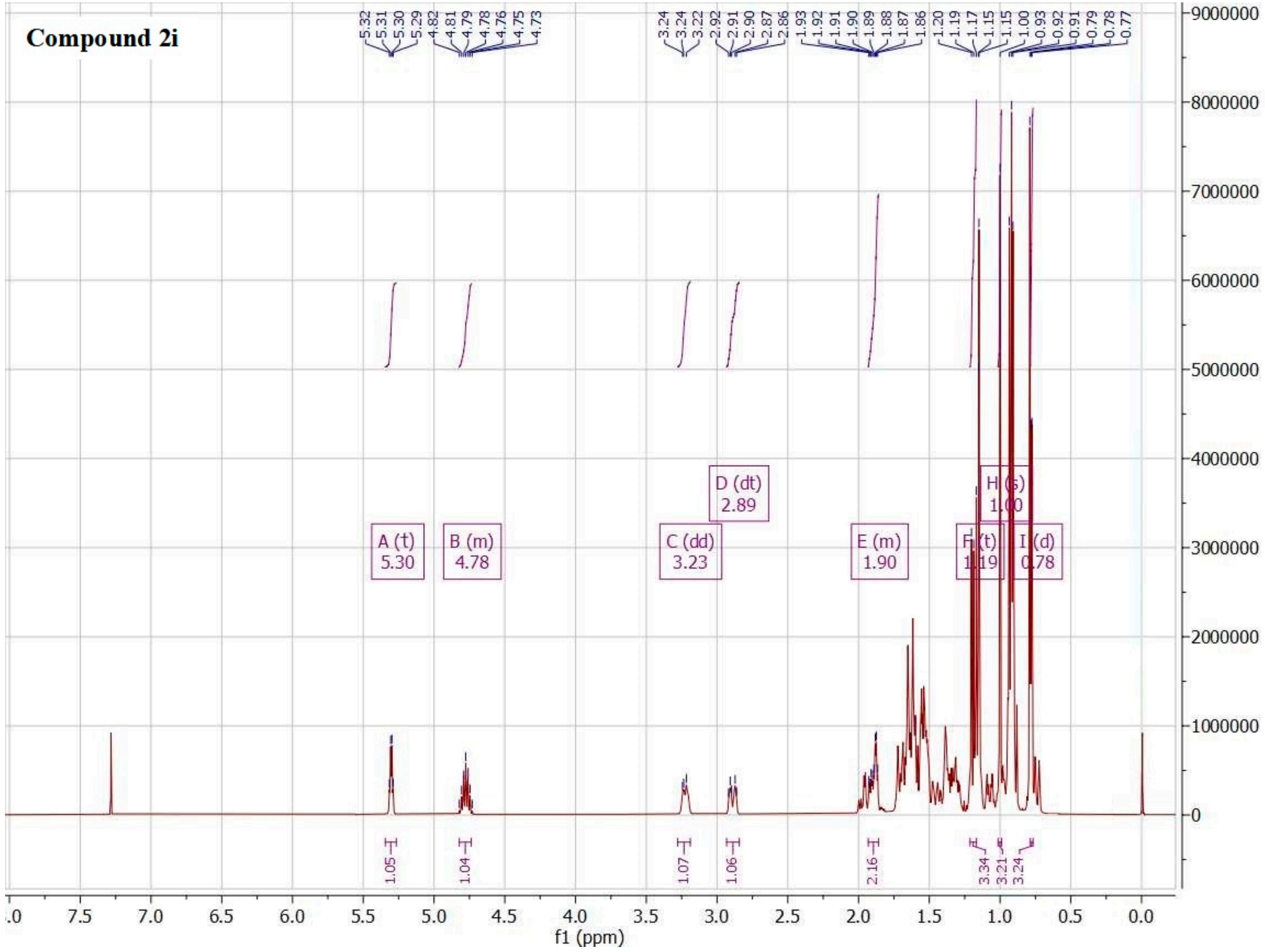

5.32
5.31
5.30
5.29
4.82
4.81
4.79
4.78
4.76
4.75
4.73
3.24
3.24
3.22
2.92
2.91
2.90
2.87
2.86
1.93
1.92
1.91
1.90
1.89
1.88
1.87
1.86
1.20
1.19
1.17
1.15
1.15
1.00
0.93
0.92
0.91
0.79
0.78
0.77
A (t) 5.30
B (m) 4.78
C (dd) 3.23
D (dt) 2.89
E (m) 1.90
F (t) 1.19
H (s) 1.00
I (d) 0.78
1.05
1.04
1.07
1.06
2.16
3.34
3.21
3.24
f1 (ppm)

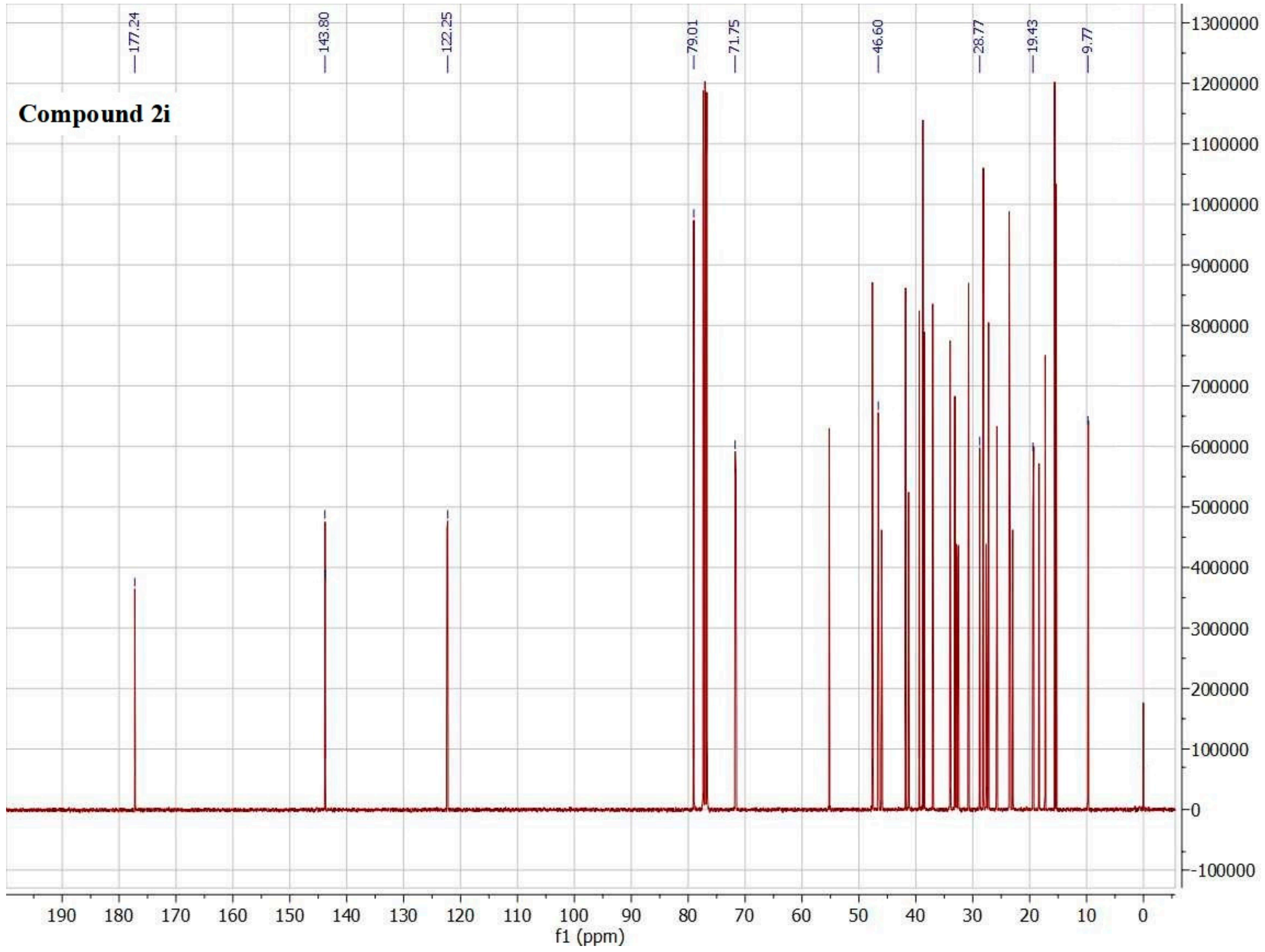
Compound 2i
177.24
143.80
122.25
79.01
71.75
46.60
28.77
19.43
9.77
f1 (ppm)

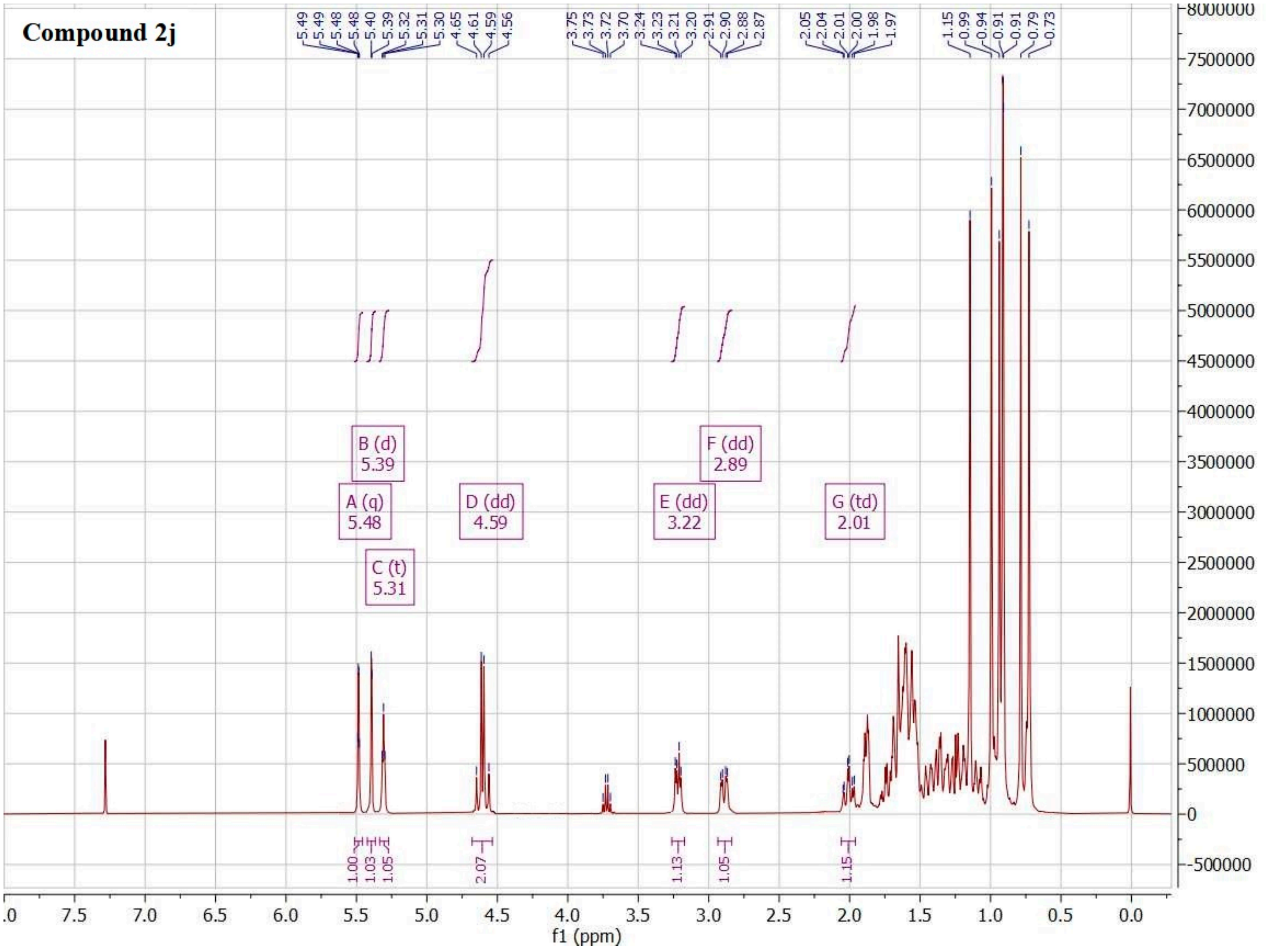
Compound 2j
5.49 5.49 5.48 5.48 5.40 5.39 5.32 5.31 5.30 4.65 4.61 4.59 4.56 3.75 3.73 3.72 3.70 3.24 3.23 3.21 3.20 2.91 2.90 2.88 2.87 2.05 2.04 2.01 2.00 1.98 1.97 1.15 0.99 0.94 0.91 0.91 0.79 0.73
A (q) 5.48
B (d) 5.39
C (t) 5.31
D (dd) 4.59
E (dd) 3.22
F (dd) 2.89
G (td) 2.01
1.00 1.03 1.05 2.07 1.13 1.05 1.15
f1 (ppm)

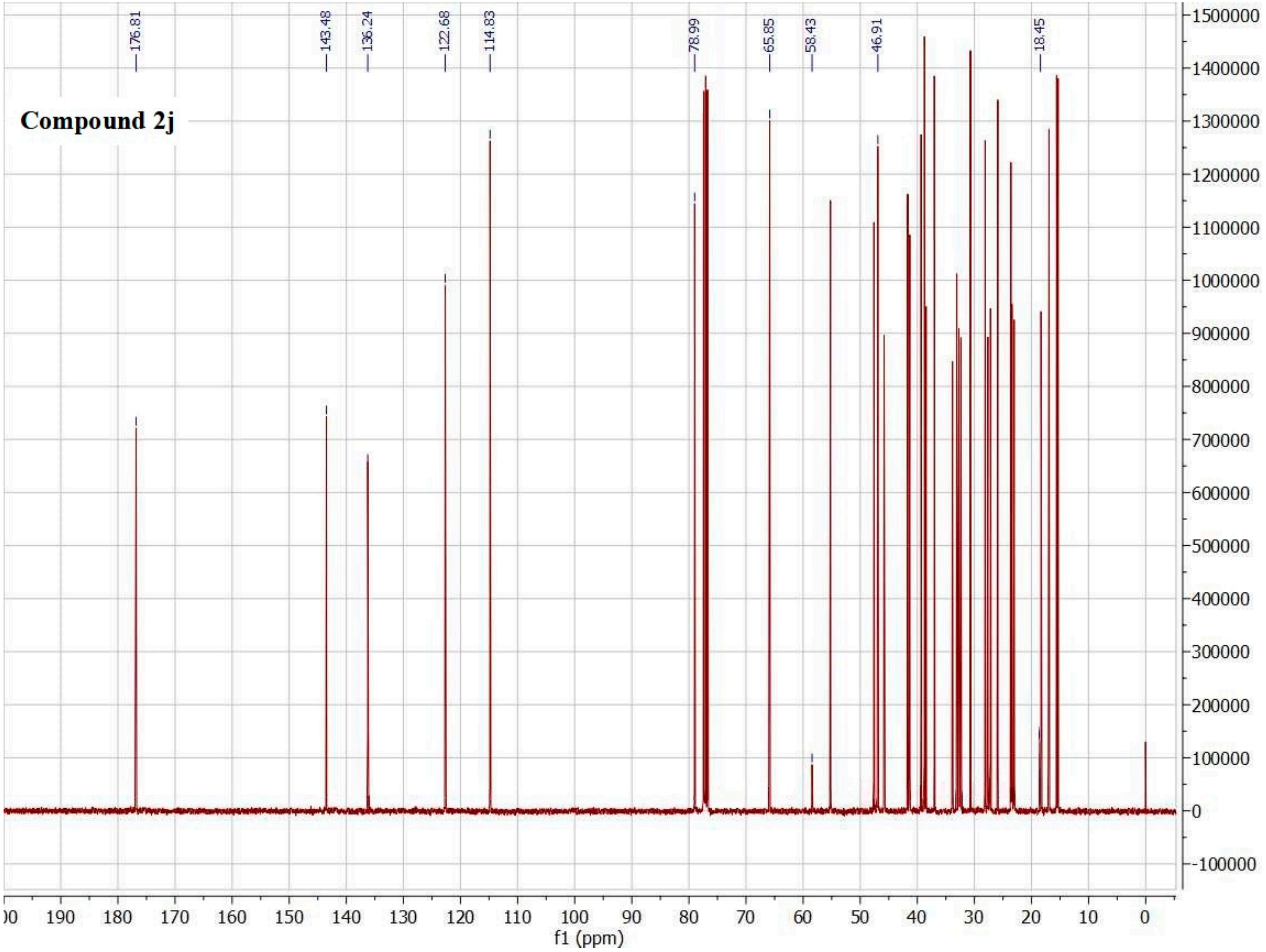

Compound 2j
176.81
143.48
136.24
122.68
114.83
78.99
65.85
58.43
46.91
18.45
f1 (ppm)

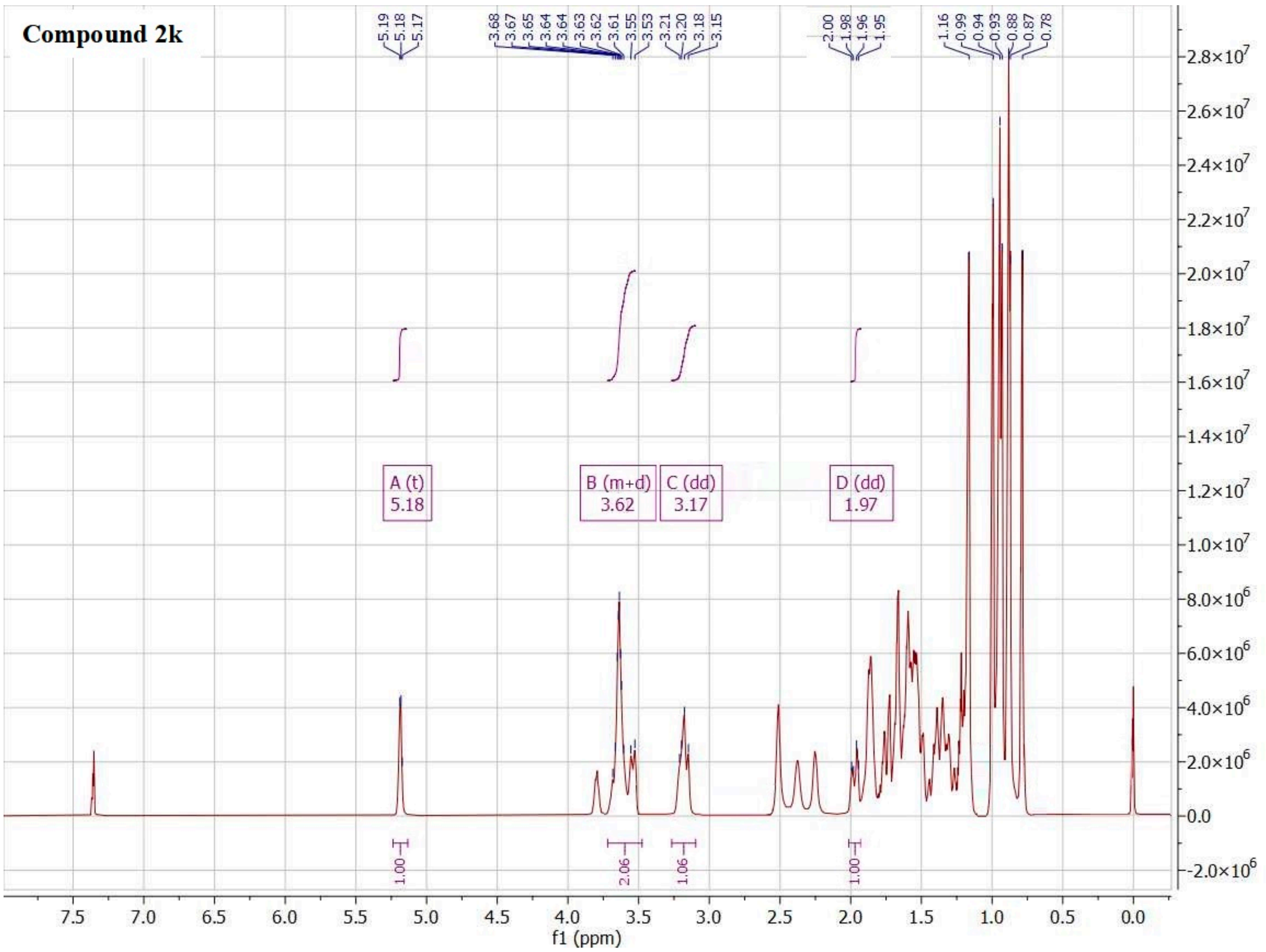

Compound 2k
5.19
5.18
5.17
3.68
3.67
3.65
3.64
3.64
3.63
3.62
3.61
3.55
3.53
3.21
3.20
3.18
3.15
2.00
1.98
1.96
1.95
1.16
0.99
0.94
0.93
0.88
0.87
0.78
A (t) 5.18
B (m+d) 3.62
C (dd) 3.17
D (dd) 1.97
1.00
2.06
1.06
1.00
f1 (ppm)

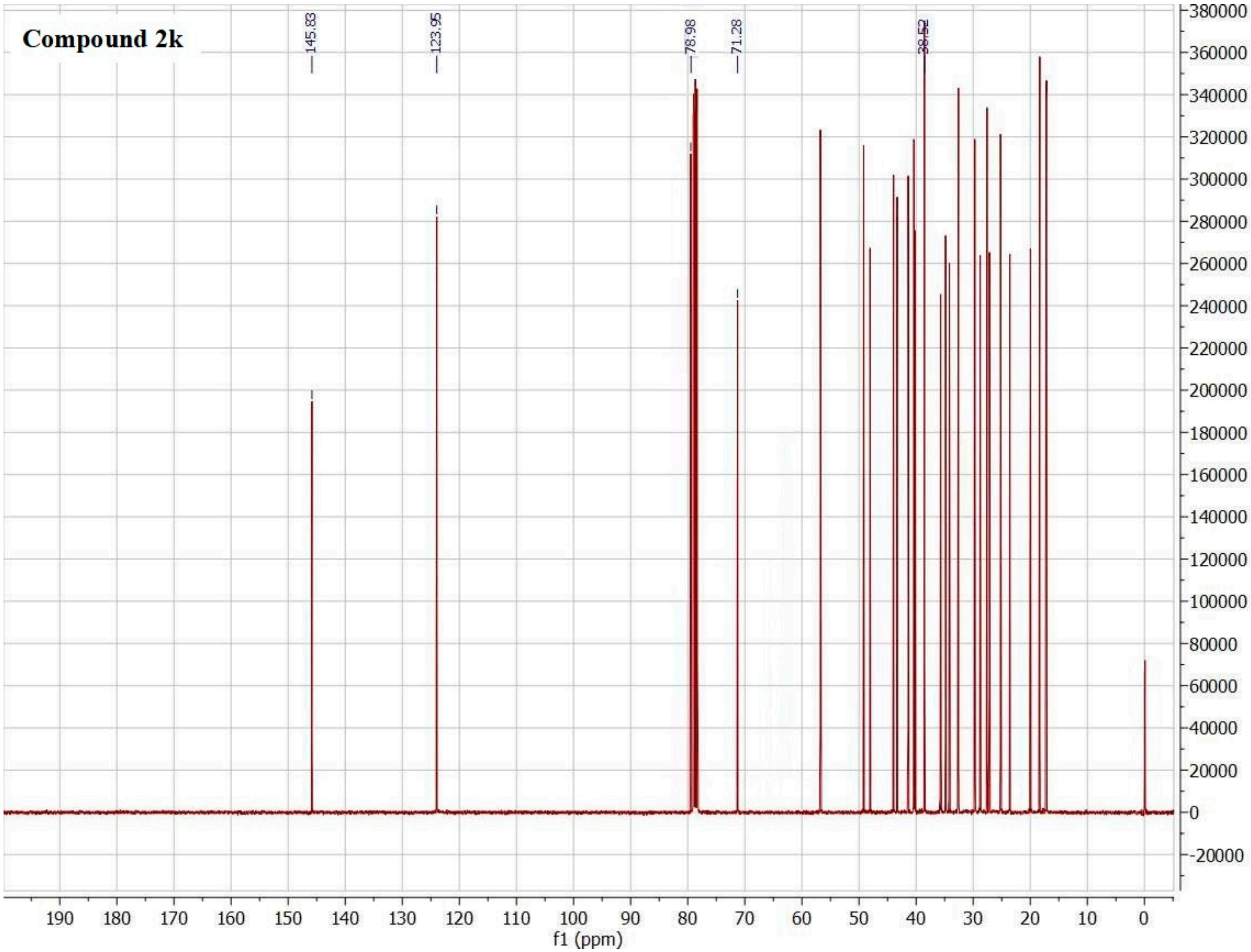
Compound 2k
145.83
123.95
78.98
71.28
38.52
f1 (ppm)

Article

Short-Chain Oleanolic Acid Esters and Furoyl Hybrids: Pharmacological Prediction, ADMETox Profiling, In Vitro Cytotoxicity Evaluation, Antioxidant Testing and EGFR Docking

Barbara Bednarczyk-Cwynar[1,2,*], Piotr Ruszkowski[3], Maciej Kulawik[4,5], Szymon Sip[4], Przemysław Zalewski[4], Dobrosława Wiśniewska[1], Andrzej Günther[1]

Supplementary Materials

Figure S2. ^{1}H NMR and ^{13}C NMR spectra of compounds **3a – 3k**

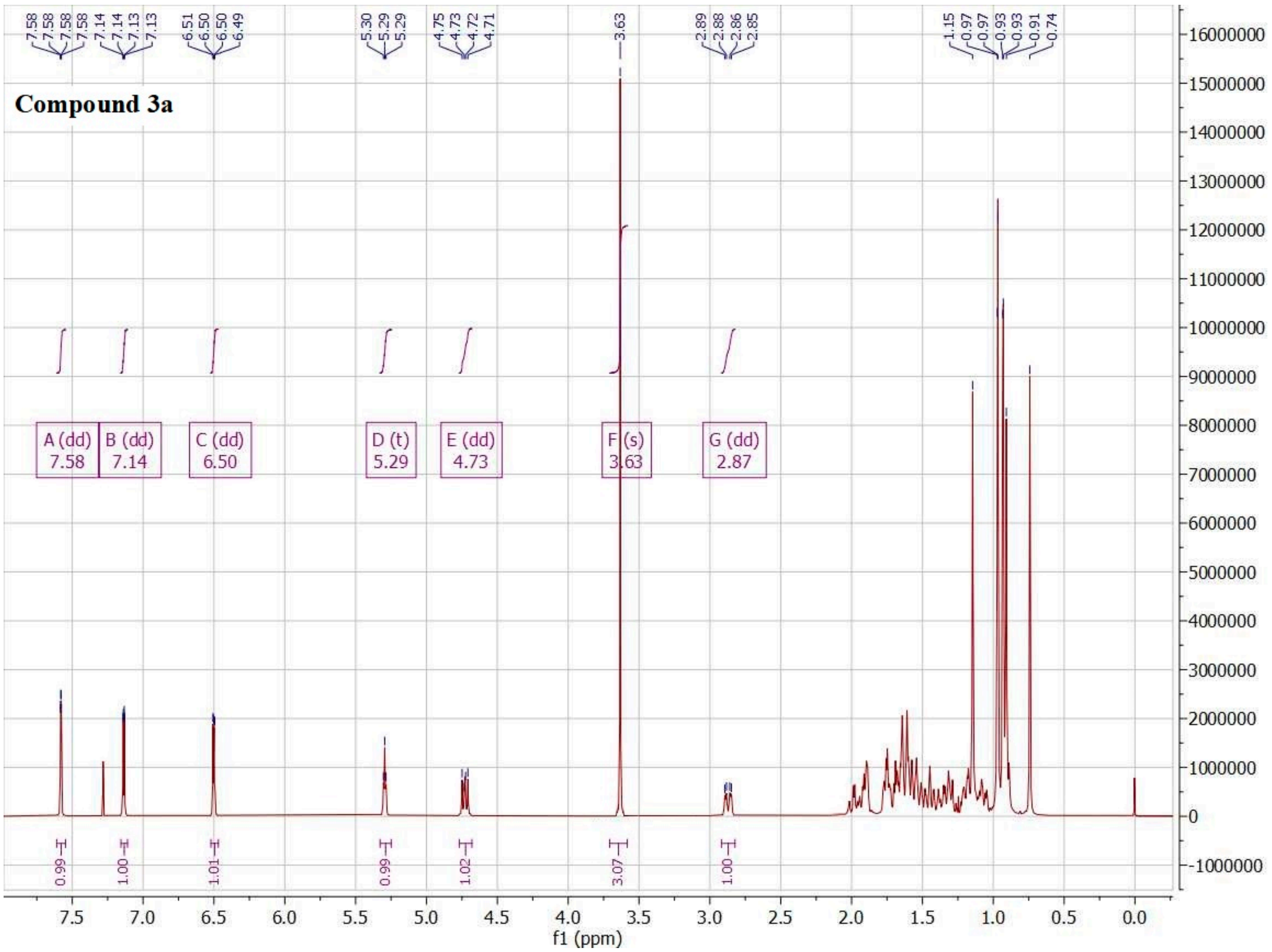
Compound 3a
A (dd) 7.58
B (dd) 7.14
C (dd) 6.50
D (t) 5.29
E (dd) 4.73
F (s) 3.63
G (dd) 2.87
0.99
1.00
1.01
0.99
1.02
3.07
1.00
f1 (ppm)

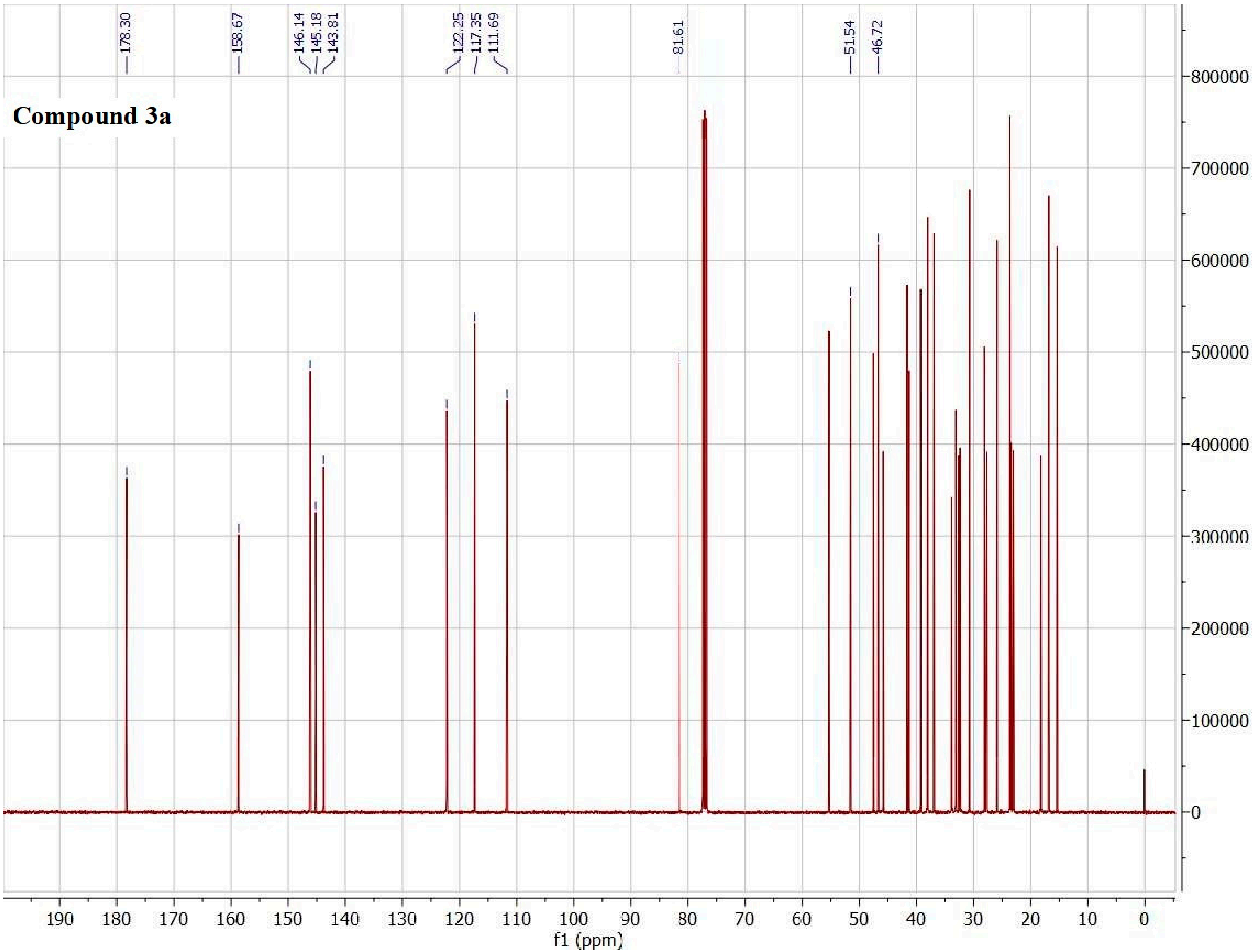

Compound 3a
178.30
158.67
146.14
145.18
143.81
122.25
117.35
111.69
81.61
51.54
46.72
800000
700000
600000
500000
400000
300000
200000
100000
0
190 180 170 160 150 140 130 120 110 100 90 80 70 60 50 40 30 20 10 0
f1 (ppm)

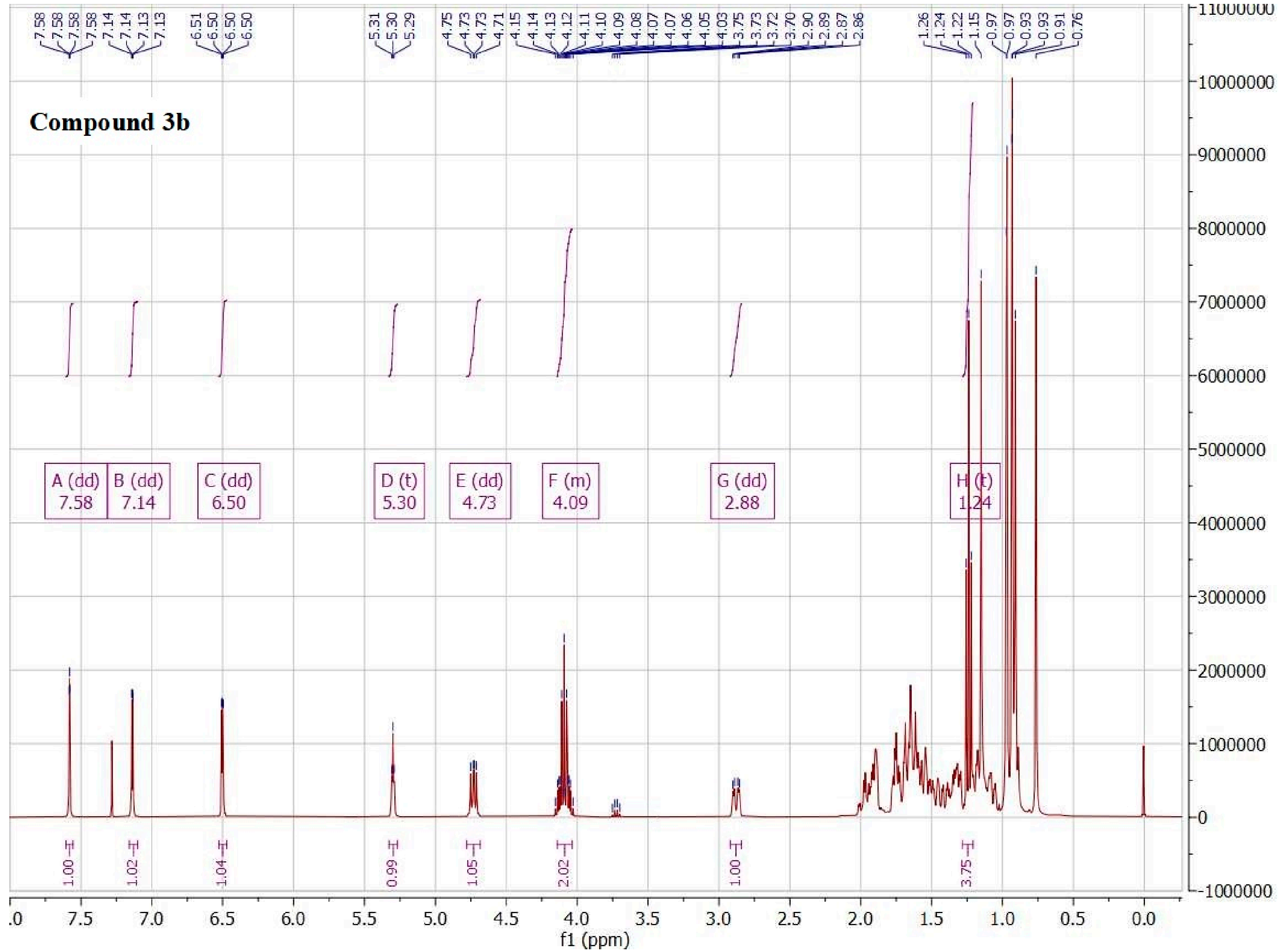
Compound 3b
7.58 7.58 7.58 7.58 7.14 7.14 7.13 7.13 6.51 6.50 6.50 6.50 5.31 5.30 5.29 4.75 4.73 4.73 4.71 4.15 4.14 4.13 4.12 4.11 4.10 4.09 4.08 4.07 4.07 4.06 4.05 4.03 3.75 3.73 3.72 3.70 2.90 2.89 2.87 2.86 1.26 1.24 1.22 1.15 0.97 0.97 0.93 0.93 0.91 0.76
A (dd) 7.58
B (dd) 7.14
C (dd) 6.50
D (t) 5.30
E (dd) 4.73
F (m) 4.09
G (dd) 2.88
H (t) 1.24
1.00
1.02
1.04
0.99
1.05
2.02
1.00
3.75
f1 (ppm)

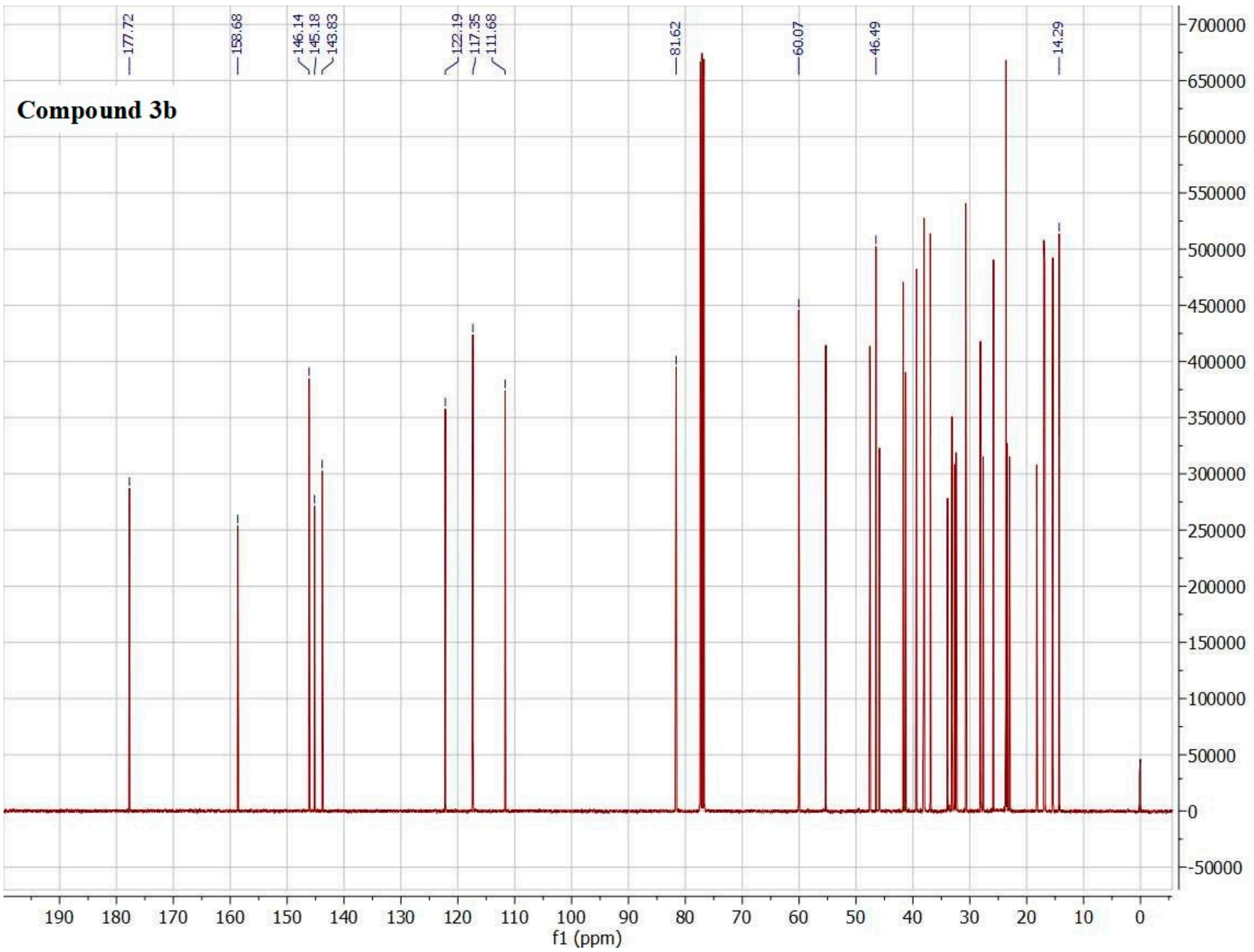

Compound 3b
177.72
158.68
146.14
145.18
143.83
122.19
117.35
111.68
81.62
60.07
46.49
14.29
700000
650000
600000
550000
500000
450000
400000
350000
300000
250000
200000
150000
100000
50000
0
-50000
190 180 170 160 150 140 130 120 110 100 90 80 70 60 50 40 30 20 10 0
f1 (ppm)

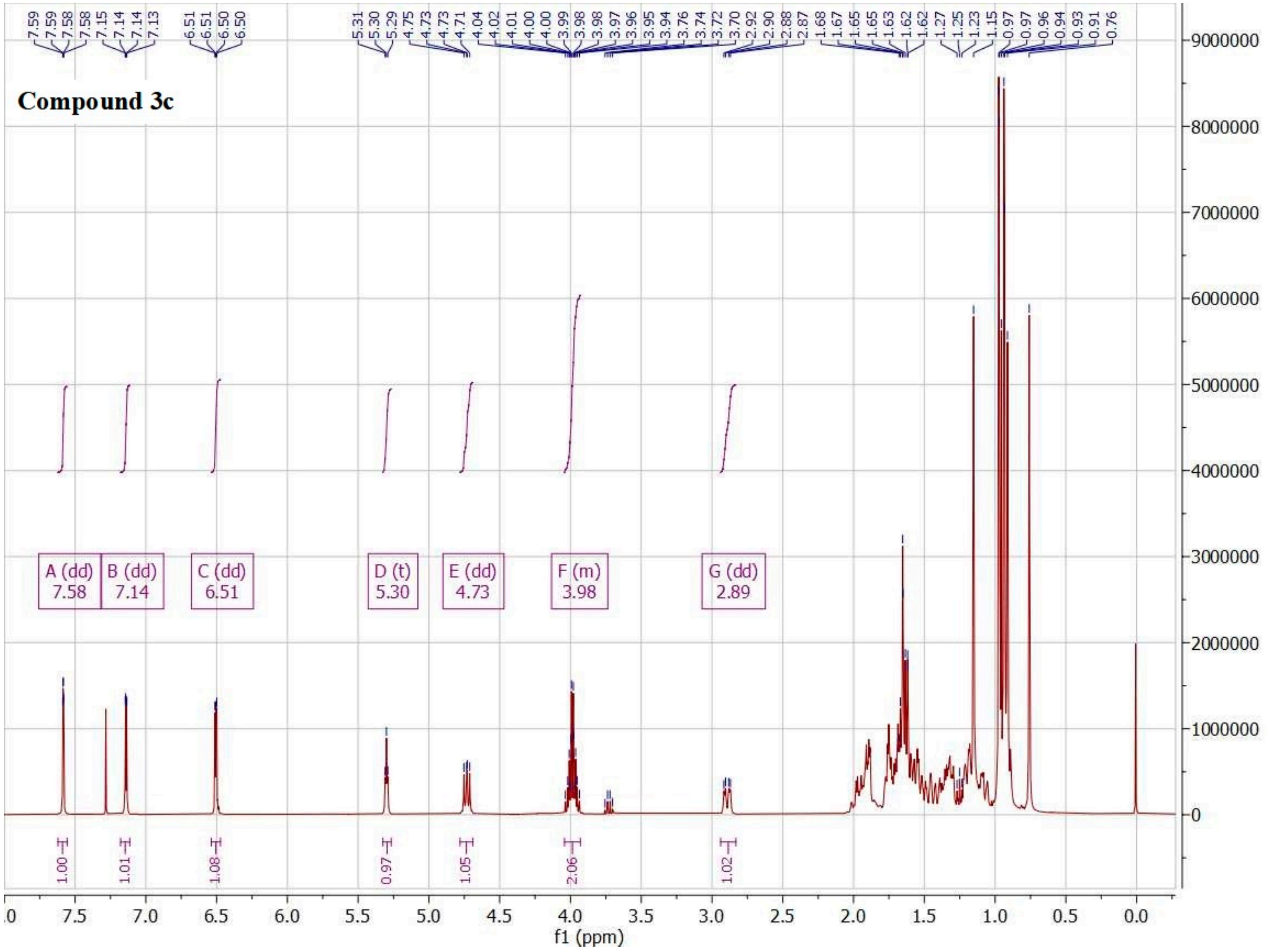

Compound 3c
7.59 7.59 7.58 7.58 7.15 7.14 7.14 7.13 6.51 6.51 6.50 6.50 5.31 5.30 5.29 4.75 4.73 4.73 4.71 4.04 4.02 4.01 4.00 4.00 3.99 3.98 3.98 3.97 3.96 3.95 3.94 3.76 3.74 3.72 3.70 2.92 2.90 2.88 2.87 1.68 1.67 1.65 1.65 1.63 1.62 1.62 1.27 1.25 1.23 1.15 0.97 0.97 0.96 0.94 0.93 0.91 0.76
A (dd) 7.58
B (dd) 7.14
C (dd) 6.51
D (t) 5.30
E (dd) 4.73
F (m) 3.98
G (dd) 2.89
1.00
1.01
1.08
0.97
1.05
2.06
1.02
9000000
8000000
7000000
6000000
5000000
4000000
3000000
2000000
1000000
0
f1 (ppm)

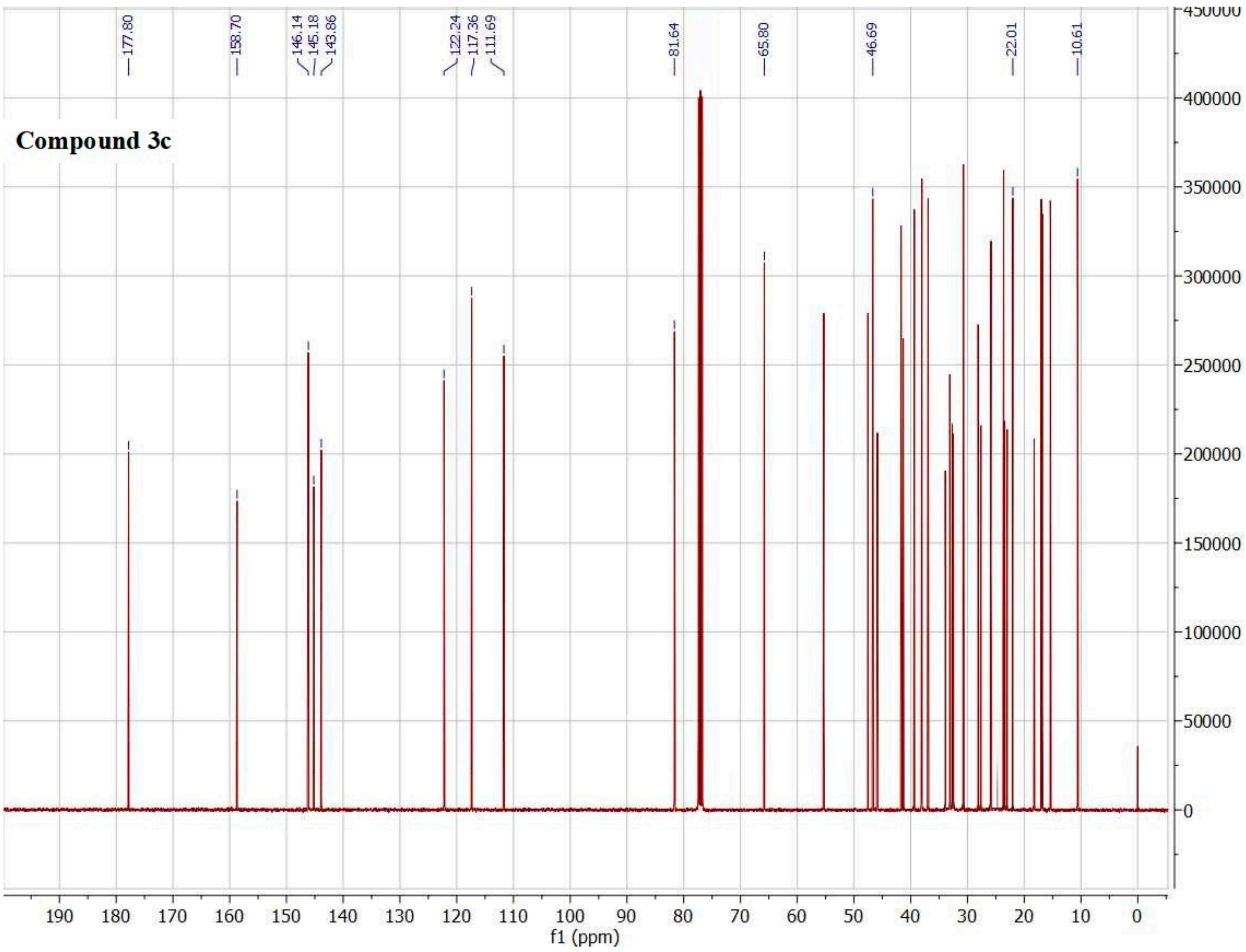
Compound 3c
177.80
158.70
146.14
145.18
143.86
122.24
117.36
111.69
81.64
65.80
46.69
22.01
10.61
450000
400000
350000
300000
250000
200000
150000
100000
50000
0
190 180 170 160 150 140 130 120 110 100 90 80 70 60 50 40 30 20 10 0
f1 (ppm)

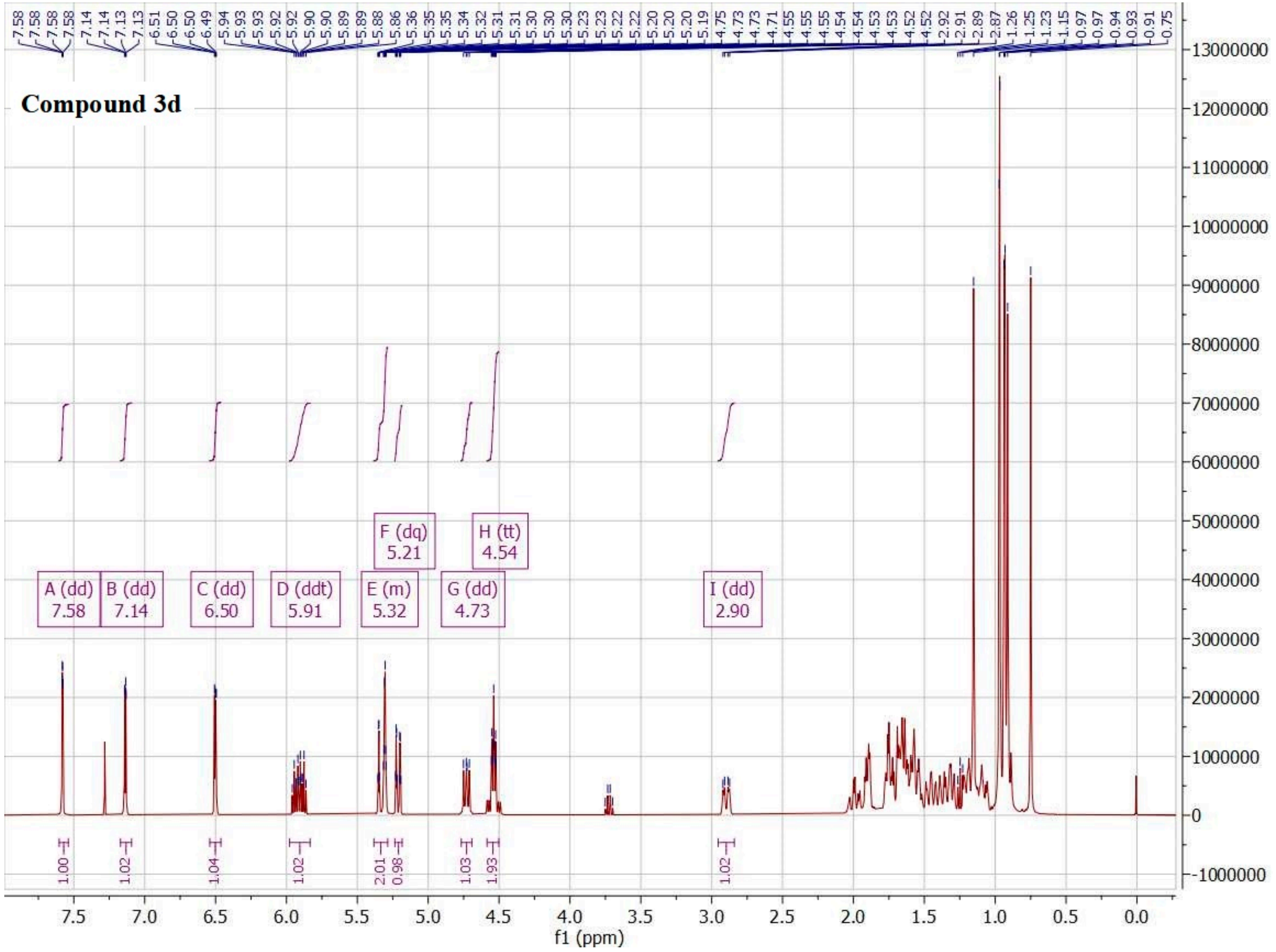
Compound 3d
A (dd) 7.58
B (dd) 7.14
C (dd) 6.50
D (ddt) 5.91
E (m) 5.32
F (dq) 5.21
G (dd) 4.73
H (tt) 4.54
I (dd) 2.90
f1 (ppm)

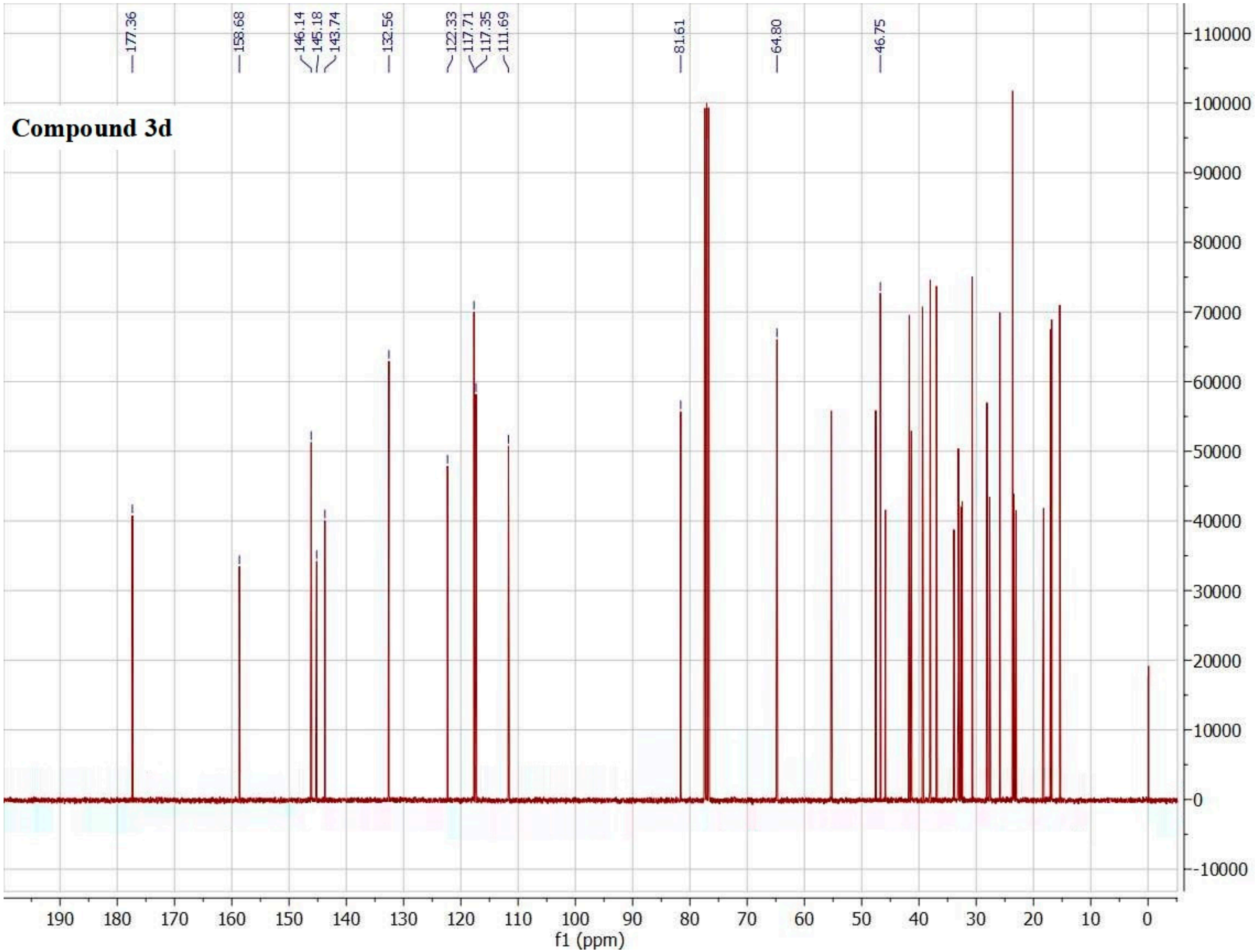

Compound 3d
177.36
158.68
146.14
145.18
143.74
132.56
122.33
117.71
117.35
111.69
81.61
64.80
46.75
f1 (ppm)

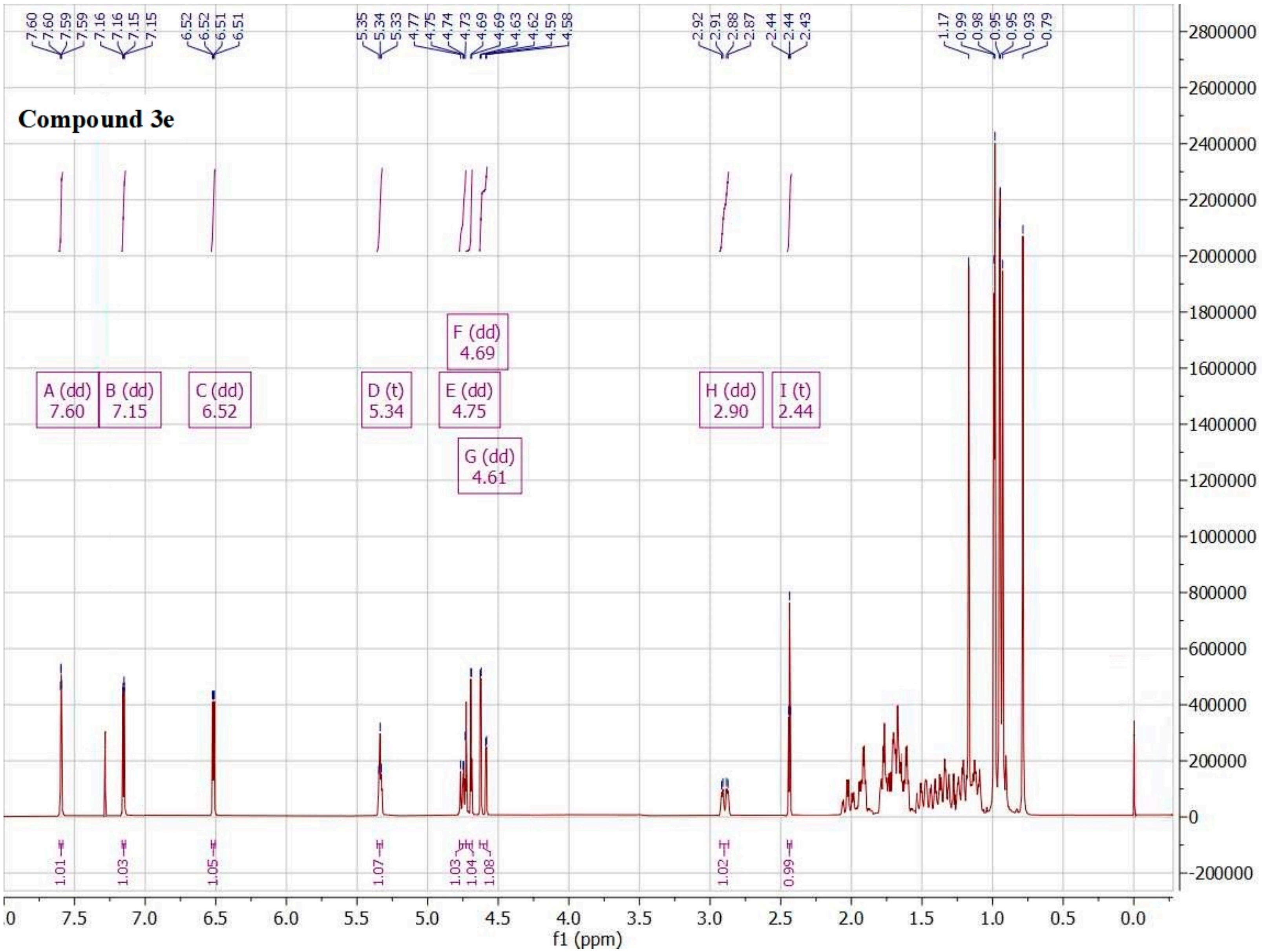

Compound 3e
7.60
7.60
7.59
7.59
7.16
7.16
7.15
7.15
6.52
6.52
6.51
6.51
5.35
5.34
5.33
4.77
4.75
4.74
4.73
4.69
4.63
4.62
4.59
4.58
2.92
2.91
2.88
2.87
2.44
2.44
2.43
1.17
0.99
0.98
0.95
0.95
0.93
0.79
A (dd) 7.60
B (dd) 7.15
C (dd) 6.52
D (t) 5.34
E (dd) 4.75
F (dd) 4.69
G (dd) 4.61
H (dd) 2.90
I (t) 2.44
1.01
1.03
1.05
1.07
1.03
1.04
1.08
1.02
0.99
f1 (ppm)

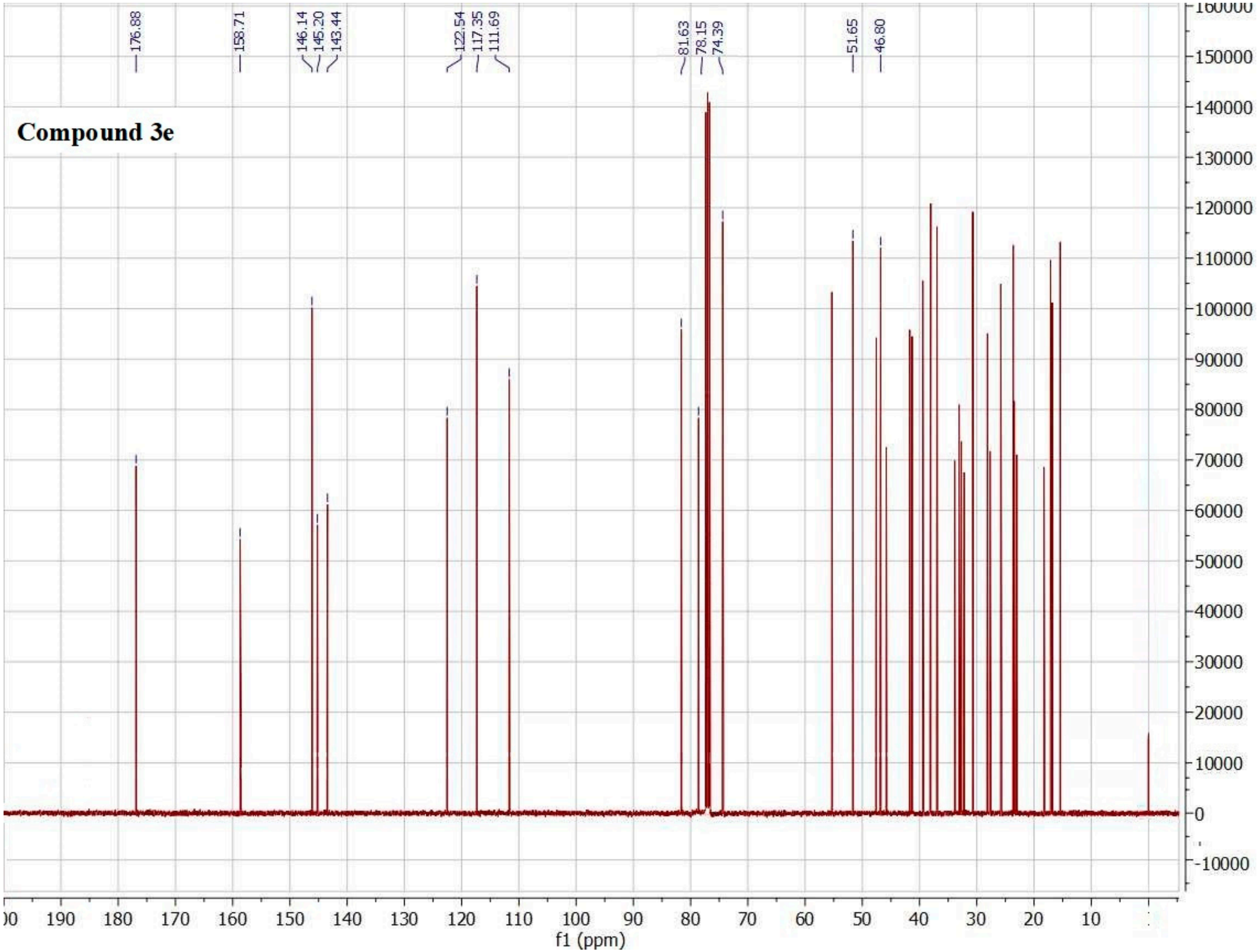

Compound 3e
176.88
158.71
146.14
145.20
143.44
122.54
117.35
111.69
81.63
78.15
74.39
51.65
46.80
f1 (ppm)

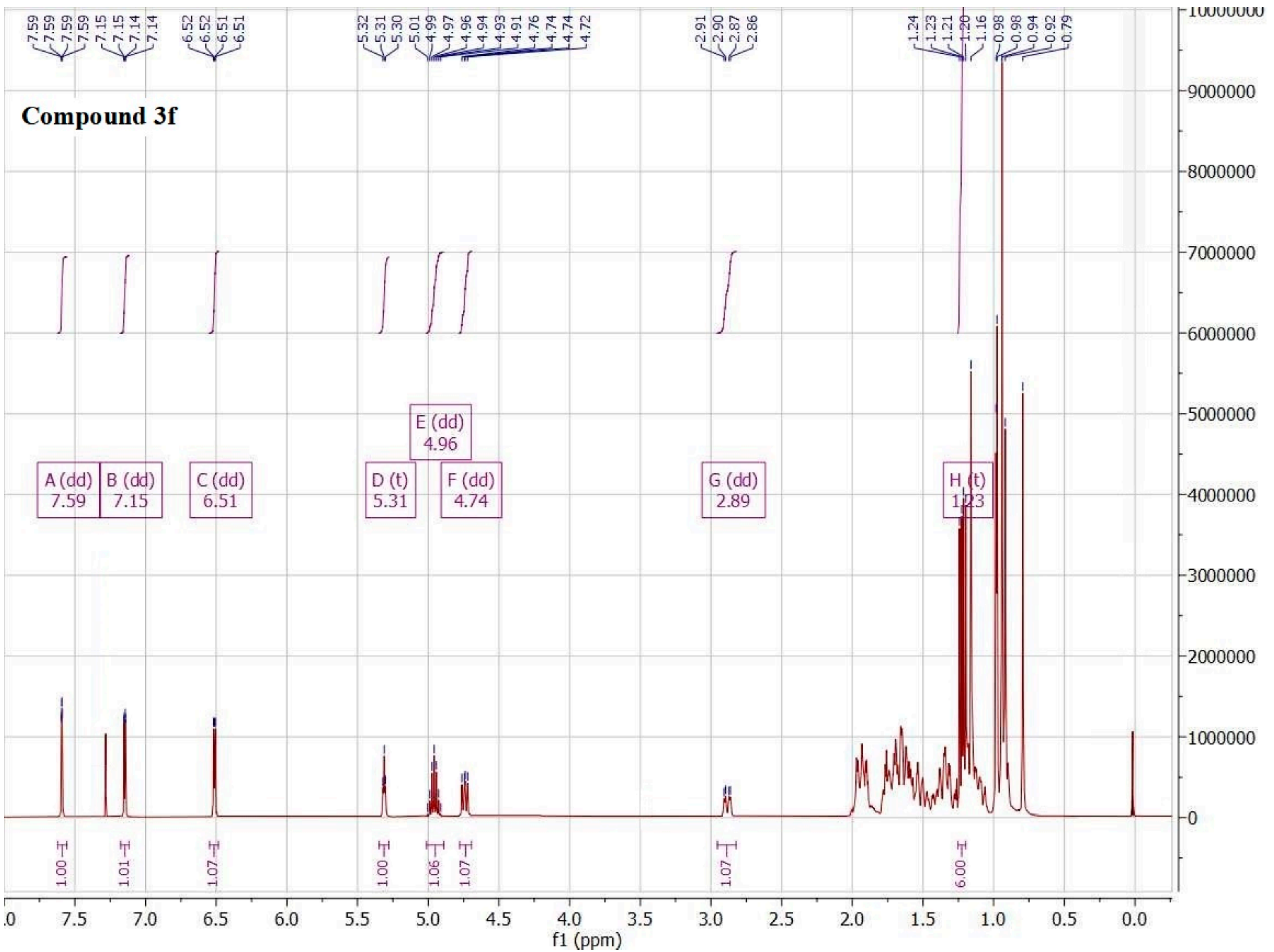
Compound 3f
A (dd) 7.59
B (dd) 7.15
C (dd) 6.51
D (t) 5.31
E (dd) 4.96
F (dd) 4.74
G (dd) 2.89
H (t) 1.23
f1 (ppm)

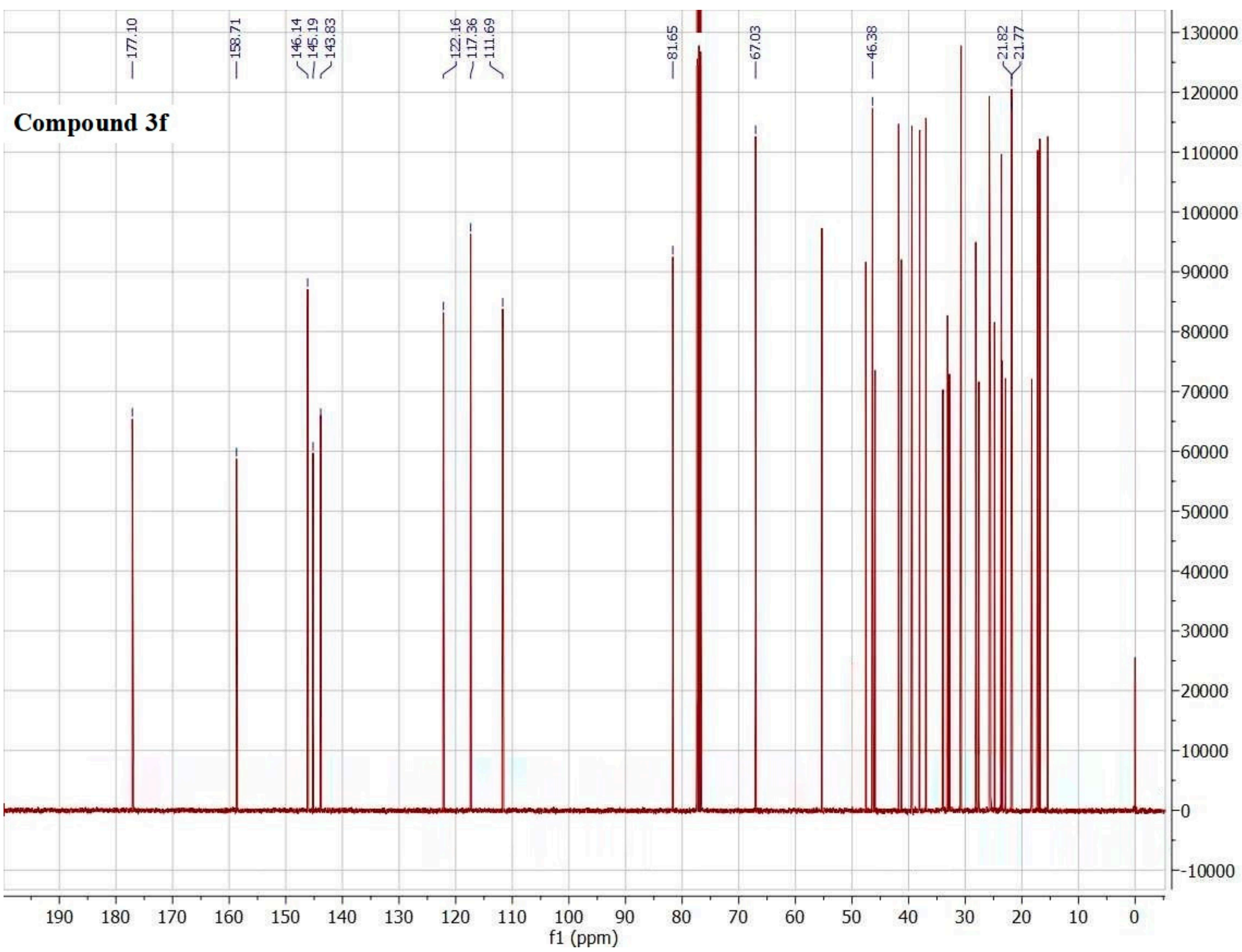

Compound 3f
177.10
158.71
146.14
145.19
143.83
122.16
117.36
111.69
81.65
67.03
46.38
21.82
21.77
130000
120000
110000
100000
90000
80000
70000
60000
50000
40000
30000
20000
10000
0
-10000
190 180 170 160 150 140 130 120 110 100 90 80 70 60 50 40 30 20 10 0
f1 (ppm)

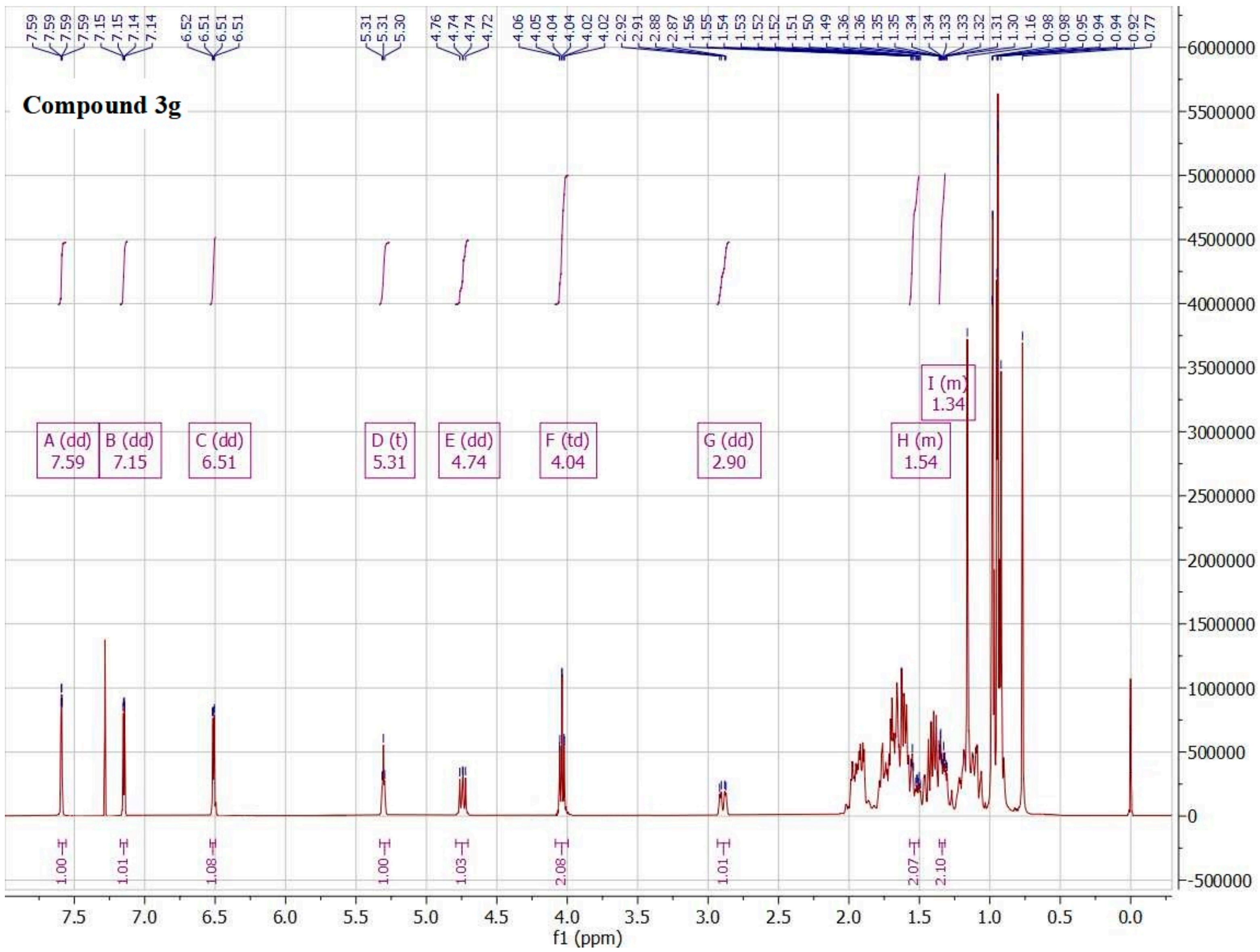
Compound 3g
A (dd) 7.59
B (dd) 7.15
C (dd) 6.51
D (t) 5.31
E (dd) 4.74
F (td) 4.04
G (dd) 2.90
H (m) 1.54
I (m) 1.34
1.00
1.01
1.08
1.00
1.03
2.08
1.01
2.07
2.10
f1 (ppm)

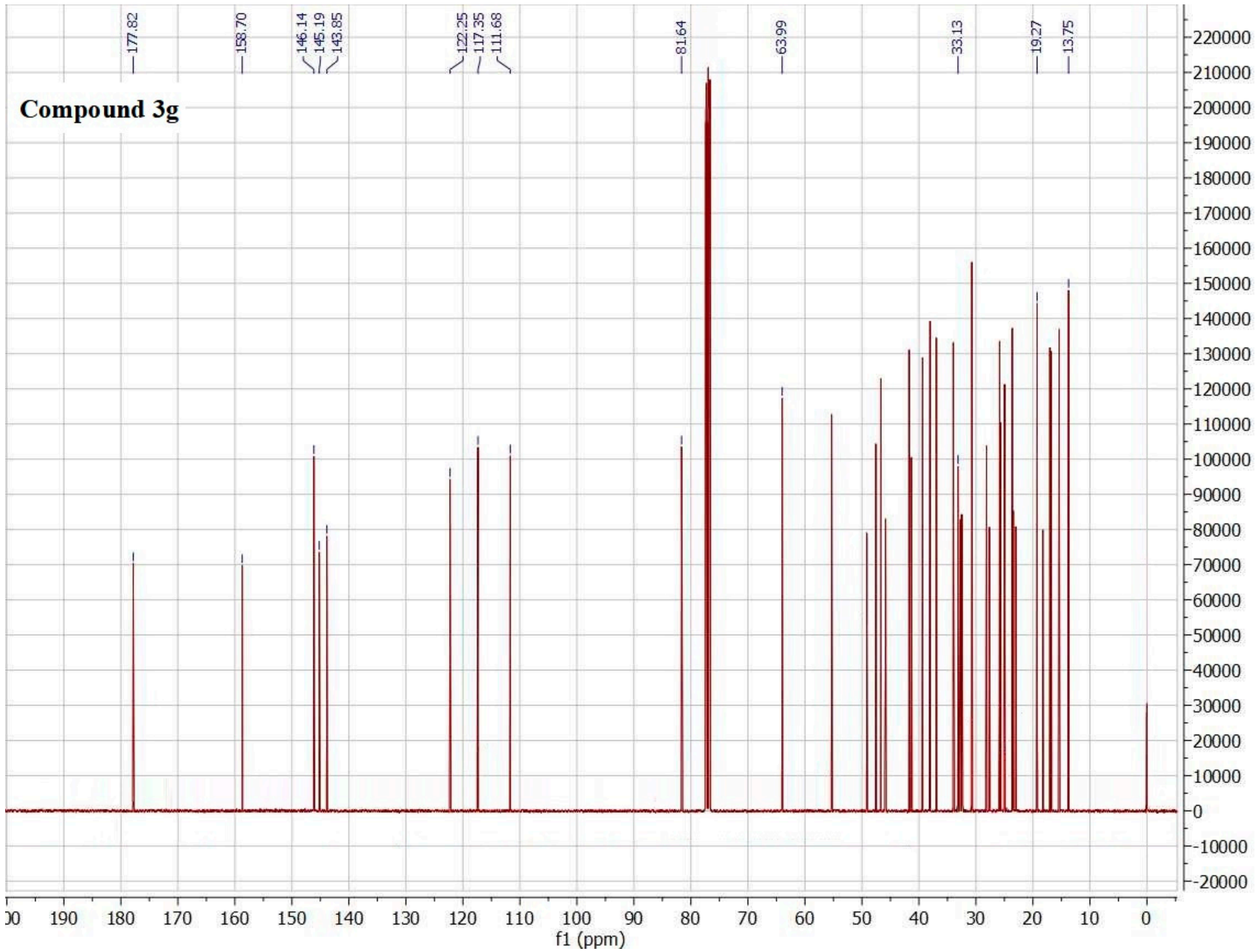
Compound 3g
177.82
158.70
146.14
145.19
143.85
122.25
117.35
111.68
81.64
63.99
33.13
19.27
13.75
f1 (ppm)

Compound 3h

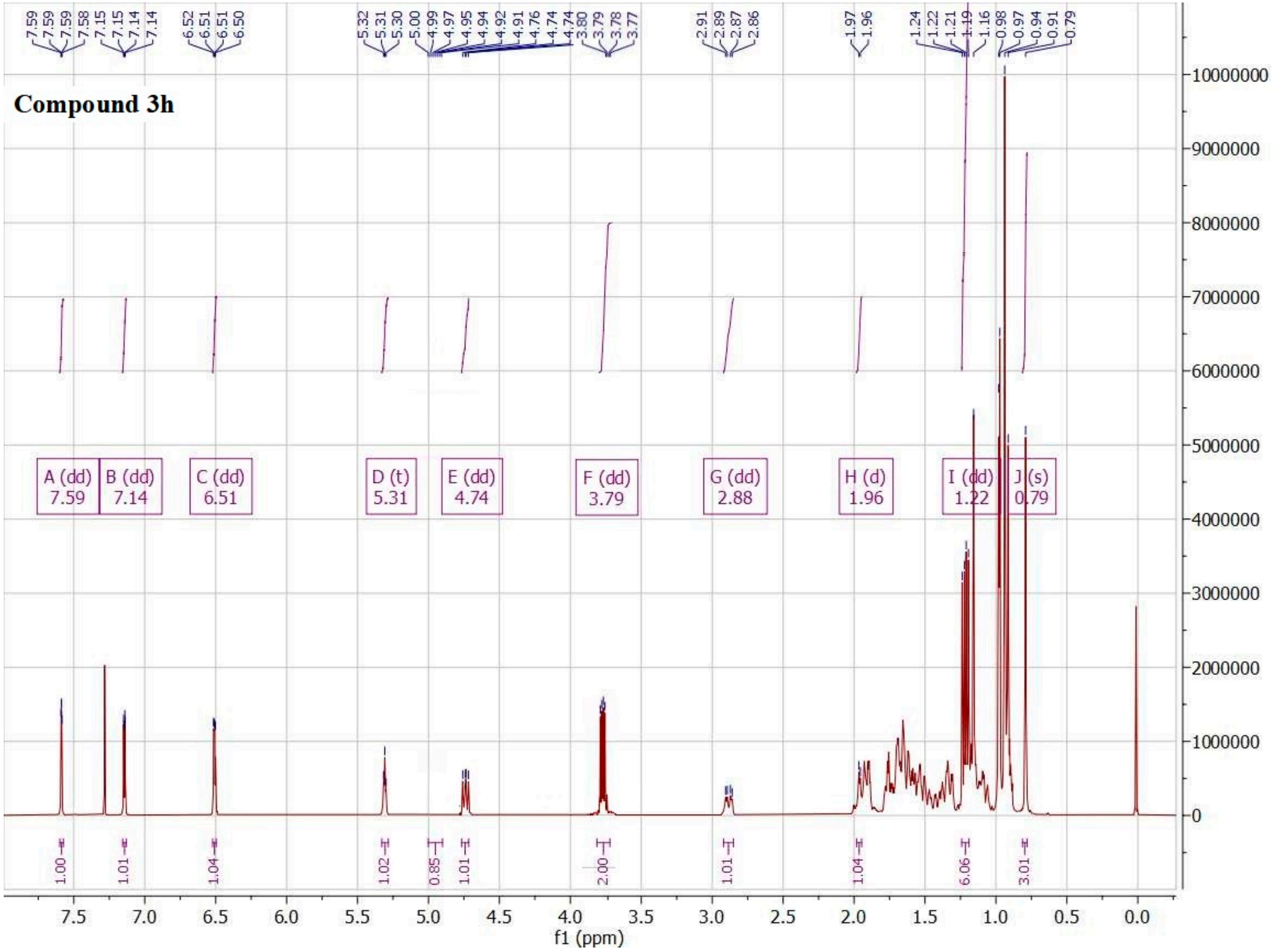

7.59
7.59
7.59
7.58
7.15
7.15
7.14
7.14
6.52
6.51
6.51
6.50
5.32
5.31
5.30
5.00
4.99
4.97
4.95
4.94
4.92
4.91
4.76
4.74
4.74
3.80
3.79
3.78
3.77
2.91
2.89
2.87
2.86
1.97
1.96
1.24
1.22
1.21
1.19
1.16
0.98
0.97
0.94
0.91
0.79
A (dd) 7.59
B (dd) 7.14
C (dd) 6.51
D (t) 5.31
E (dd) 4.74
F (dd) 3.79
G (dd) 2.88
H (d) 1.96
I (dd) 1.22
J (s) 0.79
1.00
1.01
1.04
1.02
0.85
1.01
2.00
1.01
1.04
6.06
3.01
10000000
9000000
8000000
7000000
6000000
5000000
4000000
3000000
2000000
1000000
0
7.5
7.0
6.5
6.0
5.5
5.0
4.5
4.0
3.5
3.0
2.5
2.0
1.5
1.0
0.5
0.0
f1 (ppm)

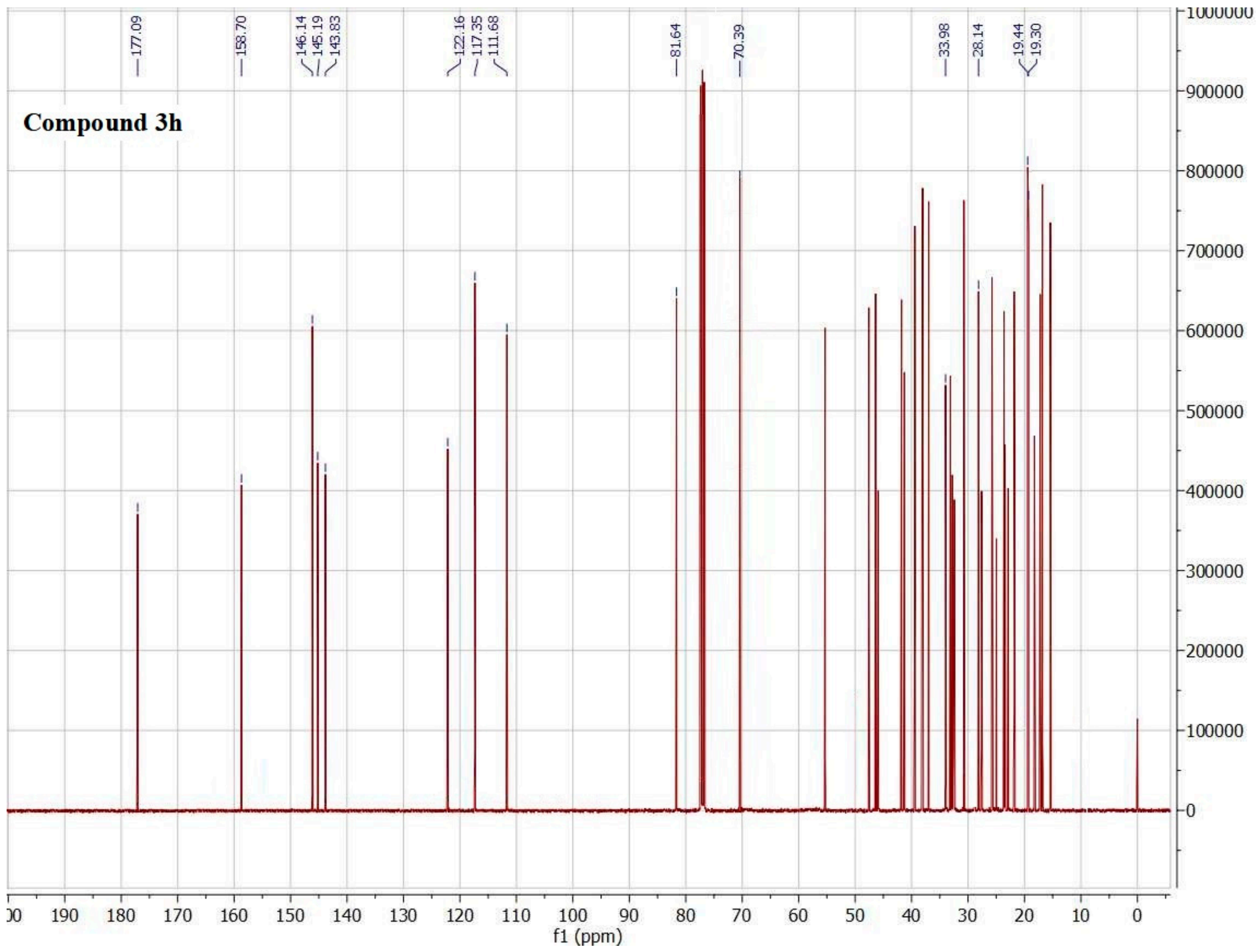

Compound 3h
177.09
158.70
146.14
145.19
143.83
122.16
117.35
111.68
81.64
70.39
33.98
28.14
19.44
19.30
f1 (ppm)

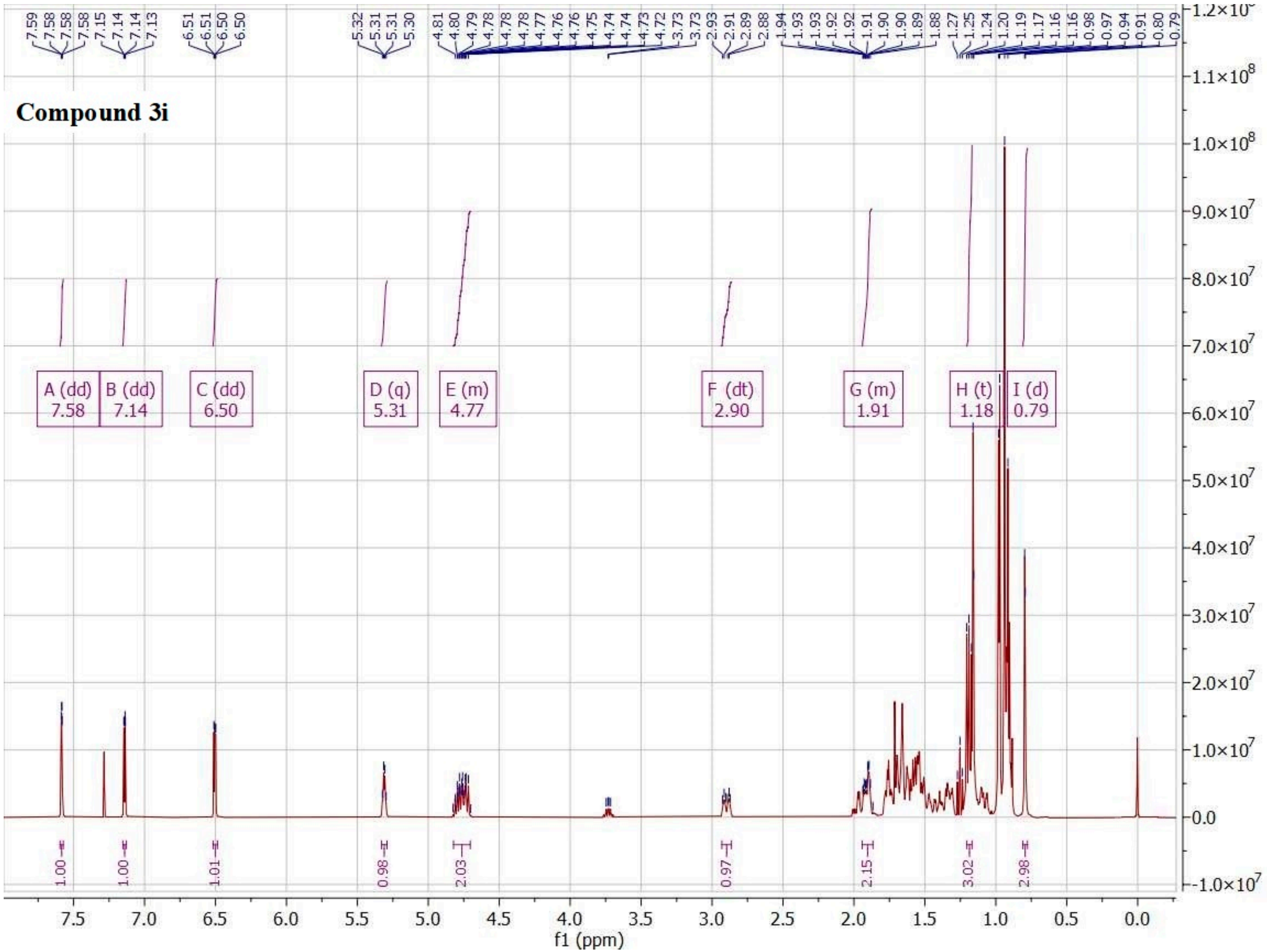
Compound 3i
A (dd) 7.58
B (dd) 7.14
C (dd) 6.50
D (q) 5.31
E (m) 4.77
F (dt) 2.90
G (m) 1.91
H (t) 1.18
I (d) 0.79
1.00
1.00
1.01
0.98
2.03
0.97
2.15
3.02
2.98
f1 (ppm)

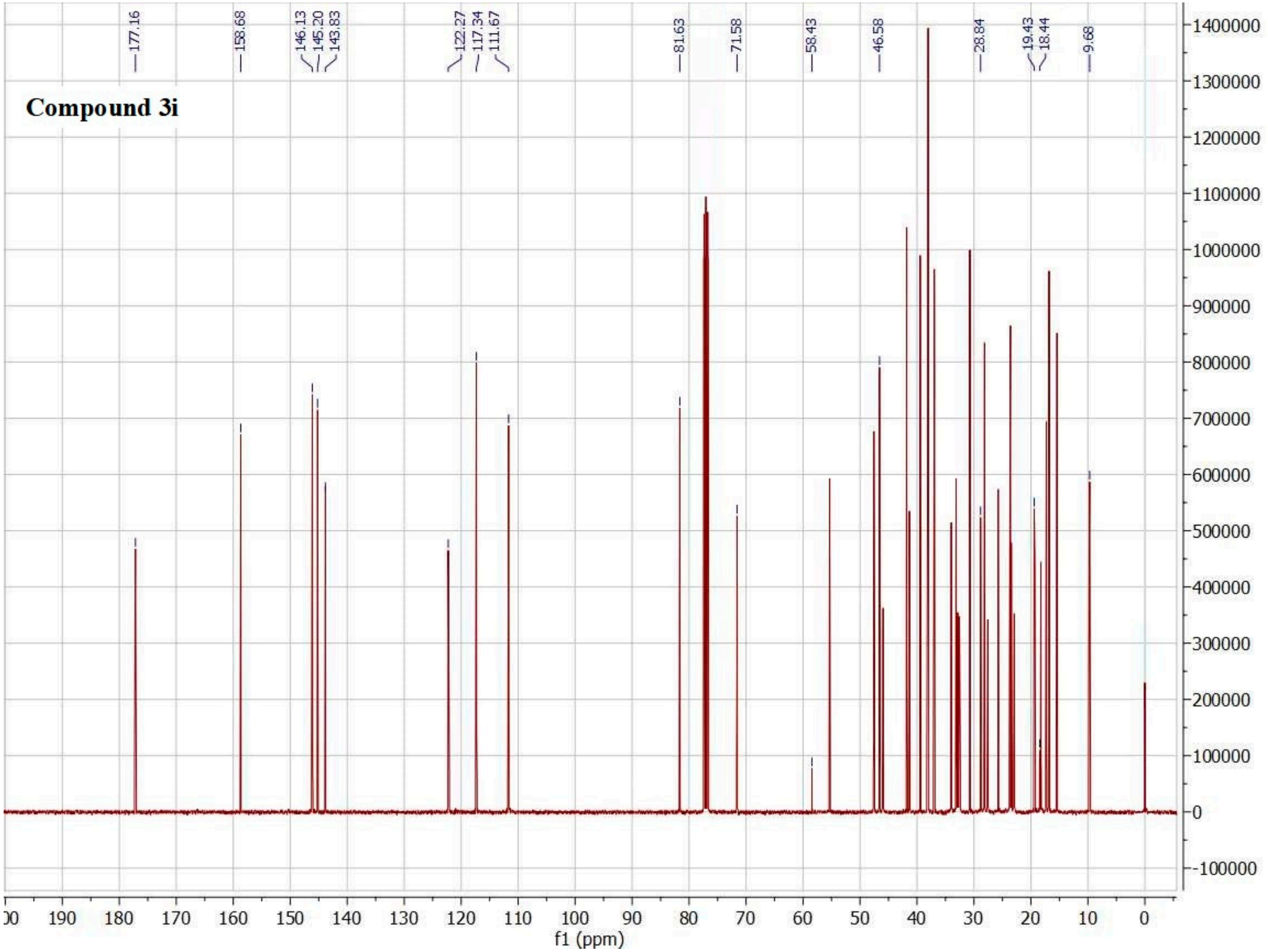

Compound 3i
177.16
158.68
146.13
145.20
143.83
122.27
117.34
111.67
81.63
71.58
58.43
46.58
28.84
19.43
18.44
9.68
f1 (ppm)

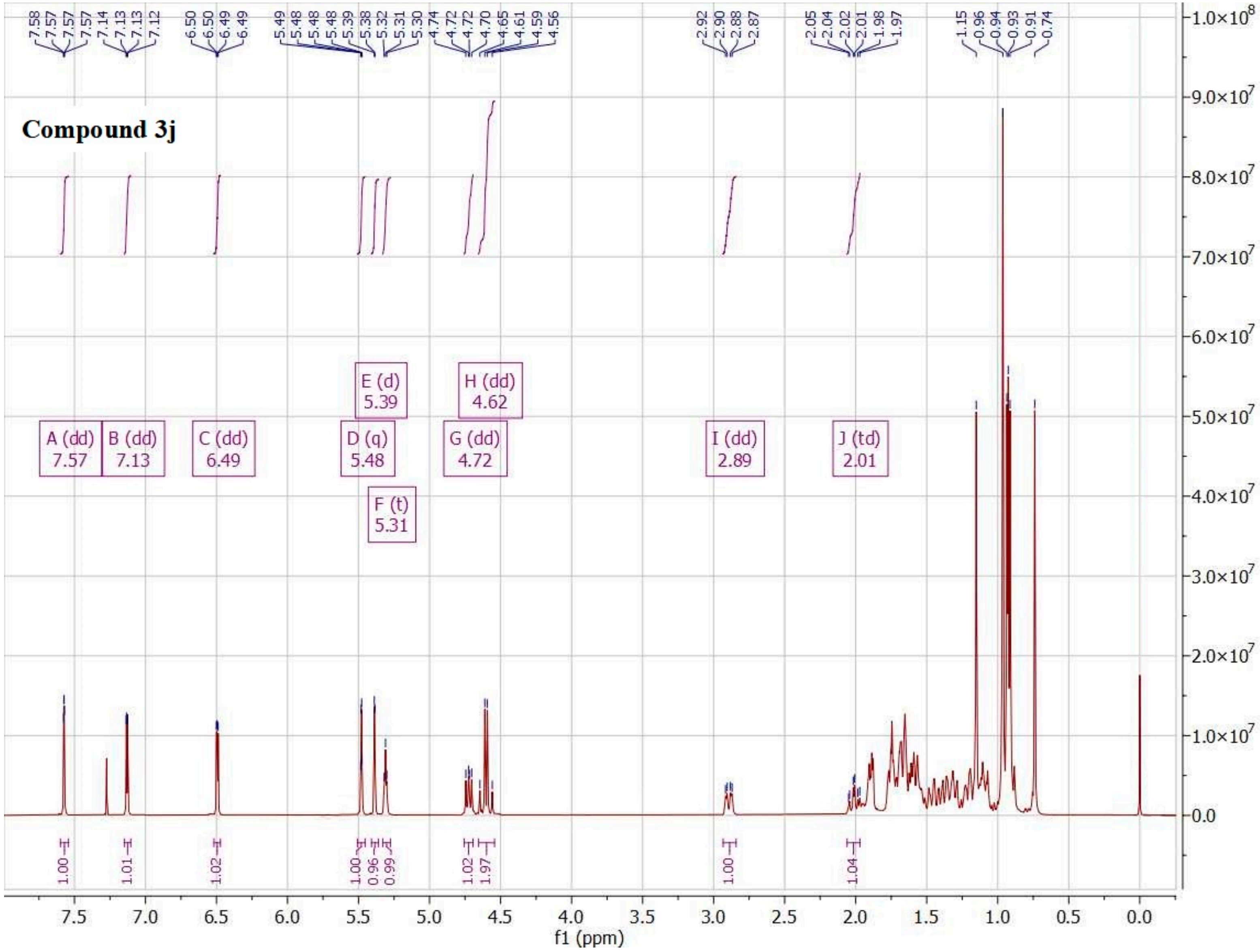
Compound 3j
7.58 7.57 7.57 7.57 7.14 7.13 7.13 7.12 6.50 6.50 6.49 6.49 5.49 5.48 5.48 5.48 5.39 5.38 5.32 5.31 5.30 4.74 4.72 4.72 4.70 4.65 4.61 4.59 4.56 2.92 2.90 2.88 2.87 2.05 2.04 2.02 2.01 1.98 1.97 1.15 0.96 0.94 0.93 0.91 0.74
A (dd) 7.57
B (dd) 7.13
C (dd) 6.49
D (q) 5.48
E (d) 5.39
F (t) 5.31
G (dd) 4.72
H (dd) 4.62
I (dd) 2.89
J (td) 2.01
1.00
1.01
1.02
1.00
0.96
0.99
1.02
1.97
1.00
1.04
f1 (ppm)

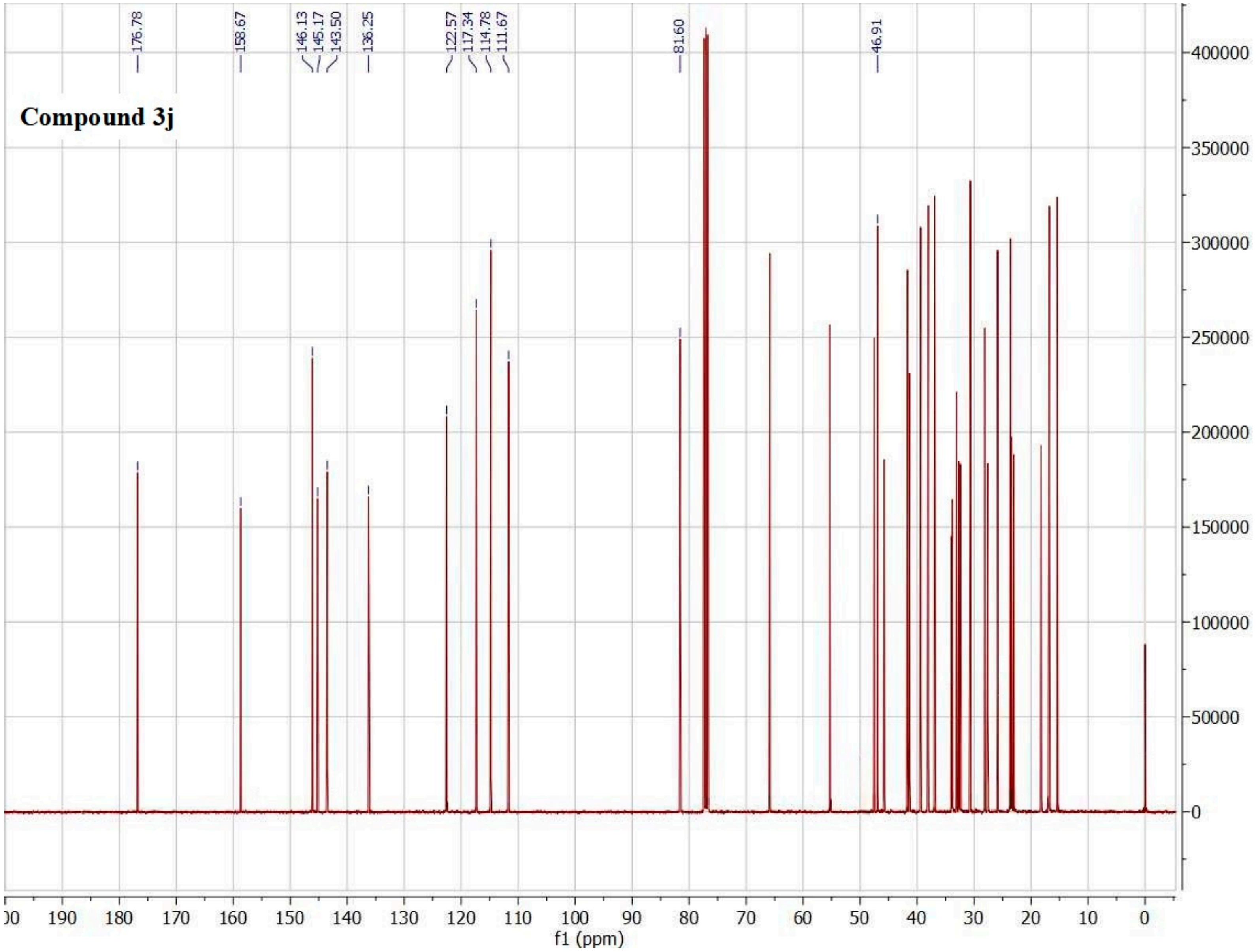

Compound 3j
176.78
158.67
146.13
145.17
143.50
136.25
122.57
117.34
114.78
111.67
81.60
46.91
f1 (ppm)

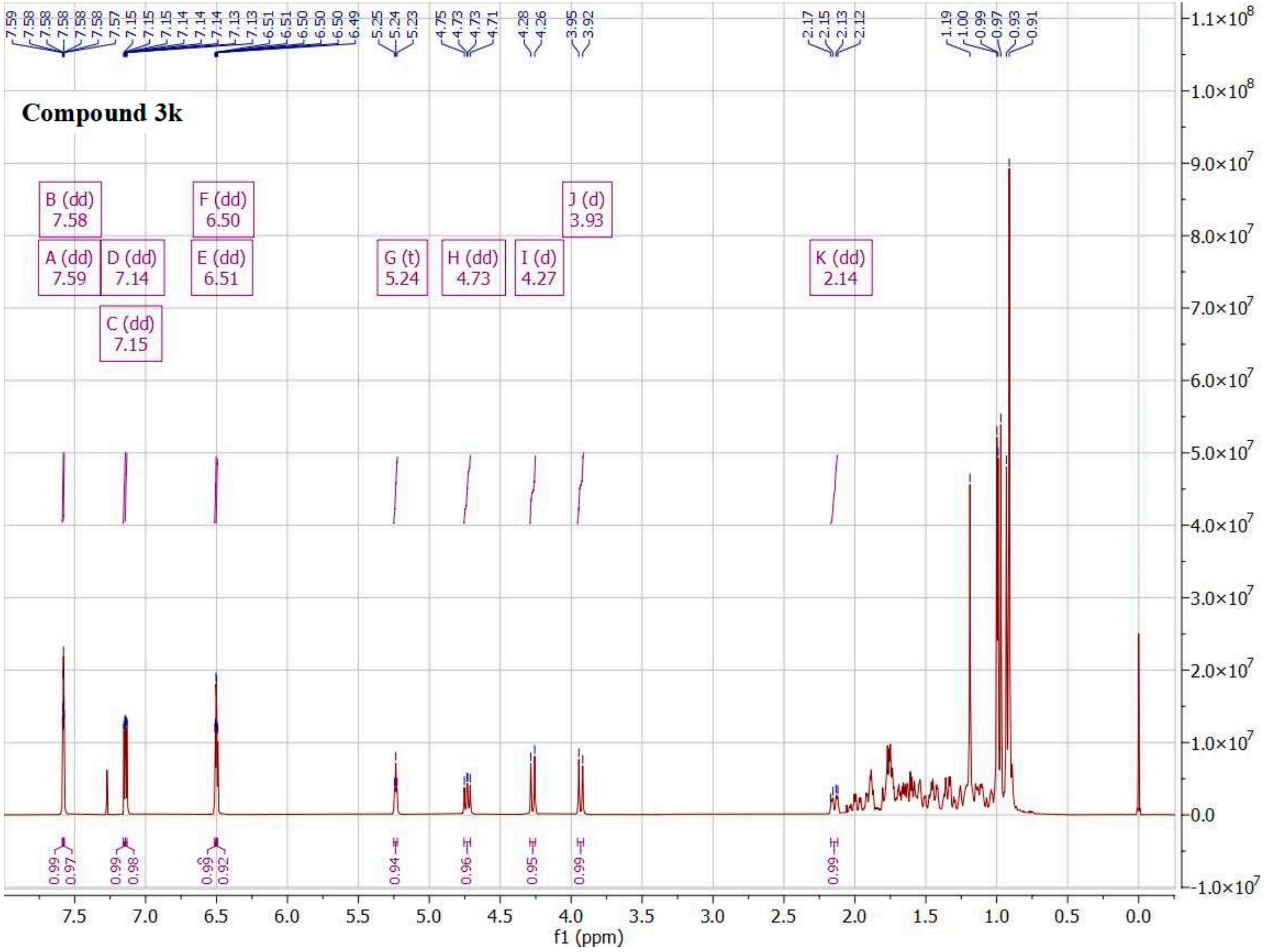
7.59 7.58 7.58 7.58 7.58 7.58 7.57 7.15 7.15 7.15 7.14 7.14 7.14 7.13 7.13 6.51 6.51 6.50 6.50 6.50 6.49
5.25 5.24 5.23
4.75 4.73 4.73 4.71
4.28 4.26
3.95 3.92
2.17 2.15 2.13 2.12
1.19 1.00 0.99 0.97 0.93 0.91
Compound 3k
B (dd) 7.58
A (dd) 7.59
D (dd) 7.14
C (dd) 7.15
F (dd) 6.50
E (dd) 6.51
G (t) 5.24
H (dd) 4.73
I (d) 4.27
J (d) 3.93
K (dd) 2.14
0.99 0.97 0.99 0.98 0.99 0.92 0.94 0.96 0.95 0.99 0.99
f1 (ppm)

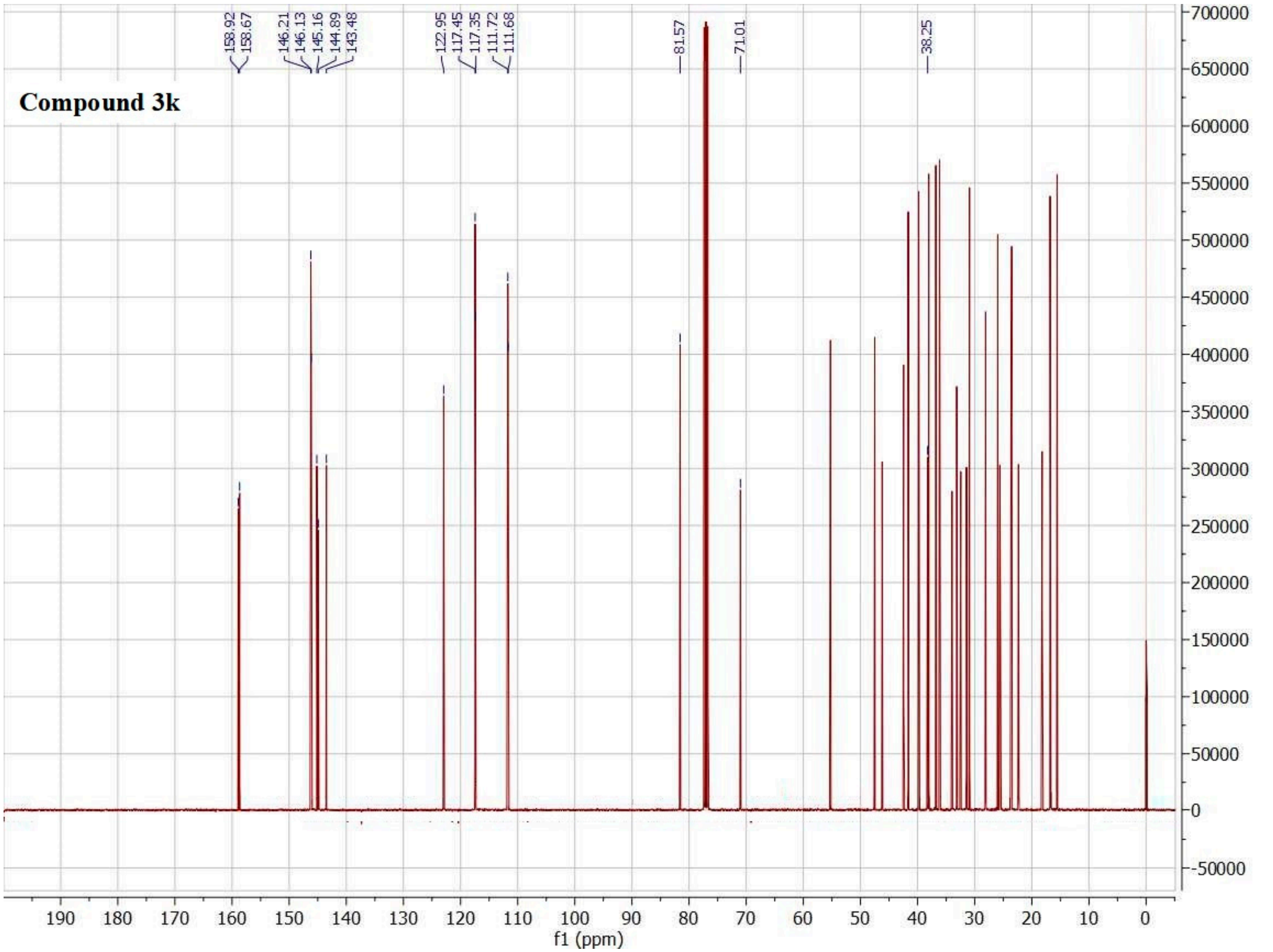
Compound 3k
158.92
158.67
146.21
146.13
145.16
144.89
143.48
122.95
117.45
117.35
111.72
111.68
81.57
71.01
38.25
f1 (ppm)

Article

Short-Chain Oleanolic Acid Esters and Furoyl Hybrids: Pharmacological Prediction, ADMETox Profiling, In Vitro Cytotoxicity Evaluation, Antioxidant Testing and EGFR Docking

Barbara Bednarczyk-Cwynar[1,2,*], Piotr Ruszkowski[3], Maciej Kulawik[4,5], Szymon Sip[4], Przemysław Zalewski[4], Dobrosława Wiśniewska[1], Andrzej Günther[1]

Supplementary Materials

Table S1. Predicted activity of oleanolic acid (**1**) and its derivatives **2a** – **2k** determined by the PASS method. **Legend: OA** = oleanolic acid; $\mathbf{P_a}$ = probability of activity; $\mathbf{P_i}$ = probability of inactivity; **AGPCh** = alkenylglycerophosphocholine, **AAGPh** = alkylacetylglycerophosphatase; **DAGOA**= diacylglycerol O-acyl, **PT** = protein-tyrosine, **T-17β-DHG** = testosterone 17beta-dehydrogenase; **values written in *italics*** = the alcohol part of the oleanolic acid ester is branched; **values underlined** = the alcohol part of the ester contains a multiple bond.

1.000 – 0.950	0.949 – 0.900	0.899 – 0.850	0.849 – 0.800	0.799 – 0.750	0.749 – 0.700	≤ 0.700

	activity	P_a factor (and P_i) factor) of compounds 1 and 2a – 2k (P_a ≤ 0.700)											
		OA (1)	2a	2b	2c	2d	2e	*2f*	2g	*2h*	*2i*	2j	2k
1	**α-Glucosidase inhibitor**	≤ 0.700	≤ 0.700	≤ 0.700	≤ 0.700	≤ 0.700	≤ 0.700	*≤ 0.700*	≤ 0.700	*≤ 0.700*	*≤ 0.700*	≤ 0.700	0.719 (0.001)
2	**Acylcarnitine hydrolase inhibitor**	0.711 (0.025)	≤ 0.700	≤ 0.700	≤ 0.700	≤ 0.700	≤ 0.700	*≤ 0.700*	≤ 0.700	*≤ 0.700*	*≤ 0.700*	≤ 0.700	≤ 0.700
3	**AGPCh hydrolase inhibitor**	0.825 (0.014)	≤ 0.700	0.724 (0.028)	0.763 (0.023)	≤ 0.700	≤ 0.700	*≤ 0.700*	0.836 (0.013)	*≤ 0.700*	*≤ 0.700*	≤ 0.700	0.823 (0.015)
4	**AAGPh inhibitor**	≤ 0.700	≤ 0.700	≤ 0.700	≤ 0.700	≤ 0.700	≤ 0.700	*≤ 0.700*	0.738 (0.016)	*≤ 0.700*	*≤ 0.700*	≤ 0.700	≤ 0.700
5	**Antiinflammatory**	0.819 (0.005)	0.809 (0.006)	0.802 (0.007)	0.799 (0.007)	0.775 (0.008)	0.753 (0.010)	*0.781 (0.008)*	0.797 (0.007)	*0.763 (0.009)*	*0.806 (0.006)*	≤ 0.700	0.769 (0.009)
6	**Antineoplastic**	0.876 (0.005)	0.867 (0.005)	0.834 (0.008)	0.811 (0.010)	0.844 (0.007)	0.854 (0.007)	*0.835 (0.008)*	0.808 (0.011)	*0.809 (0.011)*	*0.820 (0.009)*	0.794 (0.013)	0.920 (0.005)

7	**Antineoplastic (breast cancer)**	≤ 0.700	≤ 0.700	≤ 0.700	≤ 0.700	≤ 0.700	≤ 0.700	≤ 0.700	≤ 0.700	≤ 0.700	≤ 0.700	≤ 0.700	0.737 (0.005)
8	**Antineoplastic (colon cancer)**	≤ 0.700	≤ 0.700	≤ 0.700	≤ 0.700	≤ 0.700	≤ 0.700	≤ 0.700	≤ 0.700	≤ 0.700	≤ 0.700	≤ 0.700	0.734 (0.005)
9	**Antineoplastic (colorectal cancer)**	≤ 0.700	≤ 0.700	≤ 0.700	≤ 0.700	≤ 0.700	≤ 0.700	≤ 0.700	≤ 0.700	≤ 0.700	≤ 0.700	≤ 0.700	0.736 (0.005)
10	**Antineoplastic (lung cancer)**	0.766 (0.005)	0.761 (0.005)	0.732 (0.005)	0.713 (0.005)	0.720 (0.005)	≤ 0.700	0.734 (0.005)	≤ 0.700	0.726 (0.005)	≤ 0.700	≤ 0.700	0.802 (0.004)
11	**Antineoplastic (melanoma)**	≤ 0.700	≤ 0.700	≤ 0.700	≤ 0.700	≤ 0.700	≤ 0.700	≤ 0.700	≤ 0.700	≤ 0.700	≤ 0.700	≤ 0.700	0.822 (0.003)
12	**Antinociceptive**	≤ 0.700	0.848 (0.001)	0.829 (0.001)	0.810 (0.002)	0.819 (0.001)	0.759 (0.003)	0.813 (0.002)	0.810 (0.002)	0.808 (0.002)	0.794 (0.002)	≤ 0.700	0.832 (0.001)
13	**Antiprotozoal (Leishmania)**	0.721 (0.008)	0.762 (0.006)	0.765 (0.006)	0.765 (0.006)	0.819 (0.004)	≤ 0.700	≤ 0.700	0.822 (0.004)	≤ 0.700	≤ 0.700	≤ 0.700	≤ 0.700
14	**Antipruritic**	0.776 (0.004)	0.706 (0.008)	0.754 (0.005)	0.772 (0.004)	≤ 0.700	≤ 0.700	≤ 0.700	0.784 (0.004)	0.707 (0.008)	≤ 0.700	≤ 0.700	≤ 0.700
15	**Antisecretoric**	0.707 (0.009)	0.741 (0.007)	0.795 (0.004)	0.783 (0.005)	0.759 (0.005)	0.888 (0.003)	0.705 (0.009)	0.833 (0.004)	0.733 (0.007)	≤ 0.700	≤ 0.700	≤ 0.700
16	**Antiviral (Influenza)**	0.836 (0.002)	0.828 (0.003)	0.853 (0.002)	0.791 (0.003)	0.745 (0.004)	≤ 0.700	0.850 (0.002)	0.823 (0.003)	0.788 (0.003)	0.841 (0.002)	0.708 (0.005)	0.829 (0.003)
17	**Antiulcerative**	0.771 (0.004)	0.776 (0.004)	0.814 (0.004)	0.798 (0.004)	0.815 (0.004)	0.842 (0.003)	0.723 (0.005)	0.807 (0.004)	0.754 (0.004)	≤ 0.700	≤ 0.700	≤ 0.700
18	**Apoptosis agonist**	0.901 (0.004)	0.890 (0.004)	0.868 (0.005)	0.857 (0.005)	0.888 (0.005)	0.849 (0.005)	0.869 (0.005)	0.857 (0.005)	0.844 (0.005)	0.854 (0.005)	0.781 (0.009)	0.892 (0.004)
19	**Caspase 3 stimulant**	0.984 (0.002)	0.983 (0.002)	0.974 (0.002)	0.978 (0.002)	0.976 (0.002)	0.884 (0.004)	0.969 (0.002)	0.965 (0.002)	0.949 (0.003)	0.953 (0.003)	0.710 (0.010)	0.971 (0.002)
20	**Caspase 8 stimulant**	0.914 (0.001)	0.916 (0.001)	0.863 (0.001)	0.885 (0.001)	0.883 (0.001)	0.810 (0.002)	0.876 (0.001)	0.890 (0.001)	0.838 (0.001)	0.868 (0.001)	0.755 (0.003)	0.878 (0.001)
21	**Chemopreventive**	0.937 (0.002)	0.944 (0.002)	0.918 (0.002)	0.877 (0.003)	0.952 (0.002)	0.826 (0.003)	0.866 (0.003)	0.880 (0.003)	0.912 (0.002)	0.841 (0.003)	0.743 (0.005)	0.852 (0.003)
22	**Chitinase inhibitor**	0.789 (0.003)	0.706 (0.005)	0.712 (0.004)	0.725 (0.004)	0.772 (0.003)	≤ 0.700	≤ 0.700	0.765 (0.003)	≤ 0.700	≤ 0.700	≤ 0.700	0.730 (0.004)
23	**CYP2J substrate**	0.808 (0.019)	0.712 (0.046)	≤ 0.700	0.720 (0.044)	≤ 0.700	≤ 0.700	0.725 (0.042)	0.741 (0.037)	≤ 0.700	0.720 (0.044)	≤ 0.700	0.769 (0.029)
24	**Cytoprotectant**	0.709	0.715	0.733	≤ 0.700	0.722	≤ 0.700	≤ 0.700	0.723	≤ 0.700	≤ 0.700	≤ 0.700	≤ 0.700

		(0.004)	(0.004)	(0.004)		(0.004)			(0.004)				
25	**DAGOA transferase inhibitor**	≤ 0.700	≤ 0.700	0.831 (0.001)	0.775 (0.001)	≤ 0.700	≤ 0.700	*≤ 0.700*	0.851 (0.001)	*≤ 0.700*	*≤ 0.700*	≤ 0.700	≤ 0.700
26	**DNA ligase (ATP) inhibitor**	0.803 (0.001)	0.833 (0.001)	0.731 (0.001)	0.725 (0.001)	0.701 (0.002)	≤ 0.700	*0.722 (0.001)*	0.727 (0.001)	*≤ 0.700*	*≤ 0.700*	≤ 0.700	0.769 (0.001)
27	**Gastrin inhibitor**	≤ 0.700	≤ 0.700	≤ 0.700	≤ 0.700	≤ 0.700	≤ 0.700	*≤ 0.700*	0.719 (0.004)	*≤ 0.700*	*≤ 0.700*	≤ 0.700	≤ 0.700
28	**Hepatic disorders treatment**	0.834 (0.004)	0.835 (0.004)	0.823 (0.004)	0.799 (0.004)	0.798 (0.004)	0.751 (0.004)	*0.865 (0.003)*	0.802 (0.004)	*0.816 (0.004)*	*0.818 (0.004)*	≤ 0.700	0.806 (0.004)
29	**Hepatoprotectant**	0.930 (0.002)	0.969 (0.001)	0.965 (0.001)	0.948 (0.002)	0.956 (0.001)	0.915 (0.002)	*0.981 (0.001)*	0.967 (0.001)	*0.968 (0.001)*	*0.958 (0.001)*	0.803 (0.004)	0.906 (0.002)
30	**Hypolipemic**	0.713 (0.013)	0.799 (0.007)	0.858 (0.005)	0.836 (0.006)	0.825 (0.006)	0.715 (0.012)	*0.859 (0.005)*	0.877 (0.004)	*0.840 (0.005)*	*0.894 (0.004)*	≤ 0.700	0.829 (0.006)
31	**ICAM1 expression inhibitor**	0.747 (0.001)	≤ 0.700	0.712 (0.002)	≤ 0.700	≤ 0.700	≤ 0.700	*≤ 0.700*	≤ 0.700	*≤ 0.700*	*≤ 0.700*	≤ 0.700	0.810 (0.001)
32	**Insulin promotor**	0.869 (0.004)	0.978 (0.001)	0.982 (0.001)	0.975 (0.001)	0.950 (0.002)	0.942 (0.002)	*0.977 (0.001)*	0.970 (0.001)	*0.977 (0.001)*	*0.973 (0.001)*	0.930 (0.002)	0.970 (0.001)
33	**Lipid metabolism regulator**	≤ 0.700	0.833 (0.005)	0.928 (0.003)	0.884 (0.004)	0.960 (0.002)	≤ 0.700	*0.923 (0.003)*	0.922 (0.003)	*0.890 (0.004)*	*0.854 (0.004)*	≤ 0.700	0.815 (0.005)
34	**Lipid peroxidase inhibitor**	0.810 (0.003)	0.755 (0.004)	0.774 (0.004)	0.763 (0.004)	≤ 0.700	≤ 0.700	*0.830 (0.003)*	0.784 (0.004)	*≤ 0.700*	*0.742 (0.004)*	≤ 0.700	0.752 (0.004)
35	**Membrane integrity antagonist**	0.928 (0.002)	0.914 (0.002)	0.930 (0.002)	0.928 (0.002)	0.874 (0.004)	0.874 (0.004)	*0.910 (0.002)*	0.941 (0.001)	*0.911 (0.002)*	*0.909 (0.002)*	0.817 (0.006)	0.898 (0.003)
36	**Mucomembranous protectant**	0.894 (0.005)	0.807 (0.017)	0.804 (0.017)	0.808 (0.016)	0.872 (0.006)	≤ 0.700	*0.783 (0.023)*	0.815 (0.015)	*0.808 (0.016)*	*0.758 (0.032)*	≤ 0.700	0.824 (0.013)
37	**Nitric oxide antagonist**	0.814 (0.002)	0.794 (0.003)	0.773 (0.003)	0.738 (0.003)	0.761 (0.003)	0.746 (0.003)	*0.798 (0.003)*	0.734 (0.003)	*0.732 (0.003)*	*0.755 (0.003)*	≤ 0.700	0.784 (0.003)
38	**Oxidoreductase inhibitor**	0.904 (0.002)	0.879 (0.003)	0.912 (0.002)	0.892 (0.002)	0.924 (0.001)	0.851 (0.004)	*0.868 (0.003)*	0.901 (0.002)	*0.896 (0.002)*	*0.859 (0.004)*	0.878 (0.003)	0.888 (0.003)
39	**Phosphatase inhibitor**	0.894 (0.001)	0.730 (0.008)	0.731 (0.008)	0.724 (0.009)	0.724 (0.009)	0.712 (0.011)	*0.734 (0.008)*	0.718 (0.010)	*0.720 (0.009)*	*0.721 (0.009)*	≤ 0.700	0.755 (0.005)
40	**Phospholipase C inhibitor**	≤ 0.700	≤ 0.700	≤ 0.700	0.745 (0.002)	0.734 (0.002)	≤ 0.700	*≤ 0.700*	0.888 (0.001)	*≤ 0.700*	*≤ 0.700*	≤ 0.700	≤ 0.700
41	**Protein phosphatase inhibitor**	0.782 (0.002)	0.705 (0.003)	≤ 0.700	≤ 0.700	≤ 0.700	≤ 0.700	*0.707 (0.003)*	≤ 0.700	*≤ 0.700*	*≤ 0.700*	≤ 0.700	0.718 (0.003)

42	**PT phosphatase inhibitor**	0.764 (0.002)	≤ 0.700	≤ 0.700	≤ 0.700	≤ 0.700	≤ 0.700	*≤ 0.700*	≤ 0.700	*≤ 0.700*	*≤ 0.700*	≤ 0.700	≤ 0.700
43	**T-cell PT phosphatase inhibitor**	0.726 (0.001)	≤ 0.700	≤ 0.700	≤ 0.700	≤ 0.700	≤ 0.700	*≤ 0.700*	≤ 0.700	*≤ 0.700*	*≤ 0.700*	≤ 0.700	≤ 0.700
44	**T-17β-DHG (NADP+) inhibitor**	0.892 (0.007)	0.775 (0.032)	0.754 (0.038)	0.736 (0.043)	≤ 0.700	≤ 0.700	*0.780 (0.031)*	0.705 (0.053)	*0.796 (0.026)*	*0.736 (0.043)*	≤ 0.700	0.795 (0.027)
45	**TF stimulant**	0.954 (0.001)	0.934 (0.001)	0.930 (0.001)	0.925 (0.001)	0.931 (0.001)	0.918 (0.001)	*0.935 (0.001)*	0.922 (0.001)	*0.925 (0.001)*	*0.926 (0.001)*	0.903 (0.001)	0.931 (0.001)
46	**Transcription factor NF κB inhibitor**	≤ 0.700	≤ 0.700	0.746 (0.002)	0.711 (0.002)	0.738 (0.002)	≤ 0.700	*≤ 0.700*	0.701 (0.002)	*≤ 0.700*	*≤ 0.700*	≤ 0.700	≤ 0.700
47	**Transcription factor NF κB stimulant**	0.954 (0.001)	0.934 (0.001)	0.930 (0.001)	0.925 (0.001)	0.931 (0.001)	0.918 (0.001)	*0.935 (0.001)*	0.922 (0.001)	*0.925 (0.001)*	*0.926 (0.001)*	0.903 (0.001)	0.931 (0.001)
48	**Vasodilator. peripheral**	≤ 0.700	≤ 0.700	≤ 0.700	0.715 (0.008)	≤ 0.700	≤ 0.700	*≤ 0.700*	≤ 0.700	*≤ 0.700*	*≤ 0.700*	≤ 0.700	≤ 0.700
49	**Wound healing agent**	≤ 0.700	≤ 0.700	≤ 0.700	≤ 0.700	0.720 (0.004)	≤ 0.700	*≤ 0.700*	≤ 0.700	*≤ 0.700*	*≤ 0.700*	≤ 0.700	≤ 0.700

Table S2. Predicted activity of oleanolic acid (**1**) and its acylated esters **3a** – **3k** determined by the PASS method. **Legend: P_a** = probability of activity; **P_i** = probability of inactivity; **AGPCh** – alkenylglycerophosphocholine, **DAGOA**= diacylglycerol O-acyl, **PT** = protein-tyrosine, **T-17β-dehydr.** = testosterone 17beta-dehydrogenase; **values written in *italics*** = the alcohol part of the oleanolic acid ester is branched; **values underlined** = the alcohol part of the ester contains a multiple bond.

1.000 – 0.950	0.949 – 0.900	0.899 – 0.850	0.849 – 0.800	0.799 – 0.750	0.749 – 0.700	≤ 0.700

	activity	P_a factor (and P_i) factor) of compounds 1 and 3a – 3k (P_a ≤ 0.700)											
		**OA (1)**	**3a**	**3b**	**3c**	**3d**	**3e**	***3f***	**3g**	***3h***	***3i***	**3j**	**3k**
**1**	**Acylcarnitine hydrolase inhibitor**	0.711 (0.025)	≤ 0.700	≤ 0.700	≤ 0.700	≤ 0.700	≤ 0.700	*≤ 0.700*	≤ 0.700	*≤ 0.700*	*≤ 0.700*	≤ 0.700	≤ 700
**2**	**AGPCh hydrolase inhibitor**	0.825 (0.014)	≤ 0.700	≤ 0.700	≤ 0.700	≤ 0.700	≤ 0.700	*≤ 0.700*	≤ 0.700	*≤ 0.700*	*≤ 0.700*	≤ 0.700	≤ 700
**3**	**Antiinflammatory**	0.819 (0.005)	0.802 (0.007)	0.796 (0.007)	0.793 (0.007)	0.771 (0.009)	0.751 (0.010)	*0.776 (0.008)*	0.791 (0.007)	*0.760 (0.009)*	*0.801 (0.007)*	≤ 0.700	0.767 (0.009)
**4**	**Antineoplastic**	0.876 (0.005)	0.801 (0.012)	0.764 (0.016)	0.733 (0.021)	0.781 (0.014)	0.792 (0.013)	*0.764 (0.016)*	0.730 (0.021)	*0.730 (0.021)*	*0.747 (0.019)*	0.718 (0.023)	0.810 (0.010)
**5**	**Antineoplastic (lung cancer)**	0.766 (0.005)	0.723 (0.005)	≤ 0.700	≤ 0.700	≤ 0.700	≤ 0.700	*≤ 0.700*	≤ 0.700	*≤ 0.700*	*≤ 0.700*	≤ 0.700	0.716 (0.005)
**6**	**Antinociceptive**	≤ 0.700	0.756 (0.003)	0.733 (0.003)	0.705 (0.004)	0.718 (0.003)	≤ 0.700	*0.709 (0.004)*	0.704 (0.004)	*≤ 0.700*	*≤ 0.700*	≤ 0.700	0.733 (0.003)
**7**	**Antiprotozoal (Leishmania)**	0.721 (0.008)	≤ 0.700	≤ 0.700	≤ 0.700	0.743 (0.007)	≤ 0.700	*≤ 0.700*	0.746 (0.007)	*≤ 0.700*	*≤ 0.700*	≤ 0.700	≤ 700
**8**	**Antipruritic**	0.776 (0.004)	≤ 0.700	0.741 (0.005)	0.761 (0.005)	≤ 0.700	≤ 0.700	*≤ 0.700*	0.773 (0.004)	*≤ 0.700*	*≤ 0.700*	≤ 0.700	≤ 700
**9**	**Antisecretoric**	0.707 (0.009)	≤ 0.700	0.709 (0.009)	≤ 0.700	≤ 0.700	0.820 (0.004)	*≤ 0.700*	0.747 (0.006)	*≤ 0.700*	*≤ 0.700*	≤ 0.700	≤ 700
**10**	**Antiviral (Influenza)**	0.836 (0.002)	≤ 0.700	≤ 0.700	≤ 0.700	≤ 0.700	≤ 0.700	*≤ 0.700*	≤ 0.700	*≤ 0.700*	*≤ 0.700*	≤ 0.700	≤ 700
**11**	**Antiulcerative**	0.771 (0.004)	0.743 (0.004)	0.781 (0.004)	0.765 (0.004)	0.782 (0.004)	0.820 (0.004)	*≤ 0.700*	0.774 (0.004)	*0.714 (0.005)*	*≤ 0.700*	≤ 0.700	≤ 700
**12**	**Apoptosis agonist**	0.901 (0.004)	0.849 (0.005)	0.818 (0.007)	0.804 (0.008)	0.852 (0.005)	0.793 (0.008)	*0.819 (0.007)*	0.804 (0.008)	*0.785 (0.009)*	*0.799 (0.008)*	0.706 (0.014)	0.812 (0.007)
**13**	**Caspase 3 stimulant**	0.984 (0.002)	0.850 (0.004)	0.784 (0.006)	0.806 (0.005)	0.796 (0.006)	≤ 0.700	*0.758 (0.008)*	0.744 (0.009)	*≤ 0.700*	*0.705 (0.011)*	≤ 0.700	≤ 700

14	**Caspase 8 stimulant**	0.914 (0.001)	0.830 (0.001)	0.766 (0.002)	0.794 (0.002)	0.792 (0.002)	≤ 0.700	0.783 (0.002)	0.800 (0.002)	0.730 (0.003)	0.772 (0.002)	≤ 0.700	0.755 (0.003)
15	**Chemopreventive**	0.937 (0.002)	0.956 (0.001)	0.941 (0.002)	0.912 (0.002)	0.962 (0.001)	0.856 (0.003)	0.904 (0.002)	0.915 (0.002)	0.937 (0.002)	0.874 (0.003)	0.786 (0.004)	0.880 (0.003)
16	**Chitinase inhibitor**	0.789 (0.003)	≤ 0.700	≤ 0.700	≤ 0.700	≤ 0.700	≤ 0.700	≤ 0.700	≤ 0.700	≤ 0.700	≤ 0.700	≤ 0.700	≤ 700
17	**CYP2J substrate**	0.808 (0.019)	≤ 0.700	≤ 0.700	≤ 0.700	≤ 0.700	≤ 0.700	≤ 0.700	≤ 0.700	≤ 0.700	≤ 0.700	≤ 0.700	≤ 700
18	**Cytoprotectant**	0.709 (0.004)	≤ 0.700	≤ 0.700	≤ 0.700	≤ 0.700	≤ 0.700	≤ 0.700	≤ 0.700	≤ 0.700	≤ 0.700	≤ 0.700	≤ 700
19	**DNA ligase (ATP) inhibitor**	0.803 (0.001)	≤ 0.700	≤ 0.700	≤ 0.700	≤ 0.700	≤ 0.700	≤ 0.700	≤ 0.700	≤ 0.700	≤ 0.700	≤ 0.700	≤ 700
20	**Hepatic disorders treatment**	0.834 (0.004)	0.773 (0.004)	0.754 (0.004)	0.722 (0.004)	0.722 (0.004)	≤ 0.700	0.819 (0.004)	0.726 (0.004)	0.744 (0.004)	0.748 (0.004)	≤ 0.700	0.741 (0.004)
21	**Hepatoprotectant**	0.930 (0.002)	0.896 (0.003)	0.891 (0.003)	0.866 (0.003)	0.878 (0.003)	0.801 (0.004)	0.919 (0.002)	0.893 (0.003)	0.895 (0.003)	0.880 (0.003)	≤ 0.700	0.825 (0.004)
22	**Hypolipemic**	0.713 (0.013)	0.740 (0.011)	0.815 (0.007)	0.781 (0.008)	0.767 (0.009)	≤ 0.700	0.817 (0.007)	0.840 (0.005)	0.786 (0.008)	0.859 (0.005)	≤ 0.700	0.705 (0.013)
23	**ICAM1 expression inhibitor**	0.747 (0.001)	≤ 0.700	≤ 0.700	≤ 0.700	≤ 0.700	≤ 0.700	≤ 0.700	≤ 0.700	≤ 0.700	≤ 0.700	≤ 0.700	≤ 700
24	**Insulin promotor**	0.869 (0.004)	0.839 (0.004)	0.873 (0.003)	0.805 (0.004)	≤ 0.700	≤ 0.700	0.823 (0.004)	0.764 (0.004)	0.828 (0.004)	0.791 (0.004)	≤ 0.700	0.713 (0.005)
25	**Lipid metabolism regulator**	≤ 0.700	≤ 0.700	0.794 (0.006)	0.704 (0.011)	0.890 (0.004)	≤ 0.700	0.784 (0.006)	0.781 (0.006)	0.714 (0.010)	≤ 0.700	≤ 0.700	≤ 700
26	**Lipid peroxidase inhibitor**	0.810 (0.003)	≤ 0.700	≤ 0.700	≤ 0.700	≤ 0.700	≤ 0.700	≤ 0.700	≤ 0.700	≤ 0.700	≤ 0.700	≤ 0.700	≤ 700
27	**Membrane integrity antagonist**	0.928 (0.002)	0.773 (0.009)	0.849 (0.004)	0.841 (0.005)	≤ 0.700	≤ 0.700	0.753 (0.010)	0.880 (0.003)	0.759 (0.010)	0.746 (0.011)	≤ 0.700	≤ 700
28	**Mucomemranous protectant**	0.894 (0.005)	≤ 0.700	≤ 0.700	≤ 0.700	≤ 0.700	≤ 0.700	≤ 0.700	≤ 0.700	≤ 0.700	≤ 0.700	≤ 0.700	≤ 700
29	**NF-E2-related factor 2 stimulant**	≤ 0.700	0.789 (0.001)	≤ 0.700	≤ 0.700	≤ 0.700	≤ 0.700	≤ 0.700	≤ 0.700	≤ 0.700	≤ 0.700	≤ 0.700	≤ 700
30	**Nitric oxide antagonist**	0.814 (0.002)	0.746 (0.003)	0.724 (0.003)	≤ 0.700	0.715 (0.003)	0.702 (0.003)	0.754 (0.003)	≤ 0.700	≤ 0.700	0.709 (0.003)	≤ 0.700	≤ 700
31	**Oxidoreductase inhibitor**	0.904	0.772	0.860	0.816	0.876	≤ 0.700	0.731	0.841	0.829	≤ 0.700	0.768	0.779

		(0.002)	(0.008)	(0.004)	(0.005)	(0.003)		(0.012)	(0.004)	(0.005)		(0.009)	(0.008)
**32**	**Phospholipase C inhibitor**	≤ 0.700	≤ 0.700	≤ 0.700	0.707<br>(0.012)	≤ 0.700	≤ 0.700	≤ 0.700	≤ 0.700	≤ 0.700	≤ 0.700	≤ 0.700	≤ 700
**33**	**Phosphatase inhibitor**	0.894<br>(0.001)	0.711<br>(0.011)	0.714<br>(0.010)	≤ 0.700	0.707<br>(0.012)	≤ 0.700	0.716<br>(0.010)	0.702<br>(0.013)	0.703<br>(0.012)	0.704<br>(0.012)	≤ 0.700	0.721<br>(0.009)
**34**	**Protein phosphatase inhibitor**	0.782<br>(0.002)	≤ 0.700	≤ 0.700	≤ 0.700	≤ 0.700	≤ 0.700	≤ 0.700	≤ 0.700	≤ 0.700	≤ 0.700	≤ 0.700	≤ 700
**35**	**PT phosphatase inhibitor**	0.764<br>(0.002)	≤ 0.700	≤ 0.700	≤ 0.700	≤ 0.700	≤ 0.700	≤ 0.700	≤ 0.700	≤ 0.700	≤ 0.700	≤ 0.700	≤ 700
**36**	**T-cell PT phosphatase inhibitor**	0.726<br>(0.001)	≤ 0.700	≤ 0.700	≤ 0.700	≤ 0.700	≤ 0.700	≤ 0.700	≤ 0.700	≤ 0.700	≤ 0.700	≤ 0.700	≤ 700
**37**	**T-17β-dehydr. (NADP+) inhibitor**	0.892<br>(0.007)	≤ 0.700	≤ 0.700	≤ 0.700	≤ 0.700	≤ 0.700	≤ 0.700	≤ 0.700	≤ 0.700	≤ 0.700	≤ 0.700	≤ 700
**38**	**Transcription factor NF kappa B stimulant**	0.954<br>(0.001)	0.892<br>(0.002)	0.886<br>(0.002)	0.879<br>(0.002)	0.891<br>(0.002)	0.869<br>(0.002)	≤ 0.700	0.876<br>(0.002)	0.880<br>(0.002)	0.882<br>(0.002)	0.848<br>(0.002)	0.860<br>(0.002)
**39**	**Transcription factor stimulant**	0.954<br>(0.001)	0.892<br>(0.002)	0.886<br>(0.002)	0.879<br>(0.002)	0.891<br>(0.002)	0.869<br>(0.002)	0.894<br>(0.002)	0.876<br>(0.002)	0.880<br>(0.002)	0.882<br>(0.002)	0.848<br>(0.002)	0.860<br>(0.002)

Article

Short-Chain Oleanolic Acid Esters and Furoyl Hybrids: Pharmacological Prediction, ADMETox Profiling, In Vitro Cytotoxicity Evaluation, Antioxidant Testing and EGFR Docking

Barbara Bednarczyk-Cwynar[1,2,*], Piotr Ruszkowski[3], Maciej Kulawik[4,5], Szymon Sip[4], Przemysław Zalewski[4], Dobrosława Wiśniewska[1], Andrzej Günther[1]

Supplementary Materials

Table S3. ADMETox data for oleanolic acid (**1**) and its derivatives **2a – 2k**. **Legend: For values given in a range 0.000 – 1.000** (unless stated otherwise)**: 0.000 – 0.300** = low probability; **0.301 – 0.699** = moderate probability; **0.700 – 1.000** = high probability; ***** = alerts; **Optimal values: Physicochemical Properties: Molecular Weight**: 100 ~ 600; **nHA**: 0 ~ 12; **nHD**: 0 ~ 7; **nRot**: 0 ~ 11; **nRing**: 0 ~ 6; **MaxRing**: 0 ~ 18; **nHet**: 1 ~ 15; **fChar**: -4 ~ 4; **nRig**: 0 ~ 30; **Stereo Centers**: ≤ 2; **TPSA**: 0 ~ 140; **logS**: -4 ~ 0.5; **logP**: 0 ~ 3; **logD**: 1 ~ 3. **Medicinal Chemistry: QED**: > 0.67; easy to synthesize; **Fsp**^{3}: ≥ 0.42; **MCE-18**: ≥ 4; **Npscore**: -5 ~ 5; **ALARM NMR**: 0 **BMS**: 0; **Chelator Rule**: 0; **PAINS**: 0; **Lipinski Rule**: 1. **Pfizer Rule**: 1. **GSK Rule**: 1. **Golden Triangle**: 1. **Absorption: Caco-2 Permeability**: > -5.15;. **MDCK Permeability**: high passive permeability: > 20 × 10^{-6} cm/s; **Pgp-inhibitor**: > 0.700; **Pgp-substrate**: > 0.700; **HIA**: > 0.700; **F**$_{20\%}$: > 0.700; **F**$_{30\%}$: > 0.700. **Distribution: BBB Penetr.**: > 0.700; **PPB: < 90%; VD: 0.04 – 20 L/kg; Fu: high: > 20%. Metabolism: CYP1A2 inhibitor, CYP1A2 substrate, etc.:** > 0.700. **Excretion**: **CL**: >15 mL/min/kg; **T**$_{1/2}$ long half-life >3h: > 0.700. **Toxicity: hERG Blockers, H-HT, etc: <** 0.300. **Environmental toxicity**: BCF, IG$_{50}$, etc: informative values. **Tox21 pathway: NR-AhR, NR-AR**, etc.: < 0.300. **Toxicophore Rules**: Non Biodeg. Rule, etc.: 0. **Explanation of parameters**: https://admetmesh.scbdd.com/explanation/index

High value (optimal)	Moderate value	Low value	Neutral value

Physicochemical Properties (optimal values)	Compound number											
	1 (OA)	2a	2b	2c	2d	2e	*2f*	2g	*2h*	*2i*	2j	2k
**Molecular Weight**	456.36	470.38	484.39	498.41	496.39	494.38	*498.41*	512.42	*512.42*	*512.42*	566.33	442.38
**Volume**	505.750	523.047	540.343	557.639	555.002	552.366	*557.639*	574.935	*574.935*	*574.935*	588.061	499.598

**Density**	0.902	0.899	0.896	0.894	0.894	0.895	0.894	0.891	0.891	0.891	0.963	0.885
**NHA**	3	3	3	3	3	3	3	3	3	3	3	2
**nHD**	2	1	1	1	1	1	1	1	1	1	1	1
**nRot**	1	2	3	4	4	3	3	5	4	4	5	1
**nRing**	5	5	5	5	5	5	5	5	5	5	5	5
**maxRing**	22	22	22	22	22	22	22	22	22	22	22	22
**nHet**	3	3	3	3	3	3	3	3	3	3	5	2
**fChar**	0	0	0	0	0	0	0	0	0	0	0	0
**nRig**	27	27	27	27	28	28	27	27	27	27	27	26
**Flexibility**	0.037	0.074	0.111	0.148	0.143	0.107	0.111	0.185	0.184	0.148	0.185	0.038
**Stereo Centers**	8	8	8	8	8	8	8	8	8	9	9	8
**TPSA**	57.530	46.530	46.530	46.530	46.530	46.530	46.530	46.530	46.530	46.530	46.530	40.460
**LogS**	-4.933	-6.212	-6.493	-6.714	-6.501	-6.582	-6.523	-6.829	-6.346	-6.778	-6.279	-5.769
**LogD**	4.781	4.227	5.179	5.278	5.105	5.162	5.361	5.437	5.501	5.378	5.288	4.705
**LogP**	6.370	5.149	6.937	7.285	7.238	6.593	7.104	7.704	7.574	7.522	7.244	6.538
**QED**	0.409	0.322	0.322	0.316	0.319	0.248	0.309	0.233	0.305	0.304	0.210	0.424
**SAscore**	4.589	4.608	4.605	4.647	4.736	4.812	4.707	4.636	4.687	4.822	4.990	4.702
**Fsp3**	0.900	0.903	0.906	0.909	0.848	0.848	0.909	0.912	0.912	0.912	0.909	0.933
**MCE-18**	105.368	104.441	103.574	102.762	103.574	103.574	105.397	102.000	104.615	104.615	105.397	102.207
**Npscore**	3.272	3.175	2.983	2.932	2.969	2.780	2.830	2.896	2.903	2.783	2.926	3.326
**ALARM NMR***	0	0	0	0	0	0	0	0	0	0	0	0
**BMS***	0	0	0	0	0	0	0	0	0	0	2	q
**Chelator Rule***	0	0	0	0	0	0	0	0	0	0	0	0
**PAINS***	0	0	0	0	0	0	0	0	0	0	0	0
**Lipinski Rule**	1	0	0	0	0	0	0	1	1	1	1	0
**Pfizer Rule**	0	1	1	1	1	1	1	1	1	1	1	1
**GSK Rule**	0	1	1	1	1	1	1	1	1	1	1	1
**Golden Triangle**	1	0	0	0	0	0	0	1	1	1	1	0
**Caco-2 Permeability**	-5.216	-4.963	-4.959	-4.963	-4.922	-4.973	-4.940	-4.979	-5.011	-4.926	-5.081	-4.867
**MDCK Permeability**	1.2×10^{-5}	1.2×10^{-5}	1.2×10^{-5}	1.1×10^{-5}	1.3×10^{-5}	1.2×10^{-5}	1.3×10^{-5}	1.0×10^{-5}	1.1×10^{-5}	1.1×10^{-5}	9.8×10^{-6}	8.8×10^{-6}
**Pgp Inhibitor**	0.000	0.095	0.068	0.029	0.042	0.079	0.074	0.012	0.053	0.071	0.048	0.002
**Pgp Substrate**	0.000	0.000	0.000	0.000	0.000	0.000	0.000	0.000	0.000	0.000	0.000	0.000
**HIA**	0.016	0.016	0.016	0.017	0.014	0.038	0.012	0.018	0.065	0.011	0.039	0.011
**F20%**	0.125	0.938	0.850	0.953	0.408	0.095	0.780	0.977	0.920	0.863	0.030	0.878
**F30%**	0.874	0.903	0.859	0.938	0.437	0.851	0.668	0.958	0.870	0.847	0.732	0.952

BBB Penetration	0.577	0.368	0.334	0.352	0.368	0.551	0.361	0.340	0.325	0.382	0.685	0.562
PPB	96.976	97.028	98.103	98.371	97.275	97.837	98.545	98.673	98.616	98.609	99.990	98.236
VDss	0.707	1.004	1.069	1.007	0.960	1.109	1.233	1.058	1.082	1.242	1.293	1.194
Fu	4.389	3.653	2.919	2.613	4.169	1.047	3.047	2.430	2.516	2.909	2.099	2.365
CYP1A2 Inhibitor	0.014	0.033	0.037	0.034	0.035	0.048	0.028	0.034	0.021	0.025	0.034	0.038
CYP1A2 Substrate	0.307	0.373	0.266	0.225	0.212	0.403	0.170	0.199	0.167	0.164	0.255	0.194
CYP2C19 Inhibitor	0.026	0.073	0.084	0.084	0.072	0.131	0.089	0.092	0.074	0.075	0.075	0.054
CYP2C19 Substrate	0.901	0.944	0.939	0.942	0.930	0.919	0.942	0.941	0.948	0.953	0.946	0.924
CYP2C9 Inhibitor	0.179	0.215	0.210	0.192	0.220	0.361	0.153	0.169	0.137	0.169	0.179	0.187
CYP2C9 Substrate	0.758	0.254	0.157	0.215	0.303	0.189	0.133	0.236	0.186	0.171	0.263	0.124
CYP2D6 Inhibitor	0.012	0.068	0.144	0.117	0.211	0.426	0.070	0.179	0.048	0.093	0.266	0.049
CYP2D6 Substrate	0.256	0.390	0.223	0.226	0.584	0.139	0.063	0.164	0.113	0.165	0.5816	0.245
CYP3A4 Inhibitor	0.242	0.692	0.621	0.649	0.774	0.800	0.434	0.653	0.435	0.479	0.737	0.717
CYP3A4 Substrate	0.227	0.577	0.583	0.524	0.655	0.719	0.557	0.505	0.571	0.690	0.698	0.455
CLplasma	3.813	10.512	11.094	11.328	11.584	7.605	11.224	10.870	11.439	10.550	10.129	10.509
T1/2	0.021	0.011	0.007	0.006	0.006	0.008	0.008	0.006	0.006	0.006	0.008	0.017
hERG Blockers	0.006	0.030	0.037	0.041	0.026	0.003	0.040	0.058	0.032	0.026	0.033	0.021
DILI	0.011	0.014	0.012	0.012	0.012	0.269	0.014	0.012	0.013	0.015	0.015	0.007
AMES Toxicity	0.006	0.005	0.006	0.007	0.011	0.018	0.006	0.006	0.008	0.003	0.025	0.003
ROA Toxicity	0.264	0.378	0.318	0.301	0.397	0.683	0.354	0.266	0.308	0.308	0.256	0.162
FDAMDD	0.914	0.921	0.913	0.918	0.937	0.954	0.895	0.916	0.911	0.929	0.914	0.925
Skin Sensitivity	0.046	0.050	0.084	0.110	0.169	0.111	0.071	0.146	0.133	0.084	0.070	0.076
Carcinogenecity	0.095	0.069	0.069	0.059	0.245	0.328	0.096	0.057	0.073	0.087	0.272	0.067
Eye Corrosion	0.030	0.006	0.004	0.004	0.004	0.004	0.006	0.004	0.004	0.005	0.004	0.006
Eye Irritation	0.066	0.022	0.039	0.046	0.058	0.040	0.021	0.046	0.069	0.018	0.027	0.022
Respiratory Toxicity	0.974	0.974	0.973	0.968	0.940	0.969	0.974	0.966	0.964	0.971	0.954	0.980
H-HT	0.346	0.360	0.240	0.253	0.148	0.269	0.283	0.254	0.233	0.299	0.275	0.402
BCF	1.815	2.868	2.866	2.418	2.952	2.549	3.003	2.177	2.632	2.809	2.647	3.075
IGC_{50}	4.971	5.170	5.246	5.318	5.232	5.220	5.209	5.431	5.371	5.247	5.399	5.176
$LC_{50}DM$	6.345	6.174	6.797	6.790	6.799	6.948	6.695	6.792	6.751	6.812	6.851	6.676
$LC_{50}FM$	5.884	6.144	6.339	6.357	6.726	6.648	6.090	6.446	6.444	6.191	6.537	6.126
NR-AhR	0.001	0.000	0.000	0.000	0.000	0.001	0.000	0.000	0.000	0.000	0.000	0.000
NR-AR	0.369	0.460	0.039	0.035	0.057	0.032	0.035	0.030	0.026	0.160	0.029	0.055
NR-AR-LBD	0.479	0.739	0.673	0.726	0.839	0.833	0.435	0.678	0.289	0.750	0.713	0.355
NR-Aromatase	0.798	0.630	0.637	0.604	0.548	0.637	0.610	0.575	0.545	0.575	0.674	0.702

**NR-ER**	0.672	0.536	0.490	0.749	0.747	0.818	*0.515*	0.714	*0.619*	*0.557*	0.763	0.249
**NR-ER-LBD**	0.746	0.818	0.863	0.893	0.897	0.897	*0.890*	0.888	*0.895*	*0.887*	0.912	0.765
**NR-PPAR gamma**	0.962	0.891	0.900	0.924	0.927	0.935	*0.825*	0.924	*0.709*	*0.958*	0.923	0.297
**SR-ARE**	0.669	0.396	0.441	0.456	0.576	0.742	*0.408*	0.446	*0.421*	*0.390*	0.541	0.275
**SR-ATAD5**	0.212	0.585	0.254	0.251	0.319	0.588	*0.087*	0.183	*0.101*	*0.120*	0.113	0.118
**SR-HSE**	0.818	0.576	0.567	0.537	0.691	0.727	*0.557*	0.525	*0.569*	*0.451*	0.670	0.128
**SR-MMP**	0.970	0.931	0.922	0.918	0.911	0.937	*0.935*	0.907	*0.923*	*0.941*	0.970	0.942
**SR-p53**	0.542	0.375	0.226	0.203	0.369	0.564	*0.177*	0.167	*0.165*	*0.2026*	0.474	0.130
**Non Biodegr. Rule***	0	0	0	0	0	0	*0*	0	*0*	*0*	0	0
**Non Genotoxic Carcinogenicity Rule***	0	0	0	0	0	0	*0*	0	*0*	*0*	1	0
**Sure ChEMBL Rule***	0	0	0	0	0	1	*0*	0	*0*	*0*	0	0
**LD_{50} Oral Toxicity**	0	0	0	0	0	0	*0*	0	*0*	*0*	0	0
**Skin Sensitivity Rule***	0	0	0	0	2	2	*0*	1	*0*	*0*	1	0
**Aquatic Toxicity Rule***	1	1	1	1	1	1	*1*	1	*1*	*1*	3	1
**Acute Toxicity Rule***	0	0	0	0	1	1	*1*	1	*1*	*1*	4	1
**FAF Drugs4 Rule***	0	0	0	0	1	0	*0*	0	*0*	*0*	1	0
**GCM***	0	0	0	0	1	0	*0*	0	*0*	*0*	4	0

Table S4. ADMETox data for oleanolic acid (**1**) and its derivatives **3a – 3k**. **Legend: For values given in a range 0.000 – 1.000** (unless stated otherwise)**: 0.000 – 0.300** = low probability, **0.301 – 0.699** = moderate probability, **0.700 – 1.000** = high probability; * = alerts; **Optimal values: Physicochemical Properties: Molecular Weight**: 100 ~ 600; **nHA**: 0 ~ 12; **nHD**: 0 ~ 7; **nRot**: 0 ~ 11; **nRing**: 0 ~ 6; **MaxRing**: 0 ~ 18; **nHet**: 1 ~ 15; **fChar**: -4 ~ 4; **nRig**: 0 ~ 30; **Stereo Centers**: ≤ 2; **TPSA**: 0 ~ 140; **logS**: -4 ~ 0.5; **logP**: 0 ~ 3; **logD**: 1 ~ 3. **Medicinal Chemistry**: **QED**: > 0.67; easy to synthesize; **Fsp^3**: ≥ 0.42; **MCE-18**: ≥ 4; **Npscore**: -5 ~ 5; **ALARM NMR**: 0 **BMS**: 0; **Chelator Rule**: 0; **PAINS**: 0; **Lipinski Rule**: 1. **Pfizer Rule**: 1. **GSK Rule**: 1. **Golden Triangle**: 1. **Absorption**: **Caco-2 Permeability**: > -5.15;. **MDCK Permeability**: high passive permeability: > 20 × 10^{-6} cm/s; **Pgp-inhibitor**: > 0.700; **Pgp-substrate**: > 0.700; **HIA**: > 0.700; **F$_{20\%}$**: > 0.700; **F$_{30\%}$** : > 0.700. **Distribution: BBB Penetr.**: > 0.700; **PPB: < 90%; VD: 0.04 – 20 L/kg; Fu**: **high: > 20%. Metabolism: CYP1A2 inhibitor, CYP1A2 substrate, etc.:** > 0.700. **Excretion**: **CL**: >15 mL/min/kg; **T$_{1/2}$** long half-life >3h: > 0.700. **Toxicity: hERG Blockers, H-HT, etc: <** 0.300. **Environmental toxicity**: BCF, IG$_{50}$, etc: informative values. **Tox21 pathway: NR-AhR**, **NR-AR**, etc: < 0.300. **Toxicophore Rules**: Non Biodeg. Rule, et.: 0. **Explanation of parameters**: https://admetmesh.scbdd.com/explanation/index

High value (optimal)	Moderate value	Low value	Neutral value

Physicochemical Properties (optimal values)	Compound number											
	1 (OA)	3a	3b	3c	3d	3e	*3f*	3g	*3h*	*3i*	3j	3k
Molecular Weight	456.360	564.380	578.400	592.410	590.400	588.380	*592.410*	606.430	*606.430*	*606.430*	660.330	630.390
Volume	505.750	610.642	627.938	645.234	642.598	639.961	*645.234*	662.530	*662.530*	*662.530*	675.656	674.787
Density	0.902	0.924	0.921	0.918	0.919	0.919	*0.918*	0.915	*0.915*	*0.915*	0.977	0.934
NHA	3	5	5	5	5	5	*5*	5	*5*	*5*	5	6
nHD	2	0	0	0	0	0	*0*	0	*0*	*0*	0	0
nRot	1	5	6	7	7	6	*6*	8	*7*	*7*	8	7
nRing	5	6	6	6	6	6	*6*	6	*6*	*6*	6	7
maxRing	22	33	22	22	22	22	*22*	22	*22*	*22*	22	22
nHet	3	5	5	5	5	5	*5*	5	*5*	*5*	7	6
fChar	0	0	0	0	0	0	*0*	0	*0*	*0*	0	0
nRig	27	33	33	33	34	34	*33*	33	*33*	*33*	33	38
Flexibility (≤ 2)	0.037	0.152	0.182	0.212	0.206	0.176	*0.182*	0.242	*0.212*	*0.212*	0.242	0.184
Stereo Centers	8	8	8	8	8	8	*8*	8	*8*	*9*	9	8
TPSA (0 ~ 140)	57.530	65.74	65.740	65.740	65.740	65.740	*65.740*	65.740	*65.740*	*65.740*	65.740	78.880
LogS (-4 ~ 0.5)	-4.933	-7.140	-7.251	-7.379	-7.230	-7.308	*-7.254*	-7.457	*-7.114*	*-7.433*	-6.995	-7.209
LogD (1 ~ 3)	4.781	5.494	5.642	5.744	5.572	5.638	*5.866*	5.916	*6.085*	*5.866*	5.919	5.816
LogP (0 ~ 3)	6.370	7.594	7.832	8.148	8.102	7.493	*7.981*	8.554	*8.440*	*8.389*	8.104	8.542
QED (> 0.67)	0.409	0.271	0.263	0.252	0.252	0.200	*0.257*	0.183	*0.247*	*0.246*	0.173	0.242

SAscore	4.589	4.746	4.753	4.797	4.870	4.933	4.845	4.976	4.838	4.960	5.109	4.932
Fsp3 (≥ 0.420)	0.900	0.778	0.784	0.789	0.737	0.737	0.789	0.795	0.795	0.795	0.789	0.700
MCE-18 (≥ 45)	105.368	159.5	158.667	157.882	158.667	158.667	161.471	157.143	160.714	160.714	161.471	172.235
Npscore (-5 ~ 5)	3.272	2.518	2.373	2.344	2.376	2.217	2.259	2.328	2.334	2.233	2.366	2.193
ALARM NMR*	0	0	0	0	0	0	0	0	0	0	0	0
BMS*	0	0	0	0	0	0	0	0	0	0	2	0
Chelator Rule*	0	0	0	0	0	0	0	0	0	0	0	0
PAINS*	0	0	0	0	0	0	0	0	0	0	0	0
Lipinski Rule	1	0	0	0	0	0	0	0	0	0	0	0
Pfizer Rule	0	0	0	0	0	0	0	0	0	0	0	0
GSK Rule	0	0	0	0	0	0	0	0	0	0	0	0
Golden Triangle	1	0	0	0	0	0	0	0	0	0	0	0
Caco-2 Permeability	-5.216	-4.713	-4971	-4.975	-4.934	-4.974	-4.960	-4.983	-5.026	-4.937	-5.033	-4.992
MDCK Permeability	1.2×10^{-5}	1.7×10^{-5}	1.6×10^{-5}	1.5×10^{-5}	1.9×10^{-5}	1.6×10^{-5}	1.5×10^{-5}	1.4×10^{-5}	1.4×10^{-5}	1.4×10^{-5}	1.5×10^{-5}	1.6×10^{-5}
Pgp Inhibitor	0.000	0.979	0.973	0.945	0.958	0.985	0.978	0.856	0.973	0.977	0.819	0.990
Pgp Substrate	0.000	0.000	0.000	0.000	0.000	0.000	0.000	0.000	0.000	0.000	0.000	0.000
HIA	0.016	0.007	0.006	0.006	0.007	0.016	0.006	0.006	0.016	0.005	0.015	0.009
$F_{20\%}$	0.125	0.551	0.871	0.925	0.121	0.869	0.424	0.948	0.824	0.440	0.518	0.911
$F_{30\%}$	0.874	0.763	0.623	0.780	0.138	0.710	0.505	0.843	0.656	0.631	0.422	0.630
BBB penetration	0.577	0.024	0.018	0.020	0.012	0.049	0.020	0.020	0.016	0.024	0.036	0.003
PPB	96.976	95.951	96.585	96.497	98.014	96.852	97.305	96.539	96.489	96.725	99.819	96.890
VDss	0.707	1.004	1.904	1.954	1.338	1.791	2.013	2.062	1.991	2.023	2.096	2.667
Fu (> 20%)	4.389	5.694	4.972	4.385	5.922	2.279	4.777	3.883	3.993	4.521	3.431	5.292
CYP1A2 Inhibitor	0.014	0.032	0.030	0.026	0.031	0.041	0.020	0.025	0.017	0.022	0.026	0.023
CYP1A2 Substrate	0.307	0.203	0.156	0.150	0.152	0.193	0.144	0.151	0.143	0.132	0.153	0.125
CYP2C19 Inhibitor	0.026	0.129	0.158	0.148	0.126	0.261	0.150	0.153	0.129	0.121	0.123	0.149
CYP2C19 Substrate	0.901	0.937	0.916	0.911	0.895	0.908	0.888	0.901	0.915	0.916	0.915	0.724
CYP2C9 Inhibitor	0.179	0.207	0.201	0.159	0.172	0.463	0.138	0.139	0.118	0.129	0.152	0.128
CYP2C9 Substrate	0.758	0.492	0.407	0.480	0.564	0.444	0.328	0.491	0.387	0.368	0.539	0.323
CYP2D6 Inhibitor	0.012	0.156	0.267	0.222	0.333	0.437	0.088	0.250	0.064	0.109	0.372	0.461
CYP2D6 Substrate	0.256	0.231	0.125	0.135	0.383	0.081	0.039	0.104	0.070	0.098	0.381	0.070
CYP3A4 Inhibitor	0.242	0.766	0.745	0.759	0.823	0.852	0.593	0.759	0.584	0.603	0.794	0.864
CYP3A4 Substrate	0.227	0.736	0.733	0.690	0.759	0.846	0.715	0.682	0.723	0.765	0.786	0.719
CL	3.813	9.937	9.760	9.953	10.116	7.749	10.093	9.455	9.838	9.332	8.969	9.175
T1/2 (< 3h)	0.021	0.004	0.002	0.002	0.002	0.002	0.003	0.002	0.002	0.002	0.003	0.003

hERG Blockers	0.006	0.060	0.074	0.074	0.021	0.035	0.087	0.109	0.039	0.045	0.041	0.128
DILI	0.011	0.046	0.044	0.044	0.040	0.077	0.048	0.045	0.060	0.070	0.052	0.100
AMES Toxicity	0.006	0.008	0.008	0.009	0.012	0.020	0.009	0.007	0.01	0.006	0.030	0.008
ROA Toxicity	0.264	0.432	0.389	0.400	0.420	0.615	0.404	0.374	0.421	0.346	0.391	0.500
FDAMDD	0.914	0.682	0.638	0.657	0.756	0.856	0.566	0.656	0.590	0.766	0.701	0.733
Skin Sensitivity	0.046	0.034	0.046	0.054	0.079	0.072	0.041	0.068	0.059	0.041	0.041	0.024
Carcinogenicity	0.095	0.104	0.117	0.109	0.285	0.284	0.141	0.102	0.124	0.133	0.228	0.160
Eye Corrosion	0.030	0.004	0.003	0.003	0.003	0.003	0.004	0.003	0.003	0.004	0.003	0.003
Eye Irritation	0.066	0.031	0.093	0.104	0.158	0.091	0.031	0.099	0.159	0.026	0.071	0.145
Respiratory Toxicity	0.974	0.970	0.948	0.931	0.864	0.956	0.966	0.927	0.932	0.952	0.922	0.960
H-HT	0.346	0.290	0.149	0.160	0.082	0.232	0.207	0.164	0.146	0.228	0.168	0.152
BCF	1.815	2.222	2.214	1.649	2.289	1.758	2.313	1.521	1.930	2.085	1.995	1.545
IGC$_{50}$	4.971	5.420	5.495	5.563	5.480	5.468	5.460	5.668	5.614	5.494	5.616	5.649
LC$_{50}$DM	6.345	6.797	6.810	6.802	6.813	6.910	6.709	6.806	6.764	6.822	6.852	6.920
LC$_{50}$FM	5.884	6.459	6.569	6.584	6.930	6.883	6.390	6.648	6.654	6.453	6.604	6.642
NR-AhR	0.001	0.001	0.001	0.001	0.001	0.001	0.001	0.001	0.000	0.000	0.001	0.001
NR-AR	0.369	0.516	0.037	0.044	0.088	0.033	0.034	0.036	0.022	0.215	0.030	0.026
NR-AR-LBD	0.479	0.862	0.830	0.857	0.901	0.899	0.695	0.838	0.581	0.861	0.854	0.820
NR-Aromatase	0.798	0.541	0.550	0.515	0.475	0.548	0.523	0.496	0.474	0.501	0.588	0.466
NR-ER	0.672	0.833	0.744	0.857	0.862	0.898	0.760	0.822	0.804	0.760	0.872	0.738
NR-ER-LBD	0.746	0.884	0.917	0.935	0.933	0.936	0.933	0.928	0.933	0.934	0.947	0.898
NR-PPAR gamma	0.962	0.935	0.916	0.888	0.923	0.936	0.842	0.860	0.548	0.941	0.858	0.125
SR-ARE	0.669	0.378	0.428	0.427	0.546	0.725	0.388	0.414	0.404	0.379	0.536	0.251
SR-ATAD5	0.212	0.705	0.460	0.464	0.519	0.701	0.200	0.380	0.235	0.268	0.250	0.404
SR-HSE	0.818	0.652	0.672	0.649	0.722	0.737	0.652	0.652	0.682	0.596	0.659	0.529
SR-MMP	0.970	0.866	0.834	0.814	0.828	0.872	0.857	0.781	0.819	0.864	0.922	0.795
SR-p53	0.542	0.273	0.157	0.135	0.263	0.408	0.119	0.105	0.107	0.138	0.349	0.062
Non Biodeg. Rule*	0	0	0	0	0	0	0	0	0	0	0	0
Non Genotoxic Carcinogenicity Rule*	0	0	0	0	0	0	0	0	0	0	1	0
Sure ChEMBL Rule*	0	0	0	0	0	1	0	0	0	0	0	0
LD$_{50}$ Oral Toxicity	0	0	0	0	0	0	0	0	0	0	0	0
Skin Sensit. Rule*	0	0	0	0	0	2	0	0	0	0	1	0
Aquatic Tox. Rule*	1	0	0	0	0	0	0	0	0	0	2	0
Acute Tox. Rule*	0	0	0	0	0	0	0	0	0	0	0	0

**FAF Drugs4 Rule***	0	0	0	0	0	1	*1*	1	*1*	*1*	2	1
**GCM***	0	0	0	0	1	0	*0*	0	*0*	*0*	4	0

Article

Short-Chain Oleanolic Acid Esters and Furoyl Hybrids: Pharmacological Prediction, ADMETox Profiling, In Vitro Cytotoxicity Evaluation, Antioxidant Testing and EGFR Docking

Barbara Bednarczyk-Cwynar[1,2,*], Piotr Ruszkowski[3], Maciej Kulawik[4,5], Szymon Sip[4], Przemysław Zalewski[4], Dobrosława Wiśniewska[1], Andrzej Günther[1]

Supplementary Materials

Figure S3. Standard curve for (**A**) CUPRAC and (**B**) DPPH assay as Trolox equivalent.

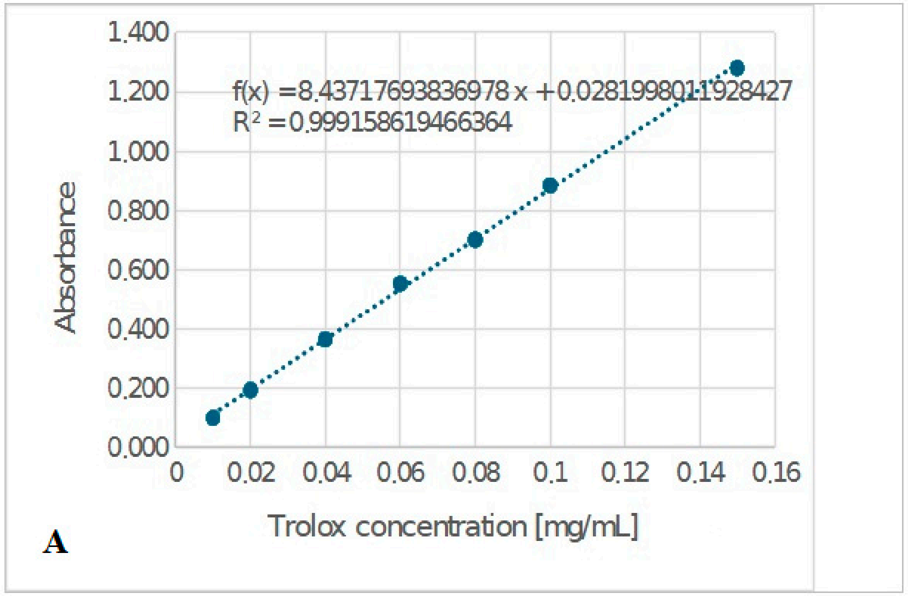

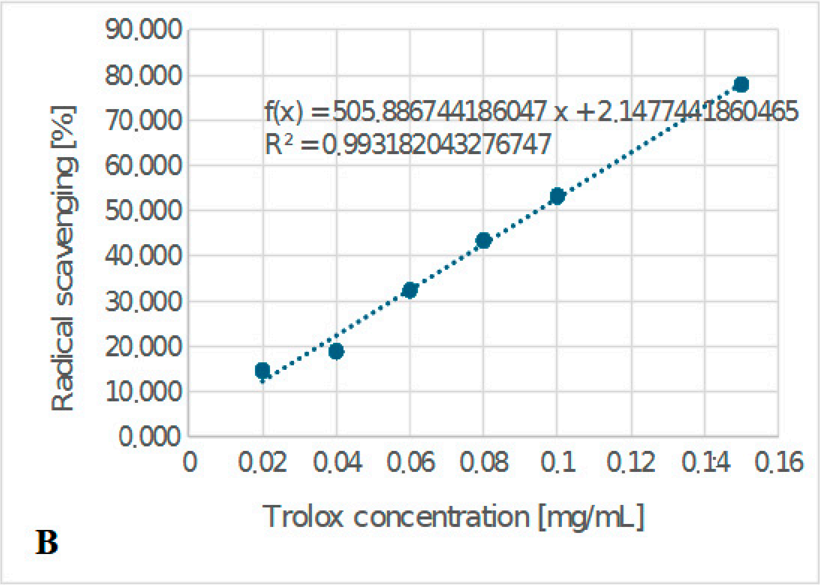

Article

Short-Chain Oleanolic Acid Esters and Furoyl Hybrids: Pharmacological Prediction, ADMETox Profiling, In Vitro Cytotoxicity Evaluation, Antioxidant Testing and EGFR Docking

Barbara Bednarczyk-Cwynar[1,2,*], Piotr Ruszkowski[3], Maciej Kulawik[4,5], Szymon Sip[4], Przemysław Zalewski[4], Dobrosława Wiśniewska[1], Andrzej Günther[1]

Supplementary Materials

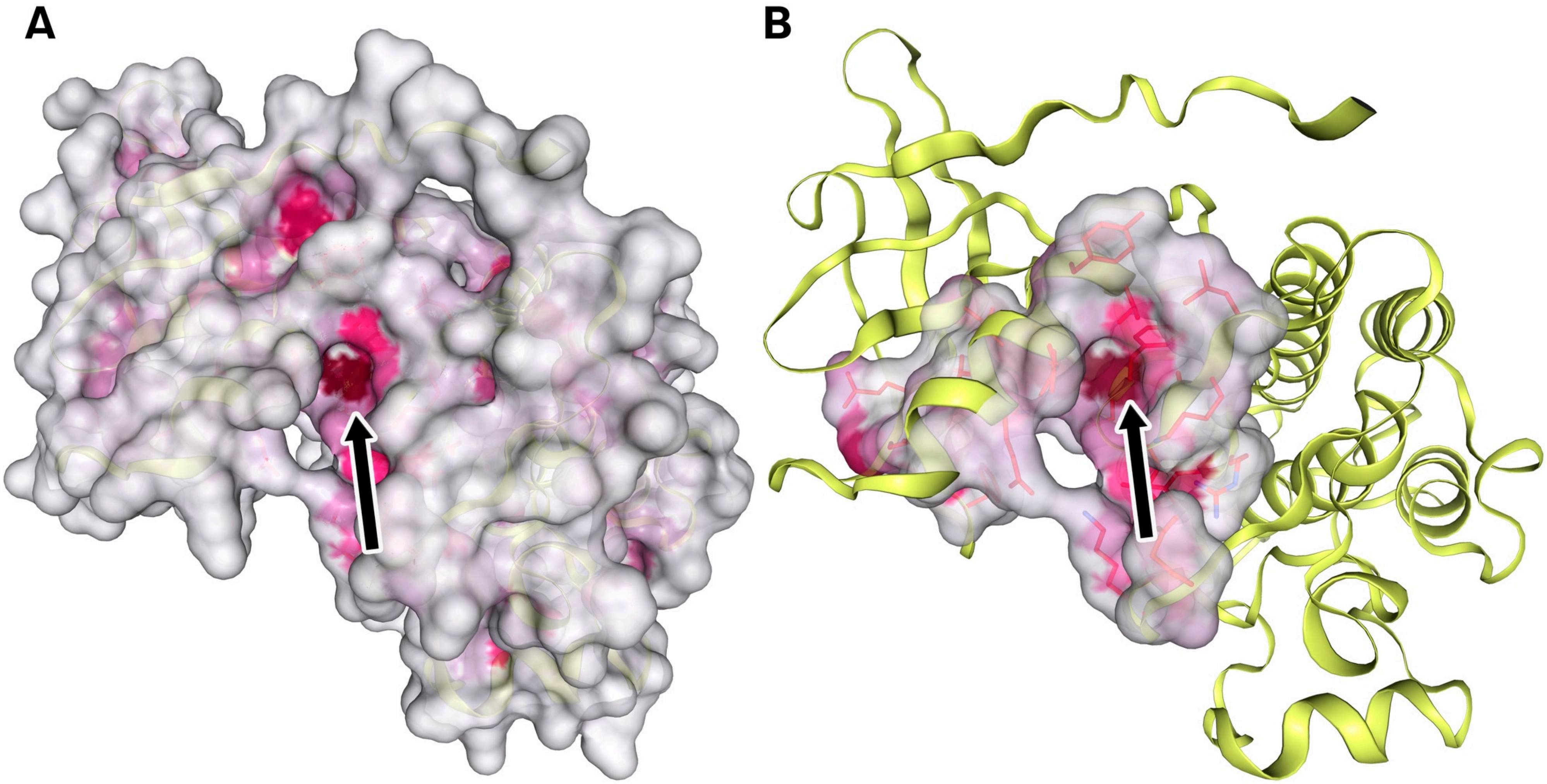

Figure S4 A. Whole 1M17 molecule, the dark pink color indicates the cavity of the molecule. **B**. View of the largest pocket of C1 with a volume of 991 Å3 indicated by a black arrow.

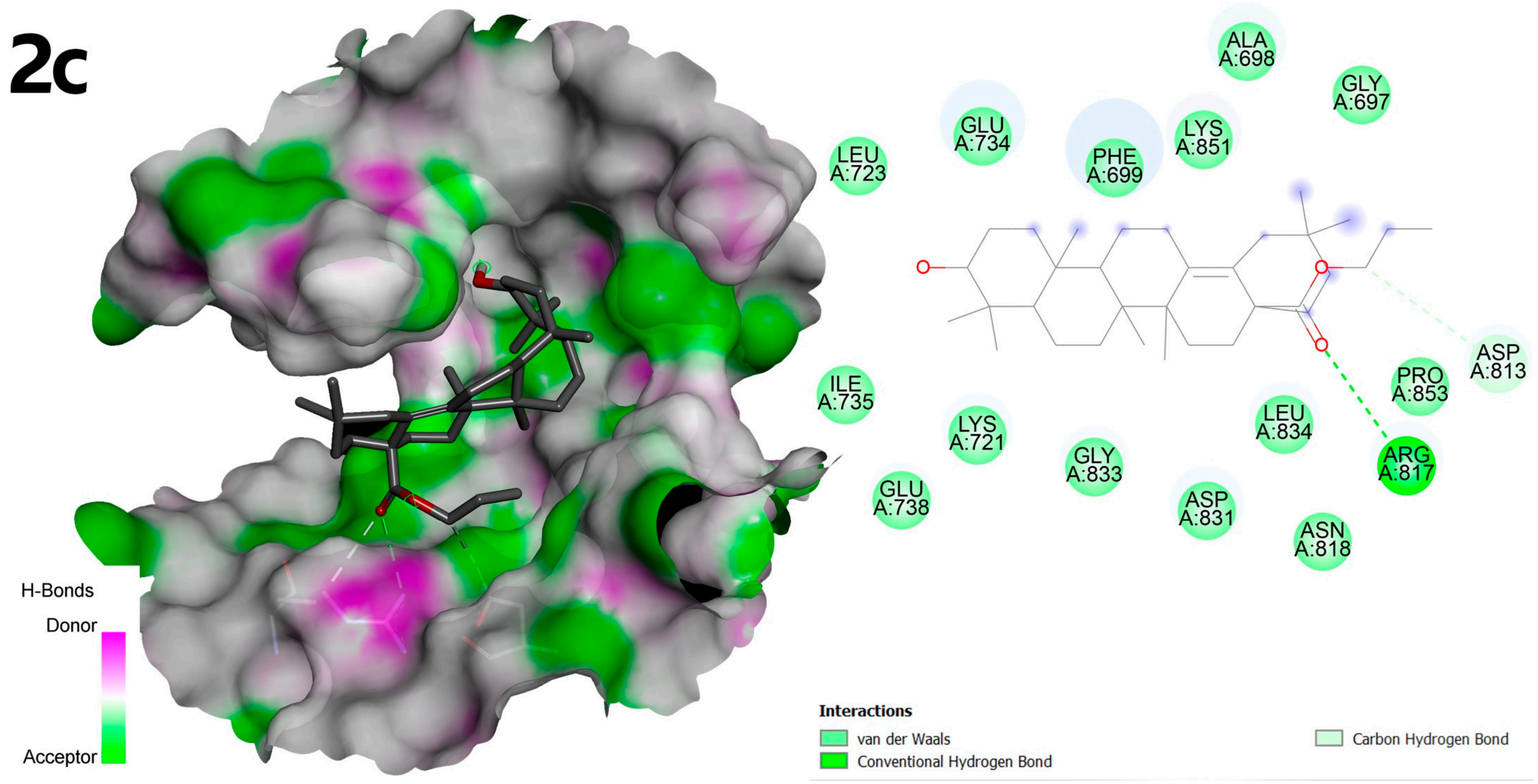

Figure S5. 3D surface of structure 1M17 with ligand compound **2c** with H-Bonds gradient map in C1 pocket and 2D Diagram with interactions.

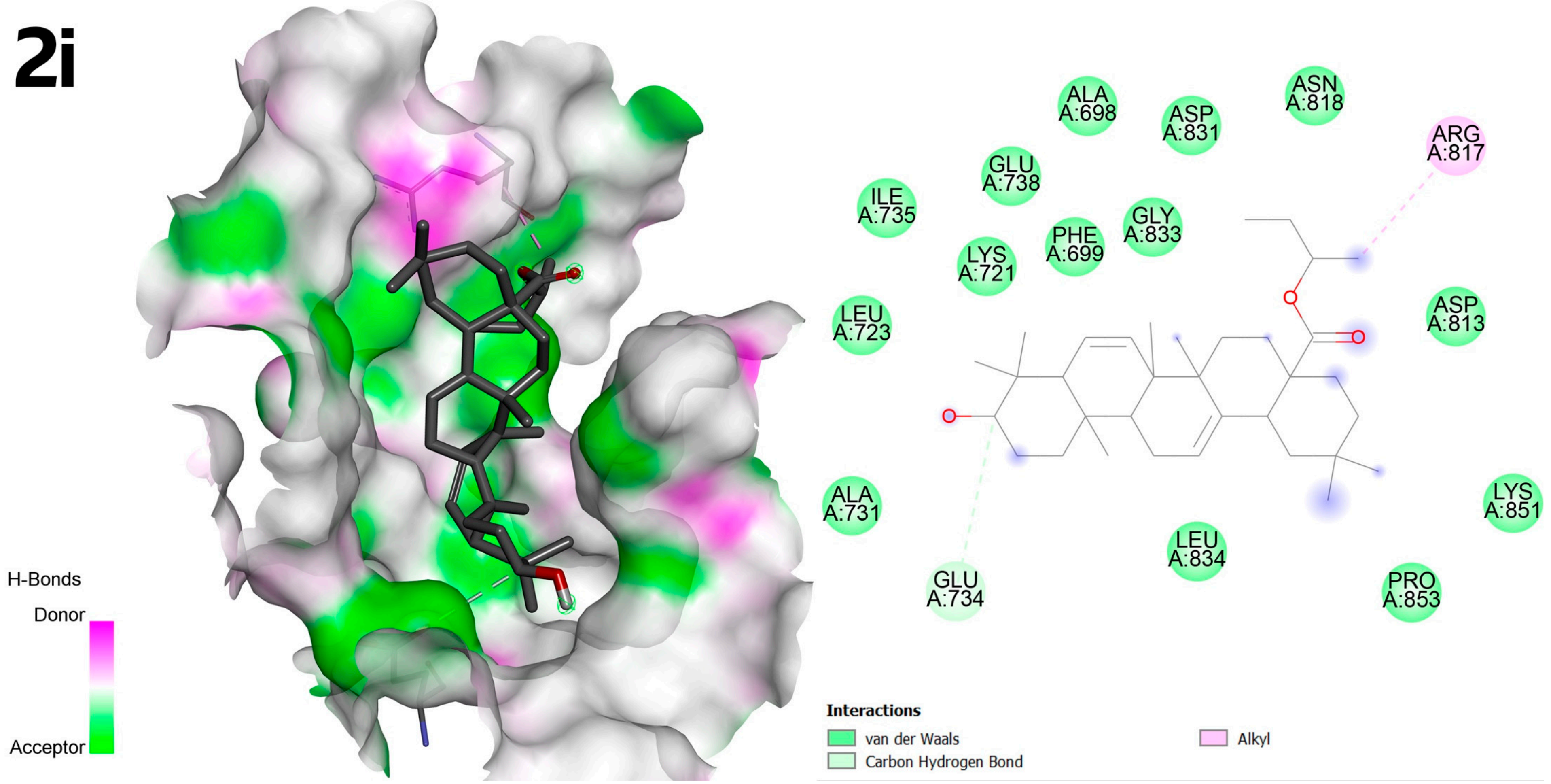

Figure S6. 3D surface of structure 1M17 with ligand compound **2i** with H-Bonds gradient map in C1 pocket and 2D Diagram with interactions.

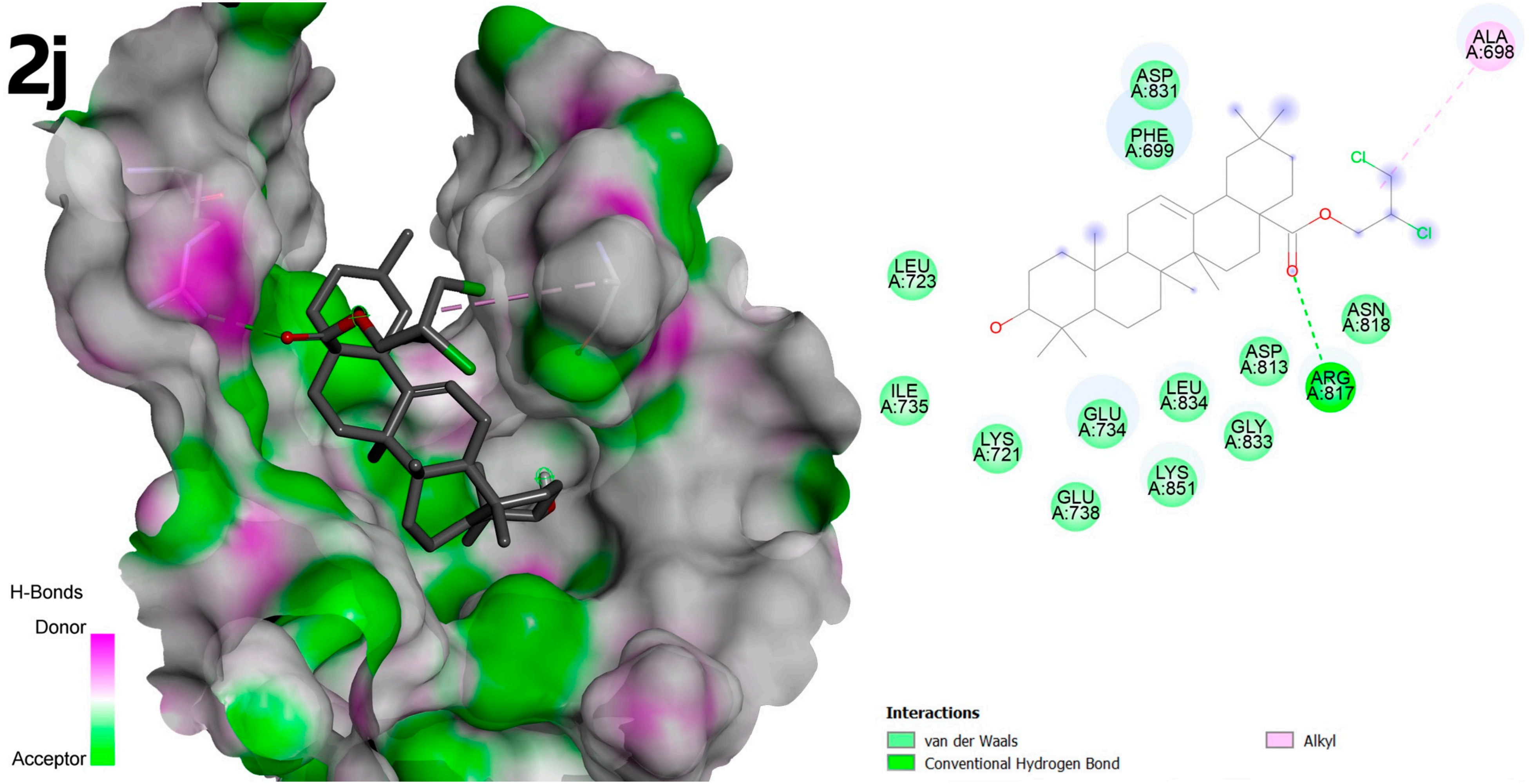

Figure S7. 3D surface of structure 1M17 with ligand compound **2j** with H-Bonds gradient map in C1 pocket and 2D Diagram with interactions.

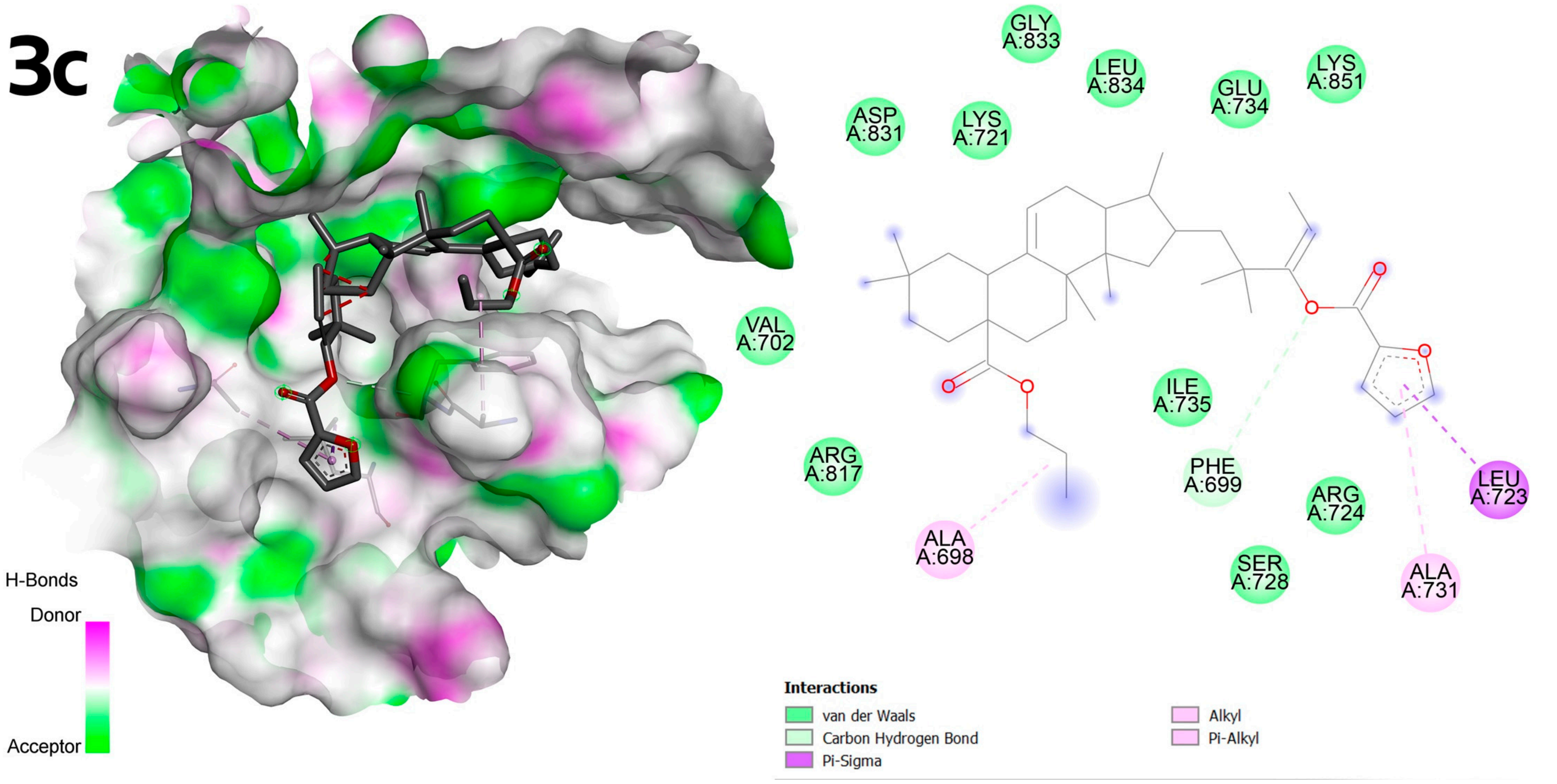

Figure S8. 3D surface of structure 1M17 with ligand compound **3c** with H-Bonds gradient map in C1 pocket and 2D Diagram with interactions.

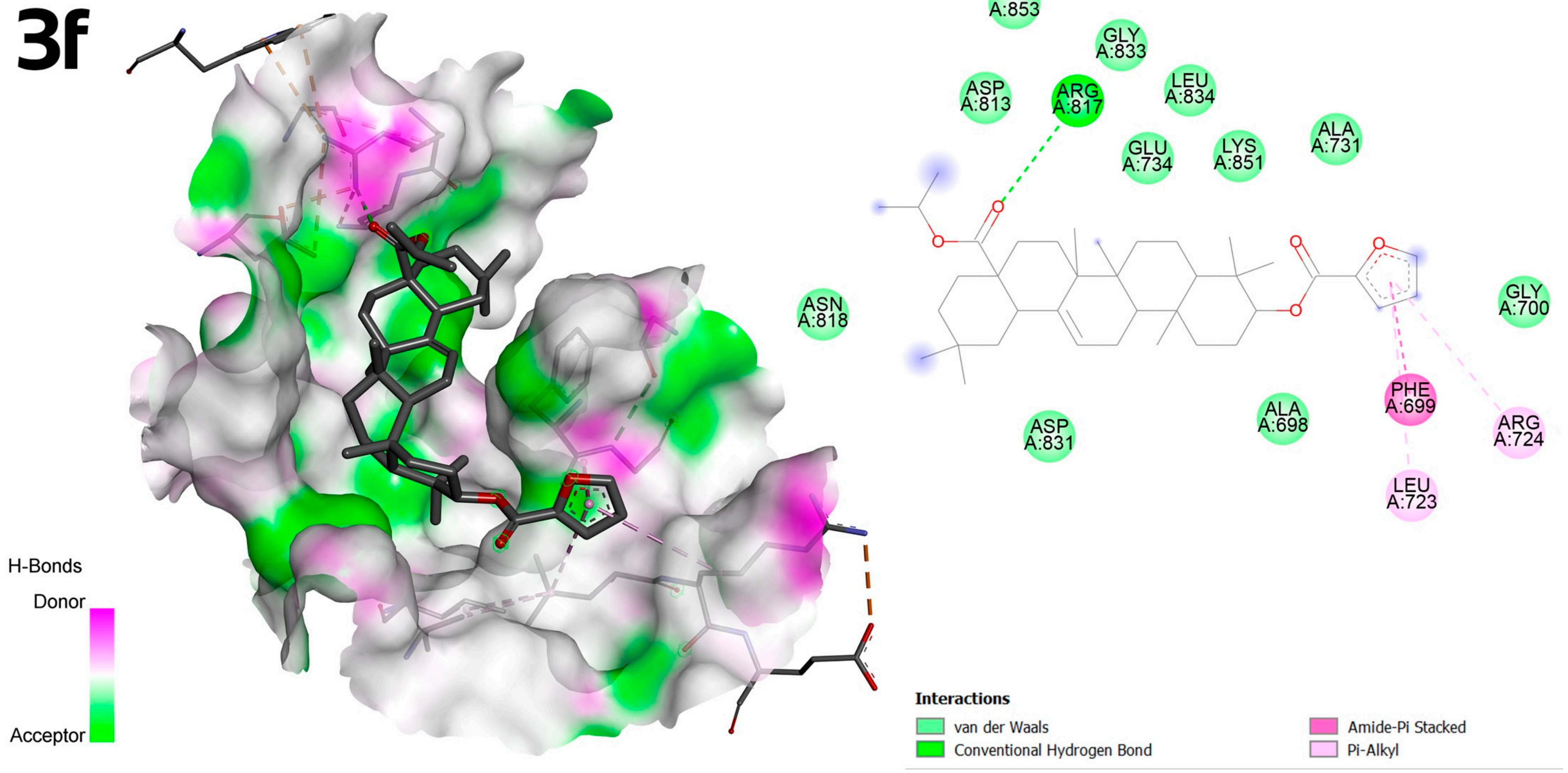

Figure S9. 3D surface of structure 1M17 with ligand compound 3f with H-Bonds gradient map in C1 pocket and 2D Diagram with interactions.

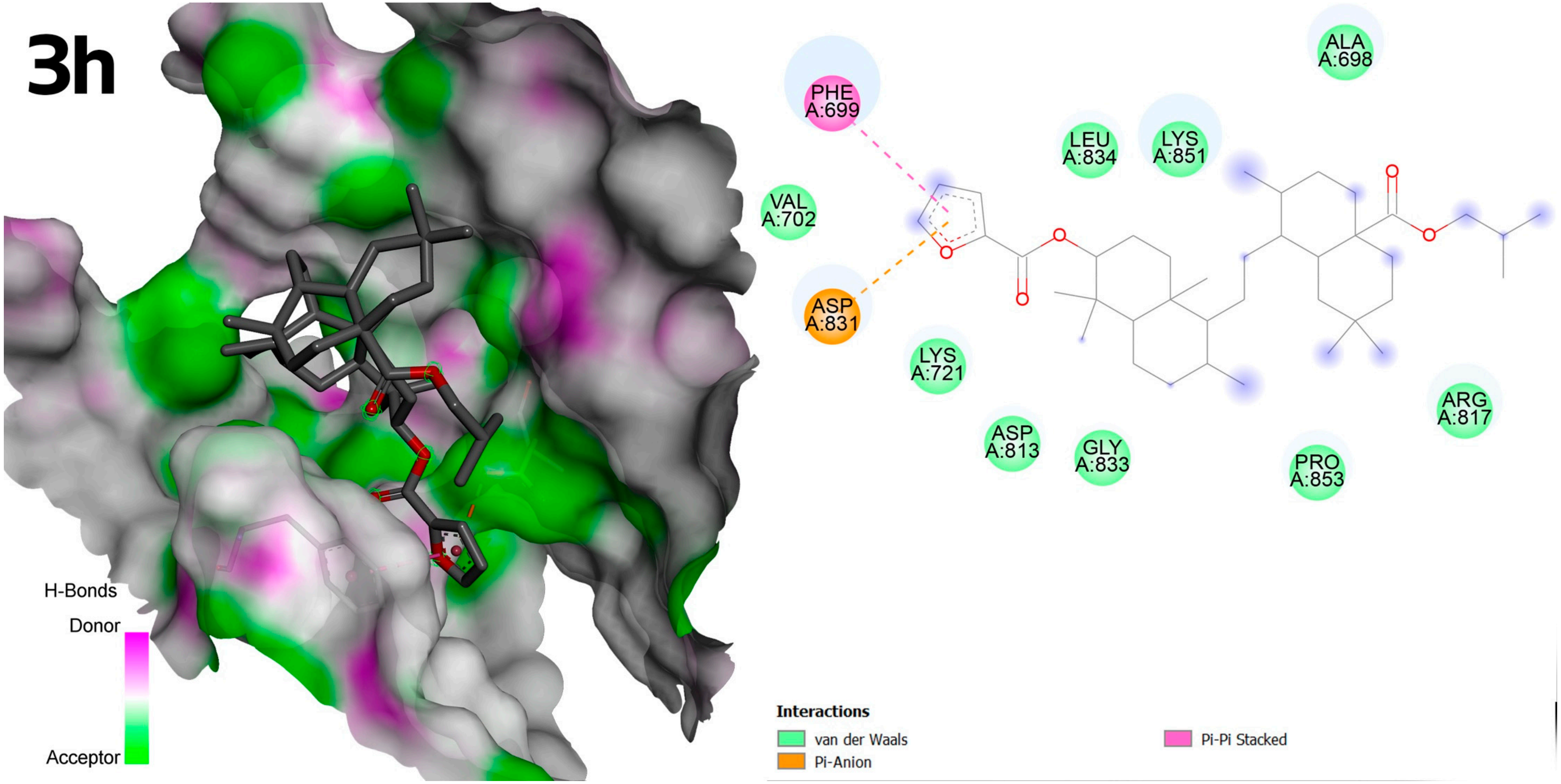

Figure S10. 3D surface of structure 1M17 with ligand compound **3h** with H-Bonds gradient map in C1 pocket and 2D Diagram with interactions.

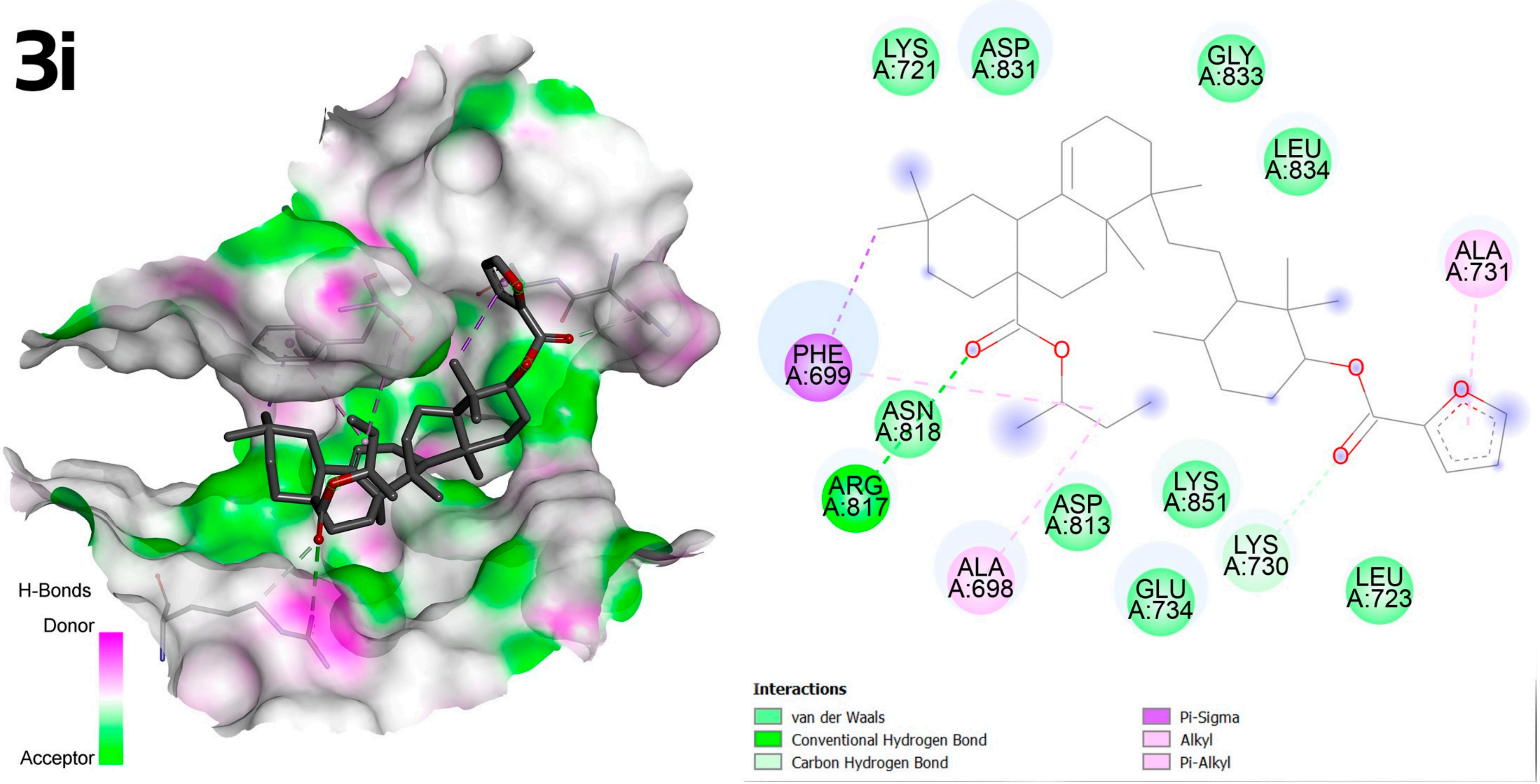

Figure S11. 3D surface of structure 1M17 with ligand compound **3i** with H-Bonds gradient map in C1 pocket and 2D Diagram with interactions.

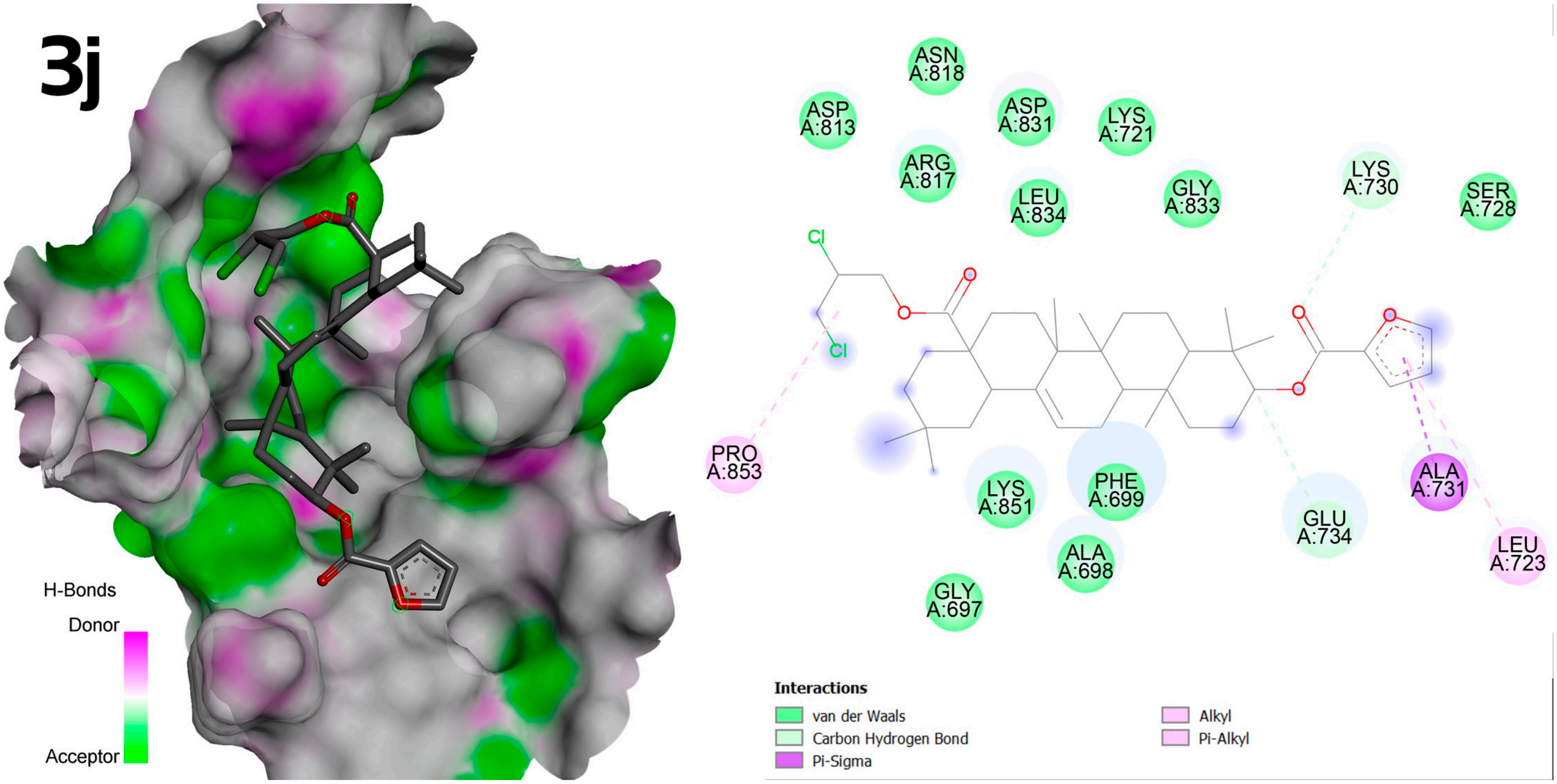

Figure S12. 3D surface of structure 1M17 with ligand compound **3j** with H-Bonds gradient map in C1 pocket and 2D Diagram with interactions.

Table S5. Data compiled from CB-Dock2 outputs for pocket C1 (volume 991 Å^3; center 36, 8, 51).

Comp. no.	Vina score (kcal x mol^{-1})	Cavity volume (Å^3)	Center (x, y, z)	Docking box size (x, y, z)
**2c**	-7.5	991	36, 8, 51	23, 23, 23
**2i**	-8.5	991	36, 8, 51	23, 23, 23
**2j**	-7.5	991	36, 8, 51	24, 24, 24
**3c**	-8.3	991	36, 8, 51	24, 24, 24
**3f**	-7.8	991	36, 8, 51	25, 25, 25
**3h**	-7.3	991	36, 8, 51	23, 23, 23
**3i**	-7.4	991	36, 8, 51	25, 25, 25
**3j**	-7.2	991	36, 8, 51	26, 26, 26
